# Health, social care and technological interventions to improve functional ability of older adults living at home: An evidence and gap map

**DOI:** 10.1002/cl2.1175

**Published:** 2021-07-07

**Authors:** Vivian Welch, Christine M. Mathew, Panteha Babelmorad, Yanfei Li, Elizabeth T. Ghogomu, Johan Borg, Monserrat Conde, Elizabeth Kristjansson, Anne Lyddiatt, Sue Marcus, Jason W. Nickerson, Kevin Pottie, Morwenna Rogers, Ritu Sadana, Ashrita Saran, Beverly Shea, Lisa Sheehy, Heidi Sveistrup, Peter Tanuseputro, Joanna Thompson‐Coon, Peter Walker, Wei Zhang, Tracey E. Howe

**Affiliations:** ^1^ Methods Centre Bruyère Research Institute Ottawa Canada; ^2^ Bruyère Research Institute University of Ottawa Ottawa Canada; ^3^ University of Ottawa Ottawa Canada; ^4^ Evidence‐Based Social Science Research Center, School of Public Health Lanzhou University Lanzhou China; ^5^ Lund University Malmo Sweden; ^6^ Cochrane Campbell Global Ageing Partnership Field Faro Portugal; ^7^ Faculty of Social Sciences, School of Psychology University of Ottawa Ottawa Canada; ^8^ Independent Consultant Ingersoll Canada; ^9^ Radcliffe Department of Medicine University of Oxford Oxford UK; ^10^ Institute of Population Health Ottawa Canada; ^11^ Family Medicine University of Ottawa Ottawa Canada; ^12^ NIHR ARC, South West Peninsula (PenARC) University of Exeter Medical School Exeter UK; ^13^ World Health Organization Geneva Switzerland; ^14^ Campbell Collaboration Delhi India; ^15^ Faculty of Health Sciences University of Ottawa Ottawa Canada; ^16^ Ottawa Hospital Research Institute Ottawa Canada; ^17^ NIHR ARC South West Peninsula (PenARC) University of Exeter Medical School Exeter UK; ^18^ Faculty of Medicine University of Ottawa Ottawa Canada; ^19^ Access to Medicines, Vaccines and Health Products World Health Organization Geneva Switzerland; ^20^ City of Glasgow College Glasgow UK

## Abstract

**Background:**

By 2030, the global population of people older than 60 years is expected to be higher than the number of children under 10 years, resulting in major health and social care system implications worldwide. Without a supportive environment, whether social or built, diminished functional ability may arise in older people. Functional ability comprises an individual's intrinsic capacity and people's interaction with their environment enabling them to be and do what they value.

**Objectives:**

This evidence and gap map aims to identify primary studies and systematic reviews of health and social support services as well as assistive devices designed to support functional ability among older adults living at home or in other places of residence.

**Search Methods:**

We systematically searched from inception to August 2018 in: MEDLINE, EMBASE, Cochrane Database of Systematic Reviews, CENTRAL, CINAHL, PsycINFO, AgeLine, Campbell Library, ASSIA, Social Science Citation Index and Social Policy & Practice. We conducted a focused search for grey literature and protocols of studies (e.g., ProQuest Theses and Dissertation Global, conference abstract databases, Help Age, PROSPERO, Cochrane and Campbell libraries and ClinicalTrials.gov).

**Selection Criteria:**

Screening and data extraction were performed independently in duplicate according to our intervention and outcome framework. We included completed and on‐going systematic reviews and randomized controlled trials of effectiveness on health and social support services provided at home, assistive products and technology for personal indoor and outdoor mobility and transportation as well as design, construction and building products and technology of buildings for private use such as wheelchairs, and ramps.

**Data Collection and Analysis:**

We coded interventions and outcomes, and the number of studies that assessed health inequities across equity factors. We mapped outcomes based on the International Classification of Function, Disability and Health (ICF) adapted categories: intrinsic capacities (body function and structures) and functional abilities (activities). We assessed methodological quality of systematic reviews using the AMSTAR II checklist.

**Main Results:**

After de‐duplication, 10,783 records were screened. The map includes 548 studies (120 systematic reviews and 428 randomized controlled trials). Interventions and outcomes were classified using domains from the International Classification of Function, Disability and Health (ICF) framework. Most systematic reviews (*n* = 71, 59%) were rated low or critically low for methodological quality.

The most common interventions were home‐based rehabilitation for older adults (*n* = 276) and home‐based health services for disease prevention (*n* = 233), mostly delivered by visiting healthcare professionals (*n* = 474). There was a relative paucity of studies on personal mobility, building adaptations, family support, personal support and befriending or friendly visits. The most measured intrinsic capacity domains were mental function (*n* = 269) and neuromusculoskeletal function (*n* = 164). The most measured outcomes for functional ability were basic needs (*n* = 277) and mobility (*n* = 160). There were few studies which evaluated outcome domains of social participation, financial security, ability to maintain relationships and communication.

There was a lack of studies in low‐ and middle‐income countries (LMICs) and a gap in the assessment of health equity issues.

**Authors' Conclusions:**

There is substantial evidence for interventions to promote functional ability in older adults at home including mostly home‐based rehabilitation for older adults and home‐based health services for disease prevention. Remotely delivered home‐based services are of greater importance to policy‐makers and practitioners in the context of the COVID‐19 pandemic. This map of studies published prior to the pandemic provides an initial resource to identify relevant home‐based services which may be of interest for policy‐makers and practitioners, such as home‐based rehabilitation and social support, although these interventions would likely require further adaptation for online delivery during the COVID‐19 pandemic. There is a need to strengthen assessment of social support and mobility interventions and outcomes related to making decisions, building relationships, financial security, and communication in future studies. More studies are needed to assess LMIC contexts and health equity issues.

**Abbreviations**
EGM: evidence and gap map
ICF framework: International Classification of Function, Disability and Health framework
LGBTQ2+: lesbian, gay, bisexual, transgender, queer (or sometimes questioning), and two‐spirited
LMIC: low‐ and middle‐income countries
PICO: population, intervention, comparison, outcome
RCT: randomized control trials
SR: systematic review
WHO: World Health Organization

## 1 PLAIN LANGUAGE SUMMARY

**[The evidence for health, social care and technological interventions to improve functional ability of older adults are unevenly distributed across intervention areas]**

The evidence for health, social care and mobility interventions to improve functional ability of older adults includes mostly home‐based rehabilitation and health services delivered by visiting healthcare professionals, and is of low or critically low quality.

### 1.1 What is this evidence and gap map (EGM) about?

By 2030, the global population of people older than 60 years is expected to be higher than the number of children under 10 years, resulting in major health and social care system implications worldwide. Without a supportive environment, whether social or built, diminished functional ability may arise in older people.

Functional ability comprises an individual's intrinsic capacity and people's interaction with their environment, enabling them to be and do what they value. This map assesses the evidence on home‐based health and social care as well as mobility interventions to improve functional ability of older adults living at home.

**What is the aim of this evidence and gap map (EGM)?**

The aim of this EGM is to identify primary studies and systematic reviews of health and social support services as well as assistive devices designed to support functional ability among older adults living at home or in other places of residence.

### 1.2 What studies are included?

The EGM includes randomized controlled studies and systematic reviews that assess the effect of interventions to improve functional ability of older adults living at home or in other places of residence. The interventions were classified as home‐based health, social care, and mobility interventions. Impact on body function and structures as well as activities were considered as outcomes.

There are 548 included studies (120 systematic reviews and 428 randomized controlled trials) in the map.

### 1.3 What is the distribution of evidence?

The most common interventions were home‐based rehabilitation for older adults (*n* = 276) and home‐based health services for disease prevention (*n* = 233), mostly delivered by visiting healthcare professionals (*n* = 474).

There was a relative paucity of studies on personal mobility, building adaptations, family support, personal support and befriending or friendly visits.

The most measured intrinsic capacity domains were mental function (*n* = 269) and neuromusculoskeletal function (*n* = 164). The most measured outcomes for functional ability were basic needs (*n* = 277) and mobility (*n* = 160). There were few studies which evaluated outcome domains of social participation, financial security, ability to maintain relationships and communication.

There was a lack of studies in low‐ and middle‐income countries (LMICs) and a gap in the assessment of health equity issues.

### 1.4 What do the findings of the map mean?

There is substantial evidence for interventions to promote functional ability in older adults at home, including mostly home‐based rehabilitation for older adults and home‐based health services for disease prevention. Remotely delivered home‐based services are of greater importance to policy‐makers and practitioners in the context of the COVID‐19 pandemic.

This map of studies published prior to the pandemic provides an initial resource to identify relevant home‐based services which may be of interest for policymakers and practitioners, such as home‐based rehabilitation and social support, although these interventions would likely require further adaptation for online delivery during the COVID‐19 pandemic.

There is need to strengthen assessment of social support and mobility interventions and outcomes related to making decisions, building relationships, financial security and communication in future studies.

More studies are needed to assess LMIC contexts and health equity issues.

### 1.5 How up‐to‐date is this EGM?

The authors searched for studies up to August 2018.

## 2 BACKGROUND

### 2.1 Introduction

#### 2.1.1 The problem, condition or issue

There is an increasing proportion of older adults in the global population, with UN population projections predicting that before 2020, people aged >65 years will outnumber children aged <10 years for the first time in history (UNDESA, 2017). LMICs such as China and India are expected to experience a rapid rise in population ageing, compared to Western Europe (UNDESA, 2017). Currently, over two‐thirds of people over 65 years of age are living with multimorbidities (Banerjee, 2015). When combined with parallel increases in disparities to health care and broader determinants of health (Sadana et al., 2016), there are major implications for health and social care systems (Beard et al., 2016; Chatterji et al., 2015; Prince et al., 2015). While many nations are becoming wealthy with the influx of global socioeconomic developments, many countries, especially LMICs, have experienced increasing health and social disparities, especially among older adults (World Health Organization [WHO], 2015). Older adults with the greatest health needs are also often those with the fewest resources to support them (Beard et al., 2016). For example, older adults in LMICs have poor access to assistive technologies and medical devices, as a result of a confluence of factors that affect the availability and accessibility of these products in local markets, including affordability and appropriateness. These factors can influence their integration into health and social systems (Garçon et al., 2016; Marasinghe et al., 2015). Furthermore, the privatization of health and social services becomes a barrier to quality of care if costs impact access to appropriate and timely care for older adults.

Functional ability is complex and comprises an individual's intrinsic capacity and people's interaction with their environment enabling them to be and do what they value (Cesari et al., 2018; WHO, 2015). The WHO considers intrinsic capacity to include the physical and mental capacities of a person. The environment defined by the WHO, includes all factors in the extrinsic world that form the context of an individual's life. For example, the home, community and society are included alongside the built environment, interpersonal relationships, attitudes, values, health and social policies, and the systems that support individuals and services (WHO, 2015).The Priority Assistive Products List of essential assistive devices includes wheelchairs, pill organizers, hearing aids, and other essential items for many older people and people with disabilities to be able to live a healthy and dignified life and mitigate declines in intrinsic capacity (WHO, 2016b).

The accumulation of exposures and environmental influences throughout life can influence the development of different risk factors that lead to chronic diseases, injuries, or other age‐related issues that contribute to declines in intrinsic capacities. Without a supportive environment, whether social or built, this will result in diminished functional ability. The gradual decline in intrinsic capacities as some people age can require increased health and social care services from informal (i.e., family or friends) and formal caregivers (i.e., healthcare professionals). Increased care needs lead to increased burden on families, stress for older adults, and costs to society. For this reason, efforts to deliver cost efficient, effective interventions that optimize functional ability at any level of intrinsic capacity, is critical for older adults. Health and social care interventions (including assistive health technologies), and related systems, services and policies may include technological tools and devices and provision of health and social supports in the home.

While it is important to offer home‐based supports that promote functional ability, we need to be mindful that existing health inequities may be worsened (Sadana et al., 2016). For example, if health and social services are provided privately and not covered by the health system or health insurance, all individuals will not have the same opportunities to achieve optimal health. Age‐based bias is seen in research on conditions that affect older adults such as stroke and osteoarthritis, with the median age of participants in research over 10 years younger than the typical patient (Gaynor et al., 2014; Liberopoulos et al., 2009).

### 2.2 Why it is important to develop the EGM

Over 85% of research investment is wasted (Chalmers & Glasziou, 2009), some of which could be avoided by prioritizing research directions and including rigorous evaluation of existing evidence using systematic reviews prior to funding or carrying out new research (Chalmers et al., 2014). An EGM is a decision‐making and research‐prioritization tool that highlights gaps in research to inform strategic health and social policy, program, and research priorities (Snilstveit et al., 2013). EGMs can be used to avoid needless duplication, and can also be used to identify where sufficient, high quality evidence from systematic reviews and randomized trials are available as a basis for decisions or where sufficient studies are available for knowledge synthesis (Snilstveit et al., 2016).

This EGM is important to inform policy and research prioritization. It is aligned with the WHO Strategy and Action Plan on Ageing and Health 2016–2020. At the 69th World Health Assembly in May 2016, the WHO launched and received endorsement from all 193 member states for the WHO Strategy and Action Plan on Ageing and Health 2016–2020. This plan outlined five strategic objectives: (1) commitment to action on healthy ageing in every country, (2) developing age‐friendly environments; (3) aligning health systems to the needs of older populations; (4) developing sustainable and equitable systems for providing long‐term care; (5) improving measurement, monitoring, and research on healthy active ageing. The WHO aims to meet these by implementing evidence‐based actions to maximize functional ability of every individual (WHO, 2016). In this way the process of “optimizing opportunities for health, participation and security will enhance the quality of life as people age” (WHO, 2015). This EGM is relevant to the first objective—a commitment to action on healthy ageing in every country. Furthermore, our objectives align with the United Nations Sustainable Development Goals and the objectives of the UN High Level meeting on preventing and controlling noncommunicable diseases (United Nations, 2019; WHO, 2018).

We took a health systems perspective to extend the focus from health care to include social care and technological interventions. The evidence is presented in terms of functional ability. We also considered determinants of health inequity. This EGM considers the multifaceted and complex nature of functional ability and the various mechanisms (e.g., services, products and individuals) involved in supporting functional ability among ageing adults.

Currently, no EGMs exist that identify and assess the available evidence on health, social care and technological interventions to support functional ability among older adults living at home.

There are three primary audiences for this EGM. First, we expect researchers (e.g., universities, government, etc.) will use the results to inform further investigations on these topics, including new empirical research and evidence synthesis products. The second main anticipated audience is decision‐makers for whom intrinsic capacity, functional ability and process outcomes are already or potentially of interest. This includes relevant ministries and programs within governments and/or donor agencies, as well as nongovernmental organizations and other advocacy and implementing organization staff. From a policy perspective, it is especially useful to know what kinds of interventions might most effectively affect intrinsic capacity, functional ability, and process outcomes. The third main anticipated audience is health and social care providers who can use the map to identify quality assessed synthesized evidence of health, social care, and technological interventions for their practice.

## 3 OBJECTIVES

The objectives of this Campbell EGM are to:

- Identify and assess the available evidence on health, social care and technological interventions to improve functional ability among older adults living at home
- Identify available systematic reviews and randomized trials
- Identify areas where systematic reviews are needed
- Identify gaps in evidence where further primary research is needed
- Assess equity considerations in available systematic reviews and randomized trials
- Assess gaps and evidence related to health equity

## 4 METHODS

### 4.1 EGM: Definition and purpose

This EGM is based on a published protocol (Welch et al., 2019). We adapted evidence gap map methods from various key papers (Bragge et al., 2011; Lum et al., 2011; Snilstveit et al., 2013, 2016) and followed a five stage process:

- Define a framework
- Identify the available evidence
- Appraise the quality of the evidence
- Extract, code and summarize the data that relate to the objectives
- Visualize and present the findings in a user‐friendly manner

This five stage process aligns with current Campbell Collaboration guidance (White et al., 2020). We used the Campbell Collaboration mapping tool developed by Digital Solution Foundary and EPPI‐Centre (Digital Solution Foundary and EPPI‐Centre, 2020) to display identified studies using the framework described below.

### 4.2 Framework development and scope

The framework was developed following a meeting with methodologists, practitioners, decision makers and consumers at the Cochrane Colloquium during the 2017 Global Evidence Summit. The colloquium participants suggested using the International Classification of Functioning, Disability and Health (ICF) framework (Sadana & Posarac, 2018; WHO 2001) to define the interventions and outcomes for this EGM. We further defined the scope of the framework in consultation with our research team which includes input from the public (A. L.), practitioners (L. S., P. T., K. P., J. B., E. T., P. W. and M. C.), information scientist (M. R.), policy‐makers (R. S. and H. S.) and researchers (V. W., S. M., J. T. C., T. H., M. C., E. K., B. S., A. S. and W. Z.). The ICF is a comprehensive framework used by the WHO to measure health and disability at both individual and population levels, as well as to operationalize the measurement of intrinsic capacities, functional ability and enabling environments (Sadana & Posarac, 2018).

As such, the EGM framework informed the inclusion and exclusion criteria. We followed the standard EGM framework as a matrix where the rows show intervention domains and the columns show outcome domains. Key dimensions of the framework and their sub‐categories are detailed in the subsequent sections.

We further limited the scope to interventions that were provided in the home of older adults. Maintaining autonomy and independence, especially being able to make their own choices and decisions, are important for older adults in all settings (Hillcoat‐Nalletamby, 2014; Plath, 2009; Welford et al., 2012). We defined the concept of home broadly, as the place of dwelling in which older adults seek to maintain their autonomy. This definition included any nonacute care places of residence such as housing units (detached and semi‐detached houses or apartments), long‐term care facilities (including hospices, and nursing homes), independent living or assisted living facilities.

### 4.3 Stakeholder engagement

We created an Advisory Group comprised of methodologists, physicians (and other healthcare professionals), policy organizations, consumers and researchers with expertise in assistive health technology, healthy ageing, long‐term care, rehabilitation, disability, memory and cognitive impairment. We held an exploratory meeting to invite feedback on the development of our EGM framework at the Global Evidence Summit in Cape Town, September 2017. The participants included family practitioners, geriatricians, social workers and methodologists. We also held a seminar at the Bruyère Research Institute Grand Rounds (26 October 2017) with family practitioner researchers, where participants provided feedback on the intervention‐outcome framework. Our decision to focus on the selected intervention categories was also informed by engaging with our public representative (A. L.). Our central team (V. W., T. H., S. M., P. B. and C. M.) met at least once a month to discuss the direction and scope of the EGM. Preliminary findings were presented at the peer review meeting of WHO Consortium on Metrics and Evidence for Healthy Ageing, 10–11 October 2019. Feedback from the reviewers was included in the final document.

### 4.4 Conceptual framework

**Figure 1:**
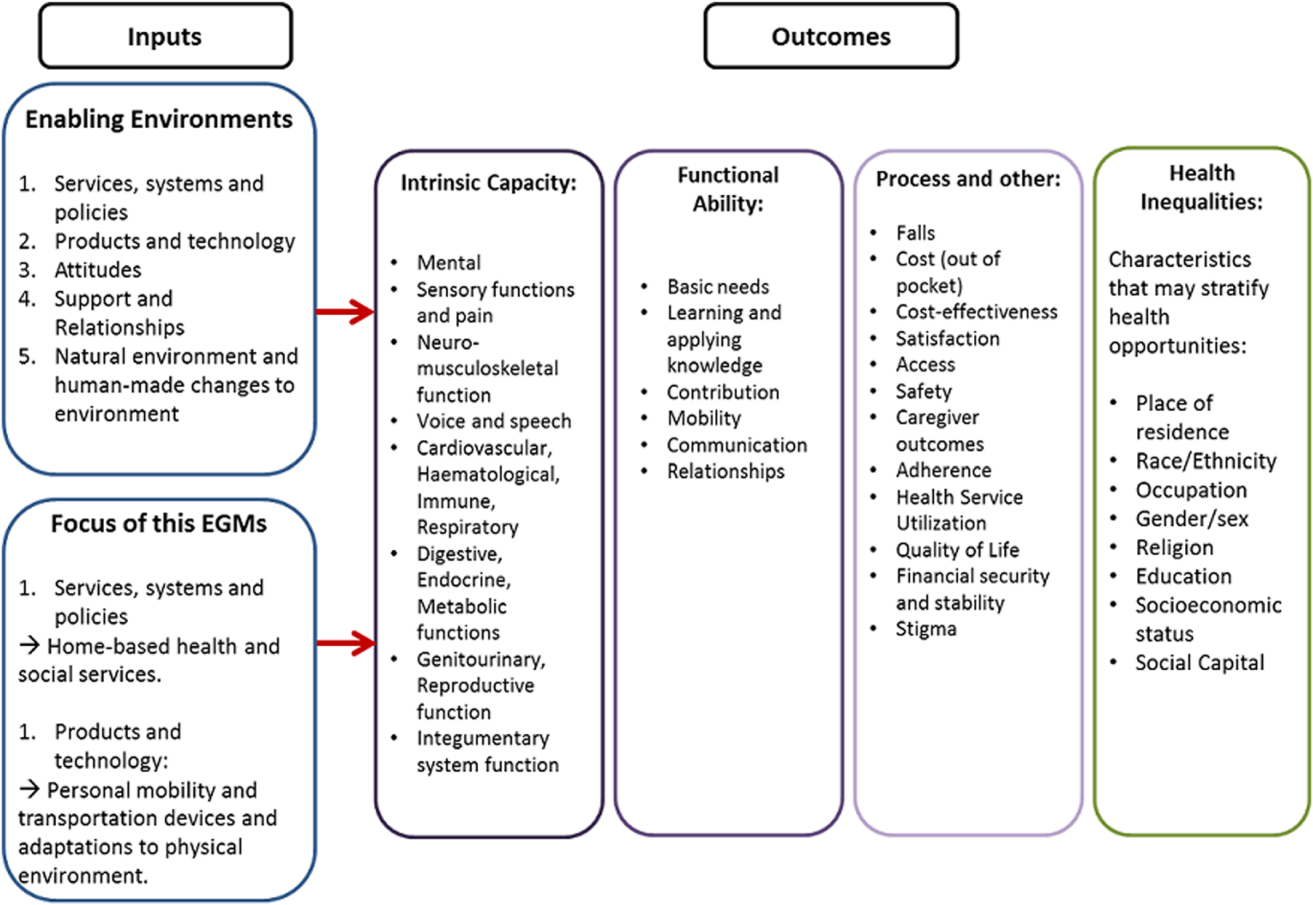
Conceptual framework adapted from the WHO International Classification of Functioning, Disability and Health (ICF)

Figure 1 below demonstrates the conceptual framework through which the inputs lead to the intended outcomes. A person's intrinsic capacity is dependent on their health characteristics (e.g., body functions, health related behaviors, disease or injuries), genetic inheritance, and personal characteristics (e.g., sex/gender or ethnicity). However, the extent to which an individual accomplishes activities that they value, functional ability, is also dependent on their interactions with the environment. Enabling environments may include services, systems and policies, and products and technology which, when implemented within a home context, can influence outcomes such as improved neuromusculoskeletal functioning, through the use of an external aid, assistance by another person or improvement in the built environment. Supportive environments can strongly influence functional ability. We also included health inequalities as an outcome of interest because we are aware that certain characteristics may stratify or impact health opportunities and outcome, such as socioeconomic status or place of residence.

### 4.5 Dimensions

#### 4.5.1 Types of study design

We included completed and on‐going systematic reviews and randomized controlled trials of effectiveness. We defined a systematic review according to the PRISMA definition (Moher et al., 2015), where the article explicitly states the methods used to identify studies (i.e., a search strategy), strategies for study selection (e.g., eligibility criteria and selection process) and explicitly detail methods of synthesis. We included studies published in grey literature such as reports, dissertations and conference abstracts.

We excluded systematic reviews of predictive factors, prognostic and diagnostic studies, and studies that primarily analyzed implementation, barriers and facilitators to effectiveness (Snilstveit et al., 2013). Literature reviews that did not describe methods used for search, data collection, and/or synthesis were excluded. We also excluded theoretical or modeling studies, editorials and commentaries. We did not include qualitative research.

#### 4.5.2 Types of intervention/problem

We contextualized interventions according to the International Classification of Functioning, Disability and Health (ICF) categorization of environmental factors. This was further divided into:

1. Health and social services, systems and policies: While we recognize that systems and policies can have an impact on the individual, we specifically focused on sections e5750 and e5800 from the ICF, which includes health and social support services provided at home such as homemaking, personal care, healthcare professional home visits, or long‐term care.
2. Products and technology related to mobility: The ICF provides a very comprehensive list of eligible interventions. We used sections e1201 and e155 that focused on assistive products and technology for personal indoor and outdoor mobility and transportation as well as design, construction and building products and technology of buildings for private use. This includes products such as wheelchairs, walking devices, transfer devices and ramps.

We decided to limit the scope of the ICF framework due to feasibility. Specifically, we excluded studies of pharmacological interventions, therapies, telemedicine or telecare, educational programs and any hospital‐based programs. We also excluded any studies that examine caregiver support services exclusively without evaluating outcomes related to older adults. A comprehensive list of interventions in each category may be found in Table 1.

**Table 1:** Interventions framework (based on the ICF)

Intervention category	Focus	Definition	Specific examples
Services, systems and policies	**e575** General social support services, systems and policies	Services, systems and policies aimed at providing support to those requiring assistance in areas such as shopping, housework, transport, child care, self‐care and care of others, in order to function more fully in society. Exclusions: social security services, systems and policies (e570); personal care providers and personal assistants (e340); health services, systems and policies (e580)	e5750 General social support services: Services and programs aimed at providing social support to people who, because of age, poverty, unemployment, health condition or disability, require public assistance in the areas of shopping, housework, transport, self‐care and care of others, in order to function more fully in society
	**e580** Health services, systems and policies	Services, systems and policies for preventing and treating health problems, providing medical rehabilitation and promoting a healthy lifestyle. Exclusions: general social support services, systems and policies	e5800 Health services: Services and programmes at a local, community, regional, state or national level, aimed at delivering interventions to individuals for their physical, psychological and social well‐being, such as health promotion and disease prevention services, primary care services, acute care, rehabilitation and long‐term care services; services that are publicly or privately funded, delivered on a short‐term, long‐term, periodic or one‐time basis, in a variety of service settings such as community, home‐based, school and work settings, general hospitals, specialty hospitals, clinics, and residential and nonresidential
Products and technology	**e120** Products and technology for personal indoor and outdoor mobility and transportation	Equipment, products and technologies used by people in activities of moving inside and outside buildings, including those adapted or specially designed, located in, on or near the person using them. Inclusions: general and assistive products and technology for personal indoor and outdoor mobility and transportation	e1201 Assistive products and technology for personal indoor and outdoor mobility and transportation. Adapted or specially designed equipment, products and technologies that assist people to move inside and outside buildings, such as walking devices (such as canes or crutches), special cars and vans, adaptations to vehicles, wheelchairs, scooters and transfer devices
	**e155** Design, construction and building products and technology of buildings for private use	Products and technology that constitute an individual's indoor and outdoor human‐made environment that is planned, designed and constructed for private use (e.g., home, dwelling), including those adapted or specially designed. Inclusions: entry and exits, facilities and routing	e1550 Design, construction and building products and technology for entering and exiting of buildings for private use. Products and technology of entry and exit from the human‐made environment that is planned, designed and constructed for private use, such as entries and exits to private homes, portable and stationary ramps, power‐assisted doors, lever door handles and level door thresholds

#### 4.5.3 Types of population (as applicable)

This EGM focused on adults over the age of 60 years, using the United Nations cut off for older adults (United Nations, 2015). Studies and reviews were included if at least 50% of the sample population was greater than 60 years old.

#### 4.5.4 Types of outcome measures (as applicable)

We mapped the evidence on outcomes that fell into one of the following ICF (WHO, 2001) adapted categories: intrinsic capacities (body function and structures) and functional abilities (activities). We also included process and other outcomes that may impact a particular outcome. We considered health inequities by examining environmental and personal attributes that may stratify health opportunities and outcomes, using the PROGRESS framework (O'Neill et al., 2014). PROGRESS is an acronym which stands for: place of residence, race/ethnicity, occupation, gender, religion, education, socioeconomic status and social capital. Our outcomes framework is provided in Table 2.

**Table 2:** Outcomes framework

Outcome category	Measure/construct
Intrinsic capacity	Mental Sensory functions and pain Neuromusculoskeletal function Voice and speech Cardiovascular, haematological, immune, respiratory Digestive, endocrine, metabolic functions Genitourinary, reproductive function Integumentary system function
Functional ability	Basic needs Learning and applying knowledge Contribution Mobility Communication Relationships
Process and other	Falls Cost (out of pocket) Cost‐effectiveness Satisfaction Access Safety Caregiver outcomes Adherence Health service utilization Quality of life Financial security and stability Stigma
Health inequalities	Place of residence Race/ethnicity Occupation Gender/sex Religion Education Socioeconomic status Social capital

The intrinsic capacity outcome category consisted of mental (e.g., depression, sleep, vitality); sensory functions and pain (e.g., vision, hearing); neuromusculoskeletal function (e.g., gait, balance); voice and speech (e.g., articulation); cardiovascular, haematological, immune, respiratory system function (e.g., blood pressure, respiration); digestive, endocrine, metabolic functions (e.g., thyroid, glucose); genitourinary and reproductive function (e.g., bladder control); and integumentary system function (e.g., skin, nails).

The functional ability outcome category consisted of the following constructs: basic needs (e.g., self‐care, acquisition of goods and services); learning and applying knowledge; contribution (e.g., community life, employment); mobility (e.g., walking); relationships (e.g., interpersonal interactions); and communication (e.g., language).

Process and other outcomes included cost (out of pocket), cost‐effectiveness, falls, satisfaction of older adult, safety, caregiver outcomes, adherence, health service utilization, quality of life, financial security, access and stigma. Access is a multifaceted concept and can be understood as the opportunity or ease with which consumers or communities are able to use appropriate services in proportion to their needs (Daniels, 1982; Whitehead, 1992). As such, the concept of access included: acceptability, approachability, availability and accommodation, affordability and appropriateness (Levesque et al., 2013).

#### 4.5.5 Other eligibility criteria

##### Types of settings

We included interventions which were provided in the home setting for older adults. We defined home as an individual's place of residence that can include housing units (houses/apartments), long‐term care (including nursing homes, and hospices), independent living (e.g., retirement residences), and assisted living facilities. We did not include any acute or sub‐acute care and convalescent care settings (e.g., geriatric rehabilitation in subacute care). Studies of mixed settings were included if the intervention took place in the home setting at least 50% of the time. We coded the settings so that the evidence can be filtered according to setting.

### 4.6 Search methods and sources

We developed and piloted a search strategy (with a selection of studies that met our inclusion criteria) with the guidance of an information scientist (M. R.). This search comprised medical and health databases (MEDLINE (via OvidSp), EMBASE (via OvidSp), Cochrane Database of Systematic Reviews, CENTRAL, CINAHL (Via EBSCOhost), APA PsycINFO (via OvidSp), AgeLine (via EBSCOhost) and databases relevant to social care and social policy (Campbell Library, ASSIA (via ProQuest), Social Science Citation Index (via Web of Science) and Social Policy & Practice (via OvidSp). The database searches were run between 26 July 2018 and 1 August 2018. No limits for language or date were used. See Table 3 for full search strategy as used in MEDLINE, and adapted for the other databases (see Appendix 1, 2, 3, 4, 5, 6, 7, 8).

**Table 3:** Search strategy for MEDLINE

Category	Terms
Population	1 exp Aged/pc, px, rh [Prevention & Control, Psychology, Rehabilitation] (8053) 2 "Aged, 80 and over"/(806254) 3 Frail Elderly/(9588) 4 elderly.ti,ab. (219354) 5 older people.ti,ab. (23442) 6 older adult*.ti,ab. (61366) 7 older men.ti,ab. (7857) 8 older women.ti,ab. (12791) 9 old* age*.ti,ab. (65408) 10 pensioners.ti,ab. (793) 11 retirement.ti,ab. (11779) 12 "end of life".ti,ab. (18653) 13 (Resident* and (old* or home* or retirement or nursing)).ti,ab. (38765) 14 geriatric*.ti,ab. (41516) 15 (veteran* and (old* or home* or retire*)).ti,ab. (5047) 16 or/1‐15 (1121318)
Intervention	17 exp Self‐Help Devices/(10537) 18 exp Orthopedic Equipment/(92047) 19 assistive devices.ti,ab. (1494) 20 assistive equipment.ti,ab. (39) 21 mobility equipment.ti,ab. (20) 22 mobility device*.ti,ab. (311) 23 mobility aid*.ti,ab. (276) 24 motility.ti,ab. (85101) 25 (walking adj2 (device* or aid* or equipment)).ti,ab. (1248) 26 cane*.ti,ab. (5734) 27 crutches.ti,ab. (1155) 28 walking stick*.ti,ab. (202) 29 (Adapt* adj3 (cars or transport or vehicles)).ti,ab. (506) 30 (Adapt* adj3 (home* or house*)).ti,ab. (1545) 31 Wheelchair*.ti,ab. (6462) 32 exp Bathroom Equipment/(10) 33 scooter*.ti,ab. (368) 34 transfer device*.ti,ab. (231) 35 (communication adj (aid* or device*)).ti,ab. (858) 36 exp Optical devices/(88276) 37 Hearing aids/(7984) 38 eyeglasses.ti,ab. (683) 39 glasses.ti,ab. (10746) 40 spectacles.ti,ab. (2316) 41 hearing device*.ti,ab. (512) 42 hearing aid*.ti,ab. (8346) 43 vision aid*.ti,ab. (364) 44 ((Adapt* or adjust*) adj3 (door* or entry or exit)).ti,ab. (239) 45 Stair lift*.ti,ab. (2) 46 stair climbing.ti,ab. (1444) 47 stairs.ti,ab. (2902) 48 stair rails.ti,ab. (2) 49 shallow steps.ti,ab. (0) 50 (ramp or ramps).ti,ab. (7094) 51 Home Care Services/(31353) 52 home care service*.ti,ab. (1605) 53 home support service*.ti,ab. (59) 54 home visit*.ti,ab. (7662) 55 community services.ti,ab. (2375) 56 shopping.ti,ab. (3322) 57 house help.ti,ab. (1) 58 home help.ti,ab. (411) 59 (food adj (preparation or assistance or help or service or delivery)).ti,ab. (3932) 60 (meal* adj3 (provision or assistance or help or service* or preparation or delivery)).ti,ab. (1137) 61 homemaking.ti,ab. (109) 62 housekeeping.ti,ab. (8477) 63 ((household or ktichen or routine) adj (jobs or tasks or chores)).ti,ab. (888) 64 bathing.ti,ab. (9571) 65 grooming.ti,ab. (5015) 66 personal hygiene.ti,ab. (1847) 67 toileting.ti,ab. (857) 68 foot care.ti,ab. (1270) 69 (medication adj2 reminders).ti,ab. (147) 70 (kitchen or bathroom or bedroom).ti,ab. (5694) 71 or/17‐70 (400411)
Outcomes	72 exp "Activities of Daily Living"/(63476) 73 Human Activities/(2170) 74 Automobile Driving/(17307) 75 Leisure Activities/(7897) 76 "activities of daily living".ti,ab. (22139) 77 "quality of life".ti,ab. (229433) 78 "Quality of Life"/(164112) 79 independence.ti,ab. (36023)80 wellbeing.ti,ab. (11362) 81 social life.ti,ab. (3877) 82 social participation.ti,ab. (2177) 83 happiness.ti,ab. (5642) 84 happier.ti,ab. (734) 85 mental health.ti,ab. (116393) 86 functional ability.ti,ab. (4311)87 depression.ti,ab. (289365) 88 cognitive.ti,ab. (296200) 89 sensory function*.ti,ab. (3884) 90 pain.ti,ab. (543562) 91 distress.ti,ab. (97018) 92 vitality.ti,ab. (10533) 93 energy.ti,ab. (544017) 94 fatigue.ti,ab. (80717) 95 tiredness.ti,ab. (3430) 96 self care.ti,ab. (14789) 97 self efficacy.ti,ab. (21966) 98 mobility.ti,ab. (123516)99 community life.ti,ab. (457) 100 security.ti,ab. (38430) 101 relationships.ti,ab. (322577) 102 satisfaction.ti,ab. (113208) 103 adherence.ti,ab. (98155) 104 reablement.ti,ab. (49) 105 institutionali?ation.ti,ab. (4370)106 or/72‐105 (2682926)
Study design	107 systematic*.ti,ab. (374866) 108 (meta‐analysis or metaanalysis).ti,ab. (112568) 109 (review* and (literature or studies or trials)).ab. (693115) 110 review.ti. (393065) 111 (evidence adj2 synthesi*).ti,ab. (5932) 112 overview.ti,ab. (139107) 113 pubmed.ab. (82182) 114 medline.ab. (94705) 115 or/107‐114 (1336239) 116 randomized controlled trial.pt. (464926) 117 controlled clinical trial.pt. (92516) 118 randomized.ti,ab. (448898) 119 randomly.ab. (294026) 120 trial.ti,ab. (509010) 121 groups.ab. (1815046)122 usual care.ab. (13020) 123 or/116‐122 (2634734) 124 115 or 123 (3780045) 125 16 and 71 and 106 and 124 (3987)

We searched for relevant trials and systematic reviews in the grey literature via ProQuest Theses and Dissertation Global, and via Conference Proceedings Citation Index. We also searched for relevant unpublished studies via relevant international organizations (e.g., Help Age, WHO, and Institute for Research on Public Policy).

We searched for ongoing systematic reviews in PROSPERO and the Cochrane and Campbell libraries as well as on the open science framework (https://osf.io/). We searched for ongoing randomized trials in ClinicalTrials.gov and the WHO International Clinical Trials Registry Platform.

### 4.7 Analysis and presentation

#### 4.7.1 Filters for presentation

Our EGM is presented as a matrix of interventions (rows) and outcomes (columns) and reports the evidence base that met our inclusion criteria.

Users of the interactive EGM can additionally filter studies by the following filters:

- Publication status: completed studies and on‐going studies (i.e., study protocols).
- Age groups: 65 years and under, 65 years and above, over 75 years, and over 85 years.
- Health conditions/status: communicable disease (e.g., flu, HIV/AIDS), noncommunicable disease (e.g., dementias, diabetes, cancer, depression), injury (e.g., fractures, falls), discharged from hospital, end‐of‐life, physical frailty (e.g., at risk of functional decline), social frailty (e.g., social isolation), care dependent (e.g., when older adult is no longer able to undertake tasks necessary for daily life without the assistance of others (WHO, 2015), and dementia.
- WHO regions: South‐east Asia, Western Pacific, Europe, Africa, the Americas, Eastern Mediterranean.
- World Bank Classifications: high‐income economies, upper‐middle income economies, lower‐middle income economies, low‐income economies.
- Proportion of women included in study: 0%–25%, 25%–50%, 50%–75% and 75%–100%.

#### 4.7.2 Dependency

We linked all publications of the same study to count as one study (this included protocols if published and any secondary analyses). It is important to note that systematic reviews are likely to include the RCTs included in the map and there may be more than one systematic review which includes the same RCT(s). All relevant randomized trials were included regardless of whether they were included in a systematic review. We elaborate further in the discussion on how the interactive map should be interpreted.

### 4.8 Data collection and analysis

#### 4.8.1 Screening and study selection

Two reviewers independently screened the titles and abstracts of all retrieved articles. We screened titles and abstracts by intervention, study design, setting and population. We did not use outcomes as an inclusion criterion. Full‐texts of potentially eligible studies were screened independently in duplicate and any conflicts were resolved through discussion or by a third reviewer (V. W.). We did not contact authors of studies or reviews for missing information. Studies published in languages other than English or French, were translated using Google Translate and/or a native speaker, recruited through professional networks. This was done at the full‐text screening and coding stages.

#### 4.8.2 Data extraction and management

Once the eligible studies were identified, we tested and piloted the data extraction form on a sample of studies, generated a draft map, and met with our advisory board to make any modifications. We also invited feedback from our larger team. Two reviewers independently extracted data on published and ongoing systematic reviews and randomized trials related to the population, intervention, comparison, outcomes, setting and other categories we used as filters. We also coded studies to indicate whether the population was socially disadvantaged across PROGRESS (O'Neill et al., 2014) and identified whether any analyses were conducted across sex/gender or any other PROGRESS characteristics. Our complete list of coding categories for data extraction is found in Appendix 9. We coded systematic reviews using the research question or eligibility criteria. We did not go back to included primary studies within a review for more details. Differences in extraction were resolved by discussion.

#### 4.8.3 Tools for assessing risk of bias/study quality of included reviews

Since systematic reviews are often used for decision making, we appraised the methodological quality of systematic reviews using the AMSTAR‐2 (Assessing the Methodological Quality of Systematic Reviews) checklist (Shea et al., 2017) in duplicate for 10% of eligible studies. *κ* statistics were also used to check agreement for each item. If agreement was over 80%, we proceeded with single data extraction with verification by a second reviewer for the remainder of studies.

The quality of randomized trials is not usually assessed in EGMs since the purpose is to identify the randomized trials available, and not to make decisions based on single trials. As such, we did not assess quality of randomized trials (Snilstveit et al., 2017).

#### 4.8.4 Methods for mapping

We used the EPPI‐Reviewer 4 software (Thomas et al., 2010) for screening and coding, and the EPPI‐Mapper (Digital Solution Foundary and EPPI‐Centre, 2020) for generating the map. EPPI‐Reviewer and EPPI‐Mapper are developed by the EPPI‐Centre at the Social Science Research Unit of the UCL Institute of Education, University of London, UK (http://eppi.ioe.ac.uk/cms/Default.aspx?alias=eppi.ioe.ac.uk/cms/er4).

## 5 RESULTS

### 5.1 Description of studies

#### 5.1.1 Results of the search

**Figure 2:**
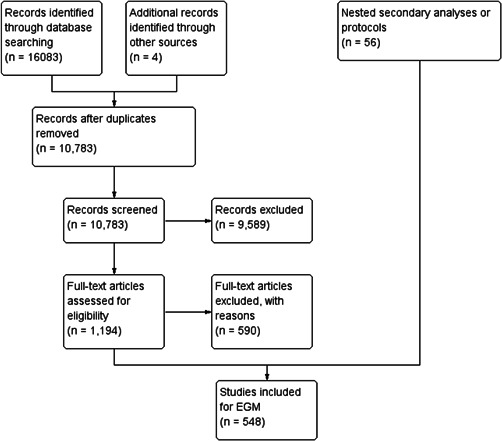
PRISMA flow chart

Our search retrieved 16,083 records from database searching with 4 additional records identified through other sources. After deduplication, 10,783 articles were screened in duplicate by title and abstract. From this, full texts of 1194 articles were screened in duplicate for eligibility. When full texts were not available, we used an interlibrary loan service. We included 548 studies in this map, of which 120 were systematic reviews (22%) and 428 were randomized controlled trials (78%). There were 502 completed studies including 117 completed systematic reviews (23%) and 385 completed randomized controlled trials (77%). Among the 46 on‐going studies, three were systematic reviews (7%) and 43 were randomized controlled trials (93%). See PRISMA flow chart in Figure 2.

#### 5.1.2 Excluded studies

The main reasons for exclusion at the full‐text screening stage were due to inappropriate intervention (*n* = 192), target population (*n* = 44), study design (*n* = 213), and setting (*n* = 141). See Supporting Information material for table of excluded studies and references.

### 5.2 Synthesis of included studies

Since many of the studies included in this EGM have been coded under multiple output indicators (e.g., more than one intervention category), a single study may appear in multiple cells. See Supporting Information interactive EGM map https://globalageing.cochrane.org/sites/globalageing.cochrane.org/files/public/uploads/ageing_egm_interactive_map_may5_20.html.

#### 5.2.1 Interventions

**Figure 3:**
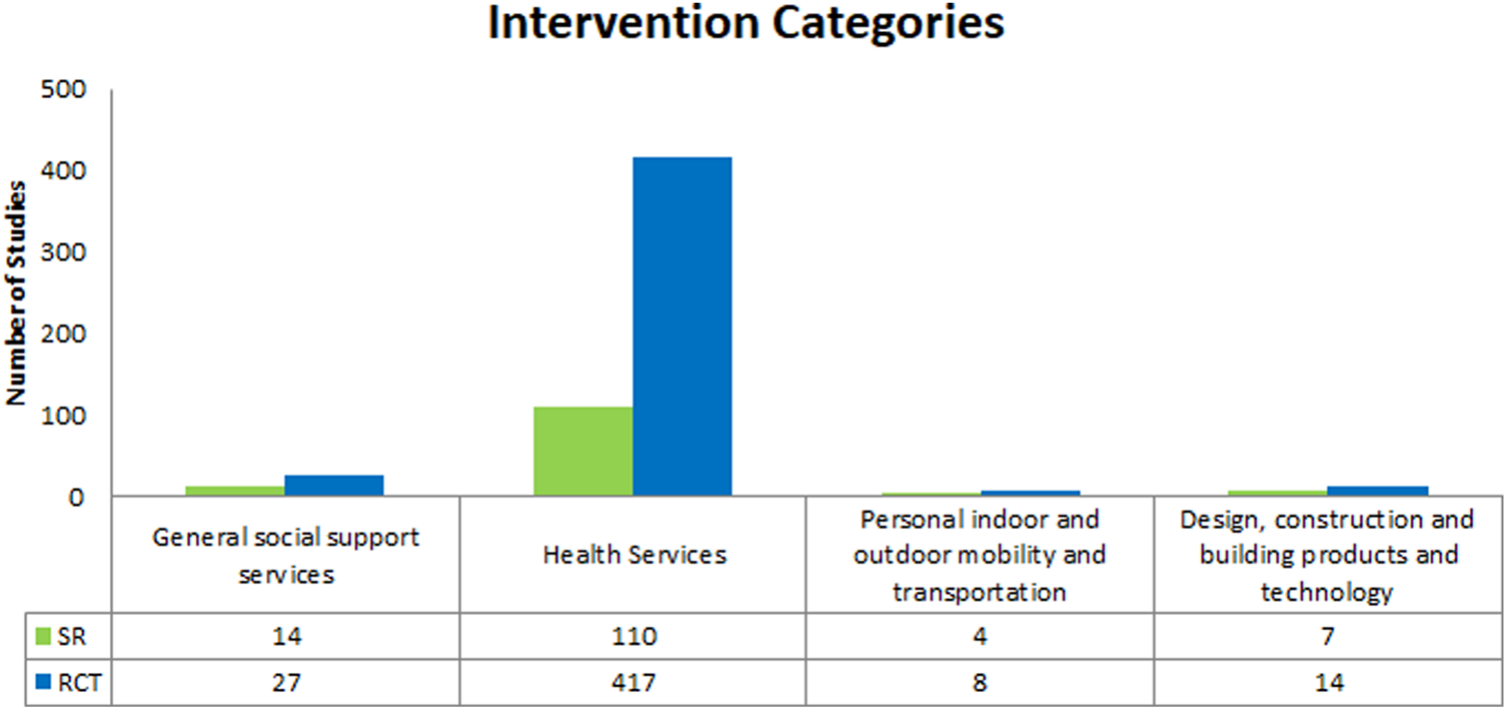
Intervention categories

As described earlier, we focused on four sections of the two broad domains of enabling environments within the ICF framework: health services, social support services, personal indoor and outdoor mobility and transportation, and design, construction and building products and technology. See Figure 3 for distribution of studies across our broad intervention categories.

Evidence, however, is not distributed evenly across the interventions and outcomes. Large clusters of randomized controlled trials and systematic reviews are present in some intervention areas (e.g., health services—rehabilitation services) while other intervention areas have very few studies (e.g., general social support services, systems and policies—transportation).

For visiting healthcare professional interventions these clusters of randomized controlled trials and systematic reviews include mental functions (*n* = 230, 186 RCTs and 104 SRs), neuro‐musculoskeletal (*n* = 138, 106 RCTs and 32 SRs), basic needs (*n* = 241, 190 RCTs and 51 SRs), mobility (*n* = 128, 115 RCTs and 13 SRs), quality of life (*n* = 189, 147 RCTs and 42 SRs), and health service utilization (*n* = 191, 147 RCTs and 44 SRs). For rehabilitation interventions these clusters include mental functions (*n* = 132, 105 RCTs and 27 SRs), neuro‐musculoskeletal (*n* = 134, 106 RCTs and 28 SRs), basic needs (*n* = 149, 111 RCTs and 38 SRs), mobility (*n* = 123, 111 RCTs and 12 SRs), quality of life (*n* = 115, 91 RCTs and 24 SRs), and health service utilization (*n* = 191, 48 RCTs and 143 SRs). For general health services for disease prevention interventions these clusters include mental functions (*n* = 118, 97 RCTs and 21 SRs), basic needs (*n* = 119, 97 RCTs and 22 SRs) and quality of life (*n* = 189, 77 RCTs and 112 SRs), and health service utilization (*n* = 129, 104 RCTs and 25 SRs).

There are few randomized controlled trials and systematic reviews that assess the following interventions across any outcomes; transportation (*n* = 2 RCTs), befriending or friendly visits (*n* = 3 RCTs), home making (*n* = 8, 7 RCTs and 1 SR), visiting lay service providers (*n* = 11 RCTs), caregiver support (*n* = 12, 8 RCTs and 4 SRs), personal mobility and transportation devices (*n* = 12, 8 RCTs and 4 SRs), adaptations to physical environments (*n* = 21, 14 RCTS and 7 SRs), personal care (*n* = 23, 14 RCTs and 9 SRs), long term care services (*n* = 14, 7 RCTs and 7 SRs), health promotion services (*n* = 27, 20 RCTs and 7 SRs).

It is important to recognize that these clusters are not suggestive of greater evidence for a (positive or negative) impact of an intervention on outcome indicators. Rather, they suggest that these relations have been investigated with greater frequency, irrespective of the actual impact documented.

#### 5.2.2 Outcomes

**Figure 4:**
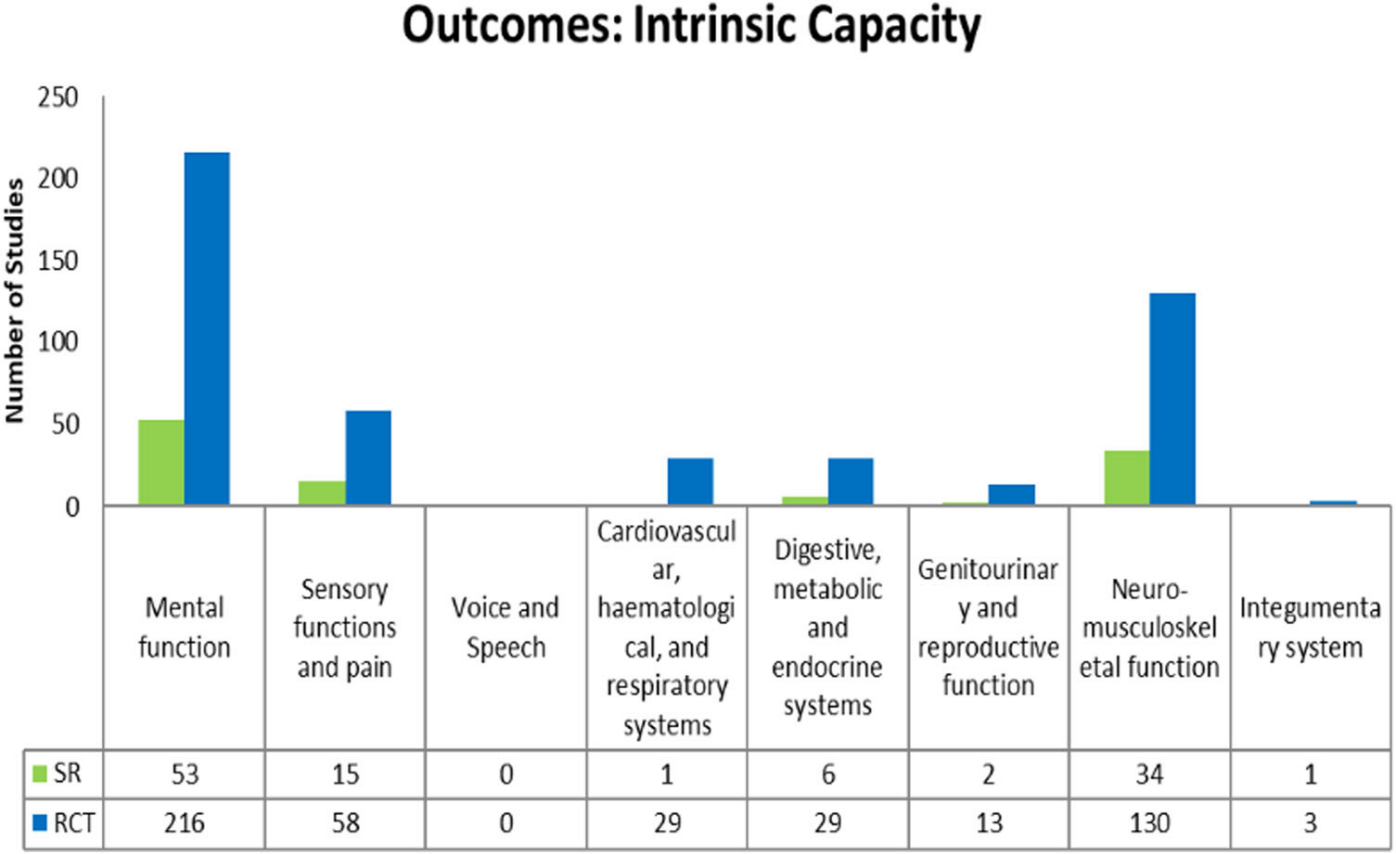
Intrinsic capacity outcomes

**Figure 5:**
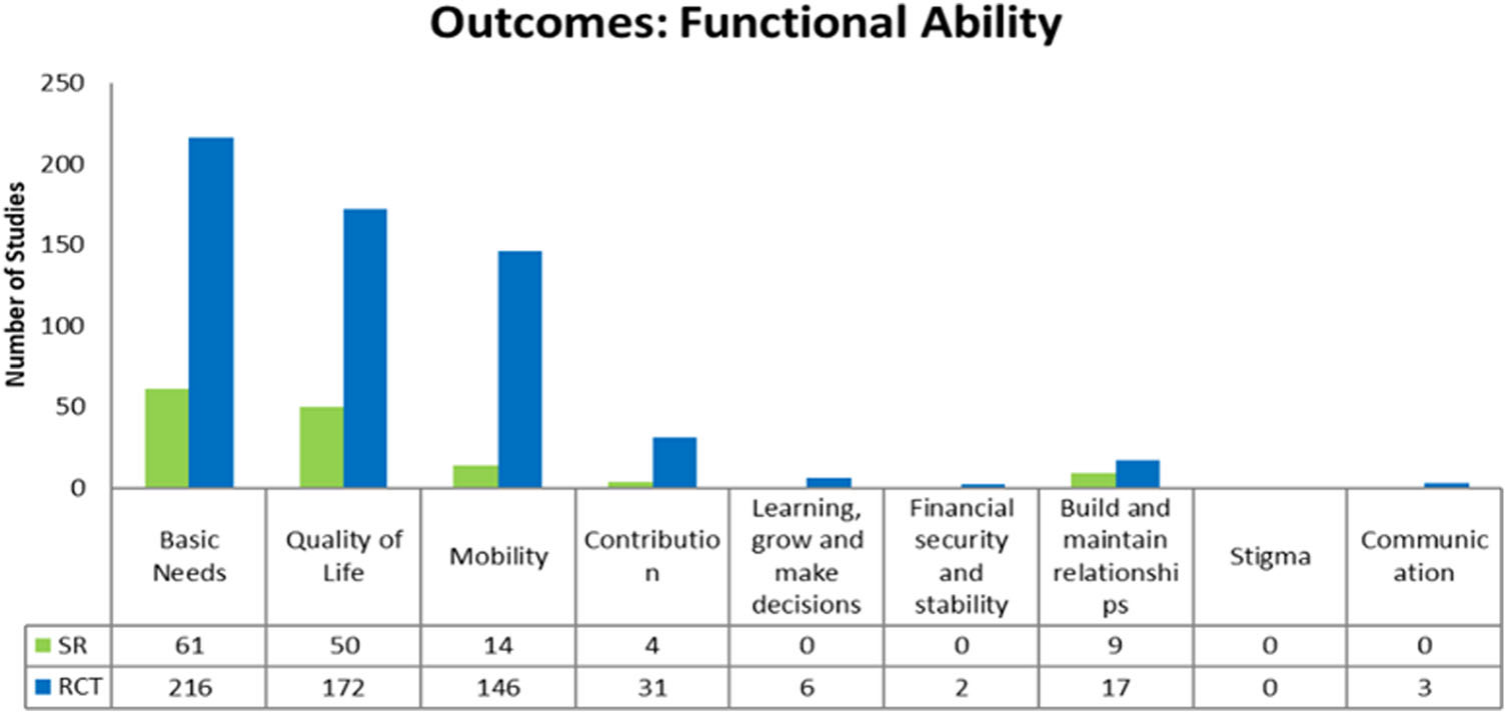
Functional ability outcomes

**Figure 6:**
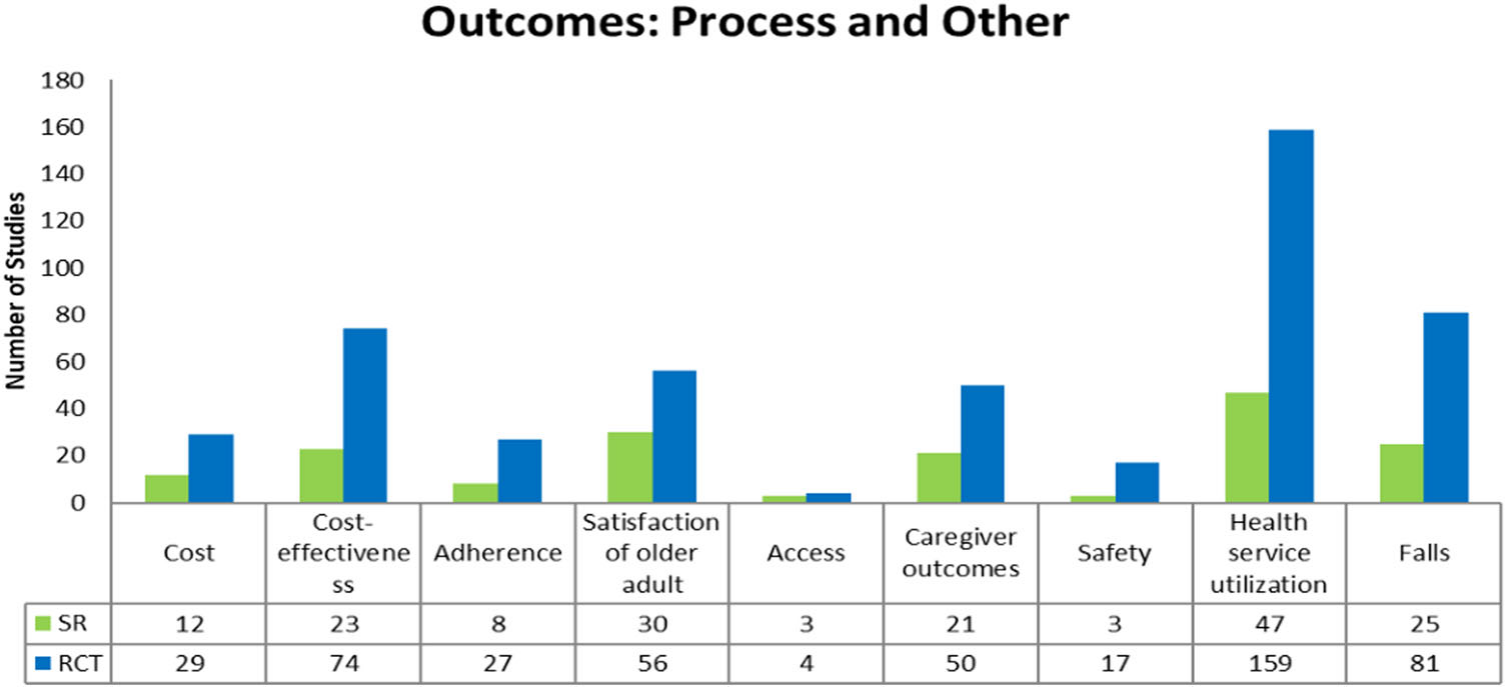
Process and other outcomes

Our EGM framework maps thirteen interventions to 26 outcomes; 8 intrinsic capacity, 9 functional ability and 9 process and other. The most frequently measured are intrinsic capacity outcomes related to mental functions (*n* = 269, 216 RCTs and 53 SRs), neuromusculoskeletal (*n* = 164, 130 RCTs and 34 SRs), sensory and pain (*n* = 73, 58 RCTS and 15 SRs) (see Figure 4); functional ability outcomes related to basic needs (*n* = 277, 216 RCTs and 61 SRs), quality of life (*n* = 222, 172 RCTs and 50 SRs) and mobility (*n* = 160, 146 RCTs and 14 SRs) (see Figure 5); and process and other outcomes related to health service utilization (*n* = 206, 159 RCTs and 47 SRs), falls (*n* = 106, 81 RCTs and 25 SRs), cost‐effectiveness (*n* = 97, 74 RCTs and 23 SRs), satisfaction of older adults (*n* = 86, 56 RCTs and 30 SRs), and caregiver outcomes (*n* = 71, 50 RCTs and 21 SRs) (see Figure 6).

For any intervention type there are no studies that assess voice and speech, and stigma. Furthermore, there are few studies that assess the following outcomes for any intervention type; financial security and stability (*n *= 2 RCTs), communication (*n* = 3 RCTs), integumentary system (*n* = 4, 3 RCTS and 1 SR), learning, grow and make decisions (*n* = 6 RCTs), access (*n* = 7, 4 RCTs and 2 SRs), genitourinary and reproductive functions (*n* = 15, 13 RCTs and 2 SRs), safety (*n* = 20, 17 RCTs and 3 SRs) (see Figures 4, 5, 6).

### 5.3 Risk of bias in included reviews

**Figure 7:**
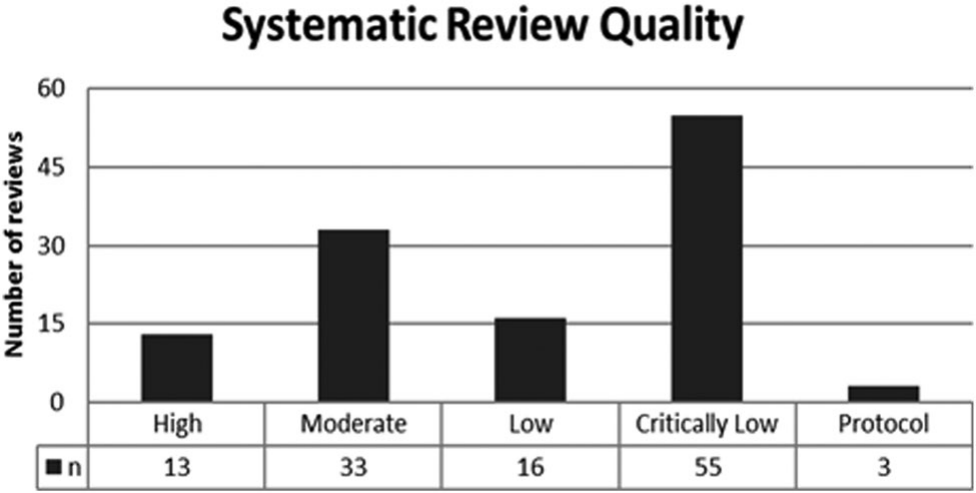
Methodological quality of systematic reviews

We assessed the methodological quality of 10% (12) systematic reviews in duplicate and once agreement was reached, we proceeded with single assessment of the rest. In total, 120 systematic reviews were assessed, of which, only 13 (11%) were high quality reviews, while the remaining rated moderate (28%), low (13%) and critically low (46%). We did not assess the methodological quality of the three on‐going systematic reviews (2%) (see Figure 7).

The main reasons for low quality are: (a) not reporting sources of funding for the studies included in the reviews, (b) not providing a list of excluded studies and justification for exclusion, (c) not accounting for risk of bias assessment in primary studies when interpreting or discussing the results, and (d) not using a satisfactory technique to assess risk of bias in individual studies included in the reviews.

### 5.4 Additional dimensions (if applicable)

#### 5.4.1 Health equity

##### Gender Inequalities

We assessed gender inequalities by:

- Checking the proportion of women included in systematic reviews and randomized controlled trials (completed and on‐going),
- We assessed whether the studies analyzed (O'Neill et al., 2014) effects of interventions by sex/gender or other PROGRESS factors.

**Figure 8:**
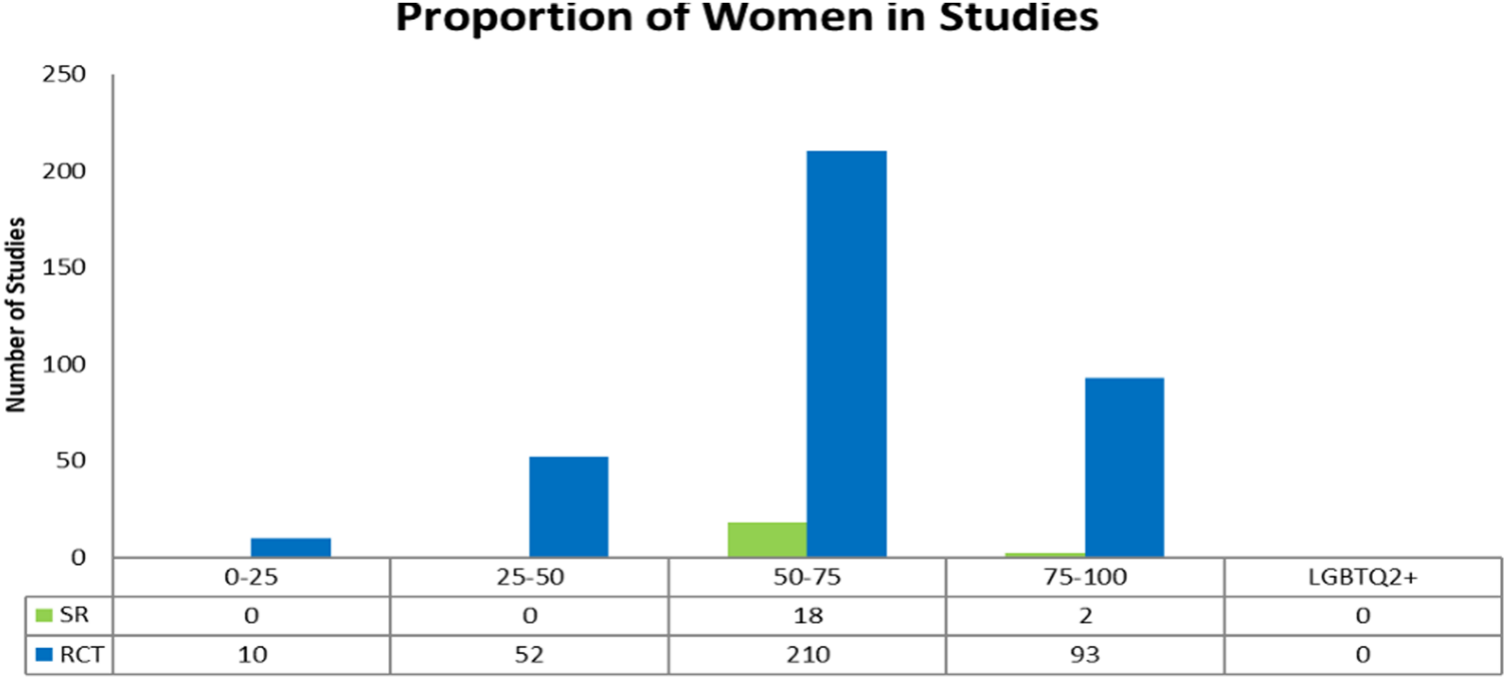
Proportion of women in studies

In 323 randomized controlled trials and 20 systematic reviews, women comprised >50% of the study participants (Figure 8). There were no studies that reported on including individuals from the LGBTQ2+ (lesbian, gay, bisexual, transgender, queer (or sometimes questioning), and two‐spirited) community.

Only 11 of the 548 included studies (2%) described the population as being socially disadvantaged across a PROGRESS characteristic: race/ethnicity, culture, language (*n* = 3 RCTs), socioeconomic status (*n* = 4, 3 RCTs and 1 SR), and social capital (*n* = 4, 2RCTs and 2 SRs).

Only one out of 548 included studies assessed effects of interventions across sex/gender and four studies assessed effects across another PROGRESS factor.

##### Region

**Figure 9:**
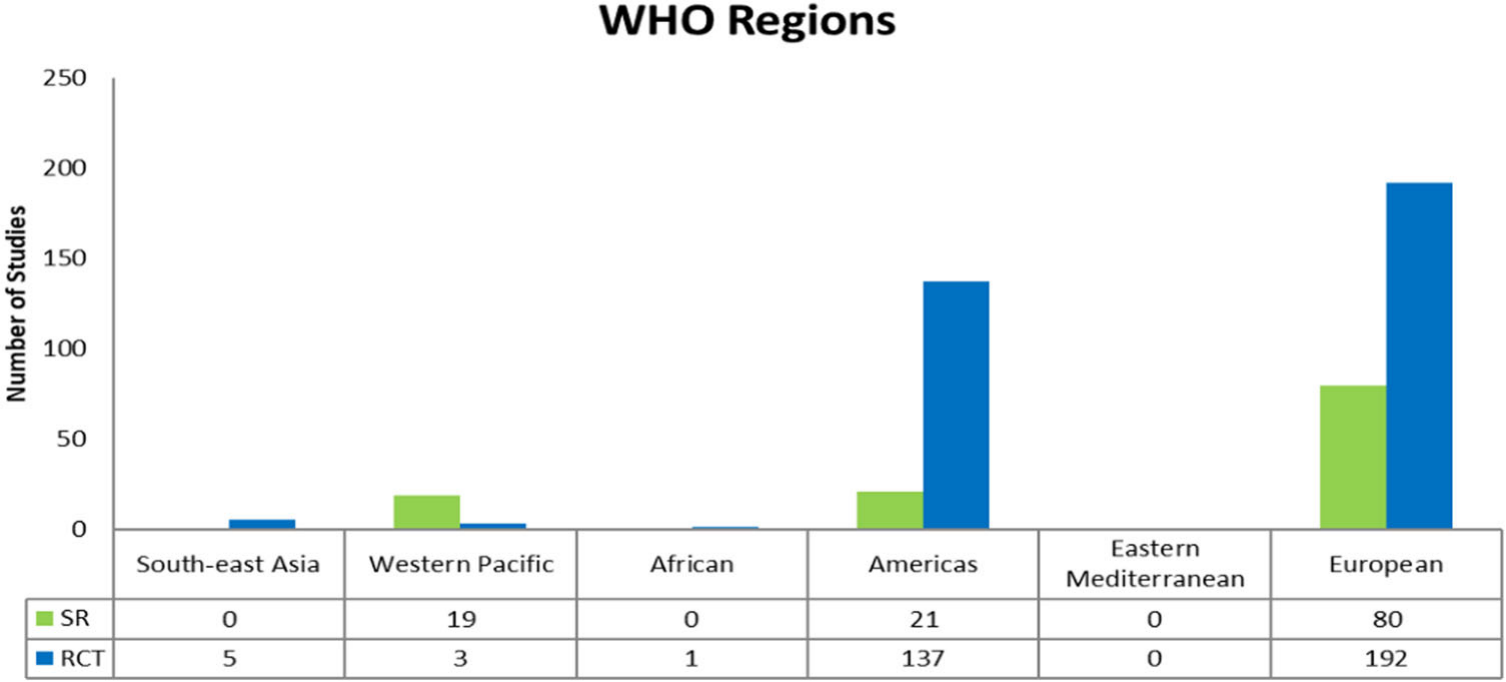
WHO regions

**Figure 10:**
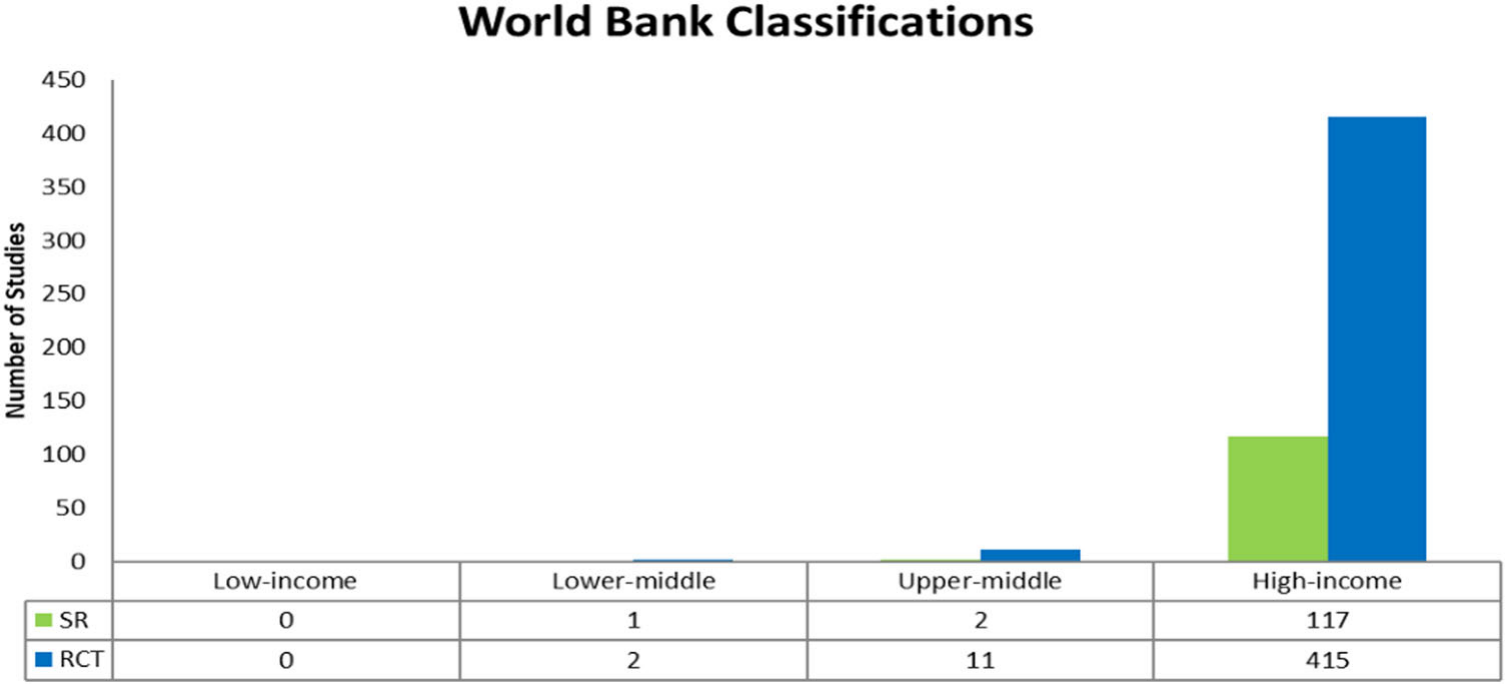
World Bank classifications

Across WHO regions, most of the studies evaluated describe and assess interventions in Europe (*n* = 272 (192 RCTs and 80 SRs); 49%), followed by the Americas (*n* = 158 (137 RCTs and 21 SRs); 29%) and Western Pacific (*n* = 112 (3 RCTs and 19 SRs); 20%) and with 5 or less studies in South‐East Asia, Africa and Eastern Mediterranean (see Figure 9). We also coded studies following the World Bank Classifications by economies. The majority of studies were from high‐income economies (*n* = 532, 415 RCTS and 117 SRs), with no studies from low‐income economies (see Figure 10). As stated earlier, please note that some studies were coded under more than one category. For example, a single study might have covered Europe and the Americas and will have been counted in both categories.

##### Setting

**Figure 11:**
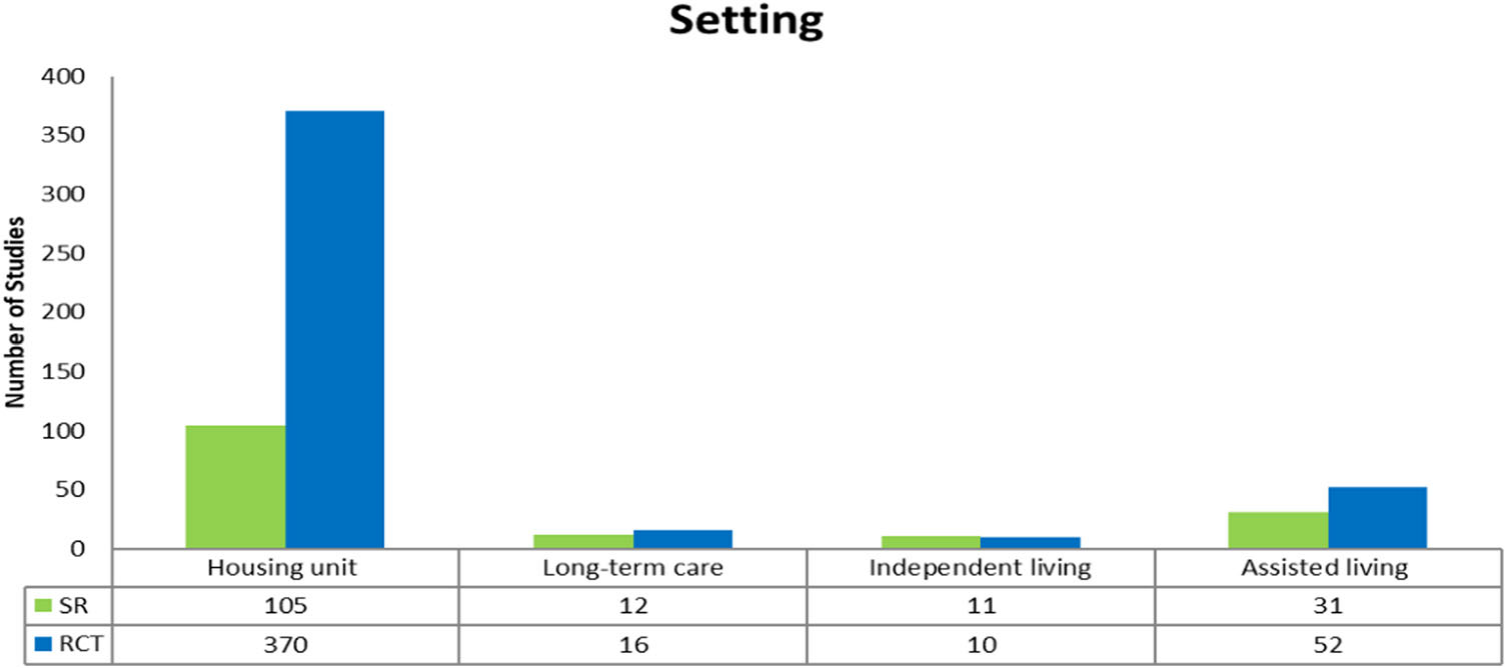
Setting

The majority of studies (*n* = 475, 370 RCTs and 105 SRs) took place in a housing unit (house or apartment) (see Figure 11). A single study may be coded in more than one setting.

##### Health condition/status

**Figure 12:**
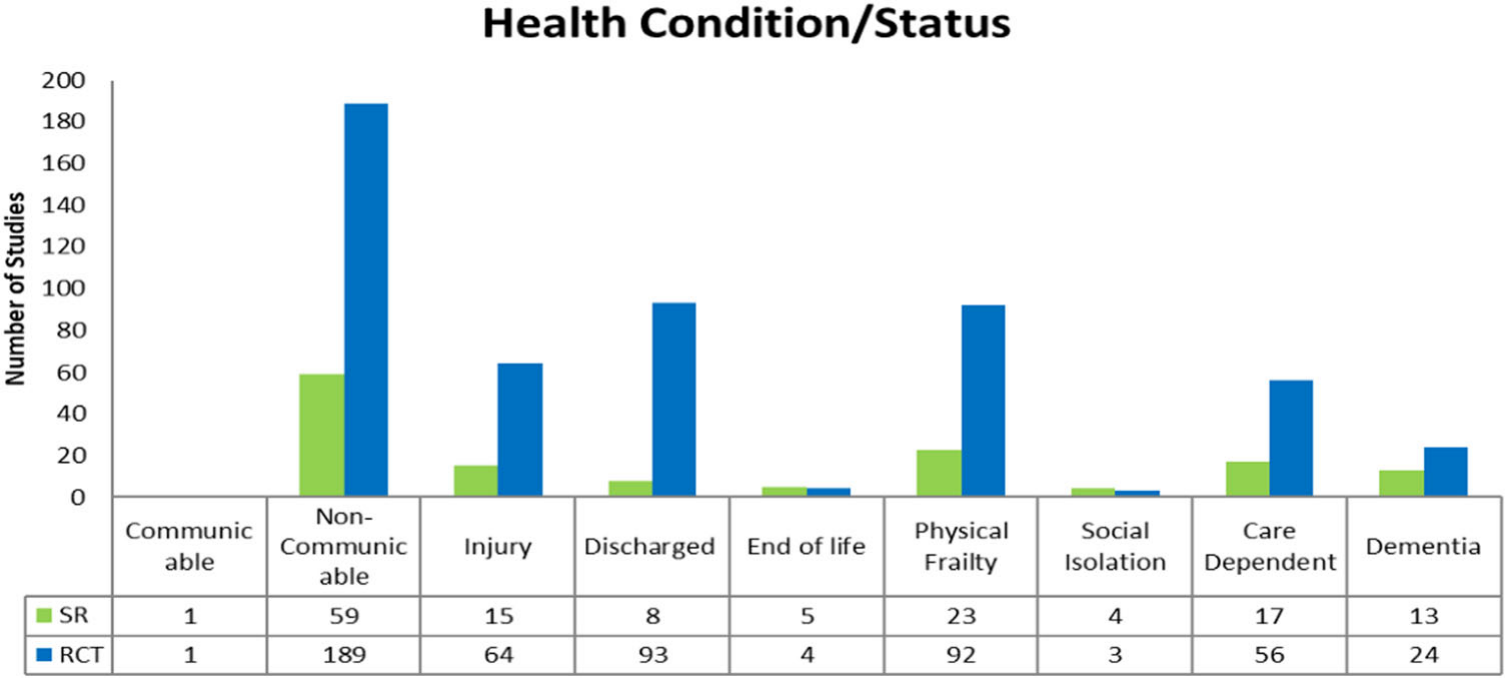
Health condition/status

We coded studies by health conditions of populations. The majority of studies included people with noncommunicable diseases (*n* = 248, 189 RCTs and 59 SRs). Very few studies (*n* = 7, 3 RCTs and 4 SRs) assessed loneliness and social isolation in older adults. We used the author's description of the population to identify studies in this domain. Most studies included populations that were coded under multiple categories (see Figure 12).

## 6 DISCUSSION

### 6.1 Summary of main results

The distribution of evidence in this EGM of health, social care and technological interventions to improve functional ability of older adults living at home or in other places of residence is not uniform. Home‐based health care has received more attention than social care or mobility support. Furthermore, the most common ICF outcome domains assessed were basic needs, quality of life and mobility, with relatively few studies reporting outcomes on societal contribution, learning, relationships, financial security. There were very few studies in LMICs (only 3%).

### 6.2 Areas of evidence clusters

The main cluster of evidence in this EGM is where interventions involve visiting healthcare professionals (*n* = 474); this is compared to a paucity of evidence exploring interventions provided by visiting lay service providers (*n* = 11). This may be because most studies took place in high‐income countries where there is greater use of home visits by healthcare professionals. However, many LMICs do not have access to home visiting healthcare professionals (Bashour et al., 2008; Ndiok & Ncama, 2019).

The evidence for rehabilitation services is clustered around neuro‐musculoskeletal function (*n* = 134) and mental health function (*n* = 131) outcomes. This may be explained in that over 20% of adults aged 60 and over suffer from a mental or neurological disorder (excluding headache disorders) and 6.6% of all disability adjusted life years (DALYs) among people over 60 years is attributed to mental and neurological disorders (WHO, 2017). Analysis of data from a WHO Study on global AGEing and adult health (SAGE) also points to the high prevalence of arthritis in low‐ and middle‐income settings, particularly among those in a lower socioeconomic position (WHO, 2001).

### 6.3 Areas of major gaps in the evidence

Our study reveals that systematic review evidence on the effects of home‐based health and social care and mobility support interventions is of limited methodological quality, with only 13 out of 120 reviews (11%) being rated as high methodological quality. Quality of systematic reviews in this area needs to be improved by adhering to methodological standards such as the Cochrane Handbook methods (Higgins et al., 2019) which include describing a clearly formulated question, describing eligibility criteria, search strategies, reasons for exclusion, publishing an a priori protocol and transparent reporting of methods (e.g., using the Preferred Reporting Items for Systematic Reviews and Meta‐analyses (Moher et al., 2015)). Importantly, quality is based on the methods of the review, not on the strength or quality of evidence within the review.

Furthermore, our EGM illustrates that studies are unevenly distributed across our full intervention‐outcome framework. Clusters emerge for some intervention—outcome combinations, in contrast with some noticeable evidence gaps. There is significant evidence (both randomized trials and systematic reviews) on health services, systems and policies (*n* = 525). Studies focusing on home‐based rehabilitation (*n* = 276) and general health services (*n* = 233) make up the largest proportion of studies in this map. There is a lack of data available on general social support services and policies (*n* = 41), personal indoor and outdoor mobility and transportation (*n* = 12), and design, construction and building products and technology (n = 21).

It is known that caregiver burden is a significant risk factor for depressive symptoms in carers of older people and may precipitate clinical depression (del‐Pino‐Casado et al., 2019) however, only 71 studies in the EGM explored caregiver outcomes. There were very few studies focused on loneliness and social isolation which is an important dimension for older adults (*n* = 7). Mobility limitations can contribute to social isolation and loneliness that may affect the mental and physical health of older adults (WHO, 2015).

Included studies mostly covered three WHO regions; Western Pacific, the Americas, and Europe. There were a small number of studies that covered South‐East Asia (*n* = 5) and Africa (*n* = 1). No studies covered the Eastern Mediterranean region. A significant proportion of studies are from high‐income economies (97%). The lack of evidence from low‐ and lower‐middle income countries points to the need for more high‐quality reviews and trials in these settings. This is particularly important since these regions, as previously mentioned, are experiencing a quicker growth in population ageing when compared to high‐income countries (UNDESA, 2017).

Diversity of characteristics and settings of older adults across age, sex/gender, ethnicity, medical conditions, settings, environments and culture may influence the impact of interventions. Over 90% of studies did not assess possible differences in effects across PROGRESS characteristics. The lack of health equity considerations within studies raise the need for future studies to consider health inequities, particularly since home‐based health, social and technology supports may not be accessible to all or require out of pocket costs, acceptability may differ across culture, country contexts and sex/gender, and programs may thus worsen or exacerbate existing health inequities.

### 6.4 Potential biases in the mapping process

We followed a systematic process with the help of an information scientist to develop our search strategy. As health and social care interventions and outcomes have different names in different contexts and languages, it is possible that we missed studies with our search strategy, even though the terms we used were developed in consultation with a search specialist and our advisory team, which included several experts in this field. In addition, we may have missed studies that were not indexed as home‐based. To mitigate this risk, we also reviewed the lists of included studies in eligible systematic reviews.

### 6.5 Limitations of the EGM

We focused on randomized trials for reasons of feasibility, thus our EGM may over‐represent interventions that lend themselves better to randomization. We mitigated the risk of over‐representing “randomizable” interventions by including systematic reviews of nonrandomized studies of interventions. However, users need to keep in mind that this EGM represents mostly randomized study evidence.

As with other EGMs, trials in our map may also be included in systematic reviews in this map and studies with multiple interventions or multiple outcomes will appear in multiple quadrants of the map. This is important to consider when interpreting the map.

Systematic reviews were assessed for eligibility and coded on the basis of their PICO question. That could mean that reviews with a broad focus could be excluded if home setting was not part of the PICO.

## 7 AUTHORS' CONCLUSIONS

This EGM is a starting point for identifying priority areas for systematic reviews and primary studies of home‐based health and social care and technological supports to support older adults at home.

### 7.1 Implications for research, practice and/or policy

There is a need for rigorous evaluation studies of home‐based social care and mobility support to promote functional ability for older adults. Despite substantial evidence on home‐based health services interventions, only 10% of included systematic reviews were high quality, thus limiting their usefulness for decision‐making.

There is a need to consider assessing outcomes of importance to older adults such as financial security, societal contribution and participation, stigma, loneliness and social isolation, caregiver outcomes, cost, and safety which were assessed in <20% of included evidence sources.

There is a need to consider analyses to assess effects of interventions across equity factors. Without evaluation of gender and health inequities, we risk promoting interventions that could exacerbate or worsen existing gender and health inequities.

At the time of publication of this map, there is a huge need to understand how to best promote functional capacity of older adults who are unable to leave their homes due to social distancing restrictions levied in the interests of slowing the spread of SARS‐Cov‐2 in the population. This map provides an initial resource to identify relevant home‐based services which may be of interest to policy‐makers and healthcare professionals such as home‐based rehabilitation and social support. Some interventions may require further adaptation for online delivery during the COVID‐19 pandemic.

## CONTRIBUTIONS OF AUTHORS

- Content: Tracey E. Howe, Vivian Welch, Heidi Sveistrup, Sue Marcus, provide content expertise in rehabilitation, assistive devices and memory and cognitive impairment. Christine M. Mathew, Lisa Sheehy, and MC also have expertise in ageing and rehabilitation. Elizabeth Kristjansson has expertise in built environments and in healthy aging. Lisa Sheehy, Johan Borg, Wei Zhang, Joanna Thompson‐Coon, Anne Lyddiatt, Jason W. Nickerson, Peter Tanuseputro, Peter Walker, and Beverly Shea provided content expertise on classifying outcomes and interventions, and will provide critical comments on final manuscript.
- EGM methods: Vivian Welch, Ashrita Saran, Sue Marcus, Tracey E. Howe, Kevin Pottie, Elizabeth T. Ghogomu and Elizabeth Kristjansson are experts in systematic review methods.
- Information retrieval: Morwenna Rogers is an information specialist with experience in designing searches for systematic reviews.

## DECLARATIONS OF INTEREST

VW is Editor in Chief of the Campbell Collaboration.

Johan Borg is employed as research manager at a commercial assistive technology company that may have an interest in the results or conclusions of this review.

JTC and MR were supported by the National Institute for Health Research Applied Research Collaboration South West Peninsula (PenARC) and this article therefore presents independent research supported by the National Institute for Health Research Applied Research Collaboration South West Peninsula (PenARC). The views expressed in this publication are those of the author(s) and not necessarily those of the National Health Service, the NIHR or the Department of Health and Social Care. The funders had no role in study design, data collection and analysis, decision to publish, or preparation of the manuscript.

The rest of the authors have no conflicts of interest with respect to the content of the EGM.

### PRELIMINARY TIMEFRAME

Approximate date for submission of the EGM: October 2019.

Please note this should be no longer than 1 year after protocol approval.

### PLANS FOR UPDATING THE EGM

Vivian Welch, Tracey Howe and Sue Marcus, as directors of Cochrane Global Ageing, have an interest in continuing to update this EGM. Frequency of updating will depend on availability of resources to do so.

## REFERENCES TO STUDIES

**INCLUDED STUDIES**

**Table:** 

Study	Publication status	Study design	Population‐age group	Population‐sex/gender	Health status/condition	WHO region	World Bank classification by income	Intervention: general social support services, systems and policies	Intervention: for personal indoor and outdoor mobility and transportation	Intervention: health services, systems and policies
Acton (2016)										
	Complete	RCT	Includes <65	50%‐75% female included	Noncommunicable disease	European	High‐income economies	Personal care		General health services for disease prevention
			Includes 65+							Rehabilitation services
										Visiting health professionals
Aimonino (2008)										
	Complete	RCT	Includes 75+	25%‐50% female included	Noncommunicable disease	European	High‐income economies			General health services for disease prevention
										Visiting health professionals
Alexander (2001)										
	Complete	RCT	Includes 65+	75%‐100% female included	Care dependent	The Americas	High‐income economies			Rehabilitation services
										Visiting lay service providers
Alexopoulos (2016)										
	Complete	RCT	Includes <65		Noncommunicable disease	The Americas	High‐income economies			General health services for disease prevention
			Includes 65+		Physical frailty					Visiting health professionals
Amjad (2018)										
	Complete	RCT	Includes 65+	50%‐75% female included	Dementia	The Americas	High‐income economies			General health services for disease prevention
					Noncommunicable disease					Visiting health professionals
Andersen (2000)										
	Complete	RCT	Includes <65	50%‐75% female included	Noncommunicable disease	European	High‐income economies			Visiting health professionals
			Includes 65+							
Anonymous (2004)										
	Complete	RCT	Includes 65+	75%‐100% female included	Noncommunicable disease	The Americas	High‐income economies			General health services for disease prevention
										Visiting health professionals
Araujo (2015)										
	On‐going	RCT	Includes 65+		Care dependent	European	High‐income economies	Transportation		
					Discharged from hospital			Personal care		
								Family and caregiver support		
Arean (2015)										
	Complete	RCT	Includes 65+		Care dependent	The Americas	High‐income economies			General health services for disease prevention
					Noncommunicable disease					Visiting health professionals
Arrieta (2018)										
	On‐going	RCT	Includes 65+	50%‐75% female included	Care dependent	European	High‐income economies			Rehabilitation services
					Noncommunicable disease					Visiting health professionals
Ashburn (2007)										
	Complete	RCT	Includes 65+	25%‐50% female included	Injury	European	High‐income economies			Rehabilitation services
					Noncommunicable disease					Visiting health professionals
Avlund (2002)										
	Complete	RCT	Includes <65		Discharged from hospital	European	High‐income economies			General health services for disease prevention
			Includes 65+							Visiting health professionals
Baker (2007)										
	Complete	RCT	Includes 65+	50%‐75% female included	Physical frailty	Western Pacific	High‐income economies			Rehabilitation services
										Visiting health professionals
Banerjee (1996)										
	Complete	RCT	Includes 65+	25%‐50% female included	Noncommunicable disease	European	High‐income economies			General health services for disease prevention
					Physical frailty					Visiting health professionals
Barnes (2017)										
	On‐going	RCT	Includes <65	75%‐100% female included	Dementia	The Americas	High‐income economies			Rehabilitation services
			Includes 65+		Noncommunicable disease					Visiting health professionals
Barreto (2018)										
	Complete	RCT	Includes 65+	50%‐75% female included	Noncommunicable disease	European	High‐income economies			Health promotion services
										Rehabilitation services
										Visiting health professionals
Batchelor‐Murphy (2017)										
	Complete	RCT	Includes 65+	75%‐100% female included	Care dependent	The Americas	High‐income economies	Personal care		Visiting health professionals
					Dementia					
					Noncommunicable disease					
Beck (2013)										
	Complete	RCT	Includes 65+	50%‐75% female included	Discharged from hospital	European	High‐income economies	Family and caregiver support		General health services for disease prevention
										Visiting health professionals
								
Beck (2016)										
	Complete	RCT	Includes 75+	50%‐75% female included	Care dependent	European	High‐income economies			General health services for disease prevention
					Noncommunicable disease					Rehabilitation services
										Visiting health professionals
Behm (2014)										
	Complete	RCT	Includes 75+	50%‐75% female included		European	High‐income economies			General health services for disease prevention
										Health promotion services
										Visiting health professionals
Behm (2016)										
	Complete	RCT	Includes 75+	50%‐75% female included	Physical frailty	European	High‐income economies			General health services for disease prevention
										Health promotion services
										Visiting health professionals
Beland (2006)										
	Complete	RCT	Includes 65+	50%‐75% female included	Care dependent	The Americas	High‐income economies	Homemaking		General health services for disease prevention
					Noncommunicable disease			Personal care		Visiting health professionals
Bennell (2018)										
	Complete	RCT	Includes <65	50%‐75% female included	Injury	Western Pacific	High‐income economies			Rehabilitation services
			Includes 65+							Visiting health professionals
Bernabei (1998)										
	Complete	RCT	Includes 65+	50%‐75% female included	Noncommunicable disease	European	High‐income economies	Personal care		General health services for disease prevention
					Physical frailty					Visiting health professionals
Bjerk (2017)										
	On‐going	RCT	Includes 65+		Care dependent	European	High‐income economies			Rehabilitation services
					Injury					Visiting health professionals
Blanchard (1999)										
	Complete	RCT	Includes 75+	75%‐100% female included	Noncommunicable disease	European	High‐income economies			General health services for disease prevention
										Visiting health professionals
Bleijenberg (2016)										
	Complete	RCT	Includes 65+	50%‐75% female included	Physical frailty	European	High‐income economies			General health services for disease prevention
										Visiting health professionals
Bonnefoy (2012)										
	Complete	RCT	Includes 75+	75%‐100% female included	Physical frailty	European	High‐income economies			Rehabilitation services
										Visiting health professionals
Boongird (2017)										
	Complete	RCT	Includes 65+	75%‐100% female included	Noncommunicable disease	South‐East Asia	Upper‐middle‐income economies			Rehabilitation services
										Visiting health professionals
Bouman (2008)										
	Complete	RCT	Includes 65+	50%‐75% female included	Physical frailty	European	High‐income economies			General health services for disease prevention
										Visiting health professionals
Boxall (2005)										
	Complete	RCT	Includes 65+	25%‐50% female included	Noncommunicable disease	Western Pacific	High‐income economies			General health services for disease prevention
										Rehabilitation services
										Visiting health professionals
Brannstrom (2014)										
	Complete	RCT	Includes 65+	25%‐50% female included	Noncommunicable disease	European	High‐income economies			General health services for disease prevention
										Long term care services
										Visiting health professionals
Brettschneider (2014)										
	Complete	RCT	Includes 75+	50%‐75% female included	Discharged from hospital	European	High‐income economies			General health services for disease prevention
										Visiting health professionals
Brovold (2012)										
	Complete	RCT	Includes 65+	50%‐75% female included	Discharged from hospital	European	High‐income economies			Rehabilitation services
										Visiting health professionals
Bruce (2015)										
	Complete	RCT	Includes 65+	50%‐75% female included	Noncommunicable disease	The Americas	High‐income economies			General health services for disease prevention
										Visiting health professionals
Bruce (2016)										
	Complete	RCT	Includes 65+	50%‐75% female included	Noncommunicable disease	The Americas	High‐income economies			General health services for disease prevention
										Visiting health professionals
Brumley (2007)										
	Complete	RCT	Includes 65+	25%‐50% female included	End‐of‐life	The Americas	High‐income economies			Long term care services
										Visiting health professionals
Burton (2013)										
	Complete	RCT	Includes 65+	75%‐100% female included	Physical frailty	Western Pacific	High‐income economies			Rehabilitation services
										Visiting health professionals
Buurman (2016)										
	Complete	RCT	Includes 65+	50%‐75% female included	Discharged from hospital	European	High‐income economies			General health services for disease prevention
										Visiting health professionals
Buys (2017)										
Complete	RCT	Includes 65+	75%‐100% female included	Discharged from hospital	The Americas	High‐income economies	Homemaking
Byles (2004)										
	Complete	RCT	Includes 75+	50%‐75% female included		Western Pacific	High‐income economies			General health services for disease prevention
										Visiting health professionals
Byrnes (2015)										
	Complete	RCT	Includes <65	25%‐50% female included	Discharged from hospital	Western Pacific	High‐income economies			General health services for disease prevention
			Includes 65+		Noncommunicable disease					Visiting health professionals
							
Callahan (2012)										
	On‐going	RCT	Includes <65		Noncommunicable disease	The Americas	High‐income economies	Personal care		General health services for disease prevention
			Includes 65+					Family and caregiver support		Visiting health professionals
Campbell (1997)										
	Complete	RCT	Includes 75+	75%‐100% female included	Injury	Western Pacific	High‐income economies			Rehabilitation services
					Noncommunicable disease					Visiting health professionals
Campbell (2005)										
	Complete	RCT	Includes 75+		Noncommunicable disease	Western Pacific	High‐income economies			Rehabilitation services
										Visiting health professionals
Canning (2015)										
	Complete	RCT	Includes <65	25%‐50% female included	Noncommunicable disease	Western Pacific	High‐income economies			Rehabilitation services
			Includes 65+							Visiting health professionals
Caplan (1999)										
	Complete	RCT	Includes 65+	75%‐100% female included	Communicable disease	Western Pacific	High‐income economies			General health services for disease prevention
					Noncommunicable disease					Visiting health professionals
Caplan (2004)										
	Complete	RCT	Includes 75+	50%‐75% female included	Discharged from hospital	Western Pacific	High‐income economies			General health services for disease prevention
										Visiting health professionals
Caplan (2006)										
	Complete	RCT	Includes 75+	50%‐75% female included	Discharged from hospital	Western Pacific	High‐income economies			Rehabilitation services
					Noncommunicable disease					Visiting health professionals
					Physical frailty					
Carroll (2007)										
	Complete	RCT	Includes 65+	50%‐75% female included	Noncommunicable disease	The Americas	High‐income economies			General health services for disease prevention
										Rehabilitation services
										Visiting health professionals
Chaiyawat (2012)										
	Complete	RCT	Includes <65	50%‐75% female included	Noncommunicable disease	South‐East Asia	Upper‐middle‐income economies			Rehabilitation services
			Includes 65+							Visiting health professionals
Chan et al. (2016)										
	Complete	RCT	Includes <65	50%‐75% female included	Discharged from hospital	Western Pacific	High‐income economies			General health services for disease prevention
			Includes 65+							Visiting health professionals
Chandler (1998)										
	Complete	RCT	Includes 65+	25%‐50% female included	Physical frailty	The Americas	High‐income economies			Rehabilitation services
										Visiting health professionals
Chang (2015)										
	Complete	RCT	Includes 65+	50%‐75% female included	Injury	The Americas	High‐income economies			Rehabilitation services
										Visiting health professionals
Chee (2013)										
	Complete	RCT	Includes 75+	75%‐100% female included	Physical frailty	Western Pacific	High‐income economies			General health services for disease prevention
										Visiting health professionals
Chen (2015)										
	Complete	RCT	Includes 65+	25%‐50% female included	Care dependent	Western Pacific	High‐income economies			Rehabilitation services
										Visiting health professionals
Chen (2015)										
	Complete	RCT	Includes 65+	25%‐50% female included	Physical frailty	Western Pacific	High‐income economies			Rehabilitation services
										Visiting health professionals
Chen (2016)										
	Complete	RCT	Includes 65+	50%‐75% female included	Noncommunicable disease	Western Pacific	High‐income economies			Rehabilitation services
										Visiting health professionals
Cho (1998)										
	Complete	RCT	Includes 75+	50%‐75% female included	Injury	The Americas	High‐income economies			General health services for disease prevention
					Noncommunicable disease					Visiting health professionals
Chow (2014)										
	Complete	RCT	Includes 65+	50%‐75% female included	Discharged from hospital	Western Pacific	High‐income economies			General health services for disease prevention
					Noncommunicable disease					Visiting health professionals
Chu (2017)										
	Complete	RCT	Includes 65+	50%‐75% female included	Injury	Western Pacific	High‐income economies		Personal mobility and transportation devices	General health services for disease prevention
										Visiting health professionals
Cichocki (2015)										
	Complete	RCT	Includes 75+	75%‐100% female included	Care dependent	European	High‐income economies			Rehabilitation services
										Visiting health professionals
							
Ciechanowski (2004)										
	Complete	RCT	Includes 65+	75%‐100% female included	Noncommunicable disease	The Americas	High‐income economies			General health services for disease prevention
										Rehabilitation services
										Visiting health professionals
Claffey (1976)										
	Complete	RCT	Includes <65		Care dependent	The Americas	High‐income economies			General health services for disease prevention
			Includes 65+							Visiting health professionals
Clegg (2014)										
	Complete	RCT	Includes 65+	50%‐75% female included	Physical frailty	European	High‐income economies			Rehabilitation services
										Visiting health professionals
Clemson (2016)										
	Complete	RCT	Includes 65+	50%‐75% female included	Discharged from hospital	European	High‐income economies			Rehabilitation services
										Visiting health professionals
Comans (2010)										
	Complete	RCT	Includes 65+	50%‐75% female included	Injury	Western Pacific	High‐income economies			Rehabilitation services
					Physical frailty					Visiting health professionals
Conradsson (2010)										
	Complete	RCT	Includes 65+	50%‐75% female included	Care dependent	European	High‐income economies			Rehabilitation services
										Visiting health professionals
Cornu (2003)										
	On‐going	RCT	Includes 75+		Care dependent	European	High‐income economies			Rehabilitation services
									
Corr (1995)										
	Complete	RCT	Includes <65	50%‐75% female included	Discharged from hospital	European	High‐income economies			Rehabilitation services
			Includes 65+							Visiting health professionals
Counsell (2007)										
	Complete	RCT	Includes 65+	75%‐100% female included	Noncommunicable disease	The Americas	High‐income economies			General health services for disease prevention
										Visiting health professionals
Courtney (2009)										
	Complete	RCT	Includes 65+	50%‐75% female included	Discharged from hospital	Western Pacific	High‐income economies			General health services for disease prevention
										Rehabilitation services
										Visiting health professionals
Courtney (2011)										
	On‐going	RCT	Includes 65+		Discharged from hospital	South‐East Asia	High‐income economies			General health services for disease prevention
					Physical frailty					Rehabilitation services
										Visiting health professionals
Courtney (2012)										
	Complete	RCT	Includes 65+	50%‐75% female included	Discharged from hospital	The Americas	High‐income economies			Rehabilitation services
										Visiting health professionals
							
Crotty (2002)										
	Complete	RCT	Includes 65+	50%‐75% female included	Discharged from hospital	Western Pacific	High‐income economies			Rehabilitation services
					Physical frailty					Visiting health professionals
Crotty (2003)										
	Complete	RCT	Includes 65+	50%‐75% female included	Injury	Western Pacific	High‐income economies			Rehabilitation services
										Visiting health professionals
Crotty (2008)										
	Complete	RCT	Includes 65+	50%‐75% female included	Discharged from hospital	Western Pacific	High‐income economies			Rehabilitation services
										Visiting health professionals
Cumming (2000)										
	Complete	RCT	Includes 65+	50%‐75% female included	Discharged from hospital	Western Pacific	High‐income economies			Rehabilitation services
					Physical frailty					Visiting health professionals
Cummings (1990)										
	Complete	RCT	Includes 65+		End‐of‐life	The Americas	High‐income economies			General health services for disease prevention
					Physical frailty					Visiting health professionals
Cunliffe (2004)										
	Complete	RCT	Includes 75+	50%‐75% female included	Discharged from hospital	European	High‐income economies			Rehabilitation services
										Visiting health professionals
Cutchin (2009)										
	On‐going	RCT	Includes 75+	50%‐75% female included	Physical frailty	The Americas	High‐income economies			Health promotion services
										Visiting health professionals
Dalby (2000)										
	Complete	RCT	Includes 65+	50%‐75% female included	Discharged from hospital	The Americas	High‐income economies			General health services for disease prevention
					Injury					Visiting health professionals
				Physical frailty			
Daly (2015)										
	On‐going	RCT	Includes 65+		Injury	Western Pacific	High‐income economies			Rehabilitation services
										Visiting health professionals
Danilovich et al. (2017)										
	On‐going	RCT	Includes <65		Noncommunicable disease	The Americas	High‐income economies			Rehabilitation services
			Includes 65+		Physical frailty					Visiting health professionals
Dano (2016)										
	Complete	RCT	Includes <65		Noncommunicable disease	European	High‐income economies			General health services for disease prevention
			Includes 65+							Visiting health professionals
Dechamps (2010)										
	Complete	RCT	Includes 65+	75%‐100% female included	Noncommunicable disease	European	High‐income economies			Rehabilitation services
										Visiting health professionals
Di Monaco (2008)										
	Complete	RCT	Includes 65+	75%‐100% female included	Injury	European	High‐income economies			Rehabilitation services
										Visiting health professionals
Di Pollina (2017)										
	Complete	RCT	Includes <65	50%‐75% female included	Physical frailty	European	High‐income economies			General health services for disease prevention
			Includes 65+							Visiting health professionals
Dias (2008)										
	Complete	RCT	Includes <65	25%‐50% female included	Dementia	South‐East Asia	Lower‐middle‐income economies			General health services for disease prevention
			Includes 65+		Noncommunicable disease					Visiting health professionals
Donald (1995)										
	Complete	RCT	Includes 65+	50%‐75% female included	Discharged from hospital	European	High‐income economies			Rehabilitation services
										Visiting health professionals
Donat (2007)										
	Complete	RCT	Includes 65+	50%‐75% female included	Injury	European	High‐income economies			Rehabilitation services
										Visiting health professionals
Dorner (2013)										
	On‐going	RCT	Includes 65+		Physical frailty	European	High‐income economies			Health promotion services
										Rehabilitation services
										Visiting lay service providers
Dorresteijn (2016)										
	Complete	RCT	Includes 65+	50%‐75% female included	Injury	European	High‐income economies			General health services for disease prevention
					Physical frailty					Visiting health professionals
Dow (2013)										
	On‐going	RCT	Includes <65		Physical frailty	Western Pacific	High‐income economies	Family and caregiver support		Rehabilitation services
			Includes 65+							Visiting health professionals
							
Draper (2008)										
	Complete	RCT	Includes 65+	50%‐75% female included	Dementia	European	High‐income economies			Rehabilitation services
					Noncommunicable disease					Visiting health professionals
Draper (2016)										
	Complete	RCT	Includes <65	50%‐75% female included	Noncommunicable disease	The Americas	High‐income economies			Rehabilitation services
			Includes 65+							Visiting health professionals
Duffy (2010)										
	Complete	RCT	Includes 65+	50%‐75% female included	Noncommunicable disease	The Americas	High‐income economies			General health services for disease prevention
										Visiting health professionals
Edgren (2015)										
	Complete	RCT	Includes <65	50%‐75% female included	Injury	European	High‐income economies			Rehabilitation services
			Includes 65+							Visiting health professionals
Eloniemi‐Sulkava (2001)										
	Complete	RCT	Includes 65+	50%‐75% female included	Noncommunicable disease	European	High‐income economies	Family and caregiver support		General health services for disease prevention
										Visiting health professionals
Eloniemi‐Sulkava (2009)										
	Complete	RCT	Includes <65	25%‐50% female included	Dementia	European	High‐income economies			General health services for disease prevention
			Includes 65+		Noncommunicable disease					Rehabilitation services
										Visiting health professionals
Engberg (2016)										
	Complete	RCT	Includes 65+	75%‐100% female included	Noncommunicable disease	The Americas	High‐income economies			Rehabilitation services
										Visiting health professionals
Enguidanos (2012)										
	Complete	RCT	Includes <65	50%‐75% female included	Discharged from hospital	The Americas	High‐income economies			General health services for disease prevention
			Includes 65+							Visiting health professionals
Eriksen (2016)										
	On‐going	RCT	Includes <65	50%‐75% female included		European	High‐income economies			Rehabilitation services
			Includes 65+							Visiting health professionals
Fabacher (1994)										
	Complete	RCT	Includes 65+	0%‐25% female included	Noncommunicable disease	The Americas	High‐income economies			General health services for disease prevention
										Visiting health professionals
										Visiting lay service providers
Faber (2006)										
	Complete	RCT	Includes <65	75%‐100% female included	Physical frailty	European	High‐income economies			Rehabilitation services
			Includes 65+							Visiting health professionals
Fahlström (2018)										
	Complete	RCT	Includes 65+	50%‐75% female included	Injury	European	High‐income economies			Rehabilitation services
										Visiting health professionals
Fairhall (2012)										
	Complete	RCT	Includes 65+	50%‐75% female included	Discharged from hospital	Western Pacific	High‐income economies			Rehabilitation services
										Visiting health professionals
Fairhall (2014)										
	Complete	RCT	Includes 65+	50%‐75% female included	Physical frailty	Western Pacific	High‐income economies		Personal mobility and transportation devices	General health services for disease prevention
										Rehabilitation services
										Visiting health professionals
Fairhall et al. (2015)										
	On‐going	RCT	Includes 65+		Physical frailty	Western Pacific	High‐income economies		Personal mobility and transportation devices	General health services for disease prevention
										Rehabilitation services
										Visiting health professionals
Fairhall (2017)										
	Complete	RCT	Includes 65+	50%‐75% female included	Physical frailty	Western Pacific	High‐income economies			General health services for disease prevention
										Rehabilitation services
										Visiting health professionals
Farag (2015)										
	Complete	RCT	Includes 75+	50%‐75% female included	Discharged from hospital	Western Pacific	High‐income economies			Rehabilitation services
										Visiting health professionals
Farag (2016)										
	Complete	RCT	Includes <65	75%‐100% female included	Discharged from hospital	Western Pacific	High‐income economies			General health services for disease prevention
			Includes 65+							Rehabilitation services
										Visiting health professionals
Fasce (2018)										
	On‐going	RCT	Includes <65		Discharged from hospital	The Americas	High‐income economies			General health services for disease prevention
			Includes 65+		Noncommunicable disease					Rehabilitation services
										Visiting health professionals
Favela (2013)										
	Complete	RCT	Includes <65	50%‐75% female included	Discharged from hospital	The Americas	Upper‐middle‐income economies			General health services for disease prevention
			Includes 65+		Physical frailty					Visiting health professionals
Feldman (2004)										
	Complete	RCT	Includes 65+	50%‐75% female included	Noncommunicable disease	The Americas	High‐income economies			General health services for disease prevention
										Visiting health professionals
Ferrer (2014)										
	Complete	RCT	Includes 85+	50%‐75% female included	Injury	The Americas	High‐income economies			General health services for disease prevention
					Noncommunicable disease					Visiting health professionals
Ferrer‐Garcia (2011)										
	Complete	RCT	Includes <65	50%‐75% female included	Noncommunicable disease	European	High‐income economies			Rehabilitation services
			Includes 65+							Visiting health professionals
Fiatarone (1994)										
	Complete	RCT	Includes 65+	50%‐75% female included	Physical frailty	The Americas	High‐income economies			Rehabilitation services
Finnema (2005)										
	Complete	RCT	Includes 65+	75%‐100% female included	Care dependent	European	High‐income economies			General health services for disease prevention
					Dementia					Visiting health professionals
					Noncommunicable disease					
Fleming (2004)										
	Complete	RCT	Includes 75+	50%‐75% female included	Discharged from hospital	European	High‐income economies			Rehabilitation services
										Visiting health professionals
Flood (2005)										
	Complete	RCT			Physical frailty	European	High‐income economies			Rehabilitation services
										Visiting health professionals
Fontan (2010)										
	Complete	RCT	Includes 65+			European	High‐income economies			General health services for disease prevention
										Visiting health professionals
Forsberg (2011)										
	Complete	RCT	Includes 65+	50%‐75% female included	Injury	European	High‐income economies			Rehabilitation services
										
Forster (1996)										
	Complete	RCT	Includes <65	25%‐50% female included	Noncommunicable disease	European	High‐income economies			Visiting health professionals
			Includes 65+							
Frese (2012)										
	Complete	RCT	Includes 65+	50%‐75% female included	Noncommunicable disease	European	High‐income economies			General health services for disease prevention
										Visiting health professionals
Friedman (2014)										
	Complete	RCT	Includes 65+	50%‐75% female included	Noncommunicable disease	The Americas	High‐income economies			General health services for disease prevention
					Physical frailty					Visiting health professionals
Gagnon (1999)										
	Complete	RCT	Includes 65+	50%‐75% female included	Physical frailty	The Americas	High‐income economies			General health services for disease prevention
										Visiting health professionals
Garcia‐Pena (2001)										
	Complete	RCT	Includes <65	50%‐75% female included	Noncommunicable disease	The Americas	Upper‐middle‐income economies			General health services for disease prevention
			Includes 65+							Visiting health professionals
Garcia‐Pena (2002)										
	Complete	RCT	Includes <65	50%‐75% female included	Noncommunicable disease	The Americas	Upper‐middle‐income economies			General health services for disease prevention
			Includes 65+							Visiting health professionals
Gawler (2016)										
	Complete	RCT	Includes 65+	50%‐75% female included	Injury	European	High‐income economies			Rehabilitation services
										Visiting health professionals
Giangregorio (2018)										
	Complete	RCT	Includes 65+	75%‐100% female included	Injury	The Americas	High‐income economies			Rehabilitation services
										Visiting health professionals
Gill (2002)										
	Complete	RCT	Includes 75+	75%‐100% female included	Physical frailty	The Americas	High‐income economies			Rehabilitation services
										Visiting health professionals
Gill (2004)										
	Complete	RCT	Includes 75+	75%‐100% female included	Physical frailty	The Americas	High‐income economies			Rehabilitation services
										Visiting health professionals
Gitlin (2001)										
	Complete	RCT	Includes 75+	50%‐75% female included	Dementia	The Americas	High‐income economies			Visiting health professionals
					Noncommunicable disease					
Gitlin (2006)										
	Complete	RCT	Includes 65+	75%‐100% female included	Physical frailty	The Americas	High‐income economies			Rehabilitation services
										Visiting health professionals
Gitlin (2008)										
	Complete	RCT	Includes 75+	25%‐50% female included	Dementia	The Americas	High‐income economies			General health services for disease prevention
					Noncommunicable disease					Visiting health professionals
Gitlin (2009)										
	Complete	RCT	Includes 75+	75%‐100% female included	Physical frailty	The Americas	High‐income economies			General health services for disease prevention
										Visiting health professionals
Gitlin (2010)										
	Complete	RCT	Includes <65		Dementia	The Americas	High‐income economies			General health services for disease prevention
			Includes 65+		Noncommunicable disease					Visiting health professionals
Gitlin (2014)										
	Complete	RCT	Includes <65	75%‐100% female included	Noncommunicable disease	The Americas	High‐income economies			General health services for disease prevention
			Includes 65+							Visiting health professionals
Gitlin (2018)										
	Complete	RCT	Includes 65+	0%‐25% female included	Dementia	The Americas	High‐income economies			Rehabilitation services
					Noncommunicable disease					Visiting health professionals
				Physical frailty			
Gladman (1993)										
	Complete	RCT	Includes 65+	25%‐50% female included	Noncommunicable disease	European	High‐income economies			Rehabilitation services
										Visiting health professionals
Godwin (2016)										
	Complete	RCT	Includes 75+	50%‐75% female included		The Americas	High‐income economies			General health services for disease prevention
										Visiting health professionals
Gozalo (2014)										
	Complete	RCT	Includes 75+	75%‐100% female included	Care dependent	The Americas	High‐income economies	Personal care		
					Dementia					
					Noncommunicable disease					
Graff (2008)										
	Complete	RCT	Includes 65+	50%‐75% female included	Dementia	European	High‐income economies			Rehabilitation services
					Noncommunicable disease					Visiting health professionals
Granbom (2017)										
	Complete	RCT	Includes 65+	50%‐75% female included	Physical frailty	European	High‐income economies			General health services for disease prevention
										Visiting health professionals
Graves (2009)										
	Complete	RCT	Includes 75+	50%‐75% female included	Discharged from hospital	Western Pacific	High‐income economies			General health services for disease prevention
										Visiting health professionals
Grimmer (2013)										
	On‐going	RCT	Includes 65+		Discharged from hospital	Western Pacific	High‐income economies			Health promotion services
										Visiting health professionals
Gronstedt (2013)										
	Complete	RCT	Includes <65	50%‐75% female included	Physical frailty	European	High‐income economies			Rehabilitation services
			Includes 65+							Visiting health professionals
Gustafsson (2012)										
	Complete	RCT	Includes 75+	50%‐75% female included		European	High‐income economies			General health services for disease prevention
			Includes 85+							Visiting health professionals
Haastregt (2000)										
	Complete	RCT	Includes 65+	50%‐75% female included	Injury	European	High‐income economies			Visiting health professionals
			Includes 75+							
Haider (2017)										
	Complete	RCT	Includes 65+	75%‐100% female included	Physical frailty	European	High‐income economies			Rehabilitation services
			Includes 75+							Visiting lay service providers
			Includes 85+							
Haider (2017)										
	Complete	RCT	Includes 65+	75%‐100% female included	Physical frailty	European	High‐income economies			Rehabilitation services
			Includes 75+							Visiting lay service providers
			Includes 85+							
Hall (1992)										
	Complete	RCT	Includes 65+	75%‐100% female included	Physical frailty	The Americas	High‐income economies			Health promotion services
			Includes 75+							Visiting health professionals
			Includes 85+							
Hammar (2009)										
	Complete	RCT	Includes 65+	50%‐75% female included	Discharged from hospital	European	High‐income economies			General health services for disease prevention
										Visiting health professionals
Hansen (1992)										
	Complete	RCT	Includes 75+	25%‐50% female included	Discharged from hospital	European	High‐income economies			Health promotion services
										Visiting health professionals
Hansen (1995)										
	Complete	RCT	Includes <65	50%‐75% female included	Discharged from hospital	European	High‐income economies			General health services for disease prevention
			Includes 65+							Visiting health professionals
			Includes 75+							
			Includes 85+							
Harris (2005)										
	Complete	RCT	Includes <65	75%‐100% female included	Discharged from hospital	Western Pacific	High‐income economies			General health services for disease prevention
			Includes 65+							Visiting health professionals
Harvey (2014)										
	Complete	RCT	Includes 65+	50%‐75% female included	Discharged from hospital	Western Pacific	High‐income economies			General health services for disease prevention
			Includes 75+							Visiting health professionals
		Includes 85+					
Hauer (2017)										
	Complete	RCT	Includes 65+	75%‐100% female included	Discharged from hospital	European	High‐income economies			Rehabilitation services
										Visiting health professionals
Helbostad (2004)										
	Complete	RCT	Includes 75+	75%‐100% female included	Injury	European	High‐income economies			
					Noncommunicable disease					
Hendriks (2008)										
	Complete	RCT	Includes 65+	50%‐75% female included	Injury	European	High‐income economies			General health services for disease prevention
										Visiting health professionals
							
Herfjord (2014)										
	Complete	RCT	Includes 75+	50%‐75% female included	Noncommunicable disease	European	High‐income economies			General health services for disease prevention
			Includes 85+							Rehabilitation services
										Visiting health professionals
Hewitt (2018)										
	Complete	RCT	Includes 65+	50%‐75% female included	Care dependent	Western Pacific	High‐income economies			Rehabilitation services
			Includes 75+							
			Includes 85+							
Hinrichs (2015)										
	Complete	RCT	Includes 75+	50%‐75% female included	Noncommunicable disease	European	High‐income economies			Rehabilitation services
			Includes 85+							
Hinrichs (2016)										
	Complete	RCT	Includes 65+	50%‐75% female included	Care dependent	European	High‐income economies			General health services for disease prevention
					Noncommunicable disease					Rehabilitation services
										Visiting health professionals
Hoenig (2015)										
	Complete	RCT	Includes 65+	0%‐25% female included		The Americas	High‐income economies		Personal mobility and transportation devices	
			Includes 75+							
Holland (2005)										
	Complete	RCT	Includes 85+		Discharged from hospital	European	High‐income economies			General health services for disease prevention
										Visiting health professionals
Holland (2017)										
	Complete	RCT	Includes 65+	25%‐50% female included	Noncommunicable disease	Western Pacific	High‐income economies			Rehabilitation services
			Includes 75+							
Houles (2010)										
	Complete	RCT	Includes 75+	50%‐75% female included	Physical frailty	European	High‐income economies			General health services for disease prevention
			Includes 85+							Visiting health professionals
							
Hsu (2016)										
	Complete	RCT	Includes 65+	50%‐75% female included	Physical frailty	Western Pacific	High‐income economies			Rehabilitation services
										Visiting health professionals
Hsu (2016)										
	Complete	RCT	Includes 65+	50%‐75% female included	Care dependent	Western Pacific	High‐income economies			Rehabilitation services
										Visiting health professionals
Huang (1998)										
	Complete	RCT	Includes 65+	25%‐50% female included		Western Pacific	High‐income economies			General health services for disease prevention
			Includes 75+							Visiting health professionals
Huang (2013)										
	Complete	RCT	Includes 65+	50%‐75% female included	Dementia	Western Pacific	High‐income economies			General health services for disease prevention
			Includes 75+		Noncommunicable disease					
			Includes 85+							
Hughes (1992)										
	Complete	RCT	Includes <65	25%‐50% female included	Physical frailty	The Americas	High‐income economies			General health services for disease prevention
			Includes 65+							Visiting health professionals
Hughes (2000)										
	Complete	RCT	Includes <65	0%‐25% female included	Discharged from hospital	The Americas	High‐income economies			Health promotion services
			Includes 65+		Noncommunicable disease					Visiting health professionals
					Physical frailty					
Hunger (2015)										
	Complete	RCT	Includes 65+	25%‐50% female included	Discharged from hospital	European	High‐income economies			General health services for disease prevention
										Visiting health professionals
Wang et al. (2016)										
	Complete	RCT	Includes <65	75%‐100% female included	Injury	Western Pacific	High‐income economies			Rehabilitation services
			Includes 65+							Visiting health professionals
Iliffe (2014)										
	Complete	RCT	Includes 65+	50%‐75% female included	Injury	European	High‐income economies			Rehabilitation services
										Visiting health professionals
Imhof (2012)										
	Complete	RCT	Includes 75+	50%‐75% female included	Care dependent	European	High‐income economies			General health services for disease prevention
					Noncommunicable disease					Visiting health professionals
Inglis (2006)										
	Complete	RCT	Includes 65+	0%‐25% female included	Noncommunicable disease	Western Pacific	High‐income economies			Visiting health professionals
			Includes 75+							
Isrctn (2018)										
	On‐going	RCT	Includes <65		Dementia	European	High‐income economies			Rehabilitation services
			Includes 65+		Noncommunicable disease					Visiting health professionals
Jakobsen (2007)										
Complete	RCT	Includes 75+	50%‐75% female included	Discharged from hospital	European	High‐income economies	Visiting health professionals
Jensen (2002)										
	Complete	RCT	Includes 75+	50%‐75% female included	Injury	European	High‐income economies		Personal mobility and transportation devices	Rehabilitation services
Jingna (2012)										
	Complete	RCT	Includes 75+	75%‐100% female included	Physical frailty	Western Pacific	Upper‐middle‐income economies			General health services for disease prevention
										Visiting health professionals
Joaquim (2017)										
	Complete	RCT	Includes <65	50%‐75% female included	Noncommunicable disease	The Americas	Upper‐middle‐income economies			General health services for disease prevention
			Includes 65+							Visiting health professionals
Johansson (2001)										
	Complete	RCT	Includes <65	50%‐75% female included	Noncommunicable disease	European	High‐income economies			General health services for disease prevention
			Includes 65+							Visiting health professionals
Johansson (2003)										
	Complete	RCT	Includes 65+	50%‐75% female included	Noncommunicable disease	European	High‐income economies			General health services for disease prevention
			Includes 75+							Visiting health professionals
Jolly (2009)										
	Complete	RCT	Includes <65	0%‐25% female included	Discharged from hospital	European	High‐income economies			Rehabilitation services
			Includes 65+		Noncommunicable disease					Visiting health professionals
Kalra (2000)										
	Complete	RCT	Includes 65+	50%‐75% female included	Discharged from hospital	European	High‐income economies			General health services for disease prevention
					Noncommunicable disease					Visiting health professionals
Kane (1984)										
Complete	RCT	Includes <65	0%‐25% female included	End‐of‐life	The Americas	High‐income economies	Long term care services
Kanemaru (2010)										
	Complete	RCT	Includes <65	75%‐100% female included	Noncommunicable disease	Western Pacific	High‐income economies			Rehabilitation services
			Includes 65+							Visiting health professionals
Kapan (2017)										
	Complete	RCT	Includes 65+	25%‐50% female included	Physical frailty	European	High‐income economies	Friendly visits		Rehabilitation services
			Includes 75+							Visiting lay service providers
		Includes 85+						
Kapan (2017)										
	Complete	RCT	Includes 65+	75%‐100% female included	Physical frailty	European	High‐income economies			General health services for disease prevention
										Rehabilitation services
										Visiting lay service providers
Karinkanta (2007)										
	Complete	RCT	Includes 65+	75%‐100% female included		European	High‐income economies			Rehabilitation services
			Includes 75+							
Karlsson (2016)										
	Complete	RCT	Includes 65+	50%‐75% female included	Injury	European	High‐income economies			Rehabilitation services
										Visiting health professionals
Kerr (2018)										
	Complete	RCT	Includes 65+	50%‐75% female included		The Americas	High‐income economies			Rehabilitation services
			Includes 75+							
		Includes 85+				
Kerse (2010)										
	Complete	RCT	Includes 75+	50%‐75% female included	Noncommunicable disease	Western Pacific	High‐income economies			Rehabilitation services
										Visiting health professionals
Kim (2011)										
	Complete	RCT	Includes 65+	50%‐75% female included	Physical frailty	South‐East Asia	High‐income economies			Rehabilitation services
										Visiting health professionals
King et al. (2012)										
	Complete	RCT	Includes 65+	50%‐75% female included	Physical frailty	Western Pacific	High‐income economies			General health services for disease prevention
										Visiting health professionals
King et al. (2012)										
	Complete	RCT	Includes 75+	50%‐75% female included	Care dependent	Western Pacific	High‐income economies			General health services for disease prevention
										Visiting health professionals
Kjerstad (2016)										
	Complete	RCT	Includes 65+	50%‐75% female included	Physical frailty	European	High‐income economies			Rehabilitation services
										Visiting health professionals
Klug (2011)										
	Complete	RCT	Includes <65	75%‐100% female included	Noncommunicable disease	European	High‐income economies			General health services for disease prevention
			Includes 65+							Visiting health professionals
Kocic (2018)										
	Complete	RCT	Includes 65+	50%‐75% female included		European	Upper‐middle‐income economies			Rehabilitation services
										Visiting health professionals
Kohei (2016)										
	Complete	RCT	Includes <65	50%‐75% female included	Noncommunicable disease	Western Pacific	High‐income economies			Rehabilitation services
			Includes 65+							Visiting health professionals
Kono (2004)										
	Complete	RCT	Includes 65+	75%‐100% female included	Care dependent	Western Pacific	High‐income economies			General health services for disease prevention
					Physical frailty					Visiting health professionals
Kono (2012)										
	Complete	RCT	Includes 65+	50%‐75% female included	Care dependent	Western Pacific	High‐income economies			Health promotion services
					Physical frailty					Visiting health professionals
Kono (2013)										
	Complete	RCT	Includes 75+	50%‐75% female included	Care dependent	Western Pacific	High‐income economies			General health services for disease prevention
										Visiting health professionals
Kono (2014)										
	On‐going	RCT	Includes 65+		Physical frailty	Western Pacific	High‐income economies			Visiting health professionals
									
						
Kono (2016)										
	Complete	RCT	Includes 65+	50%‐75% female included	Care dependent	Western Pacific	High‐income economies			General health services for disease prevention
										Visiting health professionals
Kronborg (2006)										
	Complete	RCT	Includes 75+	50%‐75% female included	Care dependent	European	High‐income economies			General health services for disease prevention
										Visiting health professionals
Kukkonen‐Harjula (2018)										
	On‐going	RCT	Includes 75+	75%‐100% female included	Physical frailty	European	High‐income economies			Rehabilitation services
										Visiting health professionals
Kwok (2004)										
	Complete	RCT	Includes 75+	25%‐50% female included	Discharged from hospital	Western Pacific	High‐income economies			Visiting health professionals
					Noncommunicable disease					
Kwok (2016)										
	Complete	RCT	Includes <65		Noncommunicable disease	Western Pacific	High‐income economies			Health promotion services
			Includes 65+							
Kyrdalen (2014)										
	Complete	RCT	Includes 75+	25%‐50% female included	Injury	European	High‐income economies			Rehabilitation services
										Visiting health professionals
Lam (2018)										
	Complete	RCT	Includes 75+	50%‐75% female included	Physical frailty	Western Pacific	High‐income economies			Rehabilitation services
Lannin (2007)										
	Complete	RCT	Includes 65+	75%‐100% female included	Discharged from hospital	Western Pacific	High‐income economies			Rehabilitation services
										Visiting health professionals
Latham (2014)										
	Complete	RCT	Includes 65+	50%‐75% female included	Injury	The Americas	High‐income economies			Rehabilitation services
			Includes 75+							
Latour (2007)										
	Complete	RCT	Includes <65	25%‐50% female included	Discharged from hospital	European	High‐income economies			General health services for disease prevention
			Includes 65+							Visiting health professionals
Lattanzio (2001)										
	Complete	RCT	Includes 65+	75%‐100% female included	Noncommunicable disease	The Americas	High‐income economies			Rehabilitation services
										Visiting health professionals
Leavitt (2018)										
	Complete	RCT	Includes 65+	25%‐50% female included	Discharged from hospital	The Americas	High‐income economies			General health services for disease prevention
					Noncommunicable disease					Visiting health professionals
Lee (2006)										
	Complete	RCT	Includes 65+	25%‐50% female included	Noncommunicable disease	Western Pacific	High‐income economies			Rehabilitation services
										Visiting health professionals
Lenaghan (2007)										
	Complete	RCT	Includes 75+	50%‐75% female included	Noncommunicable disease	European	High‐income economies			General health services for disease prevention
										Visiting health professionals
Levine (2012)										
	Complete	RCT	Includes 75+	50%‐75% female included	Noncommunicable disease	The Americas	High‐income economies			General health services for disease prevention
					Physical frailty					Visiting health professionals
Lewin (2013)										
Complete	RCT	Includes 65+	50%‐75% female included	Care dependent	Western Pacific	High‐income economies	Rehabilitation services
Lewin (2014)										
	Complete	RCT	Includes 65+	50%‐75% female included	Physical frailty	Western Pacific	High‐income economies			Rehabilitation services
										
Li (2013)										
	Complete	RCT	Includes 65+	50%‐75% female included	Care dependent	The Americas	High‐income economies	Personal care		General health services for disease prevention
										Visiting health professionals
Li (2015)										
	Complete	RCT	Includes 75+	0%‐25% female included	Discharged from hospital	Western Pacific	Upper‐middle‐income economies			Rehabilitation services
					Noncommunicable disease					Visiting health professionals
Liang (1984)										
	Complete	RCT	Includes 65+	75%‐100% female included	Noncommunicable disease	The Americas	High‐income economies			Rehabilitation services
										Visiting health professionals
Liang (1986)										
	Complete	RCT	Includes 75+	75%‐100% female included	Care dependent	The Americas	High‐income economies			Rehabilitation services
										Visiting health professionals
Liddle (1996)										
	Complete	RCT	Includes 65+	50%‐75% female included		Western Pacific	High‐income economies			Visiting health professionals
			Includes 75+							
			Includes 85+						
Liimatta (2017)										
	Complete	RCT	Includes 75+	50%‐75% female included	Noncommunicable disease	European	High‐income economies			General health services for disease prevention
										Visiting health professionals
Lin (2007)										
	Complete	RCT	Includes 65+		Injury	Western Pacific	High‐income economies			Rehabilitation services
					Noncommunicable disease					Visiting health professionals
						
Lin (2010)										
	Complete	RCT	Includes 75+	25%‐50% female included	Dementia	Western Pacific	High‐income economies			Rehabilitation services
			Includes 85+	50%‐75% female included	Noncommunicable disease					
Lindegaard (2017)										
	Complete	RCT	Includes 75+	75%‐100% female included	Care dependent	European	High‐income economies			General health services for disease prevention
					Discharged from hospital					Visiting health professionals
					Noncommunicable disease					
Lindegaard‐Pedersen (2015)										
	On‐going	RCT	Includes 75+		Discharged from hospital	European	High‐income economies			General health services for disease prevention
					Noncommunicable disease					Visiting health professionals
Liu and Lai (2014)										
	Complete	RCT	Includes 75+	50%‐75% female included	Discharged from hospital	Western Pacific	High‐income economies			General health services for disease prevention
										Rehabilitation services
										Visiting health professionals
Liu (2015)										
	Complete	RCT	Includes <65	50%‐75% female included	Dementia	The Americas	High‐income economies			General health services for disease prevention
			Includes 65+		Noncommunicable disease					Visiting health professionals
Locher (2013)										
	Complete	RCT	Includes 65+	75%‐100% female included	Noncommunicable disease	The Americas	High‐income economies			Visiting health professionals
			Includes 75+							
			Includes 85+							
Logan (2004)										
	Complete	RCT	Includes 65+	50%‐75% female included	Noncommunicable disease	European	High‐income economies			Rehabilitation services
			Includes 75+							
Lok (2017)										
	Complete	RCT	Includes 65+	25%‐50% female included	Noncommunicable disease	European	Upper‐middle‐income economies			Rehabilitation services
										Visiting health professionals
Luck (2013)										
	Complete	RCT	Includes 85+		Physical frailty	European	High‐income economies			Visiting health professionals
Lyons (2016)										
	Complete	RCT	Includes 65+	50%‐75% female included	Noncommunicable disease	The Americas	High‐income economies			Rehabilitation services
										Visiting health professionals
MacIntyre (1999)										
	Complete	RCT	Includes 75+	50%‐75% female included	Care dependent	The Americas	High‐income economies	Friendly visits		
					Social isolation					
Mahoney (2007)										
	Complete	RCT	Includes 75+	75%‐100% female included	Injury	The Americas	High‐income economies			Rehabilitation services
			Includes 85+							Visiting health professionals
Maiers (2014)										
	Complete	RCT	Includes 65+	25%‐50% female included	Noncommunicable disease	The Americas	High‐income economies			Rehabilitation services
			Includes 75+							
Mangione (2005)										
	Complete	RCT	Includes 65+	75%‐100% female included	Discharged from hospital	The Americas	High‐income economies			Rehabilitation services
					Injury					Visiting health professionals
Mangione et al. (2010)										
	Complete	RCT	Includes 75+	75%‐100% female included	Injury	The Americas	High‐income economies			Rehabilitation services
			Includes 85+							
Mann (1999)										
	Complete	RCT	Includes 75+	75%‐100% female included	Physical frailty	The Americas	High‐income economies		Personal mobility and transportation devices	Visiting health professionals
Marek (2014)										
	Complete	RCT	Includes 75+	50%‐75% female included	Noncommunicable disease	The Americas	High‐income economies			Visiting health professionals
										
Markle‐Reid (2003)										
	Complete	RCT	Includes 75+	75%‐100% female included	Physical frailty	The Americas	High‐income economies			General health services for disease prevention
										Health promotion services
							Visiting health professionals
Markle‐Reid (2006)										
	Complete	RCT	Includes 75+	75%‐100% female included	Physical frailty	The Americas	High‐income economies	Homemaking		General health services for disease prevention
			Includes 85+					Personal care		Visiting health professionals
Markle‐Reid (2010)										
	Complete	RCT	Includes 75+	50%‐75% female included	Injury	The Americas	High‐income economies	Transportation		General health services for disease prevention
								Visiting health professionals
Markle‐Reid et al. (2017)										
	Complete	RCT	Includes 65+	50%‐75% female included	Noncommunicable disease	The Americas	High‐income economies			General health services for disease prevention
										Health promotion services
										Visiting health professionals
Martin (1994)										
	Complete	RCT	Includes 75+	75%‐100% female included	Discharged from hospital	European	High‐income economies			General health services for disease prevention
										Visiting health professionals
Maru (2015)										
	Complete	RCT	Includes 65+		Discharged from hospital	Western Pacific	High‐income economies			General health services for disease prevention
					Noncommunicable disease					Visiting health professionals
Matzen (2007)										
	Complete	RCT	Includes 65+	50%‐75% female included	Noncommunicable disease	European	High‐income economies			General health services for disease prevention
										Visiting health professionals
Mayo (2008)										
	Complete	RCT	Includes 65+	25%‐50% female included	Discharged from hospital	The Americas	High‐income economies			General health services for disease prevention
					Noncommunicable disease					Visiting health professionals
McCorkle (1989)										
	Complete	RCT	Includes <65	25%‐50% female included	Noncommunicable disease	The Americas	High‐income economies			General health services for disease prevention
			Includes 65+							Visiting health professionals
McCorkle (2000)										
	Complete	RCT	Includes <65	50%‐75% female included	Discharged from hospital	The Americas	High‐income economies			General health services for disease prevention
			Includes 65+		Noncommunicable disease					Visiting health professionals
McMurdo (1995)										
	Complete	RCT	Includes 75+	75%‐100% female included	Physical frailty	European	High‐income economies			Rehabilitation services
			Includes 85+							Visiting health professionals
McWilliam (1999)										
	Complete	RCT	Includes 65+	50%‐75% female included	Discharged from hospital	The Americas	High‐income economies			Health promotion services
			Includes 75+		Noncommunicable disease					Visiting health professionals
McWilliam (1999)										
	Complete	RCT	Includes 65+	50%‐75% female included	Discharged from hospital	The Americas	High‐income economies			Health promotion services
			Includes 75+		Noncommunicable disease					Visiting health professionals
Meisinger (2013)										
	Complete	RCT	Includes 65+	25%‐50% female included	Noncommunicable disease	European	High‐income economies			General health services for disease prevention
			Includes 75+							Visiting health professionals
Melin (1992)										
	Complete	RCT	Includes <65	50%‐75% female included	Discharged from hospital	European	High‐income economies			Rehabilitation services
			Includes 65+							Visiting health professionals
			Includes 75+							
			Includes 85+							
Melin (1993)										
	Complete	RCT	Includes 65+	50%‐75% female included	Discharged from hospital	European	High‐income economies			General health services for disease prevention
					Physical frailty					Visiting health professionals
Melin (1993)										
	Complete	RCT	Includes 65+	50%‐75% female included	Discharged from hospital	European	High‐income economies			Rehabilitation services
			Includes 75+							Visiting health professionals
			Includes 85+							
Melin (1995)										
	Complete	RCT	Includes 65+	50%‐75% female included	Discharged from hospital	European	High‐income economies			Visiting health professionals
			Includes 75+							
Melis (2008)										
	Complete	RCT	Includes 65+		Care dependent	European	High‐income economies			General health services for disease prevention
										Visiting health professionals
Melis (2008)										
	Complete	RCT	Includes 75+	75%‐100% female included	Care dependent	European	High‐income economies			General health services for disease prevention
					Noncommunicable disease					Visiting health professionals
Mihalko (1996)										
	Complete	RCT	Includes 65+	75%‐100% female included	Physical frailty	The Americas	High‐income economies			Rehabilitation services
										Visiting health professionals
Miller (2005)										
	Complete	RCT	Includes 75+	50%‐75% female included	Discharged from hospital	European	High‐income economies			Rehabilitation services
					Noncommunicable disease					Visiting health professionals
Milte (2016)										
	Complete	RCT	Includes 75+	50%‐75% female included	Injury	Western Pacific	High‐income economies			Rehabilitation services
			Includes 85+	75%‐100% female included						Visiting health professionals
Mitchell (2005)										
	Complete	RCT	Includes 65+	50%‐75% female included	Injury	European	High‐income economies			Rehabilitation services
					Noncommunicable disease					Visiting health professionals
Mohide (1990)										
	Complete	RCT	Includes 75+	25%‐50% female included	Dementia	The Americas	High‐income economies			Visiting health professionals
					Noncommunicable disease					
Molassiotis (2009)										
	Complete	RCT	Includes <65	50%‐75% female included	Noncommunicable disease	European	High‐income economies			General health services for disease prevention
			Includes 65+							Visiting health professionals
Moller (2014)										
	Complete	RCT	Includes 65+	50%‐75% female included	Injury	European	High‐income economies			Health promotion services
					Physical frailty					Visiting health professionals
Montgomery (2003)										
	Complete	RCT	Includes 75+	50%‐75% female included	Physical frailty	The Americas	High‐income economies			General health services for disease prevention
			Includes 85+							
Morris (2017)										
	Complete	RCT	Includes <65	25%‐50% female included	Noncommunicable disease	Western Pacific	High‐income economies			Rehabilitation services
			Includes 65+							Visiting health professionals
			Includes 75+							
Mortensen (2016)										
	Complete	RCT	Includes <65		Noncommunicable disease	European	High‐income economies			Rehabilitation services
			Includes 65+							Visiting health professionals
Mulrow (1994)										
	Complete	RCT	Includes 65+	50%‐75% female included	Physical frailty	The Americas	High‐income economies			Rehabilitation services
										Visiting health professionals
Murphy (2005)										
	Complete	RCT	Includes <65	25%‐50% female included	Discharged from hospital	European	High‐income economies			Rehabilitation services
			Includes 65+							Visiting health professionals
Naunton (2003)										
	Complete	RCT	Includes 65+	50%‐75% female included	Discharged from hospital	Western Pacific	High‐income economies			General health services for disease prevention
										Visiting health professionals
Naylor (1999)										
	Complete	RCT	Includes 65+	50%‐75% female included	Discharged from hospital	The Americas	High‐income economies			General health services for disease prevention
			Includes 75+							Visiting health professionals
Naylor (2004)										
	Complete	RCT	Includes 65+	50%‐75% female included	Noncommunicable disease	The Americas	High‐income economies			Visiting health professionals
			Includes 75+							
Naylor (2004)										
	Complete	RCT	Includes 65+	50%‐75% female included	Discharged from hospital	The Americas	High‐income economies			General health services for disease prevention
					Noncommunicable disease					Visiting health professionals
Nazareth (2001)										
	Complete	RCT	Includes 75+		Discharged from hospital	European	High‐income economies			General health services for disease prevention
										Visiting health professionals
Nct (2005)										
	Complete	RCT	Includes 75+	50%‐75% female included		European	High‐income economies			General health services for disease prevention
										Visiting health professionals
Nct (2006)										
	Complete	RCT	Includes 85+	50%‐75% female included		European	High‐income economies			Rehabilitation services
										Visiting health professionals
Nct (2011)										
	On‐going	RCT	Includes <65		Discharged from hospital	The Americas	High‐income economies			General health services for disease prevention
			Includes 65+		Noncommunicable disease					Visiting health professionals
Nct (2011)										
	On‐going	RCT	Includes 65+		Noncommunicable disease	The Americas	High‐income economies			Rehabilitation services
			Includes 75+							
Nct (2012)										
	On‐going	RCT	Includes 65+		Injury	The Americas	High‐income economies			General health services for disease prevention
					Noncommunicable disease					Visiting health professionals
Nct (2013)										
	Complete	RCT	Includes 65+		Social isolation	European	High‐income economies	Homemaking		
								Friendly visits		
Nct (2014)										
	On‐going	RCT	Includes 65+		Care dependent	Western Pacific	High‐income economies			Rehabilitation services
										Visiting health professionals
Nct (2014)										
	On‐going	RCT	Includes 65+		Noncommunicable disease	The Americas	High‐income economies			General health services for disease prevention
										Visiting health professionals
Nct (2014)										
	On‐going	RCT	Includes 65+		Discharged from hospital	The Americas	High‐income economies			Visiting health professionals
Nct (2015)										
	On‐going	RCT	Includes 75+		Care dependent	The Americas	High‐income economies		Personal mobility and transportation devices	Rehabilitation services
					Injury					Visiting health professionals
Nct (2017)										
	On‐going	RCT	Includes 65+			Western Pacific	High‐income economies			General health services for disease prevention
										Health promotion services
										Visiting health professionals
Nct (2017)										
	On‐going	RCT	Includes 65+		Physical frailty	The Americas	High‐income economies			Rehabilitation services
Nct (2017)										
	On‐going	RCT	Includes 65+		Injury	The Americas	High‐income economies			General health services for disease prevention
			Includes 75+							Health promotion services
Nct (2017)										
	On‐going	RCT	Includes <65		End‐of‐life	Western Pacific	High‐income economies			Long term care services
		Includes 65+				
Nct (2018)										
	On‐going	RCT	Includes 65+		Noncommunicable disease	The Americas	High‐income economies			Rehabilitation services
					Physical frailty					Visiting health professionals
Nct (2018)										
	On‐going	RCT	Includes 65+		Injury	European	High‐income economies			Rehabilitation services
										Visiting health professionals
Neumann (2017)										
	Complete	RCT	Includes 65+	50%‐75% female included		European	High‐income economies			General health services for disease prevention
			Includes 75+							Health promotion services
										Visiting health professionals
Nicolaides‐Bouman (2004)										
	On‐going	RCT	Includes 65+	50%‐75% female included	Care dependent	European	High‐income economies			General health services for disease prevention
										Visiting health professionals
Nielsen (1972)										
	Complete	RCT	Includes 65+	50%‐75% female included	Discharged from hospital	The Americas	High‐income economies	Homemaking		
					Injury			Personal care		
					Noncommunicable disease					
Nikolaus (1999)										
	Complete	RCT	Includes 65+	50%‐75% female included	Discharged from hospital	European	High‐income economies			General health services for disease prevention
			Includes 75+							Visiting health professionals
Nikolaus (2003)										
	Complete	RCT	Includes 75+	50%‐75% female included	Discharged from hospital	European	High‐income economies			General health services for disease prevention
					Injury					Visiting health professionals
Nobili (2004)										
	Complete	RCT	Includes 65+	50%‐75% female included	Care dependent	European	High‐income economies	Family and caregiver support		Rehabilitation services
					Dementia					Visiting health professionals
				Noncommunicable disease				
Nowalk (2001)										
	Complete	RCT	Includes 65+	75%‐100% female included		The Americas	High‐income economies			Rehabilitation services
			Includes 75+							
			Includes 85+							
Oerkild (2011)										
	Complete	RCT	Includes 65+	25%‐50% female included	Noncommunicable disease	European	High‐income economies			Rehabilitation services
										Visiting health professionals
Oerkild (2012)										
	Complete	RCT	Includes 65+	75%‐100% female included	Noncommunicable disease	European	High‐income economies			Rehabilitation services
			Includes 75+							
Olaleye (2014)										
	Complete	RCT	Includes <65	50%‐75% female included	Noncommunicable disease	African	Lower‐middle‐income economies			Rehabilitation services
			Includes 65+							
Olesen (2014)										
	Complete	RCT	Includes 65+	50%‐75% female included	Noncommunicable disease	European	High‐income economies			Visiting health professionals
			Includes 75+							
Olson (2011)										
	Complete	RCT	Includes <65	50%‐75% female included	Injury	The Americas	High‐income economies			Rehabilitation services
			Includes 65+							Visiting health professionals
Oosting (2012)										
	Complete	RCT	Includes 65+	50%‐75% female included	Physical frailty	European	High‐income economies			Rehabilitation services
			Includes 75+	75%‐100% female included						
Orrell (2017)										
	Complete	RCT	Includes 75+	75%‐100% female included	Dementia	European	High‐income economies			Rehabilitation services
			Includes 85+		Noncommunicable disease					
Ouslander (2005)										
	Complete	RCT	Includes <65	0%‐25% female included	Care dependent	The Americas	High‐income economies			Rehabilitation services
			Includes 65+							Visiting health professionals
Özdemir (2001)										
	Complete	RCT	Includes <65	25%‐50% female included	Noncommunicable disease	European	Upper‐middle‐income economies			Rehabilitation services
			Includes 65+							
Padala (2017)										
	Complete	RCT	Includes 65+	25%‐50% female included	Noncommunicable disease	The Americas	High‐income economies			Rehabilitation services
			Includes 75+							
Padula (2009)										
	Complete	RCT	Includes <65	50%‐75% female included	Noncommunicable disease	The Americas	High‐income economies			Rehabilitation services
			Includes 65+							Visiting health professionals
Papaioannou (2003)										
	Complete	RCT	Includes 65+	75%‐100% female included	Noncommunicable disease	The Americas	High‐income economies			Rehabilitation services
			Includes 75+							
Pardessus (2002)										
	Complete	RCT	Includes 75+	75%‐100% female included	Discharged from hospital	European	High‐income economies			General health services for disease prevention
					Injury					Visiting health professionals
Parker (2009)										
	Complete	RCT	Includes <65	75%‐100% female included	Care dependent	European	High‐income economies			Rehabilitation services
			Includes 65+							Visiting health professionals
Parker (2011)										
	Complete	RCT	Includes <65	25%‐50% female included	Noncommunicable disease	European	High‐income economies			Rehabilitation services
			Includes 65+							Visiting health professionals
Parsons et al. (2013)										
	Complete	RCT	Includes 65+	50%‐75% female included	Care dependent	Western Pacific	High‐income economies			General health services for disease prevention
										Visiting health professionals
Parsons (2017)										
	Complete	RCT	Includes 65+	50%‐75% female included	Physical frailty	European	High‐income economies			General health services for disease prevention
										Rehabilitation services
							Visiting health professionals
Patterson (2009)										
	Complete	RCT	Includes 65+	75%‐100% female included		Western Pacific	High‐income economies			General health services for disease prevention
										Visiting lay service providers
Pedersen (2016)										
	Complete	RCT	Includes 75+	75%‐100% female included	Discharged from hospital	European	High‐income economies			General health services for disease prevention
					Physical frailty					Visiting health professionals
Peeters (2007)										
	On‐going	RCT	Includes 65+		Injury	European	High‐income economies			General health services for disease prevention
										Visiting health professionals
Pizzi (2014)										
	Complete	RCT	Includes <65	75%‐100% female included	Noncommunicable disease	The Americas	High‐income economies			General health services for disease prevention
			Includes 65+							Visiting health professionals
			Includes 75+							
Portegijs (2013)										
	Complete	RCT	Includes 65+			European	High‐income economies			Rehabilitation services
										Visiting health professionals
Prick (2015)										
	Complete	RCT	Includes <65	25%‐50% female included	Dementia	European	High‐income economies	Family and caregiver support		Rehabilitation services
			Includes 65+		Noncommunicable disease					Visiting health professionals
Pröfener (2016)										
	Complete	RCT	Includes 65+	75%‐100% female included	Physical frailty	European	High‐income economies			General health services for disease prevention
										Visiting health professionals
Radwany (2014)										
	Complete	RCT	Includes <65	50%‐75% female included	Noncommunicable disease	The Americas	High‐income economies			General health services for disease prevention
			Includes 65+							Long term care services
							Visiting health professionals
Rasmussen (2016)										
	Complete	RCT	Includes 65+			European	High‐income economies			Rehabilitation services
										Visiting health professionals
Ray (1997)										
	Complete	RCT	Includes 65+	25%‐50% female included	Injury	The Americas	High‐income economies			General health services for disease prevention
										Visiting health professionals
Reckrey (2018)										
	On‐going	RCT	Includes 65+		Care dependent	The Americas	High‐income economies			General health services for disease prevention
										Visiting health professionals
Reeves (2004)										
	Complete	RCT	Includes 75+	50%‐75% female included	Noncommunicable disease	European	High‐income economies			Rehabilitation services
										Visiting health professionals
Regan (2017)										
	Complete	RCT	Includes 65+	25%‐50% female included	Noncommunicable disease	Western Pacific	High‐income economies			Rehabilitation services
			Includes 75+	50%‐75% female included						
Resnick (2009)										
	Complete	RCT	Includes 65+	75%‐100% female included	Noncommunicable disease	The Americas	High‐income economies			Rehabilitation services
										Visiting health professionals
Richards (1998)										
	Complete	RCT	Includes 65+	50%‐75% female included	Injury	European	High‐income economies			General health services for disease prevention
			Includes 75+		Noncommunicable disease					Visiting health professionals
Robertson et al. (2001)										
	Complete	RCT	Includes 75+	50%‐75% female included	Care dependent	Western Pacific	High‐income economies			Rehabilitation services
					Injury					Visiting health professionals
				Noncommunicable disease			
Roderick (2001)										
	Complete	RCT	Includes 65+		Noncommunicable disease	European	High‐income economies			Rehabilitation services
										Visiting health professionals
Rosendahl (2006)										
	Complete	RCT	Includes 65+		Physical frailty	European	High‐income economies			Rehabilitation services
										Visiting health professionals
Rosstad (2017)										
	Complete	RCT	Includes 65+	50%‐75% female included	Discharged from hospital	European	High‐income economies			General health services for disease prevention
					Noncommunicable disease					Visiting health professionals
Rossum (1993)										
	Complete	RCT	Includes 75+	50%‐75% female included	Care dependent	European	High‐income economies			General health services for disease prevention
										Visiting health professionals
Rubenstein (1994)										
	Complete	RCT	Includes 75+	50%‐75% female included	Care dependent	The Americas	High‐income economies			General health services for disease prevention
					Noncommunicable disease					Visiting health professionals
Runciman (1996)										
	Complete	RCT	Includes 75+		Discharged from hospital	European	High‐income economies			Visiting health professionals
									
Ryan (2006)										
	Complete	RCT	Includes 65+	50%‐75% female included	Injury	European	High‐income economies			Rehabilitation services
					Noncommunicable disease					Visiting health professionals
Rytter (2010)										
	Complete	RCT	Includes 75+	50%‐75% female included	Discharged from hospital	European	High‐income economies			General health services for disease prevention
										Visiting health professionals
Sackley (2007)										
	Complete	RCT	Includes <65	75%‐100% female included	Care dependent	European	High‐income economies			Rehabilitation services
			Includes 65+		Physical frailty					Visiting health professionals
					Social isolation					
Sackley (2009)										
	Complete	RCT	Includes 75+	75%‐100% female included	Care dependent	European	High‐income economies			Rehabilitation services
					Noncommunicable disease					Visiting health professionals
Sackley (2015)										
	Complete	RCT	Includes 75+	50%‐75% female included	Noncommunicable disease	European	High‐income economies		Personal mobility and transportation devices	Rehabilitation services
										Visiting health professionals
Sahlen (2016)										
	Complete	RCT			Noncommunicable disease	European	High‐income economies			Long term care services
Salminen (2009)										
	Complete	RCT	Includes 65+	75%‐100% female included	Injury	European	High‐income economies			General health services for disease prevention
										Visiting health professionals
Salpakoski (2014)										
	Complete	RCT	Includes 75+	75%‐100% female included	Injury	European	High‐income economies			Rehabilitation services
										Visiting health professionals
Samus (2014)										
	Complete	RCT	Includes 75+	50%‐75% female included	Care dependent	The Americas	High‐income economies			General health services for disease prevention
					Noncommunicable disease					Visiting health professionals
Sandberg (2015)										
	Complete	RCT	Includes 65+	50%‐75% female included	Physical frailty	European	High‐income economies			General health services for disease prevention
										Visiting health professionals
Sandberg (2015)										
	Complete	RCT	Includes 65+	50%‐75% female included	Care dependent	European	High‐income economies			General health services for disease prevention
					Physical frailty					Visiting health professionals
Sanford (2006)										
	Complete	RCT	Includes <65		Care dependent	The Americas	High‐income economies			Rehabilitation services
			Includes 65+							Visiting health professionals
Schnelle (1996)										
	Complete	RCT	Includes 65+	75%‐100% female included	Noncommunicable disease	The Americas	High‐income economies			Rehabilitation services
					Physical frailty					Visiting health professionals
Schnelle (2010)										
	Complete	RCT	Includes 75+	75%‐100% female included	Noncommunicable disease	The Americas	High‐income economies	Personal care		Rehabilitation services
										Visiting health professionals
Seidl (2015)										
	Complete	RCT	Includes 65+	25%‐50% female included	Noncommunicable disease	European	High‐income economies			General health services for disease prevention
			Includes 75+							Visiting health professionals
Senior (2014)										
	Complete	RCT	Includes 65+	50%‐75% female included	Physical frailty	Western Pacific	High‐income economies			General health services for disease prevention
										Rehabilitation services
							Visiting health professionals
Serra‐Rexach (2011)										
	Complete	RCT	Includes 85+	75%‐100% female included	Care dependent	European	High‐income economies			Rehabilitation services
					Noncommunicable disease					Visiting health professionals
					Physical frailty					
Sheffield (2013)										
	Complete	RCT	Includes 65+	75%‐100% female included	Physical frailty	The Americas	High‐income economies			Rehabilitation services
										Visiting health professionals
Shepperd and Iliffe (1998)										
	Complete	RCT	Includes <65		Discharged from hospital	European	High‐income economies			General health services for disease prevention
			Includes 65+							Visiting health professionals
Shepperd and Iliffe (1998)										
	Complete	RCT	Includes <65	50%‐75% female included	Injury	European	High‐income economies			General health services for disease prevention
			Includes 65+		Noncommunicable disease					Visiting health professionals
Shepperd (2017)										
	On‐going	RCT	Includes 65+		Noncommunicable disease	European	High‐income economies			General health services for disease prevention
										Visiting health professionals
Sherman (2016)										
	Complete	RCT	Includes 75+	50%‐75% female included		European	High‐income economies			General health services for disease prevention
										Visiting health professionals
Sherrington (2015)										
	Complete	RCT	Includes <65	50%‐75% female included	Discharged from hospital	Western Pacific	High‐income economies			Rehabilitation services
			Includes 65+							Visiting health professionals
Sherrington (2016)										
	On‐going	RCT	Includes <65		Injury	Western Pacific	High‐income economies			Rehabilitation services
			Includes 65+							Visiting health professionals
Shyu (2008)										
	Complete	RCT	Includes 65+	25%‐50% female included	Discharged from hospital	Western Pacific	High‐income economies	Family and caregiver support		General health services for disease prevention
										Visiting health professionals
Shyu (2016)										
	Complete	RCT	Includes <65	50%‐75% female included	Injury	Western Pacific	High‐income economies			General health services for disease prevention
			Includes 65+							Rehabilitation services
		Includes 75+					
Siggeirsdottir (2005)										
	Complete	RCT	Includes <65	50%‐75% female included	Injury	European	High‐income economies			Rehabilitation services
			Includes 65+							Visiting health professionals
Simmons (2002)										
	Complete	RCT	Includes 75+	75%‐100% female included	Noncommunicable disease	The Americas	High‐income economies			Rehabilitation services
										Visiting health professionals
Simmons (2005)										
	Complete	RCT	Includes 65+	75%‐100% female included	Noncommunicable disease	The Americas	High‐income economies	Personal care		
Sloane (2004)										
	Complete	RCT	Includes <65	75%‐100% female included	Care dependent	The Americas	High‐income economies	Personal care		
			Includes 65+		Dementia					
				Noncommunicable disease			
Steele (2008)										
	Complete	RCT	Includes <65		Noncommunicable disease	The Americas	High‐income economies			Rehabilitation services
			Includes 65+							Visiting health professionals
Steinberg (2009)										
	Complete	RCT	Includes 65+	50%‐75% female included	Dementia	The Americas	High‐income economies			Rehabilitation services
					Noncommunicable disease					Visiting health professionals
Stelmack et al. (2007)										
	Complete	RCT	Includes 65+	0%‐25% female included	Noncommunicable disease	The Americas	High‐income economies			Rehabilitation services
										Visiting health professionals
Stevens (2001)										
	Complete	RCT	Includes 75+	50%‐75% female included		Western Pacific	High‐income economies			Rehabilitation services
										Visiting health professionals
Stevens‐Lapsley (2016)										
	Complete	RCT	Includes 65+	50%‐75% female included	Discharged from hospital	The Americas	High‐income economies			Rehabilitation services
										Visiting health professionals
Stewart et al. (2005)										
	Complete	RCT	Includes 65+		Physical frailty	European	High‐income economies			Rehabilitation services
										Visiting health professionals
Stewart (2012)										
	Complete	RCT	Includes <65	25%‐50% female included	Noncommunicable disease	Western Pacific	High‐income economies			General health services for disease prevention
			Includes 65+							Visiting health professionals
Stuck et al. (1995)										
	Complete	RCT	Includes 75+	50%‐75% female included	Noncommunicable disease	European	High‐income economies			General health services for disease prevention
										Visiting health professionals
Stuck et al. (1995)										
	Complete	RCT	Includes 75+	50%‐75% female included		The Americas	High‐income economies			General health services for disease prevention
										Visiting health professionals
Stuck (2000)										
	Complete	RCT	Includes 75+	75%‐100% female included	Care dependent	European	High‐income economies			General health services for disease prevention
										Visiting health professionals
Suominen (2015)										
	Complete	RCT	Includes 65+	25%‐50% female included	Care dependent	European	High‐income economies			General health services for disease prevention
					Noncommunicable disease					Visiting health professionals
Suttanon (2013)										
	Complete	RCT	Includes 65+	50%‐75% female included	Noncommunicable disease	Western Pacific	High‐income economies			Rehabilitation services
										Visiting health professionals
Szanton (2014)										
	On‐going	RCT	Includes 65+		Physical frailty	The Americas	High‐income economies			General health services for disease prevention
										Rehabilitation services
										Visiting health professionals
Talley (2017)										
	Complete	RCT	Includes 75+	75%‐100% female included	Noncommunicable disease	The Americas	High‐income economies			General health services for disease prevention
										Rehabilitation services
							Visiting health professionals
Taube (2017)										
	Complete	RCT	Includes 65+	25%‐50% female included	Noncommunicable disease	European	High‐income economies			General health services for disease prevention
										Visiting health professionals
Thomas (2016)										
	Complete	RCT	Includes 65+		Care dependent	The Americas	High‐income economies	Homemaking		
Thomas (2018)										
	Complete	RCT	Includes 65+		Physical frailty	The Americas	High‐income economies	Homemaking		
Thygesen (2015)										
	Complete	RCT	Includes 65+	25%‐50% female included	Discharged from hospital	European	High‐income economies			General health services for disease prevention
										Visiting health professionals
Tibaldi (2004)										
	Complete	RCT	Includes 75+	50%‐75% female included	Noncommunicable disease	European	High‐income economies			General health services for disease prevention
					Physical frailty					Visiting health professionals
Tibaldi (2009)										
	Complete	RCT	Includes 65+	50%‐75% female included	Noncommunicable disease	European	High‐income economies			General health services for disease prevention
										Visiting health professionals
Tinetti (1999)										
	Complete	RCT	Includes 65+	75%‐100% female included	Discharged from hospital	The Americas	High‐income economies			Rehabilitation services
					Noncommunicable disease					Visiting health professionals
Toots (2017)										
	Complete	RCT	Includes 65+	75%‐100% female included	Dementia	European	High‐income economies			Rehabilitation services
					Noncommunicable disease					Visiting health professionals
Townsend (1988)										
	Complete	RCT	Includes 75+	50%‐75% female included	Discharged from hospital	European	High‐income economies			General health services for disease prevention
										Visiting health professionals
Tsaih (2011)										
	Complete	RCT	Includes 65+	25%‐50% female included	Injury	Western Pacific	High‐income economies			Rehabilitation services
										Visiting health professionals
Tseng (2016)										
	Complete	RCT	Includes 65+	50%‐75% female included	Injury	Western Pacific	High‐income economies			General health services for disease prevention
										Rehabilitation services
										Visiting health professionals
Tsuchihashi‐Makaya (2013)										
	Complete	RCT	Includes 65+	25%‐50% female included	Noncommunicable disease	Western Pacific	High‐income economies			General health services for disease prevention
										Visiting health professionals
Turunen (2017)										
	On‐going	RCT	Includes 65+		Discharged from hospital	European	High‐income economies			Rehabilitation services
										Visiting health professionals
Underwood (2013)										
	Complete	RCT	Includes 65+	50%‐75% female included	Noncommunicable disease	European	High‐income economies			Rehabilitation services
										Visiting health professionals
Valdes (2015)										
	Complete	RCT	Includes <65	75%‐100% female included	Injury	The Americas	High‐income economies			Rehabilitation services
			Includes 65+							Visiting health professionals
Van Der Pols‐Vijlbrief (2016)										
	Complete	RCT	Includes 65+	50%‐75% female included	Noncommunicable disease	European	High‐income economies			General health services for disease prevention
										Visiting health professionals
van Haastregt (2000)										
	Complete	RCT	Includes 65+	50%‐75% female included	Injury	European	High‐income economies			General health services for disease prevention
										Visiting health professionals
van Hout (2010)										
	Complete	RCT	Includes 75+	50%‐75% female included	Noncommunicable disease	European	High‐income economies			General health services for disease prevention
										Visiting health professionals
van Houten (2007)										
	Complete	RCT	Includes 65+	75%‐100% female included	Noncommunicable disease	European	High‐income economies			Rehabilitation services
										Visiting health professionals
Vass (2007)										
	Complete	RCT	Includes 75+	50%‐75% female included		European	High‐income economies			General health services for disease prevention
										Visiting health professionals
Verweij (2018)										
	On‐going	RCT	Includes 65+		Discharged from hospital	European	High‐income economies			General health services for disease prevention
					Noncommunicable disease					Rehabilitation services
										Visiting health professionals
Vogler (2009)										
	Complete	RCT	Includes 75+	50%‐75% female included	Discharged from hospital	Western Pacific	High‐income economies			Rehabilitation services
				75%‐100% female included						Visiting health professionals
Weir (1998)										
	Complete	RCT	Includes <65	50%‐75% female included	Discharged from hospital	The Americas	High‐income economies			General health services for disease prevention
			Includes 65+							Rehabilitation services
										Visiting health professionals
Whitehead (2014)										
	On‐going	RCT	Includes <65			European	High‐income economies			Rehabilitation services
			Includes 65+							Visiting health professionals
Wilhelmson (2013)										
	Complete	RCT	Includes 75+	50%‐75% female included	Physical frailty	European	High‐income economies			Health promotion services
										Visiting health professionals
Wilson (2009)										
	Complete	RCT	Includes <65	75%‐100% female included	Noncommunicable disease	The Americas	High‐income economies			Rehabilitation services
			Includes 65+							Visiting health professionals
Wishart (2000)										
	Complete	RCT	Includes 75+	75%‐100% female included	Noncommunicable disease	The Americas	High‐income economies			Rehabilitation services
										Visiting lay service providers
Wisniowska‐Szurlej (2017)										
	On‐going	RCT	Includes 65+		Care dependent	European	High‐income economies			Rehabilitation services
Wong (2015)										
	Complete	RCT	Includes <65	50%‐75% female included	Discharged from hospital	Western Pacific	High‐income economies			General health services for disease prevention
			Includes 65+							Visiting health professionals
Wong (2016)										
	Complete	RCT	Includes 75+	50%‐75% female included	Discharged from hospital	Western Pacific	High‐income economies			Long term care services
					Noncommunicable disease					Visiting health professionals
										Visiting lay service providers
Wylie (2017)										
	Complete	RCT	Includes 65+	75%‐100% female included	Injury	European	High‐income economies	Personal care		Rehabilitation services
Young (1992)										
	Complete	RCT	Includes <65	25%‐50% female included	Discharged from hospital	The Americas	High‐income economies			Rehabilitation services
			Includes 65+		Noncommunicable disease					Visiting health professionals
Ziden (2008)										
	Complete	RCT	Includes 75+	50%‐75% female included	Care dependent	European	High‐income economies			Rehabilitation services
					Injury					Visiting health professionals
Ziden (2010)										
	Complete	RCT	Includes 65+	75%‐100% female included	Injury	European	High‐income economies			Rehabilitation services
										Visiting health professionals
Ziden (2014)										
	Complete	RCT	Includes 85+	50%‐75% female included	Injury	European	High‐income economies			General health services for disease prevention
										Rehabilitation services
										Visiting health professionals
Zimmer (1985)										
	Complete	RCT	Includes 65+	50%‐75% female included	Care dependent	The Americas	High‐income economies			General health services for disease prevention
					Noncommunicable disease					Visiting health professionals
					Physical frailty					Visiting lay service providers
Abdulla (2013)										
	Complete	Systematic review	Includes 65+		Noncommunicable disease	European	High‐income economies			Rehabilitation services
					Physical frailty					Visiting health professionals
Allen et al. (2014)										
	Complete	Systematic review	Includes <65		Discharged from hospital	Western Pacific	High‐income economies			General health services for disease prevention
			Includes 65+							Visiting health professionals
Andy (2016)										
	Complete	Systematic review	Includes 65+	50%‐75% female included	Care dependent	European	High‐income economies			Rehabilitation services
										Visiting health professionals
Apostolo (2018)										
	Complete	Systematic review	Includes 65+		Physical frailty	European	High‐income economies			General health services for disease prevention
										Rehabilitation services
						Visiting health professionals
Baldwin (2011)										
	Complete	Systematic review	Includes <65		Physical frailty	European	High‐income economies			Health promotion services
			Includes 65+							Visiting health professionals
Baxter (2016)										
	Complete	Systematic review	Includes <65			European	High‐income economies			Rehabilitation services
			Includes 65+							Visiting health professionals
Berger (2013)										
	Complete	Systematic review			Noncommunicable disease	The Americas	High‐income economies			Rehabilitation services
										Visiting health professionals
Beswick (2010)										
	Complete	Systematic review	Includes 65+		Discharged from hospital	European	High‐income economies			General health services for disease prevention
					Injury					Visiting health professionals
			Physical frailty			
Blythe (2009)										
	Complete	Systematic review	Includes <65		Care dependent	Western Pacific	High‐income economies			General health services for disease prevention
			Includes 65+		Dementia					Visiting health professionals
					Noncommunicable disease					
Bryant‐Lukosius (2015)										
	Complete	Systematic review	Includes <65		Communicable disease	The Americas	High‐income economies			General health services for disease prevention
			Includes 65+		Discharged from hospital					Visiting health professionals
Bula (2011)										
	Complete	Systematic review	Includes <65		Injury	European	High‐income economies			Rehabilitation services
			Includes 65+							Visiting health professionals
Bunn (2016)										
	Complete	Systematic review	Includes <65		Dementia	European	High‐income economies	Homemaking		
			Includes 65+		Noncommunicable disease					
Burns (2001)										
	Complete	Systematic review	Includes <65		Noncommunicable disease	European	High‐income economies			General health services for disease prevention
			Includes 65+							Visiting health professionals
Burton (2015)										
	Complete	Systematic review	Includes 65+		Dementia	Western Pacific	High‐income economies			Rehabilitation services
					Noncommunicable disease					Visiting health professionals
Burton (2015)										
	Complete	Systematic review	Includes 65+	75%‐100% female included	Physical frailty	Western Pacific	High‐income economies			Rehabilitation services
										Visiting health professionals
Cadore (2013)										
	Complete	Systematic review	Includes 65+		Physical frailty	European	High‐income economies			Rehabilitation services
										Visiting health professionals
Candy (2011)										
	Complete	Systematic review			End‐of‐life	European	High‐income economies			Long term care services
										Visiting health professionals
Cattan (2005)										
	Complete	Systematic review	Includes <65		Social isolation	European	High‐income economies	Family and caregiver support		Health promotion services
			Includes 65+							Visiting health professionals
Chiung‐Ju (2013)										
	Complete	Systematic review	Includes 65+		Noncommunicable disease	The Americas	High‐income economies			Rehabilitation services
										Visiting health professionals
Chou (2012)										
	Complete	Systematic review	Includes 75+	50%‐75% female included	Care dependent	Western Pacific	Upper‐middle‐income economies			Rehabilitation services
					Physical frailty					Visiting health professionals
Clarkson (2018)										
	Complete	Systematic review	Includes <65		Dementia	European	High‐income economies	Family and caregiver support		General health services for disease prevention
			Includes 65+		Noncommunicable disease					Visiting health professionals
Clegg (2012)										
	Complete	Systematic review	Includes 65+		Physical frailty	European	High‐income economies			Rehabilitation services
			Includes 75+							Visiting health professionals
Cobban (2012)										
	Complete	Systematic review	Includes 65+		Noncommunicable disease	The Americas	High‐income economies			General health services for disease prevention
										Rehabilitation services
										Visiting health professionals
Cochrane (2014)										
	Complete	Systematic review	Includes 65+	50%‐75% female included	Care dependent	European	High‐income economies			Rehabilitation services
										Visiting health professionals
Corrieri (2011)										
	Complete	Systematic review	Includes 65+			European	High‐income economies			General health services for disease prevention
										Health promotion services
										Visiting health professionals
Crocker (2013)										
	Complete	Systematic review	Includes <65		Care dependent	European	High‐income economies			Rehabilitation services
			Includes 65+		Noncommunicable disease					Visiting health professionals
Daniels (2008)										
	Complete	Systematic review	Includes 75+		Physical frailty	European	High‐income economies			Rehabilitation services
										Visiting health professionals
Davis (2015)										
	Complete	Systematic review	Includes <65		End‐of‐life	The Americas	High‐income economies			Long term care services
			Includes 65+							Visiting health professionals
De Coninck (2017)										
	Complete	Systematic review	Includes <65		Injury	European	High‐income economies			Rehabilitation services
			Includes 65+		Noncommunicable disease					Long term care services
de Vries (2012)										
	Complete	Systematic review	Includes 65+		Physical frailty	European	High‐income economies			Rehabilitation services
										Visiting health professionals
Desheng (2018)										
	Complete	Systematic review	Includes 65+		Injury	Western Pacific	Upper‐middle‐income economies			Rehabilitation services
										Visiting health professionals
Dickens (2011)										
	Complete	Systematic review	Includes <65		Social isolation	European	High‐income economies			General health services for disease prevention
			Includes 65+							Visiting health professionals
						
Eklund (2009)										
	Complete	Systematic review	Includes 65+		Physical frailty	European	High‐income economies			General health services for disease prevention
										Visiting health professionals
Elkan (2001)										
	Complete	Systematic review	Includes 65+		Physical frailty	European	High‐income economies	Family and caregiver support		General health services for disease prevention
										Health promotion services
										Visiting health professionals
Evans (2003)										
	Complete	Systematic review	Includes 65+		Noncommunicable disease	European	High‐income economies			Rehabilitation services
										Visiting health professionals
Fletcher‐Smith (2013)										
	Complete	Systematic review	Includes <65		Noncommunicable disease	European	High‐income economies			Rehabilitation services
			Includes 65+							Visiting health professionals
Fomiatti (2013)										
	Complete	Systematic review	Includes 65+		Noncommunicable disease	Western Pacific	High‐income economies		Personal mobility and transportation devices	
Forbes (2015)										
	Complete	Systematic review	Includes 65+		Dementia	The Americas	High‐income economies			Rehabilitation services
					Noncommunicable disease					Visiting health professionals
Franck (2016)										
	Complete	Systematic review	Includes <65		Social isolation	Western Pacific	High‐income economies			General health services for disease prevention
			Includes 65+							Rehabilitation services
										Visiting health professionals
Gillespie (2012)										
	Complete	Systematic review	Includes <65	50%‐75% female included	Injury	European	High‐income economies		Personal mobility and transportation devices	Rehabilitation services
			Includes 65+							Visiting health professionals
Gine‐Garriga (2014)										
	Complete	Systematic review	Includes <65		Physical frailty	European	High‐income economies			Rehabilitation services
			Includes 65+							Visiting health professionals
Golding‐Day (2017)										
	Complete	Systematic review	Includes <65		Physical frailty	European	High‐income economies	Personal care		Visiting health professionals
			Includes 65+							
Gomes (2013)										
	Complete	Systematic review	Includes <65		End‐of‐life	European	High‐income economies			Long term care services
			Includes 65+							Visiting health professionals
Grant (2014)										
	Complete	Systematic review	Includes 65+	50%‐75% female included	Noncommunicable disease	European	High‐income economies			General health services for disease prevention
										Visiting health professionals
Graybill (2014)										
	Complete	Systematic review	Includes 65+		Noncommunicable disease	European	High‐income economies			
					Physical frailty					
Hall (2011)										
	Complete	Systematic review	Includes 75+	75%‐100% female included	End‐of‐life	European	High‐income economies			Long term care services
					Noncommunicable disease					Visiting health professionals
Handoll (2009)										
	Complete	Systematic review	Includes 65+		Injury	European	High‐income economies			Rehabilitation services
					Noncommunicable disease					Visiting health professionals
Handoll (2015)										
	Complete	Systematic review	Includes <65	50%‐75% female included	Injury	European	High‐income economies			Rehabilitation services
			Includes 65+							Visiting health professionals
Hill (2015)										
	Complete	Systematic review	Includes <65		Injury	Western Pacific	High‐income economies			Rehabilitation services
			Includes 65+							Visiting health professionals
Hobbs (2013)										
	Complete	Systematic review	Includes <65		Noncommunicable disease	European	High‐income economies			Rehabilitation services
			Includes 65+							Visiting health professionals
Howe (2011)										
	Complete	Systematic review	Includes <65		Physical frailty	European	High‐income economies			Rehabilitation services
			Includes 65+							Visiting health professionals
Hunter (2018)										
	Complete	Systematic review	Includes 65+		Noncommunicable disease	The Americas	High‐income economies			Rehabilitation services
										Visiting health professionals
Huss (2008)										
	Complete	Systematic review	Includes 65+			European	High‐income economies			General health services for disease prevention
										Visiting health professionals
Jane (2017)										
	Complete	Systematic review	Includes 65+		Care dependent	European	High‐income economies			
					Noncommunicable disease					
Kang‐Yi (2010)										
	Complete	Systematic review	Includes 65+		Care dependent	The Americas	High‐income economies			Rehabilitation services
					Noncommunicable disease					Visiting health professionals
Konno (2011)										
	On‐going	Systematic review	Includes <65		Dementia	Western Pacific	High‐income economies	Personal care		Visiting health professionals
			Includes 65+		Noncommunicable disease					
Konno (2013)										
	Complete	Systematic review			Dementia	Western Pacific	High‐income economies	Personal care		Visiting health professionals
					Noncommunicable disease					
Konno (2014)										
	Complete	Systematic review	Includes <65		Care dependent	Western Pacific	High‐income economies	Personal care		
			Includes 65+		Dementia					
			Noncommunicable disease			
Kurz (2011)										
	Complete	Systematic review	Includes <65		Noncommunicable disease	European	High‐income economies			General health services for disease prevention
			Includes 65+							Rehabilitation services
										Visiting health professionals
Lacroix (2017)										
	Complete	Systematic review	Includes 65+			European	High‐income economies			Rehabilitation services
										Visiting health professionals
Legg (2004)										
	Complete	Systematic review	Includes 65+		Noncommunicable disease	European	High‐income economies			Rehabilitation services
Legg (2017)										
	Complete	Systematic review	Includes <65		Noncommunicable disease	European	High‐income economies			Rehabilitation services
			Includes 65+							Visiting health professionals
Lewis (2017)										
	Complete	Systematic review	Includes 65+		Noncommunicable disease	Western Pacific	High‐income economies			Rehabilitation services
										Visiting health professionals
Liimatta (2016)										
	Complete	Systematic review	Includes 65+	50%‐75% female included	Physical frailty	European	High‐income economies			General health services for disease prevention
										Visiting health professionals
Liu (2015)										
	Complete	Systematic review	Includes 65+		Dementia	The Americas	High‐income economies	Personal care		
					Noncommunicable disease					
Liu (2018)										
	Complete	Systematic review	Includes 65+		Care dependent	The Americas	High‐income economies			Rehabilitation services
					Discharged from hospital					Visiting health professionals
			Injury			
			Physical frailty			
Low (2011)										
	Complete	Systematic review	Includes 65+		Noncommunicable disease	Western Pacific	High‐income economies			General health services for disease prevention
										Visiting health professionals
Martin (2011)										
	Complete	Systematic review	Includes 65+		Noncommunicable disease	European	High‐income economies			General health services for disease prevention
										Rehabilitation services
						Visiting health professionals
Mayo‐Wilson (2014)										
	Complete	Systematic review	Includes 65+			European	High‐income economies			General health services for disease prevention
										Visiting health professionals
McClure (2005)										
	Complete	Systematic review	Includes 65+		Injury	Western Pacific	High‐income economies			General health services for disease prevention
										Visiting health professionals
McWilliam (2000)										
	Complete	Systematic review	Includes 65+			The Americas	High‐income economies		Personal mobility and transportation devices	General health services for disease prevention
										Health promotion services
										Rehabilitation services
										Visiting health professionals
Meinck (2004)										
	Complete	Systematic review	Includes <65			European	High‐income economies			General health services for disease prevention
			Includes 65+							Visiting health professionals
Montgomery (2008)										
	Complete	Systematic review	Includes 65+		Care dependent	European	High‐income economies	Personal care		
Munk (2016)										
	Complete	Systematic review	Includes <65		Physical frailty	European	High‐income economies			General health services for disease prevention
			Includes 65+							Visiting health professionals
Oliver (2007)										
	Complete	Systematic review			Injury	European	High‐income economies			General health services for disease prevention
					Noncommunicable disease					Visiting health professionals
Outpatient (2003)										
	Complete	Systematic review	Includes <65	50%‐75% female included	Noncommunicable disease	European	High‐income economies			Rehabilitation services
			Includes 65+							Visiting health professionals
Ozdemir (2017)										
	Complete	Systematic review	Includes <65		Discharged from hospital	European	Lower‐middle‐income economies			Rehabilitation services
			Includes 65+							Visiting health professionals
Patterson (1999)										
	Complete	Systematic review	Includes 65+		Discharged from hospital	European	High‐income economies			General health services for disease prevention
										Visiting health professionals
Pitkala (2013)										
	Complete	Systematic review	Includes 75+	50%‐75% female included	Dementia	European	High‐income economies			Rehabilitation services
					Noncommunicable disease					Visiting health professionals
Poscia (2018)										
	Complete	Systematic review	Includes 65+	50%‐75% female included	Social isolation	European	High‐income economies			General health services for disease prevention
										Visiting health professionals
Potter (2011)										
	Complete	Systematic review	Includes <65		Dementia	European	High‐income economies			Rehabilitation services
			Includes 65+		Noncommunicable disease					
Reilly (2015)										
	Complete	Systematic review	Includes <65		Dementia	European	High‐income economies			General health services for disease prevention
			Includes 65+		Noncommunicable disease					Visiting health professionals
Renz (2017)										
	Complete	Systematic review	Includes <65			European	High‐income economies			General health services for disease prevention
			Includes 65+							Visiting health professionals
Resnick (2016)										
	Complete	Systematic review	Includes 65+		Injury	The Americas	High‐income economies			Rehabilitation services
					Noncommunicable disease					Visiting health professionals
Roe (2015)										
	Complete	Systematic review	Includes <65		Noncommunicable disease	European	High‐income economies	Personal care		Health promotion services
			Includes 65+							Visiting health professionals
Roets‐Merken (2015)										
	Complete	Systematic review	Includes <65		Care dependent	European	High‐income economies			Rehabilitation services
			Includes 65+		Noncommunicable disease					Visiting health professionals
Santomassino (2012)										
	Complete	Systematic review	Includes <65		Noncommunicable disease	The Americas	High‐income economies			General health services for disease prevention
			Includes 65+							Visiting health professionals
Sean (2014)										
	Complete	Systematic review	Includes 65+	50%‐75% female included	Noncommunicable disease	European	High‐income economies			General health services for disease prevention
										Visiting health professionals
Shaw (2009)										
	Complete	Systematic review	Includes 65+	50%‐75% female included	Physical frailty	European	High‐income economies	Family and caregiver support		
Shepperd (2005)										
	Complete	Systematic review	Includes 65+		Discharged from hospital	European	High‐income economies			General health services for disease prevention
										Visiting health professionals
Shepperd (2011)										
	Complete	Systematic review	Includes <65		End‐of‐life	European	High‐income economies			Long term care services
			Includes 65+							Visiting health professionals
Shepperd (2016)										
	Complete	Systematic review	Includes <65			European	High‐income economies			General health services for disease prevention
			Includes 65+							Visiting health professionals
Shvedko (2018)										
	Complete	Systematic review	Includes <65	50%‐75% female included	Noncommunicable disease	European	High‐income economies			Rehabilitation services
			Includes 65+							Visiting health professionals
Simek (2012)										
	Complete	Systematic review	Includes <65		Injury	Western Pacific	High‐income economies			Rehabilitation services
			Includes 65+							Visiting health professionals
Sims‐Gould (2017)										
	Complete	Systematic review	Includes 65+	50%‐75% female included	Care dependent	The Americas	High‐income economies			Rehabilitation services
					Noncommunicable disease					Visiting health professionals
					Physical frailty					
Skelton (2013)										
	Complete	Systematic review	Includes <65		Noncommunicable disease	European	High‐income economies			
			Includes 65+							
Smeeth (2006)										
	Complete	Systematic review	Includes 65+			European	High‐income economies			General health services for disease prevention
										Visiting health professionals
Smith (2016)										
	Complete	Systematic review	Includes <65		Noncommunicable disease	European	High‐income economies			General health services for disease prevention
		Includes 65+				
Stall (2014)										
	Complete	Systematic review	Includes 65+		Care dependent	The Americas	High‐income economies			General health services for disease prevention
										Visiting health professionals
Steultjens (2004)										
	Complete	Systematic review	Includes 65+		Noncommunicable disease	European	High‐income economies			Rehabilitation services
										Visiting health professionals
Steultjens (2004)										
	Complete	Systematic review	Includes <65			European	High‐income economies			Rehabilitation services
			Includes 65+							Visiting health professionals
Stolee (2012)										
	Complete	Systematic review	Includes <65		Noncommunicable disease	The Americas	High‐income economies			Rehabilitation services
			Includes 65+							Visiting health professionals
Stuck (2002)										
	Complete	Systematic review	Includes 65+			European	High‐income economies			General health services for disease prevention
										Visiting health professionals
Talley (2011)										
	Complete	Systematic review	Includes 65+		Noncommunicable disease	The Americas	High‐income economies			General health services for disease prevention
										Visiting health professionals
Tappenden (2012)										
	Complete	Systematic review	Includes 75+		Physical frailty	European	High‐income economies			Health promotion services
										Visiting health professionals
Therapy‐based rehabilitation (2003)										
	Complete	Systematic review	Includes 65+		Noncommunicable disease	European	High‐income economies			Rehabilitation services
										Visiting health professionals
Thiebaud (2014)										
	Complete	Systematic review	Includes 75+	50%‐75% female included		The Americas	High‐income economies			Rehabilitation services
										Visiting health professionals
Toles (2016)										
	Complete	Systematic review	Includes 75+	50%‐75% female included	Discharged from hospital	The Americas	High‐income economies			General health services for disease prevention
										Visiting health professionals
Tseng (2011)										
	Complete	Systematic review	Includes 65+		Noncommunicable disease	Western Pacific	High‐income economies			Rehabilitation services
										Visiting health professionals
Vaapio (2009)										
	Complete	Systematic review	Includes <65		Injury	European	High‐income economies			Rehabilitation services
			Includes 65+							Visiting health professionals
van Abbema (2015)										
	Complete	Systematic review	Includes 65+		Physical frailty	European	High‐income economies			Rehabilitation services
										Visiting health professionals
Van Citters (2004)										
	Complete	Systematic review	Includes 65+		Noncommunicable disease	The Americas	High‐income economies			General health services for disease prevention
										Visiting health professionals
Ward (2003)										
	Complete	Systematic review	Includes <65		Care dependent	European	High‐income economies			Rehabilitation services
			Includes 65+		Noncommunicable disease					Visiting health professionals
Watanabe (2015)										
	On‐going	Systematic review	Includes <65		Noncommunicable disease	Western Pacific	High‐income economies		Personal mobility and transportation devices	
			Includes 65+							
Weber (2018)										
	Complete	Systematic review	Includes <65	50%‐75% female included	Care dependent	European	High‐income economies			Rehabilitation services
			Includes 65+		Injury					Visiting health professionals
Winkel (2008)										
	Complete	Systematic review	Includes <65		Noncommunicable disease	European	High‐income economies			General health services for disease prevention
			Includes 65+							Rehabilitation services
Yi (2015)										
	On‐going	Systematic review	Includes <65		Dementia	Western Pacific	High‐income economies	Personal care		General health services for disease prevention
			Includes 65+		Noncommunicable disease					Visiting health professionals
Young (2017)										
	Complete	Systematic review	Includes 65+		Care dependent	European	High‐income economies			Long term care services
					Noncommunicable disease					Visiting health professionals
Zhu (2013)										
	Complete	Systematic review	Includes <65		Care dependent	The Americas	High‐income economies	Personal care		
			Includes 65+		Noncommunicable disease					
Zubala (2017)										
	Complete	Systematic review	Includes <65	50%‐75% female included		European	High‐income economies			Rehabilitation services
			Includes 65+							Visiting health professionals

**Table:** 

Study	Intervention: design, construction and building products and technology of buildings for private use	Outcomes: intrinsic capacity	Outcomes: functional ability	Outcomes: process and other outcomes	Setting	Comparison	PROGRESS factors	Gender inequalities	Other health inequalities	Study quality
Acton (2016)										
		Mental functions	Basic needs		Residential home/apartment	Usual care		No ‐ assessment of effects by sex/gender NOT present	No ‐ assessment of effects across any other PROGRESS characteristics? For example, socioeconomic status	RCT
		Sensory functions and pain	Quality of life							
Aimonino (2008)										
		Mental functions	Basic needs	Cost (e.g.,out of pocket)	Residential home/apartment	Usual care		No ‐ assessment of effects by sex/gender NOT present	No ‐ assessment of effects across any other PROGRESS characteristics? For example, socioeconomic status	RCT
		Functions of the digestive, metabolic and endocrine systems	Quality of life	Satisfaction of older adult						
			Mobility	Caregiver outcomes						
				Health service utilization						
Alexander (2001)										
			Mobility		Assisted living	Other		No ‐ assessment of effects by sex/gender NOT present	No ‐ assessment of effects across any other PROGRESS characteristics? For example, socioeconomic status	RCT
Alexopoulos (2016)										
		Mental functions	Learning, grow and make decisions		Residential home/apartment	Other	Socioeconomic status	No ‐ assessment of effects by sex/gender NOT present	No ‐ assessment of effects across any other PROGRESS characteristics? For example, socioeconomic status	RCT
Amjad (2018)										
			Quality of life	Cost (e.g., out of pocket)	Residential home/apartment	Usual care		No ‐ assessment of effects by sex/gender NOT present	No ‐ assessment of effects across any other PROGRESS characteristics? For example, socioeconomic status	RCT
				Health service utilization	Independent living					
Andersen (2000)										
		Mental functions	Basic needs	Health service utilization	Residential home/apartment	Usual care		No ‐ assessment of effects by sex/gender NOT present	No ‐ assessment of effects across any other PROGRESS characteristics? For example, socioeconomic status	RCT
		Neuromusculoskeletal function	Mobility							
Anonymous (2004)										
		Mental functions	Quality of life		Residential home/apartment	Usual care	Social capital	No ‐ assessment of effects by sex/gender NOT present	No ‐ assessment of effects across any other PROGRESS characteristics? For example, socioeconomic status	RCT
Araujo (2015)										
			Basic needs	Caregiver outcomes	Residential home/apartment	Usual care		No ‐ assessment of effects by sex/gender NOT present	No ‐ assessment of effects across any other PROGRESS characteristics? For example, socioeconomic status	RCT
			Quality of life	Health service utilization						
			Mobility							
Arean (2015)										
		Mental functions	Basic needs		Residential home/apartment	Other	Socioeconomic status	No ‐ assessment of effects by sex/gender NOT present	Yes ‐ assessment of effects across any other PROGRESS characteristics? For example, socioeconomic status	RCT
			Mobility							
Arrieta (2018)										
		Mental functions	Basic needs		Assisted living	Usual care		No ‐ assessment of effects by sex/gender NOT present	No ‐ assessment of effects across any other PROGRESS characteristics? For example, socioeconomic status	RCT
		Neuromusculoskeletal function	Quality of life							
			Mobility							
			Contribution							
			Financial security and stability							
Ashburn (2007)										
			Basic needs	Falls	Residential home/apartment	Usual care		No ‐ assessment of effects by sex/gender NOT present	No ‐ assessment of effects across any other PROGRESS characteristics? For example, socioeconomic status	RCT
			Mobility							
Avlund (2002)										
			Basic needs		Residential home/apartment	Usual care		No ‐ assessment of effects by sex/gender NOT present	No ‐ assessment of effects across any other PROGRESS characteristics? For example, socioeconomic status	RCT
			Mobility							
Baker (2007)										
		Neuromusculoskeletal function	Mobility		Independent living	Usual care		No ‐ assessment of effects by sex/gender NOT present	No ‐ assessment of effects across any other PROGRESS characteristics? For example, socioeconomic status	RCT
Banerjee (1996)										
		Mental functions			Residential home/apartment	Usual care		No ‐ assessment of effects by sex/gender NOT present	No ‐ assessment of effects across any other PROGRESS characteristics? For example, socioeconomic status	RCT
Barnes (2017)										
			Basic needs		Residential home/apartment	Other		No ‐ assessment of effects by sex/gender NOT present	No ‐ assessment of effects across any other PROGRESS characteristics? For example, socioeconomic status	RCT
			Quality of life							
			Mobility					
Barreto (2018)										
		Neuromusculoskeletal function	Mobility	Adherence	Residential home/apartment	Usual care		No ‐ assessment of effects by sex/gender NOT present	No ‐ assessment of effects across any other PROGRESS characteristics? For example, socioeconomic status	RCT
Batchelor‐Murphy (2017)										
		Functions of the digestive, metabolic and endocrine systems	Basic needs		Assisted living	Other		No ‐ assessment of effects by sex/gender NOT present	No ‐ assessment of effects across any other PROGRESS characteristics? For example, socioeconomic status	RCT
Beck (2013)										
		Mental functions	Basic needs	Health service utilization	Residential home/apartment	Usual care		No ‐ assessment of effects by sex/gender NOT present	No ‐ assessment of effects across any other PROGRESS characteristics? For example, socioeconomic status	RCT
		Neuromusculoskeletal function	Mobility							
			Contribution							
Beck (2016)										
		Functions of the digestive, metabolic and endocrine systems	Basic needs	Health service utilization	Residential home/apartment	Usual care		No ‐ assessment of effects by sex/gender NOT present	No ‐ assessment of effects across any other PROGRESS characteristics? For example, socioeconomic status	RCT
		Neuromusculoskeletal function	Quality of life		Assisted living					
			Mobility							
Behm (2014)										
		Mental functions	Quality of life	Satisfaction of older adult	Residential home/apartment	Other		No ‐ assessment of effects by sex/gender NOT present	No ‐ assessment of effects across any other PROGRESS characteristics? For example, socioeconomic status	RCT
		Sensory functions and pain								
		Neuromusculoskeletal function								
Behm (2016)										
		Mental functions			Residential home/apartment	Usual care		No ‐ assessment of effects by sex/gender NOT present	No ‐ assessment of effects across any other PROGRESS characteristics? For example, socioeconomic status	RCT
		Sensory functions and pain								
		Neuromusculoskeletal function								
Beland (2006)										
		Mental functions	Basic needs	Cost (e.g., out of pocket)	Residential home/apartment	Usual care		No ‐ assessment of effects by sex/gender NOT present	No ‐ assessment of effects across any other PROGRESS characteristics? For example, socioeconomic status	RCT
			Mobility	Satisfaction of older adult						
				Cost‐effectiveness						
				Health service utilization						
Bennell (2018)										
		Mental functions	Basic needs		Residential home/apartment	Usual care		No ‐ assessment of effects by sex/gender NOT present	No ‐ assessment of effects across any other PROGRESS characteristics? For example, socioeconomic status	RCT
		Sensory functions and pain								
Bernabei (1998)										
			Basic needs	Cost‐effectiveness	Residential home/apartment	Usual care		No ‐ assessment of effects by sex/gender NOT present	No ‐ assessment of effects across any other PROGRESS characteristics? For example, socioeconomic status	RCT
			Mobility	Health service utilization						
Bjerk (2017)										
		Neuromusculoskeletal function	Quality of life	Adherence	Residential home/apartment	Usual care		No ‐ assessment of effects by sex/gender NOT present	No ‐ assessment of effects across any other PROGRESS characteristics? For example, socioeconomic status	RCT
			Mobility	Falls						
Blanchard (1999)										
		Mental functions			Residential home/apartment	Usual care		No ‐ assessment of effects by sex/gender NOT present	No ‐ assessment of effects across any other PROGRESS characteristics? For example, socioeconomic status	RCT
Bleijenberg (2016)										
			Basic needs	Satisfaction of older adult	Residential home/apartment	Other		No ‐ assessment of effects by sex/gender NOT present	No ‐ assessment of effects across any other PROGRESS characteristics? For example, socioeconomic status	RCT
			Quality of life	Caregiver outcomes						
				Health service utilization						
Bonnefoy (2012)										
		Functions of the digestive, metabolic and endocrine systems	Basic needs	Adherence	Residential home/apartment	Other		No ‐ assessment of effects by sex/gender NOT present	No ‐ assessment of effects across any other PROGRESS characteristics? For example, socioeconomic status	RCT
		Neuromusculoskeletal function	Mobility	Safety						
Boongird (2017)										
		Mental functions	Quality of life	Adherence	Residential home/apartment	Usual care		No ‐ assessment of effects by sex/gender NOT present	No ‐ assessment of effects across any other PROGRESS characteristics? For example, socioeconomic status	RCT
		Neuromusculoskeletal function		Falls						
Bouman (2008)										
		Mental functions	Basic needs		Residential home/apartment	Usual care		No ‐ assessment of effects by sex/gender NOT present	No ‐ assessment of effects across any other PROGRESS characteristics? For example, socioeconomic status	RCT
			Quality of life							
Boxall (2005)										
		Mental functions	Quality of life	Health service utilization	Residential home/apartment	Usual care		No ‐ assessment of effects by sex/gender NOT present	No ‐ assessment of effects across any other PROGRESS characteristics? For example, socioeconomic status	RCT
		Functions of the cardiovascular, haematological, immunological and respiratory systems	Mobility							
		Neuromusculoskeletal function								
Brannstrom (2014)										
		Mental functions	Basic needs	Health service utilization	Residential home/apartment	Usual care		No ‐ assessment of effects by sex/gender NOT present	No ‐ assessment of effects across any other PROGRESS characteristics? For example, socioeconomic status	RCT
		Sensory functions and pain	Quality of life							
		Functions of the cardiovascular, haematological, immunological and respiratory systems	Mobility							
Brettschneider (2014)										
		Mental functions	Basic needs	Cost‐effectiveness	Residential home/apartment	Usual care		No ‐ assessment of effects by sex/gender NOT present	No ‐ assessment of effects across any other PROGRESS characteristics? For example, socioeconomic status	RCT
		Sensory functions and pain	Quality of life	Health service utilization						
Brovold (2012)										
		Mental functions	Quality of life		Residential home/apartment	Other		No ‐ assessment of effects by sex/gender NOT present	No ‐ assessment of effects across any other PROGRESS characteristics? For example, socioeconomic status	RCT
		Sensory functions and pain	Mobility							
		Neuromusculoskeletal function	Build and maintain relationships							
Bruce (2015)										
		Mental functions	Mobility		Assisted living	Usual care		No ‐ assessment of effects by sex/gender NOT present	No ‐ assessment of effects across any other PROGRESS characteristics? For example, socioeconomic status	RCT
Bruce (2016)										
				Health service utilization	Residential home/apartment	Usual care		No ‐ assessment of effects by sex/gender NOT present	No ‐ assessment of effects across any other PROGRESS characteristics? For example, socioeconomic status	RCT
Brumley (2007)										
				Cost (e.g., out of pocket)	Residential home/apartment	Usual care		No ‐ assessment of effects by sex/gender NOT present	No ‐ assessment of effects across any other PROGRESS characteristics? For example, socioeconomic status	RCT
				Satisfaction of older adult						
Burton (2013)										
		Mental functions	Basic needs	Falls	Residential home/apartment	Usual care		No ‐ assessment of effects by sex/gender NOT present	No ‐ assessment of effects across any other PROGRESS characteristics? For example, socioeconomic status	RCT
		Neuromusculoskeletal function	Mobility							
Buurman (2016)										
			Quality of life	Caregiver outcomes	Residential home/apartment	Usual care		No ‐ assessment of effects by sex/gender NOT present	No ‐ assessment of effects across any other PROGRESS characteristics? For example, socioeconomic status	RCT
			Mobility	Health service utilization	Assisted living					
Buys (2017)										
		Functions of the digestive, metabolic and endocrine systems	Basic needs	Satisfaction of older adult	Residential home/apartment	Usual care		No ‐ assessment of effects by sex/gender NOT present	No ‐ assessment of effects across any other PROGRESS characteristics? For example, socioeconomic status	RCT
				Access						
				Health service utilization						
Byles (2004)										
		Mental functions	Quality of life	Health service utilization	Residential home/apartment	Usual care		No ‐ assessment of effects by sex/gender NOT present	No ‐ assessment of effects across any other PROGRESS characteristics? For example, socioeconomic status	RCT
		Sensory functions and pain	Contribution							
		Neuromusculoskeletal function								
Byrnes (2015)										
			Quality of life	Cost (e.g., out of pocket)	Residential home/apartment	Usual care		No ‐ assessment of effects by sex/gender NOT present	No ‐ assessment of effects across any other PROGRESS characteristics? For example, socioeconomic status	RCT
				Cost‐effectiveness						
				Health service utilization						
Callahan (2012)										
		Neuromusculoskeletal function	Basic needs		Assisted living	Usual care		No ‐ assessment of effects by sex/gender NOT present	No ‐ assessment of effects across any other PROGRESS characteristics? For example, socioeconomic status	RCT
			Mobility							
Campbell (1997)										
		Neuromusculoskeletal function		Falls	Residential home/apartment	Other		No ‐ assessment of effects by sex/gender NOT present	No ‐ assessment of effects across any other PROGRESS characteristics? For example, socioeconomic status	RCT
Campbell (2005)										
	Adaptations to physical environment			Cost‐effectiveness	Residential home/apartment	Other		No ‐ assessment of effects by sex/gender NOT present	No ‐ assessment of effects across any other PROGRESS characteristics? For example, socioeconomic status	RCT
				Falls						
Canning (2015)										
		Neuromusculoskeletal function	Basic needs	Falls	Residential home/apartment	Usual care		No ‐ assessment of effects by sex/gender NOT present	No ‐ assessment of effects across any other PROGRESS characteristics? For example, socioeconomic status	RCT
Caplan (1999)										
		Mental functions		Satisfaction of older adult	Residential home/apartment	Usual care		No ‐ assessment of effects by sex/gender NOT present	No ‐ assessment of effects across any other PROGRESS characteristics? For example, socioeconomic status	RCT
		Functions of the digestive, metabolic and endocrine systems		Caregiver outcomes						
		Genitourinary and reproductive functions		Safety						
		Integumentary system		Health service utilization						
				Falls						
Caplan (2004)										
		Mental functions	Basic needs	Health service utilization	Residential home/apartment	Usual care		No ‐ assessment of effects by sex/gender NOT present	No ‐ assessment of effects across any other PROGRESS characteristics? For example, socioeconomic status	RCT
Caplan (2006)										
		Mental functions		Satisfaction of older adult	Residential home/apartment	Other		No ‐ assessment of effects by sex/gender NOT present	No ‐ assessment of effects across any other PROGRESS characteristics? For example, socioeconomic status	RCT
				Cost‐effectiveness						
				Health service utilization						
Carroll (2007)										
				Adherence	Residential home/apartment	Usual care		No ‐ assessment of effects by sex/gender NOT present	No ‐ assessment of effects across any other PROGRESS characteristics? For example, socioeconomic status	RCT
				Health service utilization						
Chaiyawat (2012)										
		Mental functions	Basic needs		Residential home/apartment	Usual care		No ‐ assessment of effects by sex/gender NOT present	No ‐ assessment of effects across any other PROGRESS characteristics? For example, socioeconomic status	RCT
			Quality of life							
Chan et al. (2016)										
			Learning, grow and make decisions		Residential home/apartment	Usual care		No ‐ assessment of effects by sex/gender NOT present	No ‐ assessment of effects across any other PROGRESS characteristics? For example, socioeconomic status	RCT
Chandler (1998)										
		Mental functions	Mobility	Falls	Residential home/apartment	Other		No ‐ assessment of effects by sex/gender NOT present	No ‐ assessment of effects across any other PROGRESS characteristics? For example, socioeconomic status	RCT
		Neuromusculoskeletal function								
Chang (2015)										
			Basic needs		Residential home/apartment	Other		No ‐ assessment of effects by sex/gender NOT present	No ‐ assessment of effects across any other PROGRESS characteristics? For example, socioeconomic status	RCT
			Mobility							
Chee (2013)										
	Adaptations to physical environment	Mental functions	Basic needs		Residential home/apartment	Other		No ‐ assessment of effects by sex/gender NOT present	No ‐ assessment of effects across any other PROGRESS characteristics? For example, socioeconomic status	RCT
Chen (2015)										
		Mental functions			Assisted living	Usual care		No ‐ assessment of effects by sex/gender NOT present	No ‐ assessment of effects across any other PROGRESS characteristics? For example, socioeconomic status	RCT
Chen (2015)										
		Neuromusculoskeletal function	Basic needs		Residential home/apartment	Usual care		No ‐ assessment of effects by sex/gender NOT present	No ‐ assessment of effects across any other PROGRESS characteristics? For example, socioeconomic status	RCT
			Mobility							
Chen (2016)										
		Functions of the cardiovascular, haematological, immunological and respiratory systems	Basic needs		Assisted living	Usual care		No ‐ assessment of effects by sex/gender NOT present	No ‐ assessment of effects across any other PROGRESS characteristics? For example, socioeconomic status	RCT
		Neuromusculoskeletal function	Mobility							
Cho (1998)										
			Basic needs		Residential home/apartment	Usual care		No ‐ assessment of effects by sex/gender NOT present	No ‐ assessment of effects across any other PROGRESS characteristics? For example, socioeconomic status	RCT
Chow (2014)										
		Mental functions	Basic needs	Health service utilization	Residential home/apartment	Usual care		No ‐ assessment of effects by sex/gender NOT present	No ‐ assessment of effects across any other PROGRESS characteristics? For example, socioeconomic status	RCT
		Sensory functions and pain	Quality of life							
Chu (2017)										
	Adaptations to physical environment	Mental functions	Basic needs	Health service utilization	Residential home/apartment	Other		No ‐ assessment of effects by sex/gender NOT present	No ‐ assessment of effects across any other PROGRESS characteristics? For example, socioeconomic status	RCT
				Falls						
Cichocki (2015)										
		Mental functions	Quality of life	Satisfaction of older adult	Assisted living	Usual care		No ‐ assessment of effects by sex/gender NOT present	No ‐ assessment of effects across any other PROGRESS characteristics? For example, socioeconomic status	RCT
		Sensory functions and pain	Mobility							
		Neuromusculoskeletal function	Build and maintain relationships							
Ciechanowski (2004)										
		Mental functions	Quality of life	Cost (e.g., out of pocket)	Residential home/apartment	Usual care		No ‐ assessment of effects by sex/gender NOT present	No ‐ assessment of effects across any other PROGRESS characteristics? For example, socioeconomic status	RCT
				Health service utilization						
Claffey (1976)										
				Cost‐effectiveness	Residential home/apartment	Usual care		No ‐ assessment of effects by sex/gender NOT present	No ‐ assessment of effects across any other PROGRESS characteristics? For example, socioeconomic status	RCT
Clegg (2014)										
		Mental functions	Basic needs		Residential home/apartment	Usual care		No ‐ assessment of effects by sex/gender NOT present	No ‐ assessment of effects across any other PROGRESS characteristics? For example, socioeconomic status	RCT
			Quality of life							
			Mobility							
Clemson (2016)										
			Basic needs	Health service utilization	Residential home/apartment	Usual care		No ‐ assessment of effects by sex/gender NOT present	No ‐ assessment of effects across any other PROGRESS characteristics? For example, socioeconomic status	RCT
Comans (2010)										
		Mental functions	Basic needs	Falls	Residential home/apartment	Usual care		No ‐ assessment of effects by sex/gender NOT present	No ‐ assessment of effects across any other PROGRESS characteristics? For example, socioeconomic status	RCT
		Sensory functions and pain	Quality of life							
		Functions of the digestive, metabolic and endocrine systems	Contribution							
		Genitourinary and reproductive functions								
		Neuromusculoskeletal function								
Conradsson (2010)										
		Mental functions			Assisted living	Other		No ‐ assessment of effects by sex/gender NOT present	No ‐ assessment of effects across any other PROGRESS characteristics? For example, socioeconomic status	RCT
Cornu (2003)										
			Basic needs	Adherence	Residential home/apartment	Usual care		No ‐ assessment of effects by sex/gender NOT present	No ‐ assessment of effects across any other PROGRESS characteristics? For example, socioeconomic status	RCT
				Health service utilization						
Corr (1995)										
		Mental functions	Basic needs	Health service utilization	Residential home/apartment	Usual care		No ‐ assessment of effects by sex/gender NOT present	No ‐ assessment of effects across any other PROGRESS characteristics? For example, socioeconomic status	RCT
Counsell (2007)										
		Mental functions	Basic needs	Health service utilization	Residential home/apartment	Usual care	Race, ethnicity, culture, language	No ‐ assessment of effects by sex/gender NOT present	No ‐ assessment of effects across any other PROGRESS characteristics? For example, socioeconomic status	RCT
		Sensory functions and pain					Socioeconomic status			
Courtney (2009)										
		Mental functions	Basic needs	Health service utilization	Residential home/apartment	Usual care		No ‐ assessment of effects by sex/gender NOT present	No ‐ assessment of effects across any other PROGRESS characteristics? For example, socioeconomic status	RCT
			Quality of life							
Courtney (2011)										
		Mental functions	Basic needs	Cost‐effectiveness	Residential home/apartment	Usual care		No ‐ assessment of effects by sex/gender NOT present	No ‐ assessment of effects across any other PROGRESS characteristics? For example, socioeconomic status	RCT
				Health service utilization						
Courtney (2012)										
			Basic needs	Health service utilization	Residential home/apartment	Usual care		No ‐ assessment of effects by sex/gender NOT present	No ‐ assessment of effects across any other PROGRESS characteristics? For example, socioeconomic status	RCT
			Mobility							
			Contribution							
Crotty (2002)										
		Mental functions	Quality of life	Satisfaction of older adult	Residential home/apartment	Usual care		No ‐ assessment of effects by sex/gender NOT present	No ‐ assessment of effects across any other PROGRESS characteristics? For example, socioeconomic status	RCT
				Caregiver outcomes						
				Health service utilization						
				Falls						
Crotty (2003)										
		Mental functions	Basic needs	Caregiver outcomes	Residential home/apartment	Usual care		No ‐ assessment of effects by sex/gender NOT present	No ‐ assessment of effects across any other PROGRESS characteristics? For example, socioeconomic status	RCT
			Quality of life							
			Mobility							
Crotty (2008)										
		Mental functions	Basic needs		Assisted living	Other		No ‐ assessment of effects by sex/gender NOT present	No ‐ assessment of effects across any other PROGRESS characteristics? For example, socioeconomic status	RCT
			Quality of life							
Cumming (2000)										
		Mental functions		Falls	Residential home/apartment	Other		No ‐ assessment of effects by sex/gender NOT present	No ‐ assessment of effects across any other PROGRESS characteristics? For example, socioeconomic status	RCT
Cummings (1990)										
		Mental functions	Basic needs	Satisfaction of older adult	Residential home/apartment	Other		No ‐ assessment of effects by sex/gender NOT present	No ‐ assessment of effects across any other PROGRESS characteristics? For example, socioeconomic status	RCT
				Cost‐effectiveness						
		Caregiver outcomes					
		Health service utilization					
Cunliffe (2004)										
		Mental functions	Basic needs		Residential home/apartment	Usual care		No ‐ assessment of effects by sex/gender NOT present	No ‐ assessment of effects across any other PROGRESS characteristics? For example, socioeconomic status	RCT
		Functions of the cardiovascular, haematological, immunological and respiratory systems			Communication					
		Functions of the digestive, metabolic and endocrine systems								
		Neuromusculoskeletal function								
Cutchin (2009)										
		Mental functions	Contribution	Satisfaction of older adult	Residential home/apartment	Other		No ‐ assessment of effects by sex/gender NOT present	No ‐ assessment of effects across any other PROGRESS characteristics? For example, socioeconomic status	RCT
				Neuromusculoskeletal function		Cost‐effectiveness				
				Health service utilization						
Dalby (2000)										
				Health service utilization	Residential home/apartment	Usual care		No ‐ assessment of effects by sex/gender NOT present	No ‐ assessment of effects across any other PROGRESS characteristics? For example, socioeconomic status	RCT
Daly (2015)										
		Mental functions	Basic needs	Adherence	Independent living	Usual care		No ‐ assessment of effects by sex/gender NOT present	No ‐ assessment of effects across any other PROGRESS characteristics? For example, socioeconomic status	RCT
		Functions of the digestive, metabolic and endocrine systems	Quality of life	Cost‐effectiveness						
		Neuromusculoskeletal function	Mobility	Safety						
		Falls					
Danilovich et al. (2017)										
		Mental functions	Basic needs	Satisfaction of older adult	Residential home/apartment	Usual care		No ‐ assessment of effects by sex/gender NOT present	No ‐ assessment of effects across any other PROGRESS characteristics? For example, socioeconomic status	RCT
			Quality of life	Caregiver outcomes						
			Contribution							
Dano (2016)										
		Mental functions	Quality of life		Residential home/apartment	Usual care		No ‐ assessment of effects by sex/gender NOT present	No ‐ assessment of effects across any other PROGRESS characteristics? For example, socioeconomic status	RCT
		Neuromusculoskeletal function								
Dechamps (2010)										
		Mental functions	Basic needs	Caregiver outcomes	Long‐term care	Usual care		No ‐ assessment of effects by sex/gender NOT present	No ‐ assessment of effects across any other PROGRESS characteristics? For example, socioeconomic status	RCT
					Assisted living					
Di Monaco (2008)										
			Basic needs	Falls	Residential home/apartment	Usual care		No ‐ assessment of effects by sex/gender NOT present	No ‐ assessment of effects across any other PROGRESS characteristics? For example, socioeconomic status	RCT
Di Pollina (2017)										
				Health service utilization	Residential home/apartment	Usual care		No ‐ assessment of effects by sex/gender NOT present	No ‐ assessment of effects across any other PROGRESS characteristics? For example, socioeconomic status	RCT
				Falls						
Dias (2008)										
		Mental functions	Basic needs	Caregiver outcomes	Residential home/apartment	Other		No ‐ assessment of effects by sex/gender NOT present	No ‐ assessment of effects across any other PROGRESS characteristics? For example, socioeconomic status	RCT
Donald (1995)										
		Mental functions	Basic needs		Residential home/apartment	Usual care		No ‐ assessment of effects by sex/gender NOT present	No ‐ assessment of effects across any other PROGRESS characteristics? For example, socioeconomic status	RCT
		Genitourinary and reproductive functions								
		Neuromusculoskeletal function								
Donat (2007)										
		Mental functions	Mobility		Independent living	Other		No ‐ assessment of effects by sex/gender NOT present	No ‐ assessment of effects across any other PROGRESS characteristics? For example, socioeconomic status	RCT
		Sensory functions and pain								
		Neuromusculoskeletal function								
Dorner (2013)										
		Mental functions	Quality of life	Health service utilization	Residential home/apartment	Other		No ‐ assessment of effects by sex/gender NOT present	No ‐ assessment of effects across any other PROGRESS characteristics? For example, socioeconomic status	RCT
		Neuromusculoskeletal function	Mobility							
Dorresteijn (2016)										
			Basic needs	Falls	Residential home/apartment	Usual care		No ‐ assessment of effects by sex/gender NOT present	No ‐ assessment of effects across any other PROGRESS characteristics? For example, socioeconomic status	RCT
Dow (2013)										
		Mental functions		Cost‐effectiveness	Residential home/apartment	Other		No ‐ assessment of effects by sex/gender NOT present	No ‐ assessment of effects across any other PROGRESS characteristics? For example, socioeconomic status	RCT
		Neuromusculoskeletal function		Caregiver outcomes						
				Health service utilization						
Draper (2008)										
		Mental functions	Basic needs	Cost‐effectiveness	Residential home/apartment	Usual care		No ‐ assessment of effects by sex/gender NOT present	No ‐ assessment of effects across any other PROGRESS characteristics? For example, socioeconomic status	RCT
				Caregiver outcomes						
Draper (2016)										
		Sensory functions and pain	Mobility		Residential home/apartment	Usual care	Race, ethnicity, culture, language	No ‐ assessment of effects by sex/gender NOT present	No ‐ assessment of effects across any other PROGRESS characteristics? For example, socioeconomic status	RCT
Duffy (2010)										
			Quality of life	Cost (e.g., out of pocket)	Residential home/apartment	Usual care		No ‐ assessment of effects by sex/gender NOT present	No ‐ assessment of effects across any other PROGRESS characteristics? For example, socioeconomic status	RCT
				Satisfaction of older adult						
				Health service utilization						
Edgren (2015)										
		Neuromusculoskeletal function	Basic needs	Falls	Residential home/apartment	Usual care		No ‐ assessment of effects by sex/gender NOT present	No ‐ assessment of effects across any other PROGRESS characteristics? For example, socioeconomic status	RCT
			Quality of life							
			Mobility							
Eloniemi‐Sulkava (2001)										
		Mental functions		Caregiver outcomes	Residential home/apartment	Other		No ‐ assessment of effects by sex/gender NOT present	No ‐ assessment of effects across any other PROGRESS characteristics? For example, socioeconomic status	RCT
				Health service utilization						
Eloniemi‐Sulkava (2009)										
		Mental functions	Basic needs	Cost (e.g., out of pocket)	Residential home/apartment	Usual care		No ‐ assessment of effects by sex/gender NOT present	No ‐ assessment of effects across any other PROGRESS characteristics? For example, socioeconomic status	RCT
				Cost‐effectiveness						
				Caregiver outcomes						
				Health service utilization						
Engberg (2016)										
		Genitourinary and reproductive functions	Quality of life	Adherence	Residential home/apartment	Other		No ‐ assessment of effects by sex/gender NOT present	No ‐ assessment of effects across any other PROGRESS characteristics? For example, socioeconomic status	RCT
Enguidanos (2012)										
			Basic needs	Caregiver outcomes	Residential home/apartment	Usual care		No ‐ assessment of effects by sex/gender NOT present	No ‐ assessment of effects across any other PROGRESS characteristics? For example, socioeconomic status	RCT
				Health service utilization						
Eriksen (2016)										
		Mental functions	Quality of life	Adherence	Residential home/apartment	Other		No ‐ assessment of effects by sex/gender NOT present	No ‐ assessment of effects across any other PROGRESS characteristics? For example, socioeconomic status	RCT
		Functions of the digestive, metabolic and endocrine systems	Mobility							
		Neuromusculoskeletal function								
Fabacher (1994)										
		Sensory functions and pain	Basic needs		Assisted living	Usual care		No ‐ assessment of effects by sex/gender NOT present	No ‐ assessment of effects across any other PROGRESS characteristics? For example, socioeconomic status	RCT
		Functions of the cardiovascular, haematological, immunological and respiratory systems								
		Functions of the digestive, metabolic and endocrine systems								
		Neuromusculoskeletal function								
Faber (2006)										
		Neuromusculoskeletal function	Basic needs	Falls	Residential home/apartment	Usual care		No ‐ assessment of effects by sex/gender NOT present	No ‐ assessment of effects across any other PROGRESS characteristics? For example, socioeconomic status	RCT
			Mobility		Assisted living					
Fahlström (2018)										
		Neuromusculoskeletal function	Basic needs	Health service utilization	Residential home/apartment	Other		No ‐ assessment of effects by sex/gender NOT present	No ‐ assessment of effects across any other PROGRESS characteristics? For example, socioeconomic status	RCT
			Quality of life	Falls						
			Mobility							
Fairhall (2012)										
		Mental functions	Mobility		Residential home/apartment	Usual care		No ‐ assessment of effects by sex/gender NOT present	No ‐ assessment of effects across any other PROGRESS characteristics? For example, socioeconomic status	RCT
		Neuromusculoskeletal function								
Fairhall (2014)										
	Adaptations to physical environment	Sensory functions and pain	Mobility	Falls	Residential home/apartment	Usual care		No ‐ assessment of effects by sex/gender NOT present	No ‐ assessment of effects across any other PROGRESS characteristics? For example, socioeconomic status	RCT
		Neuromusculoskeletal function								
Fairhall et al. (2015)										
	Adaptations to physical environment	Mental functions	Basic needs	Health service utilization	Residential home/apartment	Usual care		No ‐ assessment of effects by sex/gender NOT present	No ‐ assessment of effects across any other PROGRESS characteristics? For example, socioeconomic status	RCT
		Neuromusculoskeletal function	Quality of life	Falls						
			Mobility							
Fairhall (2017)										
		Functions of the cardiovascular, haematological, immunological and respiratory systems	Mobility		Residential home/apartment	Usual care		No ‐ assessment of effects by sex/gender NOT present	No ‐ assessment of effects across any other PROGRESS characteristics? For example, socioeconomic status	RCT
		Neuromusculoskeletal function								
Farag (2015)										
		Neuromusculoskeletal function	Quality of life	Cost‐effectiveness	Residential home/apartment	Usual care		No ‐ assessment of effects by sex/gender NOT present	No ‐ assessment of effects across any other PROGRESS characteristics? For example, socioeconomic status	RCT
			Mobility	Falls						
Farag (2016)										
				Cost‐effectiveness	Residential home/apartment	Usual care		No ‐ assessment of effects by sex/gender NOT present	No ‐ assessment of effects across any other PROGRESS characteristics? For example, socioeconomic status	RCT
				Health service utilization						
Fasce (2018)										
				Health service utilization	Residential home/apartment	Usual care		No ‐ assessment of effects by sex/gender NOT present	No ‐ assessment of effects across any other PROGRESS characteristics? For example, socioeconomic status	RCT
Favela (2013)										
		Mental functions	Basic needs		Residential home/apartment	Usual care		No ‐ assessment of effects by sex/gender NOT present	No ‐ assessment of effects across any other PROGRESS characteristics? For example, socioeconomic status	RCT
			Quality of life							
			Mobility							
Feldman (2004)										
		Mental functions	Basic needs	Satisfaction of older adult	Residential home/apartment	Usual care		No ‐ assessment of effects by sex/gender NOT present	No ‐ assessment of effects across any other PROGRESS characteristics? For example, socioeconomic status	RCT
			Quality of life	Health service utilization						
Ferrer (2014)										
				Falls	Residential home/apartment	Usual care		No ‐ assessment of effects by sex/gender NOT present	No ‐ assessment of effects across any other PROGRESS characteristics? For example, socioeconomic status	RCT
Ferrer‐Garcia (2011)										
		Functions of the cardiovascular, haematological, immunological and respiratory systems	Quality of life	Adherence	Residential home/apartment	Usual care		No ‐ assessment of effects by sex/gender NOT present	No ‐ assessment of effects across any other PROGRESS characteristics? For example, socioeconomic status	RCT
Fiatarone (1994)										
		Functions of the digestive, metabolic and endocrine systems	Mobility		Long‐term care	Usual care		No ‐ assessment of effects by sex/gender NOT present	No ‐ assessment of effects across any other PROGRESS characteristics? For example, socioeconomic status	RCT
		Neuromusculoskeletal function								
Finnema (2005)										
		Mental functions			Assisted living	Usual care		No ‐ assessment of effects by sex/gender NOT present	No ‐ assessment of effects across any other PROGRESS characteristics? For example, socioeconomic status	RCT
Fleming (2004)										
		Mental functions	Basic needs	Health service utilization	Residential home/apartment	Usual care		No ‐ assessment of effects by sex/gender NOT present	No ‐ assessment of effects across any other PROGRESS characteristics? For example, socioeconomic status	RCT
					Long‐term care					
Flood (2005)										
	Adaptations to physical environment		Quality of life	Cost‐effectiveness	Residential home/apartment			No ‐ assessment of effects by sex/gender NOT present	No ‐ assessment of effects across any other PROGRESS characteristics? For example, socioeconomic status	RCT
Fontan (2010)										
		Mental functions	Basic needs	Health service utilization	Residential home/apartment			No ‐ assessment of effects by sex/gender NOT present	No ‐ assessment of effects across any other PROGRESS characteristics? For example, socioeconomic status	RCT
			Quality of life							
			Build and maintain relationships							
Forsberg (2011)										
		Sensory functions and pain	Basic needs	Health service utilization	Residential home/apartment	Usual care		No ‐ assessment of effects by sex/gender NOT present	No ‐ assessment of effects across any other PROGRESS characteristics? For example, socioeconomic status	RCT
		Neuromusculoskeletal function	Quality of life	Falls						
Forster (1996)										
		Mental functions	Basic needs	Caregiver outcomes	Residential home/apartment	Usual care		No ‐ assessment of effects by sex/gender NOT present	No ‐ assessment of effects across any other PROGRESS characteristics? For example, socioeconomic status	RCT
		Sensory functions and pain	Mobility							
		Genitourinary and reproductive functions	Contribution							
Frese (2012)										
				Health service utilization	Residential home/apartment	Usual care		No ‐ assessment of effects by sex/gender NOT present	No ‐ assessment of effects across any other PROGRESS characteristics? For example, socioeconomic status	RCT
Friedman (2014)										
			Basic needs		Residential home/apartment	Usual care		No ‐ assessment of effects by sex/gender NOT present	No ‐ assessment of effects across any other PROGRESS characteristics? For example, socioeconomic status	RCT
Gagnon (1999)										
			Basic needs	Caregiver outcomes	Assisted living	Usual care		No ‐ assessment of effects by sex/gender NOT present	No ‐ assessment of effects across any other PROGRESS characteristics? For example, socioeconomic status	RCT
			Quality of life	Health service utilization						
Garcia‐Pena (2001)										
		Functions of the cardiovascular, haematological, immunological and respiratory systems			Residential home/apartment	Usual care		No ‐ assessment of effects by sex/gender NOT present	No ‐ assessment of effects across any other PROGRESS characteristics? For example, socioeconomic status	RCT
Garcia‐Pena (2002)										
				Cost (e.g., out of pocket)	Residential home/apartment	Usual care		No ‐ assessment of effects by sex/gender NOT present	No ‐ assessment of effects across any other PROGRESS characteristics? For example, socioeconomic status	RCT
				Cost‐effectiveness						
Gawler (2016)										
		Neuromusculoskeletal function	Mobility	Falls	Residential home/apartment	Other		No ‐ assessment of effects by sex/gender NOT present	No ‐ assessment of effects across any other PROGRESS characteristics? For example, socioeconomic status	RCT
Giangregorio (2018)										
		Neuromusculoskeletal function		Adherence	Residential home/apartment	Usual care		No ‐ assessment of effects by sex/gender NOT present	No ‐ assessment of effects across any other PROGRESS characteristics? For example, socioeconomic status	RCT
				Falls						
Gill (2002)										
	Adaptations to physical environment	Mental functions	Mobility		Residential home/apartment	Other		No ‐ assessment of effects by sex/gender NOT present	No ‐ assessment of effects across any other PROGRESS characteristics? For example, socioeconomic status	RCT
Gill (2004)										
			Basic needs		Residential home/apartment			No ‐ assessment of effects by sex/gender NOT present	No ‐ assessment of effects across any other PROGRESS characteristics? For example, socioeconomic status	RCT
			Mobility							
Gitlin (2001)										
		Mental functions	Basic needs	Caregiver outcomes	Residential home/apartment	Usual care		No ‐ assessment of effects by sex/gender NOT present	No ‐ assessment of effects across any other PROGRESS characteristics? For example, socioeconomic status	RCT
Gitlin (2006)										
	Adaptations to physical environment	Mental functions	Basic needs	Falls	Residential home/apartment	Usual care		No ‐ assessment of effects by sex/gender NOT present	No ‐ assessment of effects across any other PROGRESS characteristics? For example, socioeconomic status	RCT
		Functions of the cardiovascular, haematological, immunological and respiratory systems								
		Functions of the digestive, metabolic and endocrine systems								
Gitlin (2008)										
		Mental functions	Quality of life	Caregiver outcomes	Residential home/apartment	Other		No ‐ assessment of effects by sex/gender NOT present	No ‐ assessment of effects across any other PROGRESS characteristics? For example, socioeconomic status	RCT
		Genitourinary and reproductive functions								
Gitlin (2009)										
					Residential home/apartment	Usual care		No ‐ assessment of effects by sex/gender NOT present	Yes ‐ assessment of effects across any other PROGRESS characteristics? For example, socioeconomic status	RCT
Gitlin (2010)										
		Functions of the cardiovascular, haematological, immunological and respiratory systems	Quality of life		Residential home/apartment	Other		No ‐ assessment of effects by sex/gender NOT present	No ‐ assessment of effects across any other PROGRESS characteristics? For example, socioeconomic status	RCT
		Genitourinary and reproductive functions								
Gitlin (2014)										
		Mental functions		Health service utilization	Residential home/apartment	Other		No ‐ assessment of effects by sex/gender NOT present	No ‐ assessment of effects across any other PROGRESS characteristics? For example, socioeconomic status	RCT
Gitlin (2018)										
		Mental functions	Basic needs	Caregiver outcomes	Residential home/apartment	Other		No ‐ assessment of effects by sex/gender NOT present	No ‐ assessment of effects across any other PROGRESS characteristics? For example, socioeconomic status	RCT
		Sensory functions and pain								
							
Gladman (1993)										
		Mental functions	Basic needs	Caregiver outcomes	Residential home/apartment	Usual care		No ‐ assessment of effects by sex/gender NOT present	No ‐ assessment of effects across any other PROGRESS characteristics? For example, socioeconomic status	RCT
		Sensory functions and pain	Mobility	Health service utilization						
			Contribution							
Godwin (2016)										
		Mental functions	Quality of life	Satisfaction of older adult	Residential home/apartment	Usual care		No ‐ assessment of effects by sex/gender NOT present	No ‐ assessment of effects across any other PROGRESS characteristics? For example, socioeconomic status	RCT
		Sensory functions and pain	Contribution	Access						
		Functions of the cardiovascular, haematological, immunological and respiratory systems		Health service utilization						
		Functions of the digestive, metabolic and endocrine systems								
		Genitourinary and reproductive functions								
		Neuromusculoskeletal function								
Gozalo (2014)										
		Mental functions			Assisted living	Other		No ‐ assessment of effects by sex/gender NOT present	No ‐ assessment of effects across any other PROGRESS characteristics? For example, socioeconomic status	RCT
Graff (2008)										
				Cost‐effectiveness	Residential home/apartment	Usual care		No ‐ assessment of effects by sex/gender NOT present	No ‐ assessment of effects across any other PROGRESS characteristics? For example, socioeconomic status	RCT
Granbom (2017)										
			Contribution		Residential home/apartment	Usual care		No ‐ assessment of effects by sex/gender NOT present	No ‐ assessment of effects across any other PROGRESS characteristics? For example, socioeconomic status	RCT
			Build and maintain relationships							
Graves (2009)										
				Cost‐effectiveness	Residential home/apartment	Usual care		No ‐ assessment of effects by sex/gender NOT present	No ‐ assessment of effects across any other PROGRESS characteristics? For example, socioeconomic status	RCT
Grimmer (2013)										
			Basic needs	Health service utilization	Residential home/apartment	Usual care		No ‐ assessment of effects by sex/gender NOT present	No ‐ assessment of effects across any other PROGRESS characteristics? For example, socioeconomic status	RCT
			Quality of life	Falls						
			Mobility							
Gronstedt (2013)										
		Neuromusculoskeletal function	Mobility		Assisted living	Usual care		No ‐ assessment of effects by sex/gender NOT present	No ‐ assessment of effects across any other PROGRESS characteristics? For example, socioeconomic status	RCT
Gustafsson (2012)										
		Mental functions	Basic needs		Residential home/apartment	Usual care		No ‐ assessment of effects by sex/gender NOT present	No ‐ assessment of effects across any other PROGRESS characteristics? For example, socioeconomic status	RCT
		Neuromusculoskeletal function	Mobility							
Haastregt (2000)										
		Mental functions	Basic needs	Falls	Residential home/apartment	Usual care		No ‐ assessment of effects by sex/gender NOT present	No ‐ assessment of effects across any other PROGRESS characteristics? For example, socioeconomic status	RCT
			Mobility							
			Contribution							
Haider (2017)										
		Functions of the digestive, metabolic and endocrine systems	Mobility	Adherence	Residential home/apartment	Other		No ‐ assessment of effects by sex/gender NOT present	No ‐ assessment of effects across any other PROGRESS characteristics? For example, socioeconomic status	RCT
		Neuromusculoskeletal function		Safety						
Haider (2017)										
		Functions of the cardiovascular, haematological, immunological and respiratory systems	Mobility	Adherence	Residential home/apartment	Other		No ‐ assessment of effects by sex/gender NOT present	No ‐ assessment of effects across any other PROGRESS characteristics? For example, socioeconomic status	RCT
		Functions of the digestive, metabolic and endocrine systems								
		Neuromusculoskeletal function								
Hall (1992)										
		Mental functions		Health service utilization	Residential home/apartment	Usual care		No ‐ assessment of effects by sex/gender NOT present	No ‐ assessment of effects across any other PROGRESS characteristics? For example, socioeconomic status	RCT
Hammar (2009)										
				Cost‐effectiveness	Residential home/apartment	Usual care		No ‐ assessment of effects by sex/gender NOT present	No ‐ assessment of effects across any other PROGRESS characteristics? For example, socioeconomic status	RCT
Hansen (1992)										
				Health service utilization	Residential home/apartment	Usual care		No ‐ assessment of effects by sex/gender NOT present	No ‐ assessment of effects across any other PROGRESS characteristics? For example, socioeconomic status	RCT
Hansen (1995)										
				Health service utilization	Residential home/apartment	Usual care		No ‐ assessment of effects by sex/gender NOT present	No ‐ assessment of effects across any other PROGRESS characteristics? For example, socioeconomic status	RCT
Harris (2005)										
				Satisfaction of older adult	Residential home/apartment	Usual care		No ‐ assessment of effects by sex/gender NOT present	No ‐ assessment of effects across any other PROGRESS characteristics? For example, socioeconomic status	RCT
				Cost‐effectiveness						
Harvey (2014)										
				Satisfaction of older adult	Residential home/apartment	Usual care		No ‐ assessment of effects by sex/gender NOT present	No ‐ assessment of effects across any other PROGRESS characteristics? For example, socioeconomic status	RCT
				Health service utilization						
Hauer (2017)										
		Neuromusculoskeletal function	Mobility	Falls	Assisted living	Usual care		No ‐ assessment of effects by sex/gender NOT present	No ‐ assessment of effects across any other PROGRESS characteristics? For example, socioeconomic status	RCT
Helbostad (2004)										
		Neuromusculoskeletal function	Mobility	Adherence	Residential home/apartment	Other		No ‐ assessment of effects by sex/gender NOT present	No ‐ assessment of effects across any other PROGRESS characteristics? For example, socioeconomic status	RCT
				Falls						
Hendriks (2008)										
		Mental functions	Basic needs	Adherence	Residential home/apartment	Usual care		No ‐ assessment of effects by sex/gender NOT present	No ‐ assessment of effects across any other PROGRESS characteristics? For example, socioeconomic status	RCT
			Quality of life	Falls						
			Contribution							
Herfjord (2014)										
		Sensory functions and pain	Basic needs	Cost‐effectiveness	Assisted living	Usual care		No ‐ assessment of effects by sex/gender NOT present	No ‐ assessment of effects across any other PROGRESS characteristics? For example, socioeconomic status	RCT
			Quality of life	Health service utilization						
Hewitt (2018)										
		Mental functions	Quality of life	Falls	Long‐term care	Usual care		No ‐ assessment of effects by sex/gender NOT present	No ‐ assessment of effects across any other PROGRESS characteristics? For example, socioeconomic status	RCT
		Neuromusculoskeletal function	Mobility							
Hinrichs (2015)										
		Functions of the digestive, metabolic and endocrine systems	Mobility	Safety	Residential home/apartment	Other		No ‐ assessment of effects by sex/gender NOT present	No ‐ assessment of effects across any other PROGRESS characteristics? For example, socioeconomic status	RCT
Hinrichs (2016)										
		Neuromusculoskeletal function	Mobility	Adherence	Residential home/apartment	Usual care		No ‐ assessment of effects by sex/gender NOT present	No ‐ assessment of effects across any other PROGRESS characteristics? For example, socioeconomic status	RCT
				Safety						
Hoenig (2015)										
		Mental functions	Mobility	Safety	Residential home/apartment	Other		No ‐ assessment of effects by sex/gender NOT present	No ‐ assessment of effects across any other PROGRESS characteristics? For example, socioeconomic status	RCT
		Sensory functions and pain								
Holland (2005)										
			Quality of life	Health service utilization	Residential home/apartment	Usual care		No ‐ assessment of effects by sex/gender NOT present	No ‐ assessment of effects across any other PROGRESS characteristics? For example, socioeconomic status	RCT
Holland (2017)										
		Mental functions	Basic needs	Health service utilization	Residential home/apartment	Usual care		No ‐ assessment of effects by sex/gender NOT present	No ‐ assessment of effects across any other PROGRESS characteristics? For example, socioeconomic status	RCT
		Functions of the cardiovascular, haematological, immunological and respiratory systems	Quality of life							
			Mobility							
Houles (2010)										
		Mental functions	Basic needs		Residential home/apartment	Usual care		No ‐ assessment of effects by sex/gender NOT present	No ‐ assessment of effects across any other PROGRESS characteristics? For example, socioeconomic status	RCT
			Quality of life							
			Mobility							
Hsu (2016)										
		Mental functions			Long‐term care	Usual care		No ‐ assessment of effects by sex/gender NOT present	No ‐ assessment of effects across any other PROGRESS characteristics? For example, socioeconomic status	RCT
Hsu (2016)										
		Mental functions	Quality of life		Long‐term care	Usual care		No ‐ assessment of effects by sex/gender NOT present	No ‐ assessment of effects across any other PROGRESS characteristics? For example, socioeconomic status	RCT
Huang (1998)										
		Mental functions		Safety	Residential home/apartment	Usual care		No ‐ assessment of effects by sex/gender NOT present	No ‐ assessment of effects across any other PROGRESS characteristics? For example, socioeconomic status	RCT
				Falls						
Huang (2013)										
		Mental functions		Caregiver outcomes	Residential home/apartment	Other		No ‐ assessment of effects by sex/gender NOT present	No ‐ assessment of effects across any other PROGRESS characteristics? For example, socioeconomic status	RCT
Hughes (1992)										
			Basic needs	Cost (e.g., out of pocket)	Long‐term care	Other		No ‐ assessment of effects by sex/gender NOT present	No ‐ assessment of effects across any other PROGRESS characteristics? For example, socioeconomic status	RCT
				Cost‐effectiveness	Independent living					
				Caregiver outcomes						
				Health service utilization						
Hughes (2000)										
		Mental functions	Quality of life	Satisfaction of older adult	Assisted living	Usual care		No ‐ assessment of effects by sex/gender NOT present	No ‐ assessment of effects across any other PROGRESS characteristics? For example, socioeconomic status	RCT
				Cost‐effectiveness						
		Caregiver outcomes					
Hunger (2015)										
		Mental functions	Basic needs		Residential home/apartment	Usual care		No ‐ assessment of effects by sex/gender NOT present	No ‐ assessment of effects across any other PROGRESS characteristics? For example, socioeconomic status	RCT
		Functions of the cardiovascular, haematological, immunological and respiratory systems								
Wang et al. (2016)										
		Mental functions		Falls	Residential home/apartment	Other		No ‐ assessment of effects by sex/gender NOT present	No ‐ assessment of effects across any other PROGRESS characteristics? For example, socioeconomic status	RCT
		Neuromusculoskeletal function								
Iliffe (2014)										
		Neuromusculoskeletal function	Quality of life	Cost (e.g., out of pocket)	Residential home/apartment	Other		No ‐ assessment of effects by sex/gender NOT present	No ‐ assessment of effects across any other PROGRESS characteristics? For example, socioeconomic status	RCT
Imhof (2012)										
			Quality of life	Health service utilization	Residential home/apartment	Usual care		No ‐ assessment of effects by sex/gender NOT present	No ‐ assessment of effects across any other PROGRESS characteristics? For example, socioeconomic status	RCT
				Falls						
Inglis (2006)										
				Cost‐effectiveness	Residential home/apartment	Usual care		No ‐ assessment of effects by sex/gender NOT present	No ‐ assessment of effects across any other PROGRESS characteristics? For example, socioeconomic status	RCT
				Health service utilization						
Isrctn (2018)										
		Mental functions	Basic needs	Caregiver outcomes	Residential home/apartment	Usual care		No ‐ assessment of effects by sex/gender NOT present	No ‐ assessment of effects across any other PROGRESS characteristics? For example, socioeconomic status	RCT
		Sensory functions and pain	Quality of life							
			Build and maintain relationships							
Jakobsen (2007)										
			Basic needs	Satisfaction of older adult	Residential home/apartment	Usual care		No ‐ assessment of effects by sex/gender NOT present	No ‐ assessment of effects across any other PROGRESS characteristics? For example, socioeconomic status	RCT
				Cost‐effectiveness						
				Health service utilization						
Jensen (2002)										
	Adaptations to physical environment			Falls	Long‐term care	Usual care		No ‐ assessment of effects by sex/gender NOT present	No ‐ assessment of effects across any other PROGRESS characteristics? For example, socioeconomic status	RCT
Jingna (2012)										
		Mental functions			Residential home/apartment	Usual care		No ‐ assessment of effects by sex/gender NOT present	No ‐ assessment of effects across any other PROGRESS characteristics? For example, socioeconomic status	RCT
Joaquim (2017)										
			Basic needs		Residential home/apartment	Usual care		No ‐ assessment of effects by sex/gender NOT present	No ‐ assessment of effects across any other PROGRESS characteristics? For example, socioeconomic status	RCT
Johansson (2001)										
		Mental functions		Health service utilization	Residential home/apartment	Usual care		No ‐ assessment of effects by sex/gender NOT present	No ‐ assessment of effects across any other PROGRESS characteristics? For example, socioeconomic status	RCT
Johansson (2003)										
				Cost‐effectiveness	Residential home/apartment	Usual care		No ‐ assessment of effects by sex/gender NOT present	No ‐ assessment of effects across any other PROGRESS characteristics? For example, socioeconomic status	RCT
				Health service utilization						
Jolly (2009)										
		Mental functions		Cost‐effectiveness	Residential home/apartment	Other		No ‐ assessment of effects by sex/gender NOT present	No ‐ assessment of effects across any other PROGRESS characteristics? For example, socioeconomic status	RCT
		Functions of the cardiovascular, haematological, immunological and respiratory systems		Health service utilization						
Kalra (2000)										
			Basic needs	Health service utilization	Residential home/apartment	Other		No ‐ assessment of effects by sex/gender NOT present	No ‐ assessment of effects across any other PROGRESS characteristics? For example, socioeconomic status	RCT
Kane (1984)										
		Mental functions	Basic needs	Satisfaction of older adult	Residential home/apartment	Usual care		No ‐ assessment of effects by sex/gender NOT present	No ‐ assessment of effects across any other PROGRESS characteristics? For example, socioeconomic status	RCT
		Sensory functions and pain	Learning, grow and make decisions	Caregiver outcomes	Long‐term care					
Kanemaru (2010)										
		Neuromusculoskeletal function	Quality of life		Residential home/apartment	Usual care		No ‐ assessment of effects by sex/gender NOT present	No ‐ assessment of effects across any other PROGRESS characteristics? For example, socioeconomic status	RCT
Kapan (2017)										
		Mental functions	Basic needs	Falls	Residential home/apartment	Other		No ‐ assessment of effects by sex/gender NOT present	No ‐ assessment of effects across any other PROGRESS characteristics? For example, socioeconomic status	RCT
		Neuromusculoskeletal function	Mobility							
Kapan (2017)										
			Quality of life		Residential home/apartment	Other		No ‐ assessment of effects by sex/gender NOT present	No ‐ assessment of effects across any other PROGRESS characteristics? For example, socioeconomic status	RCT
Karinkanta (2007)										
		Neuromusculoskeletal function	Mobility		Residential home/apartment	Usual care		No ‐ assessment of effects by sex/gender NOT present	No ‐ assessment of effects across any other PROGRESS characteristics? For example, socioeconomic status	RCT
Karlsson (2016)										
			Mobility		Assisted living	Other		No ‐ assessment of effects by sex/gender NOT present	No ‐ assessment of effects across any other PROGRESS characteristics? For example, socioeconomic status	RCT
Kerr (2018)										
		Mental functions	Quality of life		Independent living	Other		No ‐ assessment of effects by sex/gender NOT present	No ‐ assessment of effects across any other PROGRESS characteristics? For example, socioeconomic status	RCT
		Sensory functions and pain	Mobility							
		Functions of the cardiovascular, haematological, immunological and respiratory systems								
Kerse (2010)										
		Mental functions	Quality of life		Residential home/apartment	Other		No ‐ assessment of effects by sex/gender NOT present	No ‐ assessment of effects across any other PROGRESS characteristics? For example, socioeconomic status	RCT
			Mobility							
Kim (2011)										
		Neuromusculoskeletal function	Quality of life		Residential home/apartment	Other		No ‐ assessment of effects by sex/gender NOT present	No ‐ assessment of effects across any other PROGRESS characteristics? For example, socioeconomic status	RCT
			Mobility							
King et al. (2012)										
		Mental functions	Quality of life		Residential home/apartment	Usual care		No ‐ assessment of effects by sex/gender NOT present	No ‐ assessment of effects across any other PROGRESS characteristics? For example, socioeconomic status	RCT
			Mobility							
King et al. (2012)										
		Mental functions	Basic needs		Residential home/apartment	Usual care		No ‐ assessment of effects by sex/gender NOT present	No ‐ assessment of effects across any other PROGRESS characteristics? For example, socioeconomic status	RCT
			Quality of life							
Kjerstad (2016)										
				Cost‐effectiveness	Residential home/apartment	Usual care		No ‐ assessment of effects by sex/gender NOT present	No ‐ assessment of effects across any other PROGRESS characteristics? For example, socioeconomic status	RCT
Klug (2011)										
		Mental functions	Quality of life	Health service utilization	Residential home/apartment	Usual care		No ‐ assessment of effects by sex/gender NOT present	No ‐ assessment of effects across any other PROGRESS characteristics? For example, socioeconomic status	RCT
Kocic (2018)										
		Neuromusculoskeletal function	Basic needs		Assisted living	Usual care		No ‐ assessment of effects by sex/gender NOT present	No ‐ assessment of effects across any other PROGRESS characteristics? For example, socioeconomic status	RCT
Kohei (2016)										
		Neuromusculoskeletal function	Quality of life		Residential home/apartment	Other		No ‐ assessment of effects by sex/gender NOT present	No ‐ assessment of effects across any other PROGRESS characteristics? For example, socioeconomic status	RCT
			Mobility							
Kono (2004)										
		Mental functions	Basic needs		Residential home/apartment	Usual care		No ‐ assessment of effects by sex/gender NOT present	No ‐ assessment of effects across any other PROGRESS characteristics? For example, socioeconomic status	RCT
Kono (2012)										
		Mental functions		Health service utilization	Residential home/apartment	Usual care		No ‐ assessment of effects by sex/gender NOT present	No ‐ assessment of effects across any other PROGRESS characteristics? For example, socioeconomic status	RCT
Kono (2013)										
				Cost‐effectiveness	Residential home/apartment	Usual care		No ‐ assessment of effects by sex/gender NOT present	No ‐ assessment of effects across any other PROGRESS characteristics? For example, socioeconomic status	RCT
				Health service utilization						
Kono (2014)										
		Mental functions	Basic needs	Satisfaction of older adult	Residential home/apartment	Usual care		No ‐ assessment of effects by sex/gender NOT present	No ‐ assessment of effects across any other PROGRESS characteristics? For example, socioeconomic status	RCT
			Quality of life	Health service utilization						
				Falls						
Kono (2016)										
		Mental functions	Basic needs	Satisfaction of older adult	Residential home/apartment	Usual care		No ‐ assessment of effects by sex/gender NOT present	No ‐ assessment of effects across any other PROGRESS characteristics? For example, socioeconomic status	RCT
				Health service utilization						
		Falls					
Kronborg (2006)										
			Basic needs	Cost‐effectiveness	Residential home/apartment	Usual care		No ‐ assessment of effects by sex/gender NOT present	No ‐ assessment of effects across any other PROGRESS characteristics? For example, socioeconomic status	RCT
Kukkonen‐Harjula (2018)										
		Mental functions	Basic needs		Residential home/apartment	Usual care		No ‐ assessment of effects by sex/gender NOT present	No ‐ assessment of effects across any other PROGRESS characteristics? For example, socioeconomic status	RCT
		Neuromusculoskeletal function	Quality of life							
Kwok (2004)										
		Mental functions	Basic needs	Caregiver outcomes	Residential home/apartment	Usual care		No ‐ assessment of effects by sex/gender NOT present	No ‐ assessment of effects across any other PROGRESS characteristics? For example, socioeconomic status	RCT
		Functions of the cardiovascular, haematological, immunological and respiratory systems		Health service utilization						
Kwok (2016)										
		Mental functions	Basic needs		Residential home/apartment	Other		No ‐ assessment of effects by sex/gender NOT present	No ‐ assessment of effects across any other PROGRESS characteristics? For example, socioeconomic status	RCT
		Sensory functions and pain	Quality of life							
			Mobility							
Kyrdalen (2014)										
		Mental functions	Quality of life	Falls	Residential home/apartment	Other		No ‐ assessment of effects by sex/gender NOT present	No ‐ assessment of effects across any other PROGRESS characteristics? For example, socioeconomic status	RCT
		Neuromusculoskeletal function	Mobility							
Lam (2018)										
		Neuromusculoskeletal function	Mobility	Falls	Assisted living	Other		No ‐ assessment of effects by sex/gender NOT present	No ‐ assessment of effects across any other PROGRESS characteristics? For example, socioeconomic status	RCT
Lannin (2007)										
			Basic needs	Health service utilization	Residential home/apartment	Usual care		No ‐ assessment of effects by sex/gender NOT present	No ‐ assessment of effects across any other PROGRESS characteristics? For example, socioeconomic status	RCT
			Quality of life	Falls						
			Mobility							
Latham (2014)										
		Neuromusculoskeletal function	Basic needs	Adherence	Residential home/apartment	Other		No ‐ assessment of effects by sex/gender NOT present	No ‐ assessment of effects across any other PROGRESS characteristics? For example, socioeconomic status	RCT
			Mobility	Safety						
		Falls					
Latour (2007)										
		Mental functions	Quality of life	Cost‐effectiveness	Residential home/apartment	Usual care		No ‐ assessment of effects by sex/gender NOT present	No ‐ assessment of effects across any other PROGRESS characteristics? For example, socioeconomic status	RCT
				Health service utilization						
Lattanzio (2001)										
		Mental functions	Basic needs		Residential home/apartment	Other		No ‐ assessment of effects by sex/gender NOT present	No ‐ assessment of effects across any other PROGRESS characteristics? For example, socioeconomic status	RCT
		Functions of the cardiovascular, haematological, immunological and respiratory systems	Quality of life							
Leavitt (2018)										
			Quality of life	Cost‐effectiveness	Residential home/apartment	Usual care		No ‐ assessment of effects by sex/gender NOT present	No ‐ assessment of effects across any other PROGRESS characteristics? For example, socioeconomic status	RCT
			Learning, grow and make decisions	Health service utilization						
Lee (2006)										
		Functions of the cardiovascular, haematological, immunological and respiratory systems	Mobility		Residential home/apartment	Usual care		No ‐ assessment of effects by sex/gender NOT present	No ‐ assessment of effects across any other PROGRESS characteristics? For example, socioeconomic status	RCT
Lenaghan (2007)										
				Health service utilization	Residential home/apartment	Usual care		No ‐ assessment of effects by sex/gender NOT present	No ‐ assessment of effects across any other PROGRESS characteristics? For example, socioeconomic status	RCT
Levine (2012)										
				Satisfaction of older adult	Residential home/apartment	Usual care		No ‐ assessment of effects by sex/gender NOT present	No ‐ assessment of effects across any other PROGRESS characteristics? For example, socioeconomic status	RCT
				Cost‐effectiveness						
				Health service utilization						
Lewin (2013)										
			Basic needs		Residential home/apartment	Usual care		No ‐ assessment of effects by sex/gender NOT present	No ‐ assessment of effects across any other PROGRESS characteristics? For example, socioeconomic status	RCT
			Quality of life							
Lewin (2014)										
				Cost (e.g., out of pocket)	Residential home/apartment	Usual care		No ‐ assessment of effects by sex/gender NOT present	No ‐ assessment of effects across any other PROGRESS characteristics? For example, socioeconomic status	RCT
				Health service utilization						
Li (2013)										
			Basic needs		Residential home/apartment	Usual care		No ‐ assessment of effects by sex/gender NOT present	No ‐ assessment of effects across any other PROGRESS characteristics? For example, socioeconomic status	RCT
Li (2015)										
		Functions of the cardiovascular, haematological, immunological and respiratory systems	Quality of life		Residential home/apartment	Usual care		No ‐ assessment of effects by sex/gender NOT present	No ‐ assessment of effects across any other PROGRESS characteristics? For example, socioeconomic status	RCT
		Neuromusculoskeletal function								
Liang (1984)										
			Basic needs	Health service utilization	Residential home/apartment	Usual care		No ‐ assessment of effects by sex/gender NOT present	No ‐ assessment of effects across any other PROGRESS characteristics? For example, socioeconomic status	RCT
			Quality of life	Falls						
			Mobility							
Liang (1986)										
			Basic needs	Satisfaction of older adult	Residential home/apartment	Usual care		No ‐ assessment of effects by sex/gender NOT present	No ‐ assessment of effects across any other PROGRESS characteristics? For example, socioeconomic status	RCT
			Quality of life	Health service utilization						
				Falls						
Liddle (1996)										
	Adaptations to physical environment	Mental functions	Basic needs	Satisfaction of older adult	Residential home/apartment	Usual care		No ‐ assessment of effects by sex/gender NOT present	No ‐ assessment of effects across any other PROGRESS characteristics? For example, socioeconomic status	RCT
			Quality of life							
			Mobility							
Liimatta (2017)										
				Satisfaction of older adult	Residential home/apartment	Usual care		No ‐ assessment of effects by sex/gender NOT present	No ‐ assessment of effects across any other PROGRESS characteristics? For example, socioeconomic status	RCT
Lin (2007)										
		Mental functions	Basic needs	Falls	Residential home/apartment	Other		No ‐ assessment of effects by sex/gender NOT present	No ‐ assessment of effects across any other PROGRESS characteristics? For example, socioeconomic status	RCT
		Neuromusculoskeletal function	Quality of life							
			Mobility							
Lin (2010)										
		Mental functions	Basic needs		Long‐term care	Usual care		No ‐ assessment of effects by sex/gender NOT present	No ‐ assessment of effects across any other PROGRESS characteristics? For example, socioeconomic status	RCT
		Functions of the digestive, metabolic and endocrine systems								
Lindegaard (2017)										
				Health service utilization	Residential home/apartment	Other		No ‐ assessment of effects by sex/gender NOT present	No ‐ assessment of effects across any other PROGRESS characteristics? For example, socioeconomic status	RCT
Lindegaard‐Pedersen (2015)										
		Mental functions	Basic needs	Health service utilization	Residential home/apartment	Usual care		No ‐ assessment of effects by sex/gender NOT present	No ‐ assessment of effects across any other PROGRESS characteristics? For example, socioeconomic status	RCT
		Neuromusculoskeletal function	Quality of life							
Liu and Lai (2014)										
		Neuromusculoskeletal function	Basic needs		Residential home/apartment	Usual care		No ‐ assessment of effects by sex/gender NOT present	No ‐ assessment of effects across any other PROGRESS characteristics? For example, socioeconomic status	RCT
Liu (2015)										
		Mental functions	Basic needs		Assisted living	Usual care		No ‐ assessment of effects by sex/gender NOT present	No ‐ assessment of effects across any other PROGRESS characteristics? For example, socioeconomic status	RCT
		Neuromusculoskeletal function								
Locher (2013)										
		Functions of the digestive, metabolic and endocrine systems	Basic needs		Residential home/apartment	Usual care		No ‐ assessment of effects by sex/gender NOT present	No ‐ assessment of effects across any other PROGRESS characteristics? For example, socioeconomic status	RCT
Logan (2004)										
		Mental functions	Basic needs	Caregiver outcomes	Residential home/apartment	Usual care		No ‐ assessment of effects by sex/gender NOT present	No ‐ assessment of effects across any other PROGRESS characteristics? For example, socioeconomic status	RCT
			Mobility							
			Contribution							
Lok (2017)										
		Mental functions	Quality of life		Residential home/apartment	Usual care		No ‐ assessment of effects by sex/gender NOT present	No ‐ assessment of effects across any other PROGRESS characteristics? For example, socioeconomic status	RCT
		Sensory functions and pain								
Luck (2013)										
				Health service utilization	Residential home/apartment	Usual care		No ‐ assessment of effects by sex/gender NOT present	No ‐ assessment of effects across any other PROGRESS characteristics? For example, socioeconomic status	RCT
				Falls						
Lyons (2016)										
			Basic needs		Residential home/apartment	Usual care		No ‐ assessment of effects by sex/gender NOT present	No ‐ assessment of effects across any other PROGRESS characteristics? For example, socioeconomic status	RCT
			Mobility							
			Learning, grow and make decisions							
			Build and maintain relationships							
MacIntyre (1999)										
		Mental functions	Quality of life	Satisfaction of older adult	Residential home/apartment	Usual care		No ‐ assessment of effects by sex/gender NOT present	No ‐ assessment of effects across any other PROGRESS characteristics? For example, socioeconomic status	RCT
		Sensory functions and pain	Build and maintain relationships							
Mahoney (2007)										
				Health service utilization	Residential home/apartment	Other		No ‐ assessment of effects by sex/gender NOT present	No ‐ assessment of effects across any other PROGRESS characteristics? For example, socioeconomic status	RCT
				Falls						
Maiers (2014)										
		Mental functions		Satisfaction of older adult	Residential home/apartment	Other		No ‐ assessment of effects by sex/gender NOT present	No ‐ assessment of effects across any other PROGRESS characteristics? For example, socioeconomic status	RCT
		Sensory functions and pain								
		Neuromusculoskeletal function								
Mangione (2005)										
		Mental functions	Mobility	Adherence	Residential home/apartment	Usual care		No ‐ assessment of effects by sex/gender NOT present	No ‐ assessment of effects across any other PROGRESS characteristics? For example, socioeconomic status	RCT
		Neuromusculoskeletal function								
Mangione et al. (2010)										
		Neuromusculoskeletal function	Basic needs		Residential home/apartment	Other		No ‐ assessment of effects by sex/gender NOT present	No ‐ assessment of effects across any other PROGRESS characteristics? For example, socioeconomic status	RCT
			Mobility							
Mann (1999)										
	Adaptations to physical environment	Mental functions	Basic needs	Cost (e.g., out of pocket)	Residential home/apartment	Usual care		No ‐ assessment of effects by sex/gender NOT present	No ‐ assessment of effects across any other PROGRESS characteristics? For example, socioeconomic status	RCT
		Sensory functions and pain	Mobility	Cost‐effectiveness						
			Contribution							
			Build and maintain relationships							
Marek (2014)										
		Mental functions	Basic needs	Cost (e.g., out of pocket)	Residential home/apartment	Usual care		No ‐ assessment of effects by sex/gender NOT present	No ‐ assessment of effects across any other PROGRESS characteristics? For example, socioeconomic status	RCT
		Neuromusculoskeletal function	Quality of life	Cost‐effectiveness						
Markle‐Reid (2003)										
		Mental functions	Basic needs	Cost (e.g., out of pocket)	Residential home/apartment	Usual care		No ‐ assessment of effects by sex/gender NOT present	No ‐ assessment of effects across any other PROGRESS characteristics? For example, socioeconomic status	RCT
		Sensory functions and pain	Quality of life	Cost‐effectiveness						
			Contribution	Caregiver outcomes						
			Build and maintain relationships	Health service utilization						
Markle‐Reid (2006)										
		Mental functions	Basic needs	Cost‐effectiveness	Residential home/apartment	Usual care		No ‐ assessment of effects by sex/gender NOT present	No ‐ assessment of effects across any other PROGRESS characteristics? For example, socioeconomic status	RCT
		Sensory functions and pain	Quality of life							
			Contribution							
Markle‐Reid (2010)										
		Mental functions	Basic needs	Cost (e.g., out of pocket)	Residential home/apartment	Usual care		No ‐ assessment of effects by sex/gender NOT present	No ‐ assessment of effects across any other PROGRESS characteristics? For example, socioeconomic status	RCT
		Neuromusculoskeletal function	Quality of life	Cost‐effectiveness						
				Falls						
Markle‐Reid et al. (2017)										
		Mental functions	Basic needs	Cost (e.g., out of pocket)	Residential home/apartment	Usual care		No ‐ assessment of effects by sex/gender NOT present	No ‐ assessment of effects across any other PROGRESS characteristics? For example, socioeconomic status	RCT
		Functions of the digestive, metabolic and endocrine systems	Quality of life	Cost‐effectiveness						
				Caregiver outcomes						
Martin (1994)										
		Mental functions	Basic needs	Health service utilization	Residential home/apartment	Usual care		No ‐ assessment of effects by sex/gender NOT present	No ‐ assessment of effects across any other PROGRESS characteristics? For example, socioeconomic status	RCT
								
Maru (2015)										
				Cost‐effectiveness	Residential home/apartment	Other		No ‐ assessment of effects by sex/gender NOT present	No ‐ assessment of effects across any other PROGRESS characteristics? For example, socioeconomic status	RCT
				Health service utilization						
Matzen (2007)										
				Satisfaction of older adult	Residential home/apartment	Other		No ‐ assessment of effects by sex/gender NOT present	No ‐ assessment of effects across any other PROGRESS characteristics? For example, socioeconomic status	RCT
				Health service utilization	Assisted living					
Mayo (2008)										
		Mental functions	Basic needs		Residential home/apartment	Usual care		No ‐ assessment of effects by sex/gender NOT present	No ‐ assessment of effects across any other PROGRESS characteristics? For example, socioeconomic status	RCT
		Neuromusculoskeletal function	Quality of life							
McCorkle (1989)										
		Mental functions		Health service utilization	Residential home/apartment	Other		No ‐ assessment of effects by sex/gender NOT present	No ‐ assessment of effects across any other PROGRESS characteristics? For example, socioeconomic status	RCT
		Sensory functions and pain								
McCorkle (2000)										
		Mental functions	Basic needs		Residential home/apartment	Usual care		No ‐ assessment of effects by sex/gender NOT present	No ‐ assessment of effects across any other PROGRESS characteristics? For example, socioeconomic status	RCT
			Contribution							
McMurdo (1995)										
		Neuromusculoskeletal function	Basic needs		Residential home/apartment	Other		No ‐ assessment of effects by sex/gender NOT present	No ‐ assessment of effects across any other PROGRESS characteristics? For example, socioeconomic status	RCT
			Quality of life							
			Mobility							
McWilliam (1999)										
		Mental functions	Quality of life	Health service utilization	Residential home/apartment	Usual care		No ‐ assessment of effects by sex/gender NOT present	No ‐ assessment of effects across any other PROGRESS characteristics? For example, socioeconomic status	RCT
								
McWilliam (1999)										
		Mental functions	Quality of life	Health service utilization	Residential home/apartment	Usual care		No ‐ assessment of effects by sex/gender NOT present	No ‐ assessment of effects across any other PROGRESS characteristics? For example, socioeconomic status	RCT
								
Meisinger (2013)										
		Mental functions	Basic needs	Cost‐effectiveness	Residential home/apartment	Usual care		No ‐ assessment of effects by sex/gender NOT present	No ‐ assessment of effects across any other PROGRESS characteristics? For example, socioeconomic status	RCT
		Functions of the cardiovascular, haematological, immunological and respiratory systems	Quality of life	Health service utilization						
Melin (1992)										
		Mental functions	Basic needs	Health service utilization	Residential home/apartment	Usual care		No ‐ assessment of effects by sex/gender NOT present	No ‐ assessment of effects across any other PROGRESS characteristics? For example, socioeconomic status	RCT
			Mobility							
			Contribution							
			Build and maintain relationships							
Melin (1993)										
			Basic needs		Residential home/apartment	Usual care		No ‐ assessment of effects by sex/gender NOT present	No ‐ assessment of effects across any other PROGRESS characteristics? For example, socioeconomic status	RCT
			Mobility							
			Contribution							
Melin (1993)										
		Mental functions	Basic needs	Cost (e.g., out of pocket)	Residential home/apartment	Usual care		No ‐ assessment of effects by sex/gender NOT present	No ‐ assessment of effects across any other PROGRESS characteristics? For example, socioeconomic status	RCT
			Mobility	Cost‐effectiveness						
			Contribution	Safety						
			Build and maintain relationships	Health service utilization						
Melin (1995)										
			Basic needs	Cost‐effectiveness	Residential home/apartment	Usual care		No ‐ assessment of effects by sex/gender NOT present	No ‐ assessment of effects across any other PROGRESS characteristics? For example, socioeconomic status	RCT
			Mobility	Health service utilization						
Melis (2008)										
		Mental functions	Basic needs	Cost‐effectiveness	Residential home/apartment	Usual care		No ‐ assessment of effects by sex/gender NOT present	No ‐ assessment of effects across any other PROGRESS characteristics? For example, socioeconomic status	RCT
					Assisted living					
Melis (2008)										
		Mental functions	Basic needs		Residential home/apartment	Usual care		No ‐ assessment of effects by sex/gender NOT present	No ‐ assessment of effects across any other PROGRESS characteristics? For example, socioeconomic status	RCT
			Quality of life							
			Mobility							
Mihalko (1996)										
		Mental functions	Basic needs	Satisfaction of older adult	Assisted living	Usual care		No ‐ assessment of effects by sex/gender NOT present	No ‐ assessment of effects across any other PROGRESS characteristics? For example, socioeconomic status	RCT
		Neuromusculoskeletal function								
Miller (2005)										
				Cost‐effectiveness	Residential home/apartment	Usual care		No ‐ assessment of effects by sex/gender NOT present	No ‐ assessment of effects across any other PROGRESS characteristics? For example, socioeconomic status	RCT
Milte (2016)										
			Quality of life	Cost‐effectiveness	Residential home/apartment	Other		No ‐ assessment of effects by sex/gender NOT present	No ‐ assessment of effects across any other PROGRESS characteristics? For example, socioeconomic status	RCT
							
Mitchell (2005)										
		Sensory functions and pain		Satisfaction of older adult	Residential home/apartment	Usual care		No ‐ assessment of effects by sex/gender NOT present	No ‐ assessment of effects across any other PROGRESS characteristics? For example, socioeconomic status	RCT
		Neuromusculoskeletal function		Health service utilization						
Mohide (1990)										
				Caregiver outcomes	Residential home/apartment	Other		No ‐ assessment of effects by sex/gender NOT present	No ‐ assessment of effects across any other PROGRESS characteristics? For example, socioeconomic status	RCT
				Health service utilization	Long‐term care					
Molassiotis (2009)										
		Mental functions	Quality of life	Health service utilization	Residential home/apartment	Usual care		No ‐ assessment of effects by sex/gender NOT present	No ‐ assessment of effects across any other PROGRESS characteristics? For example, socioeconomic status	RCT
		Sensory functions and pain								
Moller (2014)										
		Sensory functions and pain	Mobility		Residential home/apartment			No ‐ assessment of effects by sex/gender NOT present	No ‐ assessment of effects across any other PROGRESS characteristics? For example, socioeconomic status	RCT
						
Montgomery (2003)										
		Mental functions	Basic needs	Health service utilization	Residential home/apartment	Usual care		No ‐ assessment of effects by sex/gender NOT present	No ‐ assessment of effects across any other PROGRESS characteristics? For example, socioeconomic status	RCT
			Mobility							
Morris (2017)										
		Neuromusculoskeletal function	Quality of life	Cost (e.g., out of pocket)	Residential home/apartment	Usual care		No ‐ assessment of effects by sex/gender NOT present	No ‐ assessment of effects across any other PROGRESS characteristics? For example, socioeconomic status	RCT
				Cost‐effectiveness						
		Falls					
Mortensen (2016)										
		Mental functions	Basic needs		Residential home/apartment	Usual care		No ‐ assessment of effects by sex/gender NOT present	No ‐ assessment of effects across any other PROGRESS characteristics? For example, socioeconomic status	RCT
		Neuromusculoskeletal function	Contribution							
Mulrow (1994)										
		Mental functions	Basic needs	Falls	Assisted living	Usual care		No ‐ assessment of effects by sex/gender NOT present	No ‐ assessment of effects across any other PROGRESS characteristics? For example, socioeconomic status	RCT
		Neuromusculoskeletal function								
Murphy (2005)										
		Functions of the cardiovascular, haematological, immunological and respiratory systems	Quality of life		Residential home/apartment	Usual care		No ‐ assessment of effects by sex/gender NOT present	No ‐ assessment of effects across any other PROGRESS characteristics? For example, socioeconomic status	RCT
		Neuromusculoskeletal function	Mobility							
Naunton (2003)										
				Satisfaction of older adult	Residential home/apartment	Usual care		No ‐ assessment of effects by sex/gender NOT present	No ‐ assessment of effects across any other PROGRESS characteristics? For example, socioeconomic status	RCT
				Health service utilization						
Naylor (1999)										
		Mental functions	Basic needs	Cost (e.g., out of pocket)	Residential home/apartment	Usual care		No ‐ assessment of effects by sex/gender NOT present	No ‐ assessment of effects across any other PROGRESS characteristics? For example, socioeconomic status	RCT
				Satisfaction of older adult						
				Health service utilization						
Naylor (2004)										
			Basic needs	Cost (e.g., out of pocket)	Residential home/apartment	Usual care		No ‐ assessment of effects by sex/gender NOT present	No ‐ assessment of effects across any other PROGRESS characteristics? For example, socioeconomic status	RCT
			Quality of life	Satisfaction of older adult						
				Health service utilization						
Naylor (2004)										
			Basic needs	Satisfaction of older adult	Residential home/apartment	Usual care		No ‐ assessment of effects by sex/gender NOT present	No ‐ assessment of effects across any other PROGRESS characteristics? For example, socioeconomic status	RCT
			Quality of life	Health service utilization						
Nazareth (2001)										
			Quality of life	Satisfaction of older adult	Residential home/apartment	Usual care		No ‐ assessment of effects by sex/gender NOT present	No ‐ assessment of effects across any other PROGRESS characteristics? For example, socioeconomic status	RCT
				Adherence						
				Health service utilization						
Nct (2005)										
			Quality of life	Health service utilization	Residential home/apartment	Usual care		No ‐ assessment of effects by sex/gender NOT present	No ‐ assessment of effects across any other PROGRESS characteristics? For example, socioeconomic status	RCT
Nct (2006)										
			Basic needs		Residential home/apartment	Usual care		No ‐ assessment of effects by sex/gender NOT present	No ‐ assessment of effects across any other PROGRESS characteristics? For example, socioeconomic status	RCT
Nct (2011)										
			Quality of life		Residential home/apartment	Usual care		No ‐ assessment of effects by sex/gender NOT present	No ‐ assessment of effects across any other PROGRESS characteristics? For example, socioeconomic status	RCT
Nct (2011)										
		Functions of the cardiovascular, haematological, immunological and respiratory systems	Quality of life	Health service utilization	Residential home/apartment	Usual care		No ‐ assessment of effects by sex/gender NOT present	No ‐ assessment of effects across any other PROGRESS characteristics? For example, socioeconomic status	RCT
		Neuromusculoskeletal function	Mobility	Falls						
Nct (2012)										
		Mental functions		Falls	Residential home/apartment	Usual care		No ‐ assessment of effects by sex/gender NOT present	No ‐ assessment of effects across any other PROGRESS characteristics? For example, socioeconomic status	RCT
Nct (2013)										
		Mental functions	Basic needs		Residential home/apartment	Usual care		No ‐ assessment of effects by sex/gender NOT present	No ‐ assessment of effects across any other PROGRESS characteristics? For example, socioeconomic status	RCT
		Functions of the digestive, metabolic and endocrine systems	Quality of life							
		Neuromusculoskeletal function	Build and maintain relationships							
Nct (2014)										
		Sensory functions and pain	Basic needs		Assisted living	Usual care		No ‐ assessment of effects by sex/gender NOT present	No ‐ assessment of effects across any other PROGRESS characteristics? For example, socioeconomic status	RCT
		Neuromusculoskeletal function	Quality of life							
Nct (2014)										
		Mental functions	Quality of life	Cost (e.g., out of pocket)	Residential home/apartment	Usual care		No ‐ assessment of effects by sex/gender NOT present	No ‐ assessment of effects across any other PROGRESS characteristics? For example, socioeconomic status	RCT
		Neuromusculoskeletal function		Caregiver outcomes						
				Health service utilization						
Nct (2014)										
				Health service utilization	Residential home/apartment	Usual care		No ‐ assessment of effects by sex/gender NOT present	No ‐ assessment of effects across any other PROGRESS characteristics? For example, socioeconomic status	RCT
Nct (2015)										
			Basic needs	Falls	Residential home/apartment	Usual care		No ‐ assessment of effects by sex/gender NOT present	No ‐ assessment of effects across any other PROGRESS characteristics? For example, socioeconomic status	RCT
			Mobility							
Nct (2017)										
		Mental functions	Basic needs	Health service utilization	Residential home/apartment	Usual care		No ‐ assessment of effects by sex/gender NOT present	No ‐ assessment of effects across any other PROGRESS characteristics? For example, socioeconomic status	RCT
		Functions of the cardiovascular, haematological, immunological and respiratory systems	Quality of life							
		Functions of the digestive, metabolic and endocrine systems	Mobility							
			Learning, grow and make decisions							
			Build and maintain relationships							
Nct (2017)										
		Mental functions	Basic needs	Falls	Residential home/apartment	Other		No ‐ assessment of effects by sex/gender NOT present	No ‐ assessment of effects across any other PROGRESS characteristics? For example, socioeconomic status	RCT
		Sensory functions and pain	Mobility							
		Neuromusculoskeletal function								
Nct (2017)										
		Mental functions	Mobility	Falls	Residential home/apartment	Usual care		No ‐ assessment of effects by sex/gender NOT present	No ‐ assessment of effects across any other PROGRESS characteristics? For example, socioeconomic status	RCT
Nct (2017)										
		Mental functions	Quality of life	Caregiver outcomes	Residential home/apartment	Usual care		No ‐ assessment of effects by sex/gender NOT present	No ‐ assessment of effects across any other PROGRESS characteristics? For example, socioeconomic status	RCT
			Contribution		Long‐term care					
			Build and maintain relationships							
Nct (2018)										
		Mental functions	Quality of life	Safety	Residential home/apartment	Usual care		No ‐ assessment of effects by sex/gender NOT present	No ‐ assessment of effects across any other PROGRESS characteristics? For example, socioeconomic status	RCT
		Neuromusculoskeletal function								
Nct (2018)										
		Neuromusculoskeletal function	Mobility	Adherence	Residential home/apartment	Other		No ‐ assessment of effects by sex/gender NOT present	No ‐ assessment of effects across any other PROGRESS characteristics? For example, socioeconomic status	RCT
				Cost‐effectiveness						
				Health service utilization						
				Falls						
Neumann (2017)										
			Basic needs		Residential home/apartment	Usual care		No ‐ assessment of effects by sex/gender NOT present	No ‐ assessment of effects across any other PROGRESS characteristics? For example, socioeconomic status	RCT
Nicolaides‐Bouman (2004)										
		Mental functions	Basic needs	Health service utilization	Residential home/apartment	Usual care		No ‐ assessment of effects by sex/gender NOT present	No ‐ assessment of effects across any other PROGRESS characteristics? For example, socioeconomic status	RCT
			Quality of life							
Nielsen (1972)										
				Health service utilization	Residential home/apartment	Usual care		No ‐ assessment of effects by sex/gender NOT present	No ‐ assessment of effects across any other PROGRESS characteristics? For example, socioeconomic status	RCT
Nikolaus (1999)										
			Basic needs	Cost (e.g., out of pocket)	Residential home/apartment	Usual care		No ‐ assessment of effects by sex/gender NOT present	No ‐ assessment of effects across any other PROGRESS characteristics? For example, socioeconomic status	RCT
			Quality of life	Satisfaction of older adult	Long‐term care					
				Health service utilization						
Nikolaus (2003)										
				Falls	Residential home/apartment	Usual care		No ‐ assessment of effects by sex/gender NOT present	No ‐ assessment of effects across any other PROGRESS characteristics? For example, socioeconomic status	RCT
Nobili (2004)										
		Mental functions		Caregiver outcomes	Residential home/apartment	Usual care		No ‐ assessment of effects by sex/gender NOT present	No ‐ assessment of effects across any other PROGRESS characteristics? For example, socioeconomic status	RCT
							
						
Nowalk (2001)										
		Mental functions	Mobility	Falls	Long‐term care	Other		No ‐ assessment of effects by sex/gender NOT present	No ‐ assessment of effects across any other PROGRESS characteristics? For example, socioeconomic status	RCT
		Neuromusculoskeletal function								
Oerkild (2011)										
		Mental functions	Quality of life		Residential home/apartment	Usual care		No ‐ assessment of effects by sex/gender NOT present	No ‐ assessment of effects across any other PROGRESS characteristics? For example, socioeconomic status	RCT
		Functions of the cardiovascular, haematological, immunological and respiratory systems	Mobility							
Oerkild (2012)										
		Mental functions	Basic needs		Residential home/apartment	Usual care		No ‐ assessment of effects by sex/gender NOT present	No ‐ assessment of effects across any other PROGRESS characteristics? For example, socioeconomic status	RCT
		Functions of the cardiovascular, haematological, immunological and respiratory systems	Quality of life							
		Functions of the digestive, metabolic and endocrine systems	Mobility							
Olaleye (2014)										
		Neuromusculoskeletal function	Basic needs		Residential home/apartment	Other		No ‐ assessment of effects by sex/gender NOT present	No ‐ assessment of effects across any other PROGRESS characteristics? For example, socioeconomic status	RCT
			Mobility							
Olesen (2014)										
				Adherence	Residential home/apartment	Usual care		No ‐ assessment of effects by sex/gender NOT present	No ‐ assessment of effects across any other PROGRESS characteristics? For example, socioeconomic status	RCT
				Health service utilization						
Olson (2011)										
		Neuromusculoskeletal function		Falls	Residential home/apartment	Usual care		No ‐ assessment of effects by sex/gender NOT present	No ‐ assessment of effects across any other PROGRESS characteristics? For example, socioeconomic status	RCT
Oosting (2012)										
		Sensory functions and pain	Basic needs	Satisfaction of older adult	Residential home/apartment	Usual care		No ‐ assessment of effects by sex/gender NOT present	No ‐ assessment of effects across any other PROGRESS characteristics? For example, socioeconomic status	RCT
		Neuromusculoskeletal function	Quality of life	Adherence						
		Mobility	Safety							
Orrell (2017)										
		Mental functions	Basic needs	Cost (e.g., out of pocket)	Residential home/apartment	Usual care		No ‐ assessment of effects by sex/gender NOT present	No ‐ assessment of effects across any other PROGRESS characteristics? For example, socioeconomic status	RCT
			Quality of life	Cost‐effectiveness						
			Communication	Caregiver outcomes						
Ouslander (2005)										
		Mental functions	Basic needs		Assisted living	Usual care		No ‐ assessment of effects by sex/gender NOT present	No ‐ assessment of effects across any other PROGRESS characteristics? For example, socioeconomic status	RCT
		Genitourinary and reproductive functions	Mobility							
		Neuromusculoskeletal function								
Özdemir (2001)										
		Mental functions	Basic needs		Residential home/apartment	Usual care		No ‐ assessment of effects by sex/gender NOT present	No ‐ assessment of effects across any other PROGRESS characteristics? For example, socioeconomic status	RCT
		Neuromusculoskeletal function								
Padala (2017)										
		Mental functions	Basic needs		Residential home/apartment	Usual care		No ‐ assessment of effects by sex/gender NOT present	No ‐ assessment of effects across any other PROGRESS characteristics? For example, socioeconomic status	RCT
		Neuromusculoskeletal function	Quality of life							
Padula (2009)										
		Mental functions	Basic needs		Residential home/apartment	Other		No ‐ assessment of effects by sex/gender NOT present	No ‐ assessment of effects across any other PROGRESS characteristics? For example, socioeconomic status	RCT
		Functions of the cardiovascular, haematological, immunological and respiratory systems	Quality of life							
		Neuromusculoskeletal function							
Papaioannou (2003)										
		Mental functions	Quality of life		Residential home/apartment	Usual care		No ‐ assessment of effects by sex/gender NOT present	No ‐ assessment of effects across any other PROGRESS characteristics? For example, socioeconomic status	RCT
		Neuromusculoskeletal function	Mobility							
Pardessus (2002)										
			Basic needs	Health service utilization	Residential home/apartment	Other		No ‐ assessment of effects by sex/gender NOT present	No ‐ assessment of effects across any other PROGRESS characteristics? For example, socioeconomic status	RCT
				Falls						
Parker (2009)										
		Mental functions	Basic needs		Assisted living	Other		No ‐ assessment of effects by sex/gender NOT present	No ‐ assessment of effects across any other PROGRESS characteristics? For example, socioeconomic status	RCT
			Mobility							
Parker (2011)										
		Mental functions	Basic needs	Caregiver outcomes	Residential home/apartment	Usual care		No ‐ assessment of effects by sex/gender NOT present	No ‐ assessment of effects across any other PROGRESS characteristics? For example, socioeconomic status	RCT
				Health service utilization						
Parsons et al. (2013)										
		Neuromusculoskeletal function	Basic needs		Residential home/apartment	Usual care		No ‐ assessment of effects by sex/gender NOT present	No ‐ assessment of effects across any other PROGRESS characteristics? For example, socioeconomic status	RCT
Parsons (2017)										
		Mental functions	Basic needs	Caregiver outcomes	Residential home/apartment	Usual care		No ‐ assessment of effects by sex/gender NOT present	No ‐ assessment of effects across any other PROGRESS characteristics? For example, socioeconomic status	RCT
		Sensory functions and pain	Quality of life	Health service utilization						
										
Patterson (2009)										
		Mental functions	Quality of life		Residential home/apartment	Other		No ‐ assessment of effects by sex/gender NOT present	No ‐ assessment of effects across any other PROGRESS characteristics? For example, socioeconomic status	RCT
Pedersen (2016)										
		Mental functions	Basic needs	Health service utilization	Residential home/apartment	Usual care		No ‐ assessment of effects by sex/gender NOT present	No ‐ assessment of effects across any other PROGRESS characteristics? For example, socioeconomic status	RCT
			Quality of life							
Peeters (2007)										
			Basic needs	Falls	Residential home/apartment	Usual care		No ‐ assessment of effects by sex/gender NOT present	No ‐ assessment of effects across any other PROGRESS characteristics? For example, socioeconomic status	RCT
			Quality of life							
Pizzi (2014)										
				Cost‐effectiveness	Residential home/apartment	Other	Race, ethnicity, culture, language	No ‐ assessment of effects by sex/gender NOT present	No ‐ assessment of effects across any other PROGRESS characteristics? For example, socioeconomic status	RCT
Portegijs (2013)										
		Sensory functions and pain	Mobility		Residential home/apartment	Usual care		No ‐ assessment of effects by sex/gender NOT present	No ‐ assessment of effects across any other PROGRESS characteristics? For example, socioeconomic status	RCT
Prick (2015)										
		Mental functions		Adherence	Residential home/apartment	Usual care		No ‐ assessment of effects by sex/gender NOT present	No ‐ assessment of effects across any other PROGRESS characteristics? For example, socioeconomic status	RCT
				Caregiver outcomes		Other				
Pröfener (2016)										
		Mental functions		Access	Residential home/apartment	Usual care		No ‐ assessment of effects by sex/gender NOT present	No ‐ assessment of effects across any other PROGRESS characteristics? For example, socioeconomic status	RCT
Radwany (2014)										
		Mental functions	Quality of life	Health service utilization	Residential home/apartment	Usual care		No ‐ assessment of effects by sex/gender NOT present	No ‐ assessment of effects across any other PROGRESS characteristics? For example, socioeconomic status	RCT
Rasmussen (2016)										
		Mental functions	Basic needs	Cost‐effectiveness	Residential home/apartment	Usual care		No ‐ assessment of effects by sex/gender NOT present	No ‐ assessment of effects across any other PROGRESS characteristics? For example, socioeconomic status	RCT
		Functions of the digestive, metabolic and endocrine systems	Quality of life							
		Neuromusculoskeletal function	Mobility							
Ray (1997)										
				Falls	Assisted living	Other		No ‐ assessment of effects by sex/gender NOT present	No ‐ assessment of effects across any other PROGRESS characteristics? For example, socioeconomic status	RCT
Reckrey (2018)										
		Mental functions	Quality of life	Satisfaction of older adult	Residential home/apartment	Usual care		No ‐ assessment of effects by sex/gender NOT present	No ‐ assessment of effects across any other PROGRESS characteristics? For example, socioeconomic status	RCT
		Sensory functions and pain		Health service utilization						
		Functions of the cardiovascular, haematological, immunological and respiratory systems								
Reeves (2004)										
		Mental functions	Basic needs	Health service utilization	Residential home/apartment	Usual care		No ‐ assessment of effects by sex/gender NOT present	No ‐ assessment of effects across any other PROGRESS characteristics? For example, socioeconomic status	RCT
		Sensory functions and pain	Quality of life							
Regan (2017)										
		Mental functions	Quality of life	Satisfaction of older adult	Residential home/apartment	Usual care		No ‐ assessment of effects by sex/gender NOT present	No ‐ assessment of effects across any other PROGRESS characteristics? For example, socioeconomic status	RCT
				Caregiver outcomes						
Resnick (2009)										
		Neuromusculoskeletal function	Basic needs		Residential home/apartment	Other		No ‐ assessment of effects by sex/gender NOT present	No ‐ assessment of effects across any other PROGRESS characteristics? For example, socioeconomic status	RCT
			Quality of life							
			Mobility							
Richards (1998)										
		Neuromusculoskeletal function	Basic needs	Satisfaction of older adult	Residential home/apartment	Usual care		No ‐ assessment of effects by sex/gender NOT present	No ‐ assessment of effects across any other PROGRESS characteristics? For example, socioeconomic status	RCT
			Quality of life	Access						
			Contribution							
Robertson et al. (2001)										
				Cost‐effectiveness	Residential home/apartment	Other		No ‐ assessment of effects by sex/gender NOT present	No ‐ assessment of effects across any other PROGRESS characteristics? For example, socioeconomic status	RCT
				Falls						
Roderick (2001)										
		Mental functions	Basic needs	Cost (e.g., out of pocket)	Residential home/apartment	Usual care		No ‐ assessment of effects by sex/gender NOT present	No ‐ assessment of effects across any other PROGRESS characteristics? For example, socioeconomic status	RCT
			Quality of life	Cost‐effectiveness						
			Mobility							
			Contribution							
Rosendahl (2006)										
		Mental functions		Falls	Residential home/apartment	Usual care		No ‐ assessment of effects by sex/gender NOT present	No ‐ assessment of effects across any other PROGRESS characteristics? For example, socioeconomic status	RCT
		Neuromusculoskeletal function								
Rosstad (2017)										
			Basic needs	Health service utilization	Residential home/apartment	Usual care		No ‐ assessment of effects by sex/gender NOT present	No ‐ assessment of effects across any other PROGRESS characteristics? For example, socioeconomic status	RCT
			Quality of life							
Rossum (1993)										
		Mental functions	Basic needs	Cost‐effectiveness	Residential home/apartment	Usual care		No ‐ assessment of effects by sex/gender NOT present	No ‐ assessment of effects across any other PROGRESS characteristics? For example, socioeconomic status	RCT
				Health service utilization						
Rubenstein (1994)										
		Mental functions	Basic needs	Health service utilization	Residential home/apartment			No ‐ assessment of effects by sex/gender NOT present	No ‐ assessment of effects across any other PROGRESS characteristics? For example, socioeconomic status	RCT
					Independent living					
Runciman (1996)										
			Basic needs	Satisfaction of older adult	Residential home/apartment	Usual care		No ‐ assessment of effects by sex/gender NOT present	No ‐ assessment of effects across any other PROGRESS characteristics? For example, socioeconomic status	RCT
				Health service utilization						
Ryan (2006)										
		Mental functions	Basic needs		Residential home/apartment	Other		No ‐ assessment of effects by sex/gender NOT present	No ‐ assessment of effects across any other PROGRESS characteristics? For example, socioeconomic status	RCT
			Quality of life							
			Build and maintain relationships							
Rytter (2010)										
			Basic needs	Cost (e.g., out of pocket)	Residential home/apartment	Usual care		No ‐ assessment of effects by sex/gender NOT present	No ‐ assessment of effects across any other PROGRESS characteristics? For example, socioeconomic status	RCT
				Satisfaction of older adult						
				Cost‐effectiveness						
				Health service utilization						
Sackley (2007)										
			Mobility		Assisted living	Usual care		No ‐ assessment of effects by sex/gender NOT present	No ‐ assessment of effects across any other PROGRESS characteristics? For example, socioeconomic status	RCT
Sackley (2009)										
		Mental functions	Basic needs		Assisted living	Usual care		No ‐ assessment of effects by sex/gender NOT present	No ‐ assessment of effects across any other PROGRESS characteristics? For example, socioeconomic status	RCT
			Mobility							
Sackley (2015)										
	Adaptations to physical environment	Mental functions	Basic needs	Cost‐effectiveness	Assisted living	Usual care		No ‐ assessment of effects by sex/gender NOT present	No ‐ assessment of effects across any other PROGRESS characteristics? For example, socioeconomic status	RCT
			Quality of life	Safety						
			Mobility	Falls						
Sahlen (2016)										
			Quality of life	Cost‐effectiveness	Residential home/apartment	Usual care		No ‐ assessment of effects by sex/gender NOT present	No ‐ assessment of effects across any other PROGRESS characteristics? For example, socioeconomic status	RCT
Salminen (2009)										
			Basic needs	Health service utilization	Residential home/apartment	Other		No ‐ assessment of effects by sex/gender NOT present	No ‐ assessment of effects across any other PROGRESS characteristics? For example, socioeconomic status	RCT
			Quality of life	Falls	Assisted living					
			Mobility							
Salpakoski (2014)										
		Sensory functions and pain	Mobility		Residential home/apartment	Usual care		No ‐ assessment of effects by sex/gender NOT present	No ‐ assessment of effects across any other PROGRESS characteristics? For example, socioeconomic status	RCT
		Neuromusculoskeletal function								
Samus (2014)										
		Mental functions	Quality of life		Residential home/apartment	Other		No ‐ assessment of effects by sex/gender NOT present	No ‐ assessment of effects across any other PROGRESS characteristics? For example, socioeconomic status	RCT
Sandberg (2015)										
		Neuromusculoskeletal function	Basic needs	Cost (e.g., out of pocket)	Residential home/apartment	Usual care		No ‐ assessment of effects by sex/gender NOT present	No ‐ assessment of effects across any other PROGRESS characteristics? For example, socioeconomic status	RCT
			Quality of life	Satisfaction of older adult	Assisted living					
			Cost‐effectiveness					
Sandberg (2015)										
				Health service utilization	Residential home/apartment	Usual care		No ‐ assessment of effects by sex/gender NOT present	No ‐ assessment of effects across any other PROGRESS characteristics? For example, socioeconomic status	RCT
Sanford (2006)										
			Basic needs		Residential home/apartment	Other		No ‐ assessment of effects by sex/gender NOT present	No ‐ assessment of effects across any other PROGRESS characteristics? For example, socioeconomic status	RCT
Schnelle (1996)										
		Neuromusculoskeletal function	Mobility	Safety	Assisted living	Usual care		No ‐ assessment of effects by sex/gender NOT present	No ‐ assessment of effects across any other PROGRESS characteristics? For example, socioeconomic status	RCT
Schnelle (2010)										
		Mental functions	Mobility		Assisted living	Usual care		No ‐ assessment of effects by sex/gender NOT present	No ‐ assessment of effects across any other PROGRESS characteristics? For example, socioeconomic status	RCT
		Functions of the digestive, metabolic and endocrine systems								
		Genitourinary and reproductive functions								
		Neuromusculoskeletal function								
Seidl (2015)										
		Sensory functions and pain	Quality of life	Cost‐effectiveness	Residential home/apartment	Usual care		No ‐ assessment of effects by sex/gender NOT present	No ‐ assessment of effects across any other PROGRESS characteristics? For example, socioeconomic status	RCT
Senior (2014)										
		Mental functions	Basic needs	Caregiver outcomes	Residential home/apartment	Usual care		No ‐ assessment of effects by sex/gender NOT present	No ‐ assessment of effects across any other PROGRESS characteristics? For example, socioeconomic status	RCT
		Sensory functions and pain	Quality of life	Health service utilization	Independent living					
							
Serra‐Rexach (2011)										
		Neuromusculoskeletal function	Mobility		Assisted living	Usual care		No ‐ assessment of effects by sex/gender NOT present	No ‐ assessment of effects across any other PROGRESS characteristics? For example, socioeconomic status	RCT
Sheffield (2013)										
	Adaptations to physical environment	Mental functions	Basic needs	Cost‐effectiveness	Residential home/apartment	Usual care		No ‐ assessment of effects by sex/gender NOT present	No ‐ assessment of effects across any other PROGRESS characteristics? For example, socioeconomic status	RCT
			Quality of life	Falls						
Shepperd and Iliffe (1998)										
				Cost‐effectiveness	Residential home/apartment	Usual care		No ‐ assessment of effects by sex/gender NOT present	No ‐ assessment of effects across any other PROGRESS characteristics? For example, socioeconomic status	RCT
				Health service utilization						
Shepperd and Iliffe (1998)										
			Basic needs	Satisfaction of older adult	Residential home/apartment	Usual care		No ‐ assessment of effects by sex/gender NOT present	No ‐ assessment of effects across any other PROGRESS characteristics? For example, socioeconomic status	RCT
			Quality of life	Health service utilization						
Shepperd (2017)										
		Mental functions	Basic needs	Satisfaction of older adult	Independent living	Usual care		No ‐ assessment of effects by sex/gender NOT present	No ‐ assessment of effects across any other PROGRESS characteristics? For example, socioeconomic status	RCT
				Health service utilization	Assisted living					
Sherman (2016)										
		Mental functions	Basic needs	Satisfaction of older adult	Residential home/apartment	Usual care		No ‐ assessment of effects by sex/gender NOT present	No ‐ assessment of effects across any other PROGRESS characteristics? For example, socioeconomic status	RCT
		Sensory functions and pain	Mobility							
		Functions of the cardiovascular, haematological, immunological and respiratory systems	Communication							
		Functions of the digestive, metabolic and endocrine systems								
		Integumentary system								
Sherrington (2015)										
		Neuromusculoskeletal function	Basic needs	Adherence	Residential home/apartment	Usual care		No ‐ assessment of effects by sex/gender NOT present	No ‐ assessment of effects across any other PROGRESS characteristics? For example, socioeconomic status	RCT
			Quality of life	Safety						
			Mobility	Health service utilization						
			Contribution	Falls						
Sherrington (2016)										
		Mental functions	Basic needs	Cost‐effectiveness	Residential home/apartment	Usual care		No ‐ assessment of effects by sex/gender NOT present	No ‐ assessment of effects across any other PROGRESS characteristics? For example, socioeconomic status	RCT
		Sensory functions and pain	Quality of life	Safety						
		Neuromusculoskeletal function	Mobility	Health service utilization						
		Falls					
Shyu (2008)										
				Satisfaction of older adult	Residential home/apartment	Usual care		No ‐ assessment of effects by sex/gender NOT present	No ‐ assessment of effects across any other PROGRESS characteristics? For example, socioeconomic status	RCT
				Caregiver outcomes						
Shyu (2016)										
			Basic needs	Health service utilization	Residential home/apartment	Usual care		No ‐ assessment of effects by sex/gender NOT present	No ‐ assessment of effects across any other PROGRESS characteristics? For example, socioeconomic status	RCT
Siggeirsdottir (2005)										
		Sensory functions and pain	Quality of life		Residential home/apartment	Usual care		No ‐ assessment of effects by sex/gender NOT present	No ‐ assessment of effects across any other PROGRESS characteristics? For example, socioeconomic status	RCT
		Neuromusculoskeletal function								
Simmons (2002)										
		Sensory functions and pain	Mobility		Assisted living	Other		No ‐ assessment of effects by sex/gender NOT present	No ‐ assessment of effects across any other PROGRESS characteristics? For example, socioeconomic status	RCT
Simmons (2005)										
		Genitourinary and reproductive functions	Quality of life	Satisfaction of older adult	Long‐term care	Usual care		No ‐ assessment of effects by sex/gender NOT present	No ‐ assessment of effects across any other PROGRESS characteristics? For example, socioeconomic status	RCT
			Mobility							
Sloane (2004)										
		Mental functions	Basic needs		Assisted living	Usual care		No ‐ assessment of effects by sex/gender NOT present	No ‐ assessment of effects across any other PROGRESS characteristics? For example, socioeconomic status	RCT
		Integumentary system								
Steele (2008)										
			Basic needs		Residential home/apartment	Other		No ‐ assessment of effects by sex/gender NOT present	No ‐ assessment of effects across any other PROGRESS characteristics? For example, socioeconomic status	RCT
			Mobility							
Steinberg (2009)										
		Mental functions	Basic needs	Caregiver outcomes	Residential home/apartment	Other		No ‐ assessment of effects by sex/gender NOT present	No ‐ assessment of effects across any other PROGRESS characteristics? For example, socioeconomic status	RCT
		Neuromusculoskeletal function	Quality of life							
			Mobility							
Stelmack et al. (2007)										
		Mental functions	Quality of life		Residential home/apartment	Usual care		No ‐ assessment of effects by sex/gender NOT present	No ‐ assessment of effects across any other PROGRESS characteristics? For example, socioeconomic status	RCT
		Sensory functions and pain	Mobility							
Stevens (2001)										
	Adaptations to physical environment			Falls	Residential home/apartment	Other		No ‐ assessment of effects by sex/gender NOT present	No ‐ assessment of effects across any other PROGRESS characteristics? For example, socioeconomic status	RCT
Stevens‐Lapsley (2016)										
		Neuromusculoskeletal function	Mobility	Health service utilization	Residential home/apartment	Usual care		No ‐ assessment of effects by sex/gender NOT present	No ‐ assessment of effects across any other PROGRESS characteristics? For example, socioeconomic status	RCT
Stewart et al. (2005)										
		Mental functions	Quality of life	Caregiver outcomes	Residential home/apartment	Other		No ‐ assessment of effects by sex/gender NOT present	No ‐ assessment of effects across any other PROGRESS characteristics? For example, socioeconomic status	RCT
				Health service utilization						
Stewart (2012)										
			Quality of life	Cost (e.g., out of pocket)	Residential home/apartment	Other		No ‐ assessment of effects by sex/gender NOT present	No ‐ assessment of effects across any other PROGRESS characteristics? For example, socioeconomic status	RCT
				Cost‐effectiveness						
				Health service utilization						
Stuck et al. (1995)										
				Cost‐effectiveness	Residential home/apartment	Other		No ‐ assessment of effects by sex/gender NOT present	No ‐ assessment of effects across any other PROGRESS characteristics? For example, socioeconomic status	RCT
						
Stuck et al. (1995)										
			Basic needs	Cost‐effectiveness	Residential home/apartment	Usual care		No ‐ assessment of effects by sex/gender NOT present	No ‐ assessment of effects across any other PROGRESS characteristics? For example, socioeconomic status	RCT
				Health service utilization						
Stuck (2000)										
		Mental functions	Basic needs	Cost‐effectiveness	Residential home/apartment	Usual care		No ‐ assessment of effects by sex/gender NOT present	No ‐ assessment of effects across any other PROGRESS characteristics? For example, socioeconomic status	RCT
		Neuromusculoskeletal function		Health service utilization						
Suominen (2015)										
		Functions of the digestive, metabolic and endocrine systems	Quality of life	Falls	Residential home/apartment	Usual care		No ‐ assessment of effects by sex/gender NOT present	No ‐ assessment of effects across any other PROGRESS characteristics? For example, socioeconomic status	RCT
Suttanon (2013)										
		Neuromusculoskeletal function	Quality of life	Adherence	Residential home/apartment	Other		No ‐ assessment of effects by sex/gender NOT present	No ‐ assessment of effects across any other PROGRESS characteristics? For example, socioeconomic status	RCT
			Mobility	Caregiver outcomes						
				Falls						
Szanton (2014)										
		Neuromusculoskeletal function	Basic needs	Cost‐effectiveness	Residential home/apartment	Other	Socioeconomic status	No ‐ assessment of effects by sex/gender NOT present	No ‐ assessment of effects across any other PROGRESS characteristics? For example, socioeconomic status	RCT
			Mobility	Health service utilization						
Talley (2017)										
		Genitourinary and reproductive functions	Basic needs		Residential home/apartment	Usual care		No ‐ assessment of effects by sex/gender NOT present	No ‐ assessment of effects across any other PROGRESS characteristics? For example, socioeconomic status	RCT
		Neuromusculoskeletal function	Mobility							
Taube (2017)										
		Mental functions	Quality of life	Satisfaction of older adult	Residential home/apartment	Usual care		No ‐ assessment of effects by sex/gender NOT present	No ‐ assessment of effects across any other PROGRESS characteristics? For example, socioeconomic status	RCT
Thomas (2016)										
		Mental functions			Residential home/apartment	Usual care	Social capital	No ‐ assessment of effects by sex/gender NOT present	No ‐ assessment of effects across any other PROGRESS characteristics? For example, socioeconomic status	RCT
Thomas (2018)										
				Falls	Residential home/apartment	Other		No ‐ assessment of effects by sex/gender NOT present	No ‐ assessment of effects across any other PROGRESS characteristics? For example, socioeconomic status	RCT
Thygesen (2015)										
				Health service utilization	Residential home/apartment	Usual care		No ‐ assessment of effects by sex/gender NOT present	No ‐ assessment of effects across any other PROGRESS characteristics? For example, socioeconomic status	RCT
Tibaldi (2004)										
		Mental functions		Caregiver outcomes	Residential home/apartment	Usual care		No ‐ assessment of effects by sex/gender NOT present	No ‐ assessment of effects across any other PROGRESS characteristics? For example, socioeconomic status	RCT
Tibaldi (2009)										
		Mental functions	Basic needs	Health service utilization	Residential home/apartment	Usual care		No ‐ assessment of effects by sex/gender NOT present	No ‐ assessment of effects across any other PROGRESS characteristics? For example, socioeconomic status	RCT
Tinetti (1999)										
			Basic needs		Residential home/apartment	Usual care		No ‐ assessment of effects by sex/gender NOT present	No ‐ assessment of effects across any other PROGRESS characteristics? For example, socioeconomic status	RCT
			Contribution							
			Build and maintain relationships							
Toots (2017)										
		Mental functions			Assisted living	Other		No ‐ assessment of effects by sex/gender NOT present	No ‐ assessment of effects across any other PROGRESS characteristics? For example, socioeconomic status	RCT
Townsend (1988)										
			Basic needs	Cost‐effectiveness	Residential home/apartment	Usual care		No ‐ assessment of effects by sex/gender NOT present	No ‐ assessment of effects across any other PROGRESS characteristics? For example, socioeconomic status	RCT
				Health service utilization						
Tsaih (2011)										
		Neuromusculoskeletal function	Basic needs		Long‐term care	Usual care		No ‐ assessment of effects by sex/gender NOT present	No ‐ assessment of effects across any other PROGRESS characteristics? For example, socioeconomic status	RCT
			Mobility		Assisted living					
Tseng (2016)										
		Mental functions	Quality of life		Residential home/apartment	Usual care		No ‐ assessment of effects by sex/gender NOT present	No ‐ assessment of effects across any other PROGRESS characteristics? For example, socioeconomic status	RCT
		Sensory functions and pain								
		Functions of the digestive, metabolic and endocrine systems								
Tsuchihashi‐Makaya (2013)										
		Mental functions	Quality of life	Health service utilization	Residential home/apartment	Usual care		No ‐ assessment of effects by sex/gender NOT present	No ‐ assessment of effects across any other PROGRESS characteristics? For example, socioeconomic status	RCT
Turunen (2017)										
		Sensory functions and pain	Mobility		Residential home/apartment	Usual care		No ‐ assessment of effects by sex/gender NOT present	No ‐ assessment of effects across any other PROGRESS characteristics? For example, socioeconomic status	RCT
		Neuromusculoskeletal function								
Underwood (2013)										
		Mental functions	Quality of life		Assisted living	Other		No ‐ assessment of effects by sex/gender NOT present	No ‐ assessment of effects across any other PROGRESS characteristics? For example, socioeconomic status	RCT
		Sensory functions and pain	Contribution							
Valdes (2015)										
		Sensory functions and pain			Residential home/apartment	Other		No ‐ assessment of effects by sex/gender NOT present	No ‐ assessment of effects across any other PROGRESS characteristics? For example, socioeconomic status	RCT
		Neuromusculoskeletal function								
Van Der Pols‐Vijlbrief (2016)										
		Mental functions	Basic needs	Cost‐effectiveness	Assisted living	Usual care		No ‐ assessment of effects by sex/gender NOT present	No ‐ assessment of effects across any other PROGRESS characteristics? For example, socioeconomic status	RCT
		Functions of the digestive, metabolic and endocrine systems	Quality of life	Health service utilization						
		Neuromusculoskeletal function	Mobility							
van Haastregt (2000)										
		Mental functions	Basic needs	Falls	Residential home/apartment	Usual care		No ‐ assessment of effects by sex/gender NOT present	No ‐ assessment of effects across any other PROGRESS characteristics? For example, socioeconomic status	RCT
		Neuromusculoskeletal function	Mobility							
			Contribution							
van Hout (2010)										
			Basic needs	Health service utilization	Residential home/apartment	Usual care		No ‐ assessment of effects by sex/gender NOT present	No ‐ assessment of effects across any other PROGRESS characteristics? For example, socioeconomic status	RCT
							
van Houten (2007)										
		Genitourinary and reproductive functions	Basic needs		Assisted living	Usual care		No ‐ assessment of effects by sex/gender NOT present	No ‐ assessment of effects across any other PROGRESS characteristics? For example, socioeconomic status	RCT
Vass (2007)										
				Health service utilization	Residential home/apartment	Other		No ‐ assessment of effects by sex/gender NOT present	No ‐ assessment of effects across any other PROGRESS characteristics? For example, socioeconomic status	RCT
Verweij (2018)										
		Mental functions	Basic needs	Adherence	Residential home/apartment	Usual care		No ‐ assessment of effects by sex/gender NOT present	No ‐ assessment of effects across any other PROGRESS characteristics? For example, socioeconomic status	RCT
			Quality of life	Caregiver outcomes						
				Health service utilization						
Vogler (2009)										
		Mental functions	Basic needs	Safety	Residential home/apartment	Other		No ‐ assessment of effects by sex/gender NOT present	No ‐ assessment of effects across any other PROGRESS characteristics? For example, socioeconomic status	RCT
		Neuromusculoskeletal function	Quality of life	Falls	Independent living					
			Mobility							
Weir (1998)										
			Basic needs	Satisfaction of older adult	Residential home/apartment	Usual care		No ‐ assessment of effects by sex/gender NOT present	No ‐ assessment of effects across any other PROGRESS characteristics? For example, socioeconomic status	RCT
		Cost‐effectiveness								
				Caregiver outcomes						
Whitehead (2014)										
			Basic needs	Cost‐effectiveness	Residential home/apartment	Usual care		No ‐ assessment of effects by sex/gender NOT present	No ‐ assessment of effects across any other PROGRESS characteristics? For example, socioeconomic status	RCT
			Quality of life	Health service utilization						
Wilhelmson (2013)										
		Mental functions	Basic needs	Satisfaction of older adult	Residential home/apartment	Usual care		No ‐ assessment of effects by sex/gender NOT present	No ‐ assessment of effects across any other PROGRESS characteristics? For example, socioeconomic status	RCT
			Financial security and stability							
			Build and maintain relationships							
Wilson (2009)										
			Basic needs		Residential home/apartment	Usual care		No ‐ assessment of effects by sex/gender NOT present	No ‐ assessment of effects across any other PROGRESS characteristics? For example, socioeconomic status	RCT
Wishart (2000)										
			Basic needs	Satisfaction of older adult	Residential home/apartment	Other		No ‐ assessment of effects by sex/gender NOT present	No ‐ assessment of effects across any other PROGRESS characteristics? For example, socioeconomic status	RCT
			Quality of life	Cost‐effectiveness						
				Caregiver outcomes						
				Health service utilization						
Wisniowska‐Szurlej (2017)										
		Mental functions	Quality of life		Assisted living	Other		No ‐ assessment of effects by sex/gender NOT present	No ‐ assessment of effects across any other PROGRESS characteristics? For example, socioeconomic status	RCT
		Neuromusculoskeletal function	Mobility							
Wong (2015)										
		Mental functions	Basic needs	Satisfaction of older adult	Residential home/apartment	Usual care		No ‐ assessment of effects by sex/gender NOT present	No ‐ assessment of effects across any other PROGRESS characteristics? For example, socioeconomic status	RCT
			Quality of life	Health service utilization						
Wong (2016)										
		Mental functions	Basic needs	Satisfaction of older adult	Residential home/apartment	Usual care		No ‐ assessment of effects by sex/gender NOT present	No ‐ assessment of effects across any other PROGRESS characteristics? For example, socioeconomic status	RCT
		Sensory functions and pain	Quality of life	Health service utilization						
Wylie (2017)										
		Neuromusculoskeletal function	Basic needs	Adherence	Assisted living	Usual care		No ‐ assessment of effects by sex/gender NOT present	No ‐ assessment of effects across any other PROGRESS characteristics? For example, socioeconomic status	RCT
			Quality of life	Falls						
			Mobility							
Young (1992)										
		Mental functions	Basic needs	Caregiver outcomes	Residential home/apartment	Other		No ‐ assessment of effects by sex/gender NOT present	No ‐ assessment of effects across any other PROGRESS characteristics? For example, socioeconomic status	RCT
		Sensory functions and pain	Mobility							
		Neuromusculoskeletal function	Contribution							
Ziden (2008)										
			Basic needs	Falls	Residential home/apartment	Usual care		No ‐ assessment of effects by sex/gender NOT present	No ‐ assessment of effects across any other PROGRESS characteristics? For example, socioeconomic status	RCT
			Mobility							
			Contribution							
Ziden (2010)										
		Mental functions	Basic needs	Falls	Residential home/apartment	Other		No ‐ assessment of effects by sex/gender NOT present	No ‐ assessment of effects across any other PROGRESS characteristics? For example, socioeconomic status	RCT
		Neuromusculoskeletal function	Quality of life							
			Mobility							
Ziden (2014)										
		Mental functions	Basic needs		Residential home/apartment	Usual care		No ‐ assessment of effects by sex/gender NOT present	No ‐ assessment of effects across any other PROGRESS characteristics? For example, socioeconomic status	RCT
					Neuromusculoskeletal function	Mobility				
Zimmer (1985)										
				Cost (e.g., out of pocket)	Residential home/apartment	Usual care		No ‐ assessment of effects by sex/gender NOT present	No ‐ assessment of effects across any other PROGRESS characteristics? For example, socioeconomic status	RCT
				Health service utilization						
Abdulla (2013)										
	Adaptations to physical environment	Mental functions	Basic needs		Residential home/apartment	Other		No ‐ assessment of effects by sex/gender NOT present	No ‐ assessment of effects across any other PROGRESS characteristics? For example, socioeconomic status	Low/critically low quality SR
		Sensory functions and pain	Quality of life							
		Neuromusculoskeletal function								
Allen et al. (2014)										
				Cost (e.g., out of pocket)	Residential home/apartment	Usual care		No ‐ assessment of effects by sex/gender NOT present	No ‐ assessment of effects across any other PROGRESS characteristics? For example, socioeconomic status	Moderate quality SR
				Satisfaction of older adult						
				Caregiver outcomes						
				Safety						
				Health service utilization						
Andy (2016)										
			Basic needs	Satisfaction of older adult	Residential home/apartment	Usual care		No ‐ assessment of effects by sex/gender NOT present	No ‐ assessment of effects across any other PROGRESS characteristics? For example, socioeconomic status	High quality SR
			Quality of life	Cost‐effectiveness						
				Health service utilization						
Apostolo (2018)										
		Mental functions	Basic needs	Cost (e.g., out of pocket)	Assisted living	Usual care		No ‐ assessment of effects by sex/gender NOT present	No ‐ assessment of effects across any other PROGRESS characteristics? For example, socioeconomic status	Low/critically low quality SR
		Neuromusculoskeletal function	Quality of life	Cost‐effectiveness						
		Caregiver outcomes					
Baldwin (2011)										
		Functions of the cardiovascular, haematological, immunological and respiratory systems	Quality of life	Cost‐effectiveness	Residential home/apartment	Other		No ‐ assessment of effects by sex/gender NOT present	Planned but not reported	High quality SR
		Functions of the digestive, metabolic and endocrine systems								
Baxter (2016)										
		Neuromusculoskeletal function	Basic needs		Independent living	Usual care		No ‐ assessment of effects by sex/gender NOT present	No ‐ assessment of effects across any other PROGRESS characteristics? For example, socioeconomic status	Low/critically low quality SR
			Mobility			Other				
Berger (2013)										
	Adaptations to physical environment		Contribution		Residential home/apartment	Usual care		No ‐ assessment of effects by sex/gender NOT present	No ‐ assessment of effects across any other PROGRESS characteristics? For example, socioeconomic status	Low/critically low quality SR
						Other				
Beswick (2010)										
			Basic needs	Health service utilization	Residential home/apartment	Usual care		No ‐ assessment of effects by sex/gender NOT present	No ‐ assessment of effects across any other PROGRESS characteristics? For example, socioeconomic status	Low/critically low quality SR
				Falls						
Blythe (2009)										
		Mental functions			Assisted living	Other		No ‐ assessment of effects by sex/gender NOT present	No ‐ assessment of effects across any other PROGRESS characteristics? For example, socioeconomic status	Low/critically low quality SR
Bryant‐Lukosius (2015)										
			Quality of life	Cost (e.g., out of pocket)	Residential home/apartment	Other		No ‐ assessment of effects by sex/gender NOT present	No ‐ assessment of effects across any other PROGRESS characteristics? For example, socioeconomic status	Low/critically low quality SR
				Satisfaction of older adult						
				Cost‐effectiveness						
				Caregiver outcomes						
				Health service utilization						
Bula (2011)										
		Neuromusculoskeletal function		Falls	Residential home/apartment	Other		No ‐ assessment of effects by sex/gender NOT present	No ‐ assessment of effects across any other PROGRESS characteristics? For example, socioeconomic status	Low/critically low quality SR
Bunn (2016)										
		Functions of the digestive, metabolic and endocrine systems	Basic needs		Assisted living	Other		No ‐ assessment of effects by sex/gender NOT present	No ‐ assessment of effects across any other PROGRESS characteristics? For example, socioeconomic status	Low/critically low quality SR
Burns (2001)										
				Health service utilization	Residential home/apartment	Usual care		No ‐ assessment of effects by sex/gender NOT present	No ‐ assessment of effects across any other PROGRESS characteristics? For example, socioeconomic status	High quality SR
Burton (2015)										
		Mental functions	Mobility	Adherence	Residential home/apartment	Usual care		No ‐ assessment of effects by sex/gender NOT present	No ‐ assessment of effects across any other PROGRESS characteristics? For example, socioeconomic status	Low/critically low quality SR
		Neuromusculoskeletal function		Falls						
Burton (2015)										
		Neuromusculoskeletal function	Basic needs		Residential home/apartment	Other		No ‐ assessment of effects by sex/gender NOT present	No ‐ assessment of effects across any other PROGRESS characteristics? For example, socioeconomic status	Low/critically low quality SR
			Mobility							
Cadore (2013)										
		Neuromusculoskeletal function		Falls	Residential home/apartment	Usual care		No ‐ assessment of effects by sex/gender NOT present	No ‐ assessment of effects across any other PROGRESS characteristics? For example, socioeconomic status	Low/critically low quality SR
						Other				
Candy (2011)										
				Satisfaction of older adult	Residential home/apartment	Other		No ‐ assessment of effects by sex/gender NOT present	No ‐ assessment of effects across any other PROGRESS characteristics? For example, socioeconomic status	Moderate quality SR
				Cost‐effectiveness	Long‐term care					
					Assisted living					
Cattan (2005)										
		Mental functions	Build and maintain relationships		Residential home/apartment		Social capital	No ‐ assessment of effects by sex/gender NOT present	No ‐ assessment of effects across any other PROGRESS characteristics? For example, socioeconomic status	Low/critically low quality SR
					Independent living					
Chiung‐Ju (2013)										
			Basic needs		Residential home/apartment	Other		No ‐ assessment of effects by sex/gender NOT present	No ‐ assessment of effects across any other PROGRESS characteristics? For example, socioeconomic status	Low/critically low quality SR
Chou (2012)										
		Neuromusculoskeletal function	Basic needs		Residential home/apartment	Other		No ‐ assessment of effects by sex/gender NOT present	No ‐ assessment of effects across any other PROGRESS characteristics? For example, socioeconomic status	Low/critically low quality SR
			Quality of life		Assisted living					
Clarkson (2018)										
		Mental functions	Basic needs	Health service utilization	Residential home/apartment	Usual care		No ‐ assessment of effects by sex/gender NOT present	No ‐ assessment of effects across any other PROGRESS characteristics? For example, socioeconomic status	Low/critically low quality SR
		Neuromusculoskeletal function								
Clegg (2012)										
		Neuromusculoskeletal function	Basic needs	Health service utilization	Residential home/apartment	Other		No ‐ assessment of effects by sex/gender NOT present	No ‐ assessment of effects across any other PROGRESS characteristics? For example, socioeconomic status	Low/critically low quality SR
			Quality of life	Falls						
			Mobility							
Cobban (2012)										
		Functions of the digestive, metabolic and endocrine systems	Basic needs		Long‐term care	Other		No ‐ assessment of effects by sex/gender NOT present	No ‐ assessment of effects across any other PROGRESS characteristics? For example, socioeconomic status	Moderate quality SR
				Assisted living						
Cochrane (2014)										
			Basic needs	Satisfaction of older adult	Residential home/apartment			No ‐ assessment of effects by sex/gender NOT present	Planned but not reported	High quality SR
			Quality of life	Cost‐effectiveness						
				Health service utilization						
Corrieri (2011)										
				Cost‐effectiveness	Residential home/apartment	Other		No ‐ assessment of effects by sex/gender NOT present	No ‐ assessment of effects across any other PROGRESS characteristics? For example, socioeconomic status	Low/critically low quality SR
				Falls						
Crocker (2013)										
		Mental functions	Basic needs	Cost‐effectiveness	Long‐term care	Other		No ‐ assessment of effects by sex/gender NOT present	No ‐ assessment of effects across any other PROGRESS characteristics? For example, socioeconomic status	Low/critically low quality SR
		Neuromusculoskeletal function		Falls						
Daniels (2008)										
		Neuromusculoskeletal function	Basic needs		Residential home/apartment	Usual care		No ‐ assessment of effects by sex/gender NOT present	No ‐ assessment of effects across any other PROGRESS characteristics? For example, socioeconomic status	Low/critically low quality SR
Davis (2015)										
		Mental functions	Quality of life	Satisfaction of older adult	Residential home/apartment	Usual care		No ‐ assessment of effects by sex/gender NOT present	No ‐ assessment of effects across any other PROGRESS characteristics? For example, socioeconomic status	Low/critically low quality SR
				Cost‐effectiveness						
		Caregiver outcomes					
		Health service utilization					
De Coninck (2017)										
		Mental functions	Basic needs		Residential home/apartment	Usual care		No ‐ assessment of effects by sex/gender NOT present	No ‐ assessment of effects across any other PROGRESS characteristics? For example, socioeconomic status	Moderate quality SR
de Vries (2012)										
		Neuromusculoskeletal function	Quality of life		Residential home/apartment	Other		No ‐ assessment of effects by sex/gender NOT present	No ‐ assessment of effects across any other PROGRESS characteristics? For example, socioeconomic status	Low/critically low quality SR
			Mobility							
Desheng (2018)										
		Sensory functions and pain	Mobility	Health service utilization	Residential home/apartment	Other		No ‐ assessment of effects by sex/gender NOT present	No ‐ assessment of effects across any other PROGRESS characteristics? For example, socioeconomic status	Low/critically low quality SR
Dickens (2011)										
		Mental functions	Build and maintain relationships		Residential home/apartment	Usual care		No ‐ assessment of effects by sex/gender NOT present	No ‐ assessment of effects across any other PROGRESS characteristics? For example, socioeconomic status	Moderate quality SR
					Independent living					
		Assisted living				
Eklund (2009)										
				Caregiver outcomes	Residential home/apartment	Other		No ‐ assessment of effects by sex/gender NOT present	No ‐ assessment of effects across any other PROGRESS characteristics? For example, socioeconomic status	Low/critically low quality SR
				Health service utilization						
Elkan (2001)										
		Mental functions	Basic needs	Health service utilization	Residential home/apartment	Other		No ‐ assessment of effects by sex/gender NOT present	No ‐ assessment of effects across any other PROGRESS characteristics? For example, socioeconomic status	Low/critically low quality SR
								
							
Evans (2003)										
		Neuromusculoskeletal function	Basic needs		Independent living	Other		No ‐ assessment of effects by sex/gender NOT present	No ‐ assessment of effects across any other PROGRESS characteristics? For example, socioeconomic status	Low/critically low quality SR
					Assisted living					
Fletcher‐Smith (2013)										
		Mental functions	Basic needs	Satisfaction of older adult	Long‐term care	Usual care		No ‐ assessment of effects by sex/gender NOT present	No ‐ assessment of effects across any other PROGRESS characteristics? For example, socioeconomic status	Moderate quality SR
			Quality of life	Cost‐effectiveness	Assisted living					
			Mobility	Health service utilization						
Fomiatti (2013)										
			Basic needs		Residential home/apartment	Other		No ‐ assessment of effects by sex/gender NOT present	No ‐ assessment of effects across any other PROGRESS characteristics? For example, socioeconomic status	Low/critically low quality SR
			Contribution							
Forbes (2015)										
		Mental functions	Basic needs	Caregiver outcomes	Residential home/apartment	Usual care		No ‐ assessment of effects by sex/gender NOT present	No ‐ assessment of effects across any other PROGRESS characteristics? For example, socioeconomic status	Moderate quality SR
				Health service utilization	Long‐term care	Other				
					Assisted living					
Franck (2016)										
		Mental functions			Assisted living			No ‐ assessment of effects by sex/gender NOT present	No ‐ assessment of effects across any other PROGRESS characteristics? For example, socioeconomic status	Moderate quality SR
Gillespie (2012)										
	Adaptations to physical environment			Cost‐effectiveness	Residential home/apartment	Usual care		No ‐ assessment of effects by sex/gender NOT present	No ‐ assessment of effects across any other PROGRESS characteristics? For example, socioeconomic status	Moderate quality SR
				Safety	Independent living					
				Falls						
Gine‐Garriga (2014)										
		Neuromusculoskeletal function	Basic needs		Residential home/apartment			No ‐ assessment of effects by sex/gender NOT present	No ‐ assessment of effects across any other PROGRESS characteristics? For example, socioeconomic status	Low/critically low quality SR
			Mobility							
Golding‐Day (2017)										
	Adaptations to physical environment	Mental functions	Basic needs	Satisfaction of older adult	Residential home/apartment	Usual care		No ‐ assessment of effects by sex/gender NOT present	No ‐ assessment of effects across any other PROGRESS characteristics? For example, socioeconomic status	Moderate quality SR
			Quality of life	Cost‐effectiveness						
			Caregiver outcomes					
			Health service utilization					
			Falls					
Gomes (2013)										
		Sensory functions and pain	Quality of life	Cost (e.g., out of pocket)	Residential home/apartment	Usual care		No ‐ assessment of effects by sex/gender NOT present	No ‐ assessment of effects across any other PROGRESS characteristics? For example, socioeconomic status	Moderate quality SR
				Satisfaction of older adult						
				Cost‐effectiveness						
				Caregiver outcomes						
Grant (2014)										
		Mental functions	Basic needs	Health service utilization	Residential home/apartment	Other		No ‐ assessment of effects by sex/gender NOT present	No ‐ assessment of effects across any other PROGRESS characteristics? For example, socioeconomic status	High quality SR
			Quality of life	Falls						
Graybill (2014)										
	Adaptations to physical environment	Sensory functions and pain	Basic needs	Cost‐effectiveness	Residential home/apartment	Other		No ‐ assessment of effects by sex/gender NOT present	No ‐ assessment of effects across any other PROGRESS characteristics? For example, socioeconomic status	Low/critically low quality SR
			Quality of life							
Hall (2011)										
		Sensory functions and pain	Quality of life	Satisfaction of older adult	Long‐term care	Usual care		No ‐ assessment of effects by sex/gender NOT present	No ‐ assessment of effects across any other PROGRESS characteristics? For example, socioeconomic status	Moderate quality SR
					Independent living					
					Assisted living					
Handoll (2009)										
		Mental functions	Basic needs	Adherence	Residential home/apartment	Other		No ‐ assessment of effects by sex/gender NOT present	No ‐ assessment of effects across any other PROGRESS characteristics? For example, socioeconomic status	Moderate quality SR
			Quality of life	Cost‐effectiveness	Assisted living					
				Caregiver outcomes						
				Health service utilization						
Handoll (2015)										
		Sensory functions and pain	Basic needs	Cost (e.g., out of pocket)	Residential home/apartment	Usual care		No ‐ assessment of effects by sex/gender NOT present	No ‐ assessment of effects across any other PROGRESS characteristics? For example, socioeconomic status	High quality SR
		Neuromusculoskeletal function	Mobility	Satisfaction of older adult						
		Adherence					
Hill (2015)										
		Mental functions	Basic needs	Adherence	Residential home/apartment	Usual care		No ‐ assessment of effects by sex/gender NOT present	No ‐ assessment of effects across any other PROGRESS characteristics? For example, socioeconomic status	Moderate quality SR
		Neuromusculoskeletal function		Falls		Other				
Hobbs (2013)										
		Neuromusculoskeletal function			Residential home/apartment	Usual care		No ‐ assessment of effects by sex/gender NOT present	No ‐ assessment of effects across any other PROGRESS characteristics? For example, socioeconomic status	Moderate quality SR
						Other				
Howe (2011)										
		Neuromusculoskeletal function	Mobility	Adherence	Residential home/apartment	Other		Planned but not reported	No ‐ assessment of effects across any other PROGRESS characteristics? For example, socioeconomic status	High quality SR
					Independent living					
					Assisted living					
Hunter (2018)										
			Basic needs		Residential home/apartment	Usual care		No ‐ assessment of effects by sex/gender NOT present	No ‐ assessment of effects across any other PROGRESS characteristics? For example, socioeconomic status	Moderate quality SR
						
Huss (2008)										
			Basic needs	Health service utilization	Residential home/apartment	Usual care		No ‐ assessment of effects by sex/gender NOT present	No ‐ assessment of effects across any other PROGRESS characteristics? For example, socioeconomic status	Low/critically low quality SR
Jane (2017)										
	Adaptations to physical environment	Mental functions	Basic needs	Cost‐effectiveness	Residential home/apartment	Usual care		No ‐ assessment of effects by sex/gender NOT present	No ‐ assessment of effects across any other PROGRESS characteristics? For example, socioeconomic status	Low/critically low quality SR
				Falls						
Kang‐Yi (2010)										
		Mental functions			Residential home/apartment	Other		No ‐ assessment of effects by sex/gender NOT present	No ‐ assessment of effects across any other PROGRESS characteristics? For example, socioeconomic status	Low/critically low quality SR
Konno (2011)										
		Mental functions		Satisfaction of older adult	Residential home/apartment	Usual care		No ‐ assessment of effects by sex/gender NOT present	No ‐ assessment of effects across any other PROGRESS characteristics? For example, socioeconomic status	
				Caregiver outcomes	Assisted living					
Konno (2013)										
		Mental functions		Satisfaction of older adult	Residential home/apartment	Usual care		No ‐ assessment of effects by sex/gender NOT present	No ‐ assessment of effects across any other PROGRESS characteristics? For example, socioeconomic status	Low/critically low quality SR
				Caregiver outcomes	Assisted living					
Konno (2014)										
		Mental functions			Assisted living	Other		No ‐ assessment of effects by sex/gender NOT present	No ‐ assessment of effects across any other PROGRESS characteristics? For example, socioeconomic status	Low/critically low quality SR
Kurz (2011)										
		Mental functions	Basic needs	Satisfaction of older adult	Residential home/apartment	Other		No ‐ assessment of effects by sex/gender NOT present	No ‐ assessment of effects across any other PROGRESS characteristics? For example, socioeconomic status	Low/critically low quality SR
			Quality of life	Caregiver outcomes	Assisted living					
Lacroix (2017)										
		Neuromusculoskeletal function			Residential home/apartment	Other		No ‐ assessment of effects by sex/gender NOT present	No ‐ assessment of effects across any other PROGRESS characteristics? For example, socioeconomic status	Low/critically low quality SR
Legg (2004)										
		Mental functions	Basic needs	Caregiver outcomes	Residential home/apartment	Usual care		No ‐ assessment of effects by sex/gender NOT present	No ‐ assessment of effects across any other PROGRESS characteristics? For example, socioeconomic status	Low/critically low quality SR
			Quality of life	Health service utilization						
Legg (2017)										
		Mental functions	Basic needs	Satisfaction of older adult	Residential home/apartment	Usual care		No ‐ assessment of effects by sex/gender NOT present	No ‐ assessment of effects across any other PROGRESS characteristics? For example, socioeconomic status	High quality SR
			Quality of life	Caregiver outcomes						
				Health service utilization						
Lewis (2017)										
		Neuromusculoskeletal function	Basic needs	Health service utilization	Residential home/apartment	Usual care		No ‐ assessment of effects by sex/gender NOT present	No ‐ assessment of effects across any other PROGRESS characteristics? For example, socioeconomic status	Moderate quality SR
				Falls						
Liimatta (2016)										
			Basic needs	Cost (e.g., out of pocket)	Residential home/apartment	Other		No ‐ assessment of effects by sex/gender NOT present	No ‐ assessment of effects across any other PROGRESS characteristics? For example, socioeconomic status	Low/critically low quality SR
			Quality of life	Cost‐effectiveness						
				Health service utilization						
Liu (2015)										
		Mental functions			Long‐term care	Other		No ‐ assessment of effects by sex/gender NOT present	No ‐ assessment of effects across any other PROGRESS characteristics? For example, socioeconomic status	Low/critically low quality SR
		Functions of the digestive, metabolic and endocrine systems								
Liu (2018)										
			Basic needs		Residential home/apartment	Other		No ‐ assessment of effects by sex/gender NOT present	No ‐ assessment of effects across any other PROGRESS characteristics? For example, socioeconomic status	Low/critically low quality SR
Low (2011)										
		Mental functions	Basic needs	Satisfaction of older adult	Residential home/apartment	Other		No ‐ assessment of effects by sex/gender NOT present	No ‐ assessment of effects across any other PROGRESS characteristics? For example, socioeconomic status	Low/critically low quality SR
		Sensory functions and pain	Quality of life	Caregiver outcomes						
			Build and maintain relationships	Health service utilization						
Martin (2011)										
		Mental functions			Residential home/apartment	Other		No ‐ assessment of effects by sex/gender NOT present	No ‐ assessment of effects across any other PROGRESS characteristics? For example, socioeconomic status	Moderate quality SR
					Independent living					
Mayo‐Wilson (2014)										
		Mental functions	Basic needs	Health service utilization	Residential home/apartment	Other		No ‐ assessment of effects by sex/gender NOT present	No ‐ assessment of effects across any other PROGRESS characteristics? For example, socioeconomic status	Moderate quality SR
			Quality of life	Falls						
McClure (2005)										
				Falls	Residential home/apartment	Other		No ‐ assessment of effects by sex/gender NOT present	No ‐ assessment of effects across any other PROGRESS characteristics? For example, socioeconomic status	Low/critically low quality SR
McWilliam (2000)										
			Basic needs	Health service utilization	Residential home/apartment	Other		No ‐ assessment of effects by sex/gender NOT present	No ‐ assessment of effects across any other PROGRESS characteristics? For example, socioeconomic status	Low/critically low quality SR
			Quality of life		Assisted living					
			Build and maintain relationships							
Meinck (2004)										
		Mental functions	Basic needs	Health service utilization	Residential home/apartment	Other		No ‐ assessment of effects by sex/gender NOT present	No ‐ assessment of effects across any other PROGRESS characteristics? For example, socioeconomic status	Low/critically low quality SR
Montgomery (2008)										
		Mental functions	Basic needs	Cost (e.g., out of pocket)	Residential home/apartment	Other		No ‐ assessment of effects by sex/gender NOT present	No ‐ assessment of effects across any other PROGRESS characteristics? For example, socioeconomic status	Moderate quality SR
		Sensory functions and pain	Quality of life	Satisfaction of older adult						
		Neuromusculoskeletal function	Contribution	Cost‐effectiveness						
			Build and maintain relationships	Health service utilization						
Munk (2016)										
		Neuromusculoskeletal function	Basic needs	Health service utilization	Residential home/apartment	Other		No ‐ assessment of effects by sex/gender NOT present	No ‐ assessment of effects across any other PROGRESS characteristics? For example, socioeconomic status	Moderate quality SR
			Quality of life							
Oliver (2007)										
				Falls	Long‐term care	Other		No ‐ assessment of effects by sex/gender NOT present	No ‐ assessment of effects across any other PROGRESS characteristics? For example, socioeconomic status	Low/critically low quality SR
Outpatient (2003)										
		Mental functions	Basic needs	Satisfaction of older adult	Residential home/apartment	Usual care		No ‐ assessment of effects by sex/gender NOT present	No ‐ assessment of effects across any other PROGRESS characteristics? For example, socioeconomic status	Moderate quality SR
			Quality of life	Caregiver outcomes						
				Health service utilization						
Ozdemir (2017)										
		Sensory functions and pain	Basic needs		Residential home/apartment	Other		No ‐ assessment of effects by sex/gender NOT present	No ‐ assessment of effects across any other PROGRESS characteristics? For example, socioeconomic status	Low/critically low quality SR
			Quality of life							
Patterson (1999)										
				Cost (e.g., out of pocket)	Residential home/apartment	Other		No ‐ assessment of effects by sex/gender NOT present	No ‐ assessment of effects across any other PROGRESS characteristics? For example, socioeconomic status	Low/critically low quality SR
				Access						
Pitkala (2013)										
		Neuromusculoskeletal function	Basic needs		Residential home/apartment	Other		No ‐ assessment of effects by sex/gender NOT present	No ‐ assessment of effects across any other PROGRESS characteristics? For example, socioeconomic status	Low/critically low quality SR
			Mobility		Assisted living					
Poscia (2018)										
		Mental functions	Quality of life		Residential home/apartment	Other		No ‐ assessment of effects by sex/gender NOT present	No ‐ assessment of effects across any other PROGRESS characteristics? For example, socioeconomic status	Low/critically low quality SR
		Neuromusculoskeletal function	Build and maintain relationships		Assisted living					
Potter (2011)										
		Mental functions	Quality of life	Falls	Residential home/apartment	Other		No ‐ assessment of effects by sex/gender NOT present	No ‐ assessment of effects across any other PROGRESS characteristics? For example, socioeconomic status	Low/critically low quality SR
		Neuromusculoskeletal function			Assisted living					
Reilly (2015)										
		Mental functions	Basic needs	Caregiver outcomes	Residential home/apartment	Other		No ‐ assessment of effects by sex/gender NOT present	No ‐ assessment of effects across any other PROGRESS characteristics? For example, socioeconomic status	High quality SR
			Quality of life	Health service utilization	Assisted living					
Renz (2017)										
			Basic needs	Health service utilization	Residential home/apartment	Usual care		No ‐ assessment of effects by sex/gender NOT present	No ‐ assessment of effects across any other PROGRESS characteristics? For example, socioeconomic status	Moderate quality SR
			Quality of life	Falls						
Resnick (2016)										
		Mental functions	Basic needs		Residential home/apartment	Other		No ‐ assessment of effects by sex/gender NOT present	No ‐ assessment of effects across any other PROGRESS characteristics? For example, socioeconomic status	Low/critically low quality SR
		Neuromusculoskeletal function			Assisted living					
Roe (2015)										
		Genitourinary and reproductive functions			Assisted living	Other		No ‐ assessment of effects by sex/gender NOT present	No ‐ assessment of effects across any other PROGRESS characteristics? For example, socioeconomic status	Low/critically low quality SR
Roets‐Merken (2015)										
		Sensory functions and pain			Residential home/apartment			No ‐ assessment of effects by sex/gender NOT present	No ‐ assessment of effects across any other PROGRESS characteristics? For example, socioeconomic status	Low/critically low quality SR
Santomassino (2012)										
				Satisfaction of older adult	Residential home/apartment	Other		No ‐ assessment of effects by sex/gender NOT present	No ‐ assessment of effects across any other PROGRESS characteristics? For example, socioeconomic status	Low/critically low quality SR
				Health service utilization						
Sean (2014)										
		Mental functions	Basic needs	Health service utilization	Residential home/apartment	Usual care		No ‐ assessment of effects by sex/gender NOT present	No ‐ assessment of effects across any other PROGRESS characteristics? For example, socioeconomic status	High quality SR
			Quality of life	Falls						
Shaw (2009)										
				Cost (e.g., out of pocket)	Residential home/apartment	Usual care		No ‐ assessment of effects by sex/gender NOT present	No ‐ assessment of effects across any other PROGRESS characteristics? For example, socioeconomic status	Low/critically low quality SR
				Cost‐effectiveness	Long‐term care					
				Caregiver outcomes						
Shepperd (2005)										
		Mental functions		Satisfaction of older adult	Residential home/apartment	Other		No ‐ assessment of effects by sex/gender NOT present	No ‐ assessment of effects across any other PROGRESS characteristics? For example, socioeconomic status	Moderate quality SR
		Neuromusculoskeletal function		Caregiver outcomes						
				Health service utilization						
Shepperd (2011)										
		Mental functions	Quality of life	Satisfaction of older adult	Residential home/apartment	Usual care		No ‐ assessment of effects by sex/gender NOT present	No ‐ assessment of effects across any other PROGRESS characteristics? For example, socioeconomic status	Moderate quality SR
		Sensory functions and pain		Health service utilization		Other				
		Functions of the digestive, metabolic and endocrine systems								
Shepperd (2016)										
		Mental functions	Quality of life	Cost (e.g., out of pocket)	Residential home/apartment	Other		No ‐ assessment of effects by sex/gender NOT present	No ‐ assessment of effects across any other PROGRESS characteristics? For example, socioeconomic status	Low/critically low quality SR
				Satisfaction of older adult						
				Health service utilization						
Shvedko (2018)										
		Mental functions	Quality of life		Residential home/apartment	Usual care	Social capital	No ‐ assessment of effects by sex/gender NOT present	No ‐ assessment of effects across any other PROGRESS characteristics? For example, socioeconomic status	Moderate quality SR
			Build and maintain relationships							
Simek (2012)										
				Adherence	Residential home/apartment	Other		No ‐ assessment of effects by sex/gender NOT present	No ‐ assessment of effects across any other PROGRESS characteristics? For example, socioeconomic status	Moderate quality SR
				Falls						
Sims‐Gould (2017)										
		Neuromusculoskeletal function	Basic needs	Health service utilization	Residential home/apartment	Usual care		No ‐ assessment of effects by sex/gender NOT present	No ‐ assessment of effects across any other PROGRESS characteristics? For example, socioeconomic status	Moderate quality SR
			Build and maintain relationships		Assisted living					
Skelton (2013)										
	Adaptations to physical environment	Mental functions	Quality of life	Falls	Residential home/apartment	Other		No ‐ assessment of effects by sex/gender NOT present	No ‐ assessment of effects across any other PROGRESS characteristics? For example, socioeconomic status	High quality SR
Smeeth (2006)										
		Sensory functions and pain			Residential home/apartment	Other		No ‐ assessment of effects by sex/gender NOT present	No ‐ assessment of effects across any other PROGRESS characteristics? For example, socioeconomic status	Moderate quality SR
Smith (2016)										
		Mental functions	Basic needs	Satisfaction of older adult	Residential home/apartment	Usual care		No ‐ assessment of effects by sex/gender NOT present	No ‐ assessment of effects across any other PROGRESS characteristics? For example, socioeconomic status	High quality SR
			Quality of life	Adherence						
				Cost‐effectiveness						
				Access						
				Health service utilization						
Stall (2014)										
			Quality of life	Cost (e.g., out of pocket)	Residential home/apartment	Other		No ‐ assessment of effects by sex/gender NOT present	No ‐ assessment of effects across any other PROGRESS characteristics? For example, socioeconomic status	Low/critically low quality SR
				Satisfaction of older adult						
				Cost‐effectiveness						
				Caregiver outcomes						
				Health service utilization						
Steultjens (2004)										
		Mental functions	Basic needs	Health service utilization	Residential home/apartment	Usual care		No ‐ assessment of effects by sex/gender NOT present	No ‐ assessment of effects across any other PROGRESS characteristics? For example, socioeconomic status	Low/critically low quality SR
		Sensory functions and pain	Quality of life	Falls						
			Build and maintain relationships							
Steultjens (2004)										
		Mental functions	Basic needs	Health service utilization	Residential home/apartment	Other		No ‐ assessment of effects by sex/gender NOT present	No ‐ assessment of effects across any other PROGRESS characteristics? For example, socioeconomic status	Low/critically low quality SR
		Sensory functions and pain	Quality of life	Falls						
		Neuromusculoskeletal function	Contribution							
Stolee (2012)										
		Mental functions	Quality of life		Residential home/apartment	Usual care		No ‐ assessment of effects by sex/gender NOT present	No ‐ assessment of effects across any other PROGRESS characteristics? For example, socioeconomic status	Low/critically low quality SR
							
Stuck (2002)										
			Basic needs	Health service utilization	Residential home/apartment	Other		No ‐ assessment of effects by sex/gender NOT present	No ‐ assessment of effects across any other PROGRESS characteristics? For example, socioeconomic status	Low/critically low quality SR
Talley (2011)										
		Genitourinary and reproductive functions	Quality of life		Residential home/apartment	Other		No ‐ assessment of effects by sex/gender NOT present	No ‐ assessment of effects across any other PROGRESS characteristics? For example, socioeconomic status	Low/critically low quality SR
Tappenden (2012)										
		Mental functions		Satisfaction of older adult	Residential home/apartment	Usual care		No ‐ assessment of effects by sex/gender NOT present	No ‐ assessment of effects across any other PROGRESS characteristics? For example, socioeconomic status	Moderate quality SR
				Cost‐effectiveness						
				Health service utilization						
				Falls						
Therapy‐based rehabilitation. . . (2003)										
		Mental functions	Basic needs	Satisfaction of older adult	Residential home/apartment	Other		No ‐ assessment of effects by sex/gender NOT present	No ‐ assessment of effects across any other PROGRESS characteristics? For example, socioeconomic status	Low/critically low quality SR
			Quality of life	Caregiver outcomes	Independent living					
				Health service utilization						
Thiebaud (2014)										
		Neuromusculoskeletal function	Basic needs		Residential home/apartment	Usual care		No ‐ assessment of effects by sex/gender NOT present	No ‐ assessment of effects across any other PROGRESS characteristics? For example, socioeconomic status	Low/critically low quality SR
			Mobility							
Toles (2016)										
			Basic needs	Satisfaction of older adult	Residential home/apartment	Usual care		No ‐ assessment of effects by sex/gender NOT present	No ‐ assessment of effects across any other PROGRESS characteristics? For example, socioeconomic status	Moderate quality SR
			Quality of life	Health service utilization	Assisted living					
Tseng (2011)										
		Mental functions			Residential home/apartment	Other		No ‐ assessment of effects by sex/gender NOT present	No ‐ assessment of effects across any other PROGRESS characteristics? For example, socioeconomic status	Low/critically low quality SR
Vaapio (2009)										
			Quality of life		Residential home/apartment	Other		No ‐ assessment of effects by sex/gender NOT present	No ‐ assessment of effects across any other PROGRESS characteristics? For example, socioeconomic status	Low/critically low quality SR
					Assisted living					
van Abbema (2015)										
		Neuromusculoskeletal function			Residential home/apartment	Other		No ‐ assessment of effects by sex/gender NOT present	No ‐ assessment of effects across any other PROGRESS characteristics? For example, socioeconomic status	Low/critically low quality SR
Van Citters (2004)										
		Mental functions			Residential home/apartment	Other		No ‐ assessment of effects by sex/gender NOT present	No ‐ assessment of effects across any other PROGRESS characteristics? For example, socioeconomic status	Low/critically low quality SR
Ward (2003)										
			Basic needs	Satisfaction of older adult	Residential home/apartment	Other		No ‐ assessment of effects by sex/gender NOT present	No ‐ assessment of effects across any other PROGRESS characteristics? For example, socioeconomic status	Low/critically low quality SR
			Quality of life	Cost‐effectiveness	Long‐term care					
				Health service utilization	Independent living					
					Assisted living					
Watanabe (2015)										
		Sensory functions and pain	Basic needs		Residential home/apartment	Usual care		No ‐ assessment of effects by sex/gender NOT present	No ‐ assessment of effects across any other PROGRESS characteristics? For example, socioeconomic status	
			Quality of life							
			Mobility							
Weber (2018)										
		Mental functions	Mobility	Falls	Residential home/apartment	Other		No ‐ assessment of effects by sex/gender NOT present	No ‐ assessment of effects across any other PROGRESS characteristics? For example, socioeconomic status	Moderate quality SR
		Neuromusculoskeletal function								
Winkel (2008)										
			Basic needs	Cost (e.g., out of pocket)	Residential home/apartment	Usual care		No ‐ assessment of effects by sex/gender NOT present	No ‐ assessment of effects across any other PROGRESS characteristics? For example, socioeconomic status	Low/critically low quality SR
			Quality of life	Satisfaction of older adult						
				Access						
				Safety						
Yi (2015)										
		Integumentary system			Long‐term care	Other		No ‐ assessment of effects by sex/gender NOT present	No ‐ assessment of effects across any other PROGRESS characteristics? For example, socioeconomic status	
					Assisted living					
Young (2017)										
			Quality of life	Satisfaction of older adult	Residential home/apartment	Usual care		No ‐ assessment of effects by sex/gender NOT present	No ‐ assessment of effects across any other PROGRESS characteristics? For example, socioeconomic status	High quality SR
				Health service utilization	Long‐term care					
Zhu (2013)										
		Functions of the digestive, metabolic and endocrine systems			Residential home/apartment	Other		No ‐ assessment of effects by sex/gender NOT present	No ‐ assessment of effects across any other PROGRESS characteristics? For example, socioeconomic status	Low/critically low quality SR
Zubala (2017)										
		Mental functions		Adherence	Residential home/apartment	Other		No ‐ assessment of effects by sex/gender NOT present	No ‐ assessment of effects across any other PROGRESS characteristics? For example, socioeconomic status	Moderate quality SR
		Neuromusculoskeletal function			Independent living					
					Assisted living					

**EXCLUDED STUDIES**

**Table:** 

Study	Reason for Exclusion
Aasgaard et al. (2012)	EXCLUDE on study design
Abrisqueta‐Gomez et al. (2013)	EXCLUDE on study design
Aceros et al. (2016)	EXCLUDE on intervention
Achterberg (2016)	EXCLUDE on intervention
Acierno et al. (2017)	EXCLUDE on target group
Acorn (2008)	EXCLUDE on study design
Adachi et al. (2001)	EXCLUDE on intervention
Ades et al. (2003)	EXCLUDE on intervention
Afifi et al. (2014)	EXCLUDE on study design
Agmon and Embon‐Magal (2018)	EXCLUDE on setting
Agree (1999)	EXCLUDE on study design
Agree et al. (2005)	EXCLUDE on study design
Aguado et al. (2010)	EXCLUDE on intervention
Aguglia et al. (2004)	EXCLUDE on intervention
Aguila (2006)	EXCLUDE on study design
Ahlner‐Elmqvist et al. (2008)	EXCLUDE on study design
Ahmad (2016)	EXCLUDE on intervention
Ahmad (2018)	EXCLUDE on intervention
Aiken et al. (2006)	EXCLUDE on target group
Åkesson et al. (2018)	EXCLUDE on setting
Akiyama (2011)	EXCLUDE on study design
Albertsen (2011)	EXCLUDE on intervention
Albornos‐Muñoz et al. (2018)	EXCLUDE on setting
Alessi et al. (1997)	EXCLUDE on study design
Alkan et al. (2011)	EXCLUDE on intervention
Alldred et al. (2013)	EXCLUDE on intervention
Allen (1996)	EXCLUDE on study design
Allen (1999)	EXCLUDE on study design
Allen et al. (2012)	EXCLUDE on study design
Allen et al. (2014)	EXCLUDE on intervention
Allen et al. (2014)	EXCLUDE on intervention
Al‐Sari et al. (2018)	EXCLUDE on intervention
Anderson et al. (2012)	EXCLUDE on setting
Antoniak and Greig (2017)	EXCLUDE on setting
Anttila et al. (2011)	EXCLUDE on study design
Anttila et al. (2012)	EXCLUDE on study design
Aoun et al. (2015)	EXCLUDE on study design
Apóstolo (2016)	EXCLUDE on intervention
Applebaum and Phillips (1990)	EXCLUDE on study design
Applegate (1991)	EXCLUDE on study design
Arai et al. (2007)	EXCLUDE on setting
Aranda (1974)	EXCLUDE on study design
Arbesman and Mosley (2012)	EXCLUDE on setting
Arean et al. (2008)	EXCLUDE on setting
Arif et al. (2014)	EXCLUDE on study design
Armstrong et al. (2016)	EXCLUDE on study design
Arnall et al. (2012)	EXCLUDE on setting
Aronson and Neysmith (1996)	EXCLUDE on study design
Arthur (2000)	EXCLUDE on intervention
Ashok (2017)	EXCLUDE on study design
Ashworth et al. (2005)	EXCLUDE on target group
Assumpção (2014)	EXCLUDE on study design
Atienza (2001)	EXCLUDE on study design
Auger et al. (2008)	EXCLUDE on target group
Bahar‐Fuchs et al. (2017)	EXCLUDE on intervention
Bainbridge et al. (2016)	EXCLUDE on target group
Baker et al. (2001)	EXCLUDE on target group
Baker et al. (2016)	EXCLUDE on intervention
Bakker et al. (2011)	EXCLUDE on intervention
Baldwin et al. (2016)	EXCLUDE on intervention
Barnes et al. (2013)	EXCLUDE on intervention
Bateni and Maki (2005)	EXCLUDE on study design
Bates et al. (2018)	EXCLUDE on intervention
Belqaid et al. (2016)	EXCLUDE on intervention
Bentur et al. (1996)	EXCLUDE on intervention
Best and Solomon (1971)	EXCLUDE on study design
Best et al. (2014)	EXCLUDE on setting
Best et al. (2016)	EXCLUDE on setting
Beurskens (2016)	EXCLUDE on study design
Binder (2004)	EXCLUDE on setting
Bischoff‐Ferrari (2017)	EXCLUDE on intervention
Bishop et al. (2015)	EXCLUDE on intervention
Bismuth et al. (2012)	EXCLUDE on intervention
Blackwood et al. (2016)	EXCLUDE on intervention
Blake et al. (2009)	EXCLUDE on setting
Bleijenberg et al. (2013)	EXCLUDE on target group
Bleijenberg et al. (2017)	EXCLUDE on study design
Blohm (1998)	EXCLUDE on intervention
Boland et al. (2017)	EXCLUDE on study design
Bolscher‐Niehuis et al. (2016)	EXCLUDE on intervention
Borell (2018)	EXCLUDE on study design
Boucher et al. (2013)	EXCLUDE on study design
Boyd et al. (1996)	EXCLUDE on study design
Braun and Rose (1987)	EXCLUDE on study design
Braun et al. (1991)	EXCLUDE on study design
Brettschneider et al. (2015)	EXCLUDE on study design
Brismee et al. (2007)	EXCLUDE on setting
Britian (1999)	EXCLUDE on study design
Bruun‐Olsen et al. (2013)	EXCLUDE on setting
Bull (1994)	EXCLUDE on study design
Cabilan et al. (2013)	EXCLUDE on target group
Caplan et al. (2010)	EXCLUDE on intervention
Cardemil et al. (2013)	EXCLUDE on setting
Carlson et al. (2007)	EXCLUDE on intervention
Carlson et al. (2017)	EXCLUDE on target group
Challis et al. (1991)	EXCLUDE on study design
Chan et al. (2016)	EXCLUDE on setting
Chan et al. (2016)	EXCLUDE on setting
Chan et al. (2017)	EXCLUDE on setting
Chandler and Knackert (1997)	EXCLUDE on study design
Chesbro et al. (2005)	EXCLUDE on intervention
Chiatti et al. (2015)	EXCLUDE on target group
ChiCtr (2013)	EXCLUDE on intervention
Chien et al. (2008)	EXCLUDE on target group
Childress et al. (2008)	EXCLUDE on intervention
Chippendale (2012)	EXCLUDE on intervention
Chiu and Man (2004)	EXCLUDE on intervention
Chiu et al. (2015)	EXCLUDE on study design
Choi et al. (2014)	EXCLUDE on intervention
Chou (2011)	EXCLUDE on setting
Choyce et al. (2017)	EXCLUDE on target group
Chung and Zhao (2016)	EXCLUDE on setting
Cifu (2010)	EXCLUDE on study design
Ciliska et al.(1996)	EXCLUDE on target group
Clark (1998)	EXCLUDE on study design
Clarke and Colantonio (2005)	EXCLUDE on study design
Clegg (2014)	EXCLUDE on intervention
Clemson et al. (2012)	EXCLUDE on intervention
Coleman (1995)	EXCLUDE on study design
Coster et al. (2018)	EXCLUDE on study design
Cumming (2015)	EXCLUDE on setting
Cyarto et al. (2006)	EXCLUDE on study design
Dale and Brown (2006)	EXCLUDE on intervention
Dalton et al. (2018)	EXCLUDE on target group
Danilovich et al. (2017)	EXCLUDE on target group
Dapp et al. (2011)	EXCLUDE on intervention
Davison et al. (2016)	EXCLUDE on intervention
Day (2000)	EXCLUDE on intervention
Day et al. (2012)	EXCLUDE on setting
De Almeida (2015)	EXCLUDE on intervention
De Roos (2018)	EXCLUDE on setting
De van der Schueren (2017)	EXCLUDE on intervention
De Vriendt et al. (2016)	EXCLUDE on setting
De Vries (2016)	EXCLUDE on setting
Delbaere et al. (2006)	EXCLUDE on study design
Dellasega and Zerbe (2002)	EXCLUDE on target group
Der‐Fa et al. (2013)	EXCLUDE on intervention
Dohrn et al. (2017)	EXCLUDE on setting
Donaldson (1990)	EXCLUDE on study design
Donaldson and Bond (1991)	EXCLUDE on intervention
Douglas and Lawrence (2015)	EXCLUDE on study design
Douma et al. (2015)	EXCLUDE on intervention
Dozeman et al. (2011)	EXCLUDE on intervention
Dozeman et al. (2012)	EXCLUDE on intervention
Dreizler et al. (2014)	EXCLUDE on study design
Drummond et al. (2013)	EXCLUDE on setting
Duckworth et al. (2013)	EXCLUDE on study design
Dumoulin et al. (2014)	EXCLUDE on intervention
Effectiveness and cost‐effectiveness. . . (2016)	EXCLUDE on study design
Eklund et al. (2013)	EXCLUDE on setting
Elbadawy (2017)	EXCLUDE on setting
El‐Khoury et al. (2015)	EXCLUDE on setting
Eloranta (2010)	EXCLUDE on study design
Engberg et al. (1997)	EXCLUDE on study design
Evans (2007)	EXCLUDE on study design
Fahlman et al. (2007)	EXCLUDE on setting
Fairhall et al. (2015)	EXCLUDE on study design
Fanning et al. (2018)	EXCLUDE on setting
Farmer et al. (2006)	EXCLUDE on study design
Feldman et al. (2005)	EXCLUDE on intervention
Fernandez‐Barres et al. (2017)	EXCLUDE on intervention
Fields et al. (2014)	EXCLUDE on setting
Finkelstein and Fuller (2012)	EXCLUDE on study design
Fischer et al. (2015)	EXCLUDE on study design
Fitzgerald et al. (1994)	EXCLUDE on setting
Fleet et al. (2014)	EXCLUDE on intervention
Flora and Faulkner (2006)	EXCLUDE on study design
Forbes (2002)	EXCLUDE on study design
Forster et al. (2008)	EXCLUDE on setting
Forster et al. (2017)	EXCLUDE on intervention
Fowler and Kim (2015)	EXCLUDE on study design
Galle et al. (2017)	EXCLUDE on setting
Gary (2006)	EXCLUDE on intervention
Ghassemzadeh et al. (2013)	EXCLUDE on study design
Gianoudis et al. (2011)	EXCLUDE on setting
Gibson (2002)	EXCLUDE on study design
Gielen et al. (2013)	EXCLUDE on intervention
Giesbrecht et al. (2012)	EXCLUDE on study design
Gine‐Garriga et al. (2010)	EXCLUDE on setting
Giordano et al. (2016)	EXCLUDE on intervention
Gleeson et al. (2014)	EXCLUDE on intervention
Goedendorp et al. (2017)	EXCLUDE on intervention
Gollub (2002)	EXCLUDE on study design
Gomes (2018)	EXCLUDE on intervention
Gordon (1990)	EXCLUDE on study design
Gosman‐Hedstrom et al. (2002)	EXCLUDE on setting
Gothe et al. (2014)	EXCLUDE on setting
Graff et al. (2003)	EXCLUDE on study design
Graham‐Phillips et al. (2016)	EXCLUDE on study design
Gray and Sedhom (1997)	EXCLUDE on study design
Griffiths (2000)	EXCLUDE on study design
Griffiths (2013)	EXCLUDE on intervention
Gros et al. (2016)	EXCLUDE on study design
Guidetti et al. (2010)	EXCLUDE on setting
Guidon and McGee (2013)	EXCLUDE on setting
Guitard et al. (2013)	EXCLUDE on intervention
Haines et al. (2009)	EXCLUDE on intervention
Halvarsson et al. (2015)	EXCLUDE on setting
Hariprasad et al. (2013)	EXCLUDE on intervention
Harrison et al. (2008)	EXCLUDE on setting
Hauer (2015)	EXCLUDE on intervention
Hayashi et al. (2011)	EXCLUDE on setting
Healey (2011)	EXCLUDE on setting
Health (2008)	EXCLUDE on intervention
Heneka et al. (2016)	EXCLUDE on setting
Hennig et al. (2012)	EXCLUDE on study design
Henrard (1991)	EXCLUDE on study design
Herke et al. (2018)	EXCLUDE on intervention
Hile et al. (2018)	EXCLUDE on study design
Hinkka et al. (2007)	EXCLUDE on setting
Hinrichs et al. (2011)	EXCLUDE on intervention
Hirsch (2015)	EXCLUDE on study design
Hofstad et al. (2013)	EXCLUDE on study design
Holthe et al. (2018)	EXCLUDE on intervention
Hooper et al. (2014)	EXCLUDE on intervention
Hori et al. (2014)	EXCLUDE on intervention
Hu (2007)	EXCLUDE on intervention
Huang and Acton (2004)	EXCLUDE on intervention
Hughes et al. (1987)	EXCLUDE on study design
Hum et al. (2018)	EXCLUDE on study design
Humbert et al. (2007)	EXCLUDE on study design
Hummel et al. (2017)	EXCLUDE on intervention
Hung et al. (2003)	EXCLUDE on study design
Hussain (2013)	EXCLUDE on intervention
Iecovich (2007)	EXCLUDE on study design
Ifudu et al. (1994)	EXCLUDE on target group
Ilieva et al. (2013)	EXCLUDE on study design
Iliffe, Kendrick, et al. (2015)	EXCLUDE on intervention
Iliffe, Kendrick, et al. (2015)	EXCLUDE on intervention
Intiso et al. (2012)	EXCLUDE on study design
Istvandity (2017)	EXCLUDE on intervention
Iversen (2012)	EXCLUDE on study design
Iyengar et al. (2007)	EXCLUDE on study design
Jacobson et al. (2011)	EXCLUDE on intervention
Jame (2016)	EXCLUDE on intervention
Jansen et al. (2013)	EXCLUDE on setting
Jeon and Jeong (2015)	EXCLUDE on setting
Jessup et al. (2003)	EXCLUDE on setting
Jobe et al. (2001)	EXCLUDE on intervention
Johnen and Schott (2018)	EXCLUDE on study design
Johnson and Cockburn (1988)	EXCLUDE on study design
Joranson et al. (2017)	EXCLUDE on intervention
Jung et al. (2018)	EXCLUDE on intervention
Jyvakorpi et al. (2012)	EXCLUDE on intervention
Kamioka et al. (2004)	EXCLUDE on intervention
Kamioka et al. (2006)	EXCLUDE on intervention
Karlsson et al. (2013)	EXCLUDE on study design
Karmarkar (2009)	EXCLUDE on study design
Karmarkar (2009)	EXCLUDE on study design
Karmarkar et al. (2010)	EXCLUDE on study design
Katzel (2016)	EXCLUDE on setting
Kawagoshi et al. (2015)	EXCLUDE on setting
Keall et al. (2017)	EXCLUDE on intervention
Keeney et al. (2017)	EXCLUDE on study design
Kegelmeyer et al. (2013)	EXCLUDE on study design
Kelly et al. (2014)	EXCLUDE on intervention
Kemmler et al. (2010)	EXCLUDE on intervention
Kendall et al. (2018)	EXCLUDE on setting
Kerschan et al. (1998)	EXCLUDE on study design
Kerski et al. (1987)	EXCLUDE on study design
Kerwin et al. (2012)	EXCLUDE on study design
Kimura (2003)	EXCLUDE on intervention
Kind (2016)	EXCLUDE on intervention
King et al. (2012)	EXCLUDE on study design
Kiosses et al. (2010)	EXCLUDE on intervention
Kiosses et al. (2015)	EXCLUDE on study design
Kiosses et al. (2018)	EXCLUDE on study design
Kolt et al. (2009)	EXCLUDE on intervention
Konick‐McMahan et al. (2003)	EXCLUDE on study design
Konno (2012)	EXCLUDE on study design
Kono et al. (2009)	EXCLUDE on study design
Kravitz et al. (1994)	EXCLUDE on study design
Kruse et al. (2013)	EXCLUDE on study design
Kumar et al. (2017)	EXCLUDE on intervention
Kunik et al. (2017)	EXCLUDE on intervention
Kuo et al. (2013)	EXCLUDE on target group
Kuo et al. (2016)	EXCLUDE on target group
Kusumoto et al. (2007)	EXCLUDE on intervention
Lacroix et al. (2016)	EXCLUDE on setting
Lahtinen et al. (2015)	EXCLUDE on setting
Lahtinen et al. (2017)	EXCLUDE on setting
Lai (2004)	EXCLUDE on intervention
Lai (2017)	EXCLUDE on intervention
Lam et al. (2012)	EXCLUDE on setting
Laming (2017)	EXCLUDE on study design
Lapid et al. (2006)	EXCLUDE on setting
Laufer (2002)	EXCLUDE on setting
Leinonen et al. (2007)	EXCLUDE on intervention
Leone et al. (2012)	EXCLUDE on intervention
Leung (2005)	EXCLUDE on study design
Levine and Gitlin (1993)	EXCLUDE on study design
Levy‐Storms (2008)	EXCLUDE on study design
Liebel (2008)	EXCLUDE on study design
Lim et al. (2003)	EXCLUDE on setting
Lim et al. (2005)	EXCLUDE on target group
Lin et al. (2012)	EXCLUDE on setting
Lindegaard (2016)	EXCLUDE on study design
Lingler et al. (2014)	EXCLUDE on intervention
Littlewood et al. (2016)	EXCLUDE on intervention
Liu and Lai (2014)	EXCLUDE on setting
Liu and Lai (2014)	EXCLUDE on intervention
Liu et al. (2016)	EXCLUDE on intervention
Livingston et al. (2013)	EXCLUDE on target group
Lord et al. (2003)	EXCLUDE on setting
Low et al. (2013)	EXCLUDE on intervention
Low and Fletcher (2015)	EXCLUDE on study design
Luger et al. (2016)	EXCLUDE on intervention
Lundqvist et al. (2015)	EXCLUDE on study design
Luukinen et al. (2006)	EXCLUDE on intervention
MacNeil (2012)	EXCLUDE on intervention
Madara (2016)	EXCLUDE on target group
Maghsoudi et al. (2015)	EXCLUDE on study design
Majewski et al. (2015)	EXCLUDE on study design
Maki et al. (2008)	EXCLUDE on study design
Mameletzi et al. (2011)	EXCLUDE on setting
Mangione et al. (2010)	EXCLUDE on setting
Marioni et al. (2013)	EXCLUDE on intervention
Markle‐Reid et al. (2013)	EXCLUDE on study design
Marston (2007)	EXCLUDE on setting
Martini et al. (2018)	EXCLUDE on setting
Mason et al. (2007)	EXCLUDE on target group
Mazzuca et al. (1997)	EXCLUDE on intervention
McCusker et al. (2001)	EXCLUDE on setting
McEwan (1992)	EXCLUDE on study design
McMillan (2005)	EXCLUDE on study design
McNeil (1995)	EXCLUDE on study design
Mehlhorn et al. (2014)	EXCLUDE on setting
Mehrholz et al. (2015)	EXCLUDE on setting
Mehrholz (2017)	EXCLUDE on setting
Melin (1995)	EXCLUDE on setting
Melis et al. (2005)	EXCLUDE on intervention
Melis et al. (2010)	EXCLUDE on study design
Meng (2004)	EXCLUDE on intervention
Mercadante et al. (2011)	EXCLUDE on study design
Merom et al. (2016)	EXCLUDE on intervention
Messecar (1999)	EXCLUDE on study design
Messecar (2003)	EXCLUDE on study design
Michaud and Duchesne (2017)	EXCLUDE on setting
Mikkelsen et al. (2017)	EXCLUDE on study design
Miller et al. (2010)	EXCLUDE on study design
Miriam (2013)	EXCLUDE on study design
Mitchell (1987)	EXCLUDE on study design
Mitseva et al. (2012)	EXCLUDE on study design
Moffa‐Trotter and Anemaet (1996)	EXCLUDE on study design
Moholdt et al. (2012)	EXCLUDE on intervention
Molloy et al. (2006)	EXCLUDE on setting
Moniz (2012)	EXCLUDE on intervention
Monteau (2010)	EXCLUDE on study design
Montgomery (2010)	EXCLUDE on setting
Moore et al. (2016)	EXCLUDE on setting
Morag (2017)	EXCLUDE on intervention
Morey et al. (2015)	EXCLUDE on setting
Moriarty et al. (2016)	EXCLUDE on target group
Morin (2017)	EXCLUDE on setting
Mortenson et al. (2013)	EXCLUDE on intervention
Mottram et al. (2007)	EXCLUDE on study design
Mozley et al. (2007)	EXCLUDE on study design
Mozolic et al. (2008)	EXCLUDE on setting
Mukamel et al. (2007)	EXCLUDE on study design
Muller et al. (2014)	EXCLUDE on study design
Mustian et al. (2009)	EXCLUDE on intervention
Nelson et al. (2016)	EXCLUDE on study design
Ness et al. (2018)	EXCLUDE on setting
Neville (2015)	EXCLUDE on setting
Newbury and Marley (2000)	EXCLUDE on study design
Newcomer et al. (1999)	EXCLUDE on target group
Nguyen et al. (2015)	EXCLUDE on study design
Nieman et al. (2017)	EXCLUDE on intervention
Niemelä (2011)	EXCLUDE on setting
Niemela et al. (2012)	EXCLUDE on study design
Nijs et al. (2006)	EXCLUDE on intervention
Nikolaus et al. (1995)	EXCLUDE on intervention
Nikoletou et al. (2016)	EXCLUDE on intervention
Nilsson et al. (2012)	EXCLUDE on study design
Nishiguchi et al. (2015)	EXCLUDE on setting
Noelker and Bass (1989)	EXCLUDE on study design
Norrbom (1991)	EXCLUDE on intervention
Northey et al. (2018)	EXCLUDE on setting
Nour et al. (2002)	EXCLUDE on intervention
Nourhashemi (2015)	EXCLUDE on setting
Nowak et al. (1998)	EXCLUDE on target group
Oleske and Hauck (1988)	EXCLUDE on intervention
Oliveira et al. (2016)	EXCLUDE on setting
Ollonqvist et al. (2008)	EXCLUDE on setting
Olsen (2016)	EXCLUDE on setting
Orrell et al. (2007)	EXCLUDE on intervention
Orrell et al. (2014)	EXCLUDE on intervention
Ostaszkiewicz (2004)	EXCLUDE on intervention
Ottmann et al. (2013)	EXCLUDE on study design
Overbeek et al. (2018)	EXCLUDE on intervention
Padulo et al. (2018)	EXCLUDE on study design
Palvanen et al. (2012)	EXCLUDE on setting
Pan (2018)	EXCLUDE on setting
Park et al. (2017)	EXCLUDE on intervention
Parlevliet et al. (2010)	EXCLUDE on intervention
Parsons et al. (2013)	EXCLUDE on intervention
Pathy et al. (1992)	EXCLUDE on intervention
Payette et al. (2002)	EXCLUDE on intervention
Pearson et al. (2007)	EXCLUDE on intervention
Pedersen (2005)	EXCLUDE on study design
Perula et al. (2012)	EXCLUDE on setting
Pflaum et al. (2016)	EXCLUDE on study design
PG (1989)	EXCLUDE on study design
Pihl et al. (2011)	EXCLUDE on study design
Pine et al. (2002)	EXCLUDE on study design
Ploeg et al. (2010)	EXCLUDE on intervention
Podd et al. (2015)	EXCLUDE on setting
Pollack (1998)	EXCLUDE on study design
Pollock et al. (2012)	EXCLUDE on setting
Pozet et al. (2016)	EXCLUDE on target group
Pressler (2015)	EXCLUDE on study design
Prizer and Zimmerman (2018)	EXCLUDE on study design
Pynnonen et al. (2018)	EXCLUDE on setting
Ramsay et al. (2011)	EXCLUDE on intervention
Rana et al. (2010)	EXCLUDE on study design
Ranganathan et al. (2012)	EXCLUDE on study design
Rantanen et al. (2012)	EXCLUDE on setting
Ratzka (1986)	EXCLUDE on study design
Redford (1993)	EXCLUDE on study design
Reeder et al. (2013)	EXCLUDE on intervention
Reid and Ploeg (2002)	EXCLUDE on study design
Reid et al. (2017)	EXCLUDE on intervention
Rentschler et al. (2008)	EXCLUDE on study design
Resnick and Galik (2013)	EXCLUDE on study design
Ricauda et al. (2004)	EXCLUDE on setting
Richardson et al. (1989)	EXCLUDE on study design
Rideout (2004)	EXCLUDE on study design
Rizzo et al. (1996)	EXCLUDE on intervention
Robertson et al. (2001)	EXCLUDE on study design
Robinson et al. (2013)	EXCLUDE on setting
Rogers et al. (2018)	EXCLUDE on study design
Romskaug et al. (2017)	EXCLUDE on intervention
Rossi et al. (2014)	EXCLUDE on setting
Routasalo et al. (2009)	EXCLUDE on setting
Rubin et al. (1992)	EXCLUDE on setting
Rudilla et al. (2016)	EXCLUDE on intervention
Ruikes et al. (2016)	EXCLUDE on study design
Ryburn et al. (2009)	EXCLUDE on study design
Rydwik et al. (2010)	EXCLUDE on intervention
Sahota (2016)	EXCLUDE on setting
Sahyoun and Vaudin (2014)	EXCLUDE on study design
Sakurai et al. (2011)	EXCLUDE on setting
Sakurai et al. (2013)	EXCLUDE on setting
Salazar et al. (2000)	EXCLUDE on target group
Sampedro (2015)	EXCLUDE on intervention
Sampson et al. (2009)	EXCLUDE on intervention
Sanford et al. (1995)	EXCLUDE on intervention
Sayers et al. (2003)	EXCLUDE on setting
Schoenmakers et al. (2010)	EXCLUDE on target group
Schoonhoven et al. (2015)	EXCLUDE on intervention
Schwartz et al. (1990)	EXCLUDE on study design
Seitz et al. (2014)	EXCLUDE on study design
Sharpe et al. (2016)	EXCLUDE on setting
Shaw and Page (2008)	EXCLUDE on setting
Sheppard (1998)	EXCLUDE on target group
Shepperd and Iliffe (1998)	EXCLUDE on target group
Sherrington et al. (2004)	EXCLUDE on intervention
Sherwood (1980)	EXCLUDE on study design
Sherwood et al. (1986)	EXCLUDE on study design
Shimada et al. (2017)	EXCLUDE on intervention
Shishehgar et al. (2018)	EXCLUDE on intervention
Sidel et al. (1990)	EXCLUDE on intervention
Signe and Elmstahl (2008)	EXCLUDE on study design
Simmons et al. (1995)	EXCLUDE on study design
Simmons et al. (1996)	EXCLUDE on study design
Singh (2017)	EXCLUDE on setting
Singh (2018)	EXCLUDE on setting
Siragusa et al. (2005)	EXCLUDE on study design
Sjosten et al. (2008)	EXCLUDE on intervention
Sladek et al. (2011)	EXCLUDE on intervention
Smith et al. (2006)	EXCLUDE on study design
Sorensen et al. (2002)	EXCLUDE on target group
Sosnoff et al. (2014)	EXCLUDE on intervention
Sprange et al. (2013)	EXCLUDE on setting
Stark et al. (2017)	EXCLUDE on intervention
Stathi et al. (2018)	EXCLUDE on setting
Stein et al. (1981)	EXCLUDE on study design
Stelmack (2005)	EXCLUDE on intervention
Stenvall et al. (2007)	EXCLUDE on setting
Steultjens and Clemson (2006)	EXCLUDE on study design
Stevens‐Lapsley (2016)	EXCLUDE on setting
Stevenson and Gray (1981)	EXCLUDE on study design
Stewart et al. (2005)	EXCLUDE on intervention
Stewart (2006)	EXCLUDE on study design
Stewart et al. (2016)	EXCLUDE on study design
Stolle et al. (2012)	EXCLUDE on intervention
Stoltz (2004)	EXCLUDE on target group
Stow et al. (2015)	EXCLUDE on study design
Straw and Harley (1991)	EXCLUDE on study design
Stuck et al. (1995)	EXCLUDE on study design
Stuck et al. (2007)	EXCLUDE on intervention
Student (2013)	EXCLUDE on target group
Sturkenboom et al. (2016)	EXCLUDE on study design
Sungkarat et al. (2017)	EXCLUDE on setting
Suwanwela et al. (2002)	EXCLUDE on target group
Swank et al. (2011)	EXCLUDE on setting
Tappen and Debra (2014)	EXCLUDE on intervention
Tasiemski et al. (2005)	EXCLUDE on setting
Taule et al. (2015)	EXCLUDE on study design
Tennsledt et al. (1998)	EXCLUDE on intervention
Wolinksy (2011)	EXCLUDE on intervention
Thomas (1989)	EXCLUDE on study design
Thomas et al. (2007)	EXCLUDE on intervention
Thoreau (2015)	EXCLUDE on study design
Thulesius et al. (2002)	EXCLUDE on target group
Tibaek et al. (2014)	EXCLUDE on setting
Tiedemann et al. (2013)	EXCLUDE on setting
Tiedemann et al. (2015)	EXCLUDE on intervention
Tiedemann et al. (2016)	EXCLUDE on intervention
Timonen, Rantanen, Ryynanen, et al. (2002)	EXCLUDE on intervention
Timonen, Rantanen, Ryynanen, et al. (2002)	EXCLUDE on setting
Tinetti et al. (1993)	EXCLUDE on study design
Toevs (2000)	EXCLUDE on study design
Toot et al. (2011)	EXCLUDE on intervention
Torres et al. (2017)	EXCLUDE on intervention
Torres‐Arreola et al. (2009)	EXCLUDE on setting
Toseland et al. (1990)	EXCLUDE on target group
Towfighi et al. (2017)	EXCLUDE on target group
Troyer et al. (2010)	EXCLUDE on intervention
Tsai et al. (2017)	EXCLUDE on setting
Tse et al. (2016)	EXCLUDE on study design
Tsuchihashi‐Makaya et al. (2011)	EXCLUDE on target group
Ukawa, Satoh, et al. (2012)	EXCLUDE on intervention
Ukawa, Satoh, et al. (2012)	EXCLUDE on study design
Ukawa, Satoh, et al. (2012)	EXCLUDE on intervention
Ukawa (2015)	EXCLUDE on study design
Ullmann and Li (2017)	EXCLUDE on setting
Uy (2008)	EXCLUDE on setting
Vaapio et al. (2007)	EXCLUDE on setting
Vahlberg et al. (2017)	EXCLUDE on setting
van Ginneken et al. (2013)	EXCLUDE on target group
van Hout et al. (2005)	EXCLUDE on intervention
van Mulligen‐van (2013)	EXCLUDE on study design
van Ooijen et al. (2013)	EXCLUDE on setting
Van Spall (2018)	EXCLUDE on setting
Vandepitte, Noortgate, et al. (2016)	EXCLUDE on setting
VanDeVelde‐Coke (2004)	EXCLUDE on setting
Vass et al. (2004)	EXCLUDE on intervention
Vass (2005)	EXCLUDE on intervention
Venturelli et al. (2010)	EXCLUDE on setting
Verloo et al. (2015)	EXCLUDE on setting
Vetter et al. (1984)	EXCLUDE on intervention
Victor and Vetter (1988)	EXCLUDE on study design
Viswanathan et al. (2007)	EXCLUDE on study design
von Humboldt and Leal (2014)	EXCLUDE on intervention
Wallace et al. (2004)	EXCLUDE on intervention
Wanderley et al. (2015)	EXCLUDE on setting
Wang (2008)	EXCLUDE on study design
Wang et al. (2011)	EXCLUDE on intervention
Wang et al. (2013)	EXCLUDE on intervention
Wang et al. (2016)	EXCLUDE on target group
Wang and Wu (2017)	EXCLUDE on study design
Ward et al. (1978)	EXCLUDE on intervention
Warland and Tonning (1991)	EXCLUDE on study design
Warner et al. (2016)	EXCLUDE on intervention
Waterman et al. (2016)	EXCLUDE on study design
Weiss et al. (2004)	EXCLUDE on study design
Whitehead, Walker, et al. (2016)	EXCLUDE on study design
Whitehead, Walker, et al. (2016)	EXCLUDE on intervention
Whittemore et al. (2014)	EXCLUDE on study design
Whitten and Mickus (2007)	EXCLUDE on intervention
Wilhelmson et al. (2011)	EXCLUDE on intervention
Williams (2013)	EXCLUDE on study design
Williams et al. (2015)	EXCLUDE on study design
Wilson et al. (1999)	EXCLUDE on setting
Wilson and Truman (2005)	EXCLUDE on study design
Winter et al. (2016)	EXCLUDE on target group
Wittwer (2016)	EXCLUDE on intervention
Wong et al. (2014)	EXCLUDE on setting
Wong (2015)	EXCLUDE on study design
Wong et al. (2018)	EXCLUDE on setting
Wongcharoen et al. (2017)	EXCLUDE on intervention
Woolrych (2016)	EXCLUDE on study design
Wu (2007)	EXCLUDE on setting
Wu (2008)	EXCLUDE on target group
Wu (2017)	EXCLUDE on intervention
Wyatt et al. (2004)	EXCLUDE on target group
Wyers et al. (2010)	EXCLUDE on intervention
Xueyu et al. (2017)	EXCLUDE on intervention
Yoon et al. (2018)	EXCLUDE on setting
Yu (2016)	EXCLUDE on setting
Yusif et al. (2016)	EXCLUDE on study design
Yu‐Yahiro et al. (2009)	EXCLUDE on study design
Zarit et al. (2017)	EXCLUDE on study design
Zeeuwe et al. (2006)	EXCLUDE on intervention
Zhang et al. (2014)	EXCLUDE on setting
Zhao et al. (2017)	EXCLUDE on study design
Zheng et al. (2016)	EXCLUDE on setting
Zimmer (1984)	EXCLUDE on study design
Zimmer et al. (1990)	EXCLUDE on study design

## SOURCES OF SUPPORT

### Internal sources

- No sources of support provided

### External sources

- World Health Organization (WHO)
- Canadian Institute of Health Research (CIHR), Canada

## DATA AND ANALYSES

## Supporting information

Supporting information

Supporting information

Supporting information

## APPENDIX 1 AGELINE SEARCH STRATEGY

**Table:**

#	Query	Limiters/expanders	Last run via	Results
S117	S114 AND S115 AND S116	Search modes—Boolean/Phrase	Interface—EBSCOhost Research Databases Search Screen—Advanced Search Database—AgeLine	1620
S116	S97 OR S98 OR S99 OR S100 OR S101 OR S102 OR S103 OR S104 OR S105 OR S106 OR S107 OR S108 OR S109 OR S110 OR S111 OR S112	Search modes—Boolean/Phrase	Interface—EBSCOhost Research Databases Search Screen—Advanced Search Database—AgeLine	50,309
S115	S63 OR S64 OR S65 OR S66 OR S67 OR S68 OR S69 OR S70 OR S71 OR S72 OR S73 OR S74 OR S75 OR S76 OR S77 OR S78 OR S79 OR S80 OR S81 OR S82 OR S83 OR S84 OR S85 OR S86 OR S87 OR S88 OR S89 OR S90 OR S91 OR S92 OR S93 OR S94 OR S95 OR S96	Search modes—Boolean/Phrase	Interface—EBSCOhost Research Databases Search Screen—Advanced Search Database—AgeLine	76,717
S114	S13 OR S14 OR S15 OR S16 OR S17 OR S18 OR S19 OR S20 OR S21 OR S22 OR S23 OR S24 OR S25 OR S26 OR S27 OR S28 OR S29 OR S30 OR S31 OR S32 OR S33 OR S34 OR S35 OR S36 OR S37 OR S38 OR S39 OR S40 OR S41 OR S42 OR S43 OR S44 OR S45 OR S46 OR S47 OR S48 OR S49 OR S50 OR S51 OR S52 OR S53 OR S54 OR S55 OR S56 OR S57 OR S58 OR S59 OR S60 OR S61 OR S62	Search modes—Boolean/Phrase	Interface—EBSCOhost Research Databases Search Screen—Advanced Search Database—AgeLine	9488
S113	S1 OR S2 OR S3 OR S4 OR S5 OR S6 OR S7 OR S8 OR S9 OR S10 OR S11 OR S12	Search modes—Boolean/Phrase	Interface—EBSCOhost Research Databases Search Screen—Advanced Search Database—AgeLine	105,340
S112	AB "usual care" or "usual practices"	Search modes—Boolean/Phrase	Interface—EBSCOhost Research Databases Search Screen—Advanced Search Database—AgeLine	472
S111	AB groups	Search modes—Boolean/Phrase	Interface—EBSCOhost Research Databases Search Screen—Advanced Search Database—AgeLine	36,075
S110	TI trial OR AB trial	Search modes—Boolean/Phrase	Interface—EBSCOhost Research Databases Search Screen—Advanced Search Database—AgeLine	4922
S109	TI randomly OR AB randomly	Search modes—Boolean/Phrase	Interface—EBSCOhost Research Databases Search Screen—Advanced Search Database—AgeLine	2580
S108	TI randomized OR AB randomized	Search modes—Boolean/Phrase	Interface—EBSCOhost Research Databases Search Screen—Advanced Search Database—AgeLine	2866
S107	TI controlled N study OR AB controlled N2 study	Search modes—Boolean/Phrase	Interface——EBSCOhost Research Databases Search Screen—Advanced Search Database—AgeLine	347
S106	DE "Randomized Controlled Trials" OR DE "Controlled Clinical Trials"	Search modes—Boolean/Phrase	Interface—EBSCOhost Research Databases Search Screen—Advanced Search Database—AgeLine	2002
S105	AB medline	Search modes—Boolean/Phrase	Interface—EBSCOhost Research Databases Search Screen—Advanced Search Database—AgeLine	745
S104	AB pubmed	Search modes—Boolean/Phrase	Interface—EBSCOhost Research Databases Search Screen—Advanced Search Database—AgeLine	363
S103	TI overview OR AB overview	Search modes—Boolean/Phrase	Interface—EBSCOhost Research Databases Search Screen—Advanced Search Database—AgeLine	3185
S102	TI evidence N2 synthesi* OR AB evidence N2 synthesi*	Search modes—Boolean/Phrase	Interface—EBSCOhost Research Databases Search Screen—Advanced Search Database—AgeLine	44
S101	TI review	Search modes—Boolean/Phrase	Interface—EBSCOhost Research Databases Search Screen—Advanced Search Database—AgeLine	2612
S100	AB review* and (literature or studies or trials)	Search modes—Boolean/Phrase	Interface—EBSCOhost Research Databases Search Screen—Advanced Search Database—AgeLine	8168
S99	TI (meta‐analysis or metaanalysis) OR AB (meta‐analysis or metaanalysis)	Search modes—Boolean/Phrase	Interface—EBSCOhost Research Databases Search Screen—Advanced Search Database—AgeLine	697
S98	TI (meta‐analysis or metaanalysis) OR (meta‐analysis or metaanalysis)	Search modes—Boolean/Phrase	Interface—EBSCOhost Research Databases Search Screen—Advanced Search Database—AgeLine	870
S97	TI systematic* OR AB systematic*	Search modes—Boolean/Phrase	Interface—EBSCOhost Research Databases Search Screen—Advanced Search Database—AgeLine	2823
S96	TI institutionali?ation OR AB institutionali?ation	Search modes—Boolean/Phrase	Interface—EBSCOhost Research Databases Search Screen—Advanced Search Database—AgeLine	1701
S95	TI reablement OR AB reablement	Search modes—Boolean/Phrase	Interface—EBSCOhost Research Databases Search Screen—Advanced Search Database—AgeLine	8
S94	TI adherence OR AB adherence	Search modes—Boolean/Phrase	Interface—EBSCOhost Research Databases Search Screen—Advanced Search Database—AgeLine	1010
S93	TI satisfaction OR AB satisfaction	Search modes—Boolean/Phrase	Interface—EBSCOhost Research Databases Search Screen—Advanced Search Database—AgeLine	6127
S92	TI relationships OR AB relationships	Search modes—Boolean/Phrase	Interface—EBSCOhost Research Databases Search Screen—Advanced Search Database—AgeLine	20,928
S91	TI security OR AB security	Search modes—Boolean/Phrase	Interface—EBSCOhost Research Databases Search Screen—Advanced Search Database—AgeLine	9802
S90	TI "community life" OR AB "community life"	Search modes—Boolean/Phrase	Interface—EBSCOhost Research Databases Search Screen—Advanced Search Database—AgeLine	106
S89	TI mobility OR AB mobility	Search modes—Boolean/Phrase	Interface—EBSCOhost Research Databases Search Screen—Advanced Search Database—AgeLine	3638
S88	TI "self efficacy" OR AB "self efficacy"	Search modes—Boolean/Phrase	Interface—EBSCOhost Research Databases Search Screen—Advanced Search Database—AgeLine	1155
S87	TI "self care" OR AB "self care"	Search modes—Boolean/Phrase	Interface—EBSCOhost Research Databases Search Screen—Advanced Search Database—AgeLine	1221
S86	TI tiredness OR AB tiredness	Search modes—Boolean/Phrase	Interface—EBSCOhost Research Databases Search Screen—Advanced Search Database—AgeLine	72
S85	TI fatigue OR AB fatigue	Search modes—Boolean/Phrase	Interface—EBSCOhost Research Databases Search Screen—Advanced Search Database—AgeLine	678
S84	TI energy OR AB energy	Search modes—Boolean/Phrase	Interface—EBSCOhost Research Databases Search Screen—Advanced Search Database—AgeLine	1789
S83	TI vitality OR AB vitality	Search modes—Boolean/Phrase	Interface—EBSCOhost Research Databases Search Screen—Advanced Search Database—AgeLine	391
S82	TI distress OR AB distress	Search modes—Boolean/Phrase	Interface—EBSCOhost Research Databases Search Screen—Advanced Search Database—AgeLine	1763
S81	TI pain OR AB pain	Search modes—Boolean/Phrase	Interface—EBSCOhost Research Databases Search Screen—Advanced SearchDatabase—AgeLine	3778
S80	TI "sensory function*" OR AB "sensory function*"	Search modes—Boolean/Phrase	Interface—EBSCOhost Research Databases Search Screen—Advanced Search Database—AgeLine	91
S79	TI cognitive OR AB cognitive	Search modes—Boolean/Phrase	Interface—EBSCOhost Research Databases Search Screen—Advanced Search Database—AgeLine	16,478
S78	TI depression OR AB depression	Search modes—Boolean/Phrase	Interface—EBSCOhost Research Databases Search Screen—Advanced Search Database—AgeLine	12,698
S77	TI "functional ability" OR AB "functional ability"	Search modes—Boolean/Phrase	Interface—EBSCOhost Research Databases Search Screen—Advanced Search Database—AgeLine	865
S76	TI "mental health" OR AB "mental health"	Search modes—Boolean/Phrase	Interface—EBSCOhost Research Databases Search Screen—Advanced Search Database—AgeLine	6757
S75	TI (happiness or happier) OR AB (happiness or happier)	Search modes—Boolean/Phrase	Interface—EBSCOhost Research Databases Search Screen—Advanced Search Database—AgeLine	755
S74	TI "social participation" OR AB "social participation"	Search modes—Boolean/Phrase	Interface—EBSCOhost Research Databases Search Screen—Advanced Search Database—AgeLine	315
S73	TI "social life" OR AB "social life"	Search modes—Boolean/Phrase	Interface—EBSCOhost Research Databases Search Screen—Advanced Search Database—AgeLine	343
S72	TI wellbeing OR AB wellbeing	Search modes—Boolean/Phrase	Interface—EBSCOhost Research Databases Search Screen—Advanced Search Database—AgeLine	328
S71	TI independence OR AB independence	Search modes—Boolean/Phrase	Interface—EBSCOhost Research Databases Search Screen—Advanced Search Database—AgeLine	3181
S70	TI "quality of life" OR AB "quality of life"	Search modes—Boolean/Phrase	Interface—EBSCOhost Research Databases Search Screen—Advanced Search Database—AgeLine	6388
S69	TI "activities of daily living" OR AB "activities of daily living"	Search modes—Boolean/Phrase	Interface—EBSCOhost Research Databases Search Screen—Advanced Search Database—AgeLine	4863
S68	DE "Quality of Life"	Search modes—Boolean/Phrase	Interface—EBSCOhost Research Databases Search Screen—Advanced Search Database—AgeLine	5646
S67	DE "Functional Ability"	Search modes—Boolean/Phrase	Interface—EBSCOhost Research Databases Search Screen—Advanced Search Database—AgeLine	3318
S66	DE "Leisure Activities" OR DE "Games" OR DE "Hobbies" OR DE "Reading" OR DE "Recreation" OR DE "Travel"	Search modes—Boolean/Phrase	Interface—EBSCOhost Research Databases Search Screen—Advanced Search Database—AgeLine	3271
S65	DE "Instrumental Activities of Daily Living"	Search modes—Boolean/Phrase	Interface—EBSCOhost Research Databases Search Screen—Advanced Search Database—AgeLine	676
S64	DE "Daily Activities"	Search modes—Boolean/Phrase	Interface—EBSCOhost Research Databases Search Screen—Advanced Search Database—AgeLine	394
S63	DE "Activities of Daily Living"	Search modes—Boolean/Phrase	Interface—EBSCOhost Research Databases Search Screen—Advanced Search Database—AgeLine	3306
S62	TI ((kitchen or bathroom or bedroom)) OR AB ((kitchen or bathroom or bedroom))	Search modes—Boolean/Phrase	Interface—EBSCOhost Research Databases Search Screen—Advanced Search Database—AgeLine	662
S61	TI (medication N2 reminders) OR AB (medication N2 reminders)	Search modes—Boolean/Phrase	Interface—EBSCOhost Research Databases Search Screen—Advanced Search Database—AgeLine	21
S60	TI "foot care" OR AB "foot care"	Search modes—Boolean/Phrase	Interface—EBSCOhost Research Databases Search Screen—Advanced Search Database—AgeLine	56
S59	TI toileting OR AB toileting	Search modes—Boolean/Phrase	Interface—EBSCOhost Research Databases Search Screen—Advanced Search Database—AgeLine	268
S58	TI "personal hygiene" OR AB "personal hygiene"	Search modes—Boolean/Phrase	Interface—EBSCOhost Research Databases Search Screen—Advanced Search Database—AgeLine	85
S57	TI grooming OR AB grooming	Search modes—Boolean/Phrase	Interface—EBSCOhost Research Databases Search Screen—Advanced Search Database—AgeLine	151
S56	TI bathing OR AB bathing	Search modes—Boolean/Phrase	Interface—EBSCOhost Research Databases Search Screen—Advanced Search Database—AgeLine	425
S55	TI (((household or routine) N (jobs or tasks or chores))) OR AB (((household or routine) N (jobs or tasks or chores)))	Search modes—Boolean/Phrase	Interface—EBSCOhost Research Databases Search Screen—Advanced Search Database—AgeLine	117
S54	TI housekeeping OR AB housekeeping	Search modes—Boolean/Phrase	Interface—EBSCOhost Research Databases Search Screen—Advanced Search Database—AgeLine	294
S53	TI homemaking OR AB homemaking	Search modes—Boolean/Phrase	Interface—EBSCOhost Research Databases Search Screen—Advanced Search Database—AgeLine	91
S52	TI ((meal* N3 (provision or assistance or help or service* or preparation or delivery))) OR AB ((meal* N3 (provision or assistance or help or service* or preparation or delivery)))	Search modes—Boolean/Phrase	Interface—EBSCOhost Research Databases Search Screen—Advanced Search Database—AgeLine	575
S51	TI ((food N (preparation or assistance or help or service or delivery))) OR AB ((food N (preparation or assistance or help or service or delivery)))	Search modes—Boolean/Phrase	Interface—EBSCOhost Research Databases Search Screen—Advanced Search Database—AgeLine	4
S50	TI "house help" OR AB "house help"	Search modes—Boolean/Phrase	Interface—EBSCOhost Research Databases Search Screen—Advanced Search Database—AgeLine	5
S49	TI shopping OR AB shopping	Search modes—Boolean/Phrase	Interface—EBSCOhost Research Databases Search Screen—Advanced Search Database—AgeLine	873
S48	TI "community services" OR AB "community services"	Search modes—Boolean/Phrase	Interface—EBSCOhost Research Databases Search Screen—Advanced Search Database—AgeLine	949
S47	TI "home visit*" OR AB "home visit*"	Search modes—Boolean/Phrase	Interface—EBSCOhost Research Databases Search Screen—Advanced Search Database—AgeLine	630
S46	TI "home support service*" OR AB "home support service*"	Search modes—Boolean/Phrase	Interface—EBSCOhost Research Databases Search Screen—Advanced Search Database—AgeLine	44
S45	TI "home care service*" OR AB "home care service*"	Search modes—Boolean/Phrase	Interface—EBSCOhost Research Databases Search Screen—Advanced Search Database—AgeLine	668
S44	DE "Home Care"	Search modes—Boolean/Phrase	Interface—EBSCOhost Research Databases Search Screen—Advanced Search Database—AgeLine	3133
S43	TI (ramp or ramps) OR AB (ramp or ramps)	Search modes—Boolean/Phrase	Interface—EBSCOhost Research Databases Search Screen—Advanced Search Database—AgeLine	66
S42	TI "shallow steps" OR AB "shallow steps"	Search modes—Boolean/Phrase	Interface—EBSCOhost Research Databases Search Screen—Advanced Search Database—AgeLine	1
S41	TI "stair rails" OR AB "stair rails"	Search modes—Boolean/Phrase	Interface—EBSCOhost Research Databases Search Screen—Advanced Search Database—AgeLine	2
S40	TI stairs OR AB stairs	Search modes—Boolean/Phrase	Interface—EBSCOhost Research Databases Search Screen—Advanced Search Database—AgeLine	330
S39	TI "stair climbing" OR AB "stair climbing"	Search modes—Boolean/Phrase	Interface—EBSCOhost Research Databases Search Screen—Advanced Search Database—AgeLine	46
S38	TI Stair lift*" OR AB "Stair lift*"	Search modes—Boolean/Phrase	Interface—EBSCOhost Research Databases Search Screen—Advanced Search Database—AgeLine	5
S37	TI (((Adapt* or adjust*) N3 (door* or entry or exit))) OR AB (((Adapt* or adjust*) N3 (door* or entry or exit)))	Search modes—Boolean/Phrase	Interface—EBSCOhost Research Databases Search Screen—Advanced Search Database—AgeLine	12
S36	TI "vision aid*" OR AB "vision aid*"	Search modes—Boolean/Phrase	Interface—EBSCOhost Research Databases Search Screen—Advanced Search Database—AgeLine	18
S35	TI "hearing aid*" OR AB "hearing aid*"	Search modes—Boolean/Phrase	Interface—EBSCOhost Research Databases Search Screen—Advanced Search Database—AgeLine	317
S34	TI "hearing device*" OR AB "hearing device*"	Search modes—Boolean/Phrase	Interface—EBSCOhost Research Databases Search Screen—Advanced Search Database—AgeLine	11
S33	TI spectacles OR AB spectacles	Search modes—Boolean/Phrase	Interface—EBSCOhost Research Databases Search Screen—Advanced Search Database—AgeLine	20
S32	TI glasses OR AB glasses	Search modes—Boolean/Phrase	Interface—EBSCOhost Research Databases Search Screen—Advanced Search Database—AgeLine	104
S31	TI eyeglasses OR AB eyeglasses	Search modes—Boolean/Phrase	Interface—EBSCOhost Research Databases Search Screen—Advanced Search Database—AgeLine	61
S30	DE "Hearing Aids"	Search modes—Boolean/Phrase	Interface—EBSCOhost Research Databases Search Screen—Advanced Search Database—AgeLine	214
S29	TI ((communication N (aid* or device*))) OR AB ((communication N (aid* or device*)))	Search modes—Boolean/Phrase	Interface—EBSCOhost Research Databases Search Screen—Advanced Search Database—AgeLine	1
S28	TI "transfer device*" OR AB "transfer device*"	Search modes—Boolean/Phrase	Interface—EBSCOhost Research Databases Search Screen—Advanced Search Database—AgeLine	5
S27	TI scooter* OR AB scooter*	Search modes—Boolean/Phrase	Interface—EBSCOhost Research Databases Search Screen—Advanced Search Database—AgeLine	22
S26	TI Wheelchair* OR AB Wheelchair*	Search modes—Boolean/Phrase	Interface—EBSCOhost Research Databases Search Screen—Advanced Search Database—AgeLine	435
S25	TI ((Adapt* N3 (home* or house*))) OR AB ((Adapt* N3 (home* or house*)))	Search modes—Boolean/Phrase	Interface—EBSCOhost Research Databases Search Screen—Advanced Search Database—AgeLine	168
S24	TI ((Adapt* N3 (cars or transport or vehicles))) OR AB ((Adapt* N3 (cars or transport or vehicles)))	Search modes—Boolean/Phrase	Interface—EBSCOhost Research Databases Search Screen—Advanced Search Database—AgeLine	13
S23	TI "walking stick*" OR AB "walking stick*"	Search modes—Boolean/Phrase	Interface—EBSCOhost Research Databases Search Screen—Advanced Search Database—AgeLine	8
S22	TI crutches OR AB crutches	Search modes—Boolean/Phrase	Interface—EBSCOhost Research Databases Search Screen—Advanced Search Database—AgeLine	20
S21	TI cane* OR AB cane*	Search modes—Boolean/Phrase	Interface—EBSCOhost Research Databases Search Screen—Advanced Search Database—AgeLine	140
S20	TI ((walking N2 (device* or aid* or equipment))) OR AB ((walking N2 (device* or aid* or equipment)))	Search modes—Boolean/Phrase	Interface—EBSCOhost Research Databases Search Screen—Advanced Search Database—AgeLine	79
S19	TI motility OR AB motility	Search modes—Boolean/Phrase	Interface—EBSCOhost Research Databases Search Screen—Advanced Search Database—AgeLine	16
S18	TI "mobility aid*" OR AB "mobility aid*"	Search modes—Boolean/Phrase	Interface—EBSCOhost Research Databases Search Screen—Advanced Search Database—AgeLine	26
S17	TI "mobility device*" OR AB "mobility device*"	Search modes—Boolean/Phrase	Interface—EBSCOhost Research Databases Search Screen—Advanced Search Database—AgeLine	34
S16	TI "mobility equipment" OR AB "mobility equipment"	Search modes—Boolean/Phrase	Interface—EBSCOhost Research Databases Search Screen—Advanced Search Database—AgeLine	5
S15	TI "assistive equipment" OR AB "assistive equipment"	Search modes—Boolean/Phrase	Interface—EBSCOhost Research Databases Search Screen—Advanced Search Database—AgeLine	9
S14	TI "assistive devices" OR AB "assistive devices"	Search modes—Boolean/Phrase	Interface—EBSCOhost Research Databases Search Screen—Advanced Search Database—AgeLine	284
S13	DE "Assistive Devices"	Search modes—Boolean/Phrase	Interface—EBSCOhost Research Databases Search Screen—Advanced Search Database—AgeLine	751
S12	TI ((veteran* and (old* or home* or retire*))) OR AB ((veteran* and (old* or home* or retire*)))	Search modes—Boolean/Phrase	Interface—EBSCOhost Research Databases Search Screen—Advanced Search Database—AgeLine	1576
S11	TI geriatric* OR AB geriatric*	Search modes—Boolean/Phrase	Interface—EBSCOhost Research Databases Search Screen—Advanced Search Database—AgeLine	16,521
S10	TI ((Resident* and (old* or home* or retirement or nursing))) OR AB ((Resident* and (old* or home* or retirement or nursing)))	Search modes—Boolean/Phrase	Interface—EBSCOhost Research Databases Search Screen—Advanced Search Database—AgeLine	14,367
S9	TI "end of life" OR AB "end of life"	Search modes—Boolean/Phrase	Interface—EBSCOhost Research Databases Search Screen—Advanced Search Database—AgeLine	2172
S8	TI retirement OR AB retirement	Search modes—Boolean/Phrase	Interface—EBSCOhost Research Databases Search Screen—Advanced Search Database—AgeLine	16,495
S7	TI pensioners OR AB pensioners	Search modes—Boolean/Phrase	Interface—EBSCOhost Research Databases Search Screen—Advanced Search Database—AgeLine	286
S6	TI old* age* OR AB old* age*	Search modes—Boolean/Phrase	Interface—EBSCOhost Research Databases Search Screen—Advanced Search Database—AgeLine	34,385
S5	TI "older women" OR AB "older women"	Search modes—Boolean/Phrase	Interface—EBSCOhost Research Databases Search Screen—Advanced Search Database—AgeLine	3854
S4	TI "older men" OR AB "older men"	Search modes—Boolean/Phrase	Interface—EBSCOhost Research Databases Search Screen—Advanced Search Database—AgeLine	1695
S3	TI "older adult*" OR AB "older adult*"	Search modes—Boolean/Phrase	Interface—EBSCOhost Research Databases Search Screen—Advanced Search Database—AgeLine	37,102
S2	TI "older people" OR AB "older people"	Search modes—Boolean/Phrase	Interface—EBSCOhost Research Databases Search Screen—Advanced Search Database—AgeLine	9585
S1	TI elderly OR AB elderly	Search modes—Boolean/Phrase	Interface—EBSCOhost Research Databases Search Screen—Advanced Search Database—AgeLine	32,088

## APPENDIX 2 ASSIA SEARCH STRATEGY

(MAINSUBJECT.EXACT("Elderly people") OR MAINSUBJECT.EXACT("Elderly people") OR ti(elderly OR "older people" OR "older men" OR older women " or " older age " or " old age " or pensioners or retirement or " end of life " or geriatric") OR ab(elderly OR "older people" OR "older men" OR older women " or " older age " or " old age " or pensioners or retirement or " end of life " or geriatric") OR ti((Resident* AND (old* OR home* OR retirement OR nursing))) OR ti((Resident* AND (old* OR home* OR retirement OR nursing))) OR ab((veteran* AND (old* OR home* OR retire*)))) AND ((ti,ab("assistive devices" or "assistive equipment" or "mobility equipment" or "mobility devices" or "mobility aid*" or motility) OR ti,ab(walking near/2 (device* or aid* or equipment)) OR ti,ab(cane* or crutch* or "walking stick" or wheelchair* or scooter) OR ti,ab(adapt* near/3 (cars or transport or vehicles or home* or house*)) OR ti,ab(communication near/2 (device* or aid* or equipment)) OR ti,ab("hearing aid*" or hearing device* or eyeglasses or glasses or spectacles or "vision aid*") OR ti,ab((adapt* or adjust) near/3 (door* or entry or exit)) OR ti,ab(stair*) OR ti,ab(ramp or ramps) OR MAINSUBJECT.EXACT.EXPLODE("Home care")) OR (ti("home care" OR "home support" OR home visit " or " community services " or shopping or " house help " or " home help " or homemaking or housekeeping or bathing or grooming or " personal hygiene " or toileting or " footcare "") OR ab("home care" OR "home support" OR home visit " or " community services " or shopping or " house help " or " home help " or homemaking or housekeeping or bathing or grooming or " personal hygiene " or toileting or " footcare "")) OR ti,ab(food near (preparation or assistance or help or service or delivery)) OR ti,ab(meal* near/3 (provision or preparation or assistance or help or service or delivery)) OR ti,ab(kitchen or bathroom or bedroom))

## APPENDIX 3 CINAHL SEARCH STRATEGY

**Table:**

	Query	Results
S122	S16 AND S68 AND S103 AND S121	1,915
S121	S104 OR S105 OR S106 OR S107 OR S108 OR S109 OR S110 OR S111 OR S112 OR S113 OR S114 OR S115 OR S116 OR S117 OR S118 OR S119 OR S120	1,059,185
S120	AB "usual care" or "usual practices"	7,133
S119	AB groups	535,924
S118	TI trial OR AB trial	241,739
S117	TI randomly OR AB randomly	66,660
S116	TI randomized OR AB randomized	147,898
S115	TI controlled N study OR AB controlled N2 study	19,862
S114	(MH "Randomized Controlled Trials")	75,376
S113	AB medline	34,544
S112	AB pubmed	27,376
S111	TI overview OR AB overview	36,219
S110	TI evidence N2 synthesi* OR AB evidence N2 synthesi*	2,611
S109	TI review	147,301
S108	AB review* and (literature or studies or trials)	230,546
S107	TI (meta‐analysis or metaanalysis) OR AB (meta‐analysis or metaanalysis)	47,271
S106	TI (meta‐analysis or metaanalysis) OR (meta‐analysis or metaanalysis)	59,045
S105	TI systematic* OR AB systematic*	111,442
S104	(MH "Systematic Review")	65,382
S103	(S69 OR S70 OR S71 OR S72 OR S73 OR S74 OR S75 OR S76 OR S77 OR S78 OR S79 OR S80 OR S81 OR S82 OR S83 OR S84 OR S85 OR S86 OR S87 OR S88 OR S89 OR S90 OR S91 OR S92 OR S93 OR S94 OR S95 OR S96 OR S97 OR S98 OR S99 OR S100 OR S101 OR S102)	899,122
S102	TI institutionali?ation OR AB institutionali?ation	1,956
S101	TI reablement OR AB reablement	59
S100	TI adherence OR AB adherence	34,819
S99	TI satisfaction OR AB satisfaction	58,116
S98	TI relationships OR AB relationships	221,114
S97	TI security OR AB security	14,091
S96	TI "community life" OR AB "community life"	293
S95	TI mobility OR AB mobility	20,472
S94	TI "self efficacy" OR AB "self efficacy"	16,170
S93	TI "self care" OR AB "self care"	12,062
S92	TI tiredness OR AB tiredness	1,133
S91	TI fatigue OR AB fatigue	28,761
S90	TI energy OR AB energy	45,433
S89	TI vitality OR AB vitality	2,603
S88	TI distress OR AB distress	37,058
S87	TI pain OR AB pain	191,463
S86	TI "sensory function*" OR AB "sensory function*"	671
S85	TI cognitive OR AB cognitive	96,135
S84	TI depression OR AB depression	92,125
S83	TI "functional ability" OR AB "functional ability"	2,170
S82	TI "mental health" OR AB "mental health"	78,164
S81	TI (happiness or happier) OR AB (happiness or happier)	3,585
S80	TI "social participation" OR AB "social participation"	1,462
S79	TI "social life" OR AB "social life"	1,416
S78	TI wellbeing OR AB wellbeing	8,393
S77	TI independence OR AB independence	13,126
S76	TI "quality of life" OR AB "quality of life"	90,744
S75	TI "activities of daily living" OR AB "activities of daily living"	11,299
S74	(MM "Automobile Driving")	5,878
S73	(MM "Quality of Life + ")	45,014
S72	(MM "Human Activities")	619
S71	(MM "Home Maintenance")	751
S70	(MM "Leisure Activities")	3,321
S69	(MH "Activities of Daily Living + ")	55,234
S68	S17 OR S18 OR S19 OR S20 OR S21 OR S22 OR S23 OR S24 OR S25 OR S26 OR S27 OR S28 OR S29 OR S30 OR S31 OR S32 OR S33 OR S34 OR S35 OR S36 OR S37 OR S38 OR S39 OR S40 OR S41 OR S42 OR S43 OR S44 OR S45 OR S46 OR S47 OR S48 OR S49 OR S50 OR S51 OR S52 OR S53 OR S54 OR S55 OR S56 OR S57 OR S58 OR S59 OR S60 OR S61 OR S62 OR S63 OR S64 OR S65 OR S66 OR S67	53,724
S67	TI ((kitchen or bathroom or bedroom)) OR AB ((kitchen or bathroom or bedroom))	2,693
S66	TI (medication N2 reminders) OR AB (medication N2 reminders)	141
S65	TI "foot care" OR AB "foot care"	1,048
S64	TI toileting OR AB toileting	543
S63	TI "personal hygiene" OR AB "personal hygiene"	503
S62	TI grooming OR AB grooming	458
S61	TI bathing OR AB bathing	1,784
S60	TI (((household or routine) N (jobs or tasks or chores))) OR AB (((household or routine) N (jobs or tasks or chores)))	5
S59	TI housekeeping OR AB housekeeping	662
S58	TI homemaking OR AB homemaking	64
S57	TI ((meal* N3 (provision or assistance or help or service* or preparation or delivery))) OR AB ((meal* N3 (provision or assistance or help or service* or preparation or delivery)))	674
S56	TI ((food N (preparation or assistance or help or service or delivery))) OR AB ((food N (preparation or assistance or help or service or delivery)))	18
S55	TI "house help" OR AB "house help"	2
S54	TI shopping OR AB shopping	2,202
S53	TI "community services" OR AB "community services"	1,494
S52	TI "home visit*" OR AB "home visit*"	4,651
S51	TI "home support service*" OR AB "home support service*"	43
S50	TI "home care service*" OR AB "home care service*"	1,128
S49	(MM "Home Care Equipment and Supplies")	247
S48	(MH "Home Rehabilitation + ")	1,802
S47	TI (ramp or ramps) OR AB (ramp or ramps)	1,036
S46	TI "shallow steps" OR AB "shallow steps"	12
S45	TI "stair rails" OR AB "stair rails"	2
S44	TI stairs OR AB stairs	2,309
S43	TI "stair climbing" OR AB "stair climbing"	671
S42	TI Stair lift*" OR AB "Stair lift*"	12
S41	TI (((Adapt* or adjust*) N3 (door* or entry or exit))) OR AB (((Adapt* or adjust*) N3 (door* or entry or exit)))	126
S40	TI "vision aid*" OR AB "vision aid*"	53
S39	TI "hearing aid*" OR AB "hearing aid*"	4,815
S38	TI "hearing device*" OR AB "hearing device*"	296
S37	TI spectacles OR AB spectacles	505
S36	TI glasses OR AB glasses	885
S35	TI eyeglasses OR AB eyeglasses	121
S34	(MH "Hearing Aids + ")	14,658
S33	TI ((communication N (aid* or device*))) OR AB ((communication N (aid* or device*)))	3
S32	TI "transfer device*" OR AB "transfer device*"	102
S31	TI scooter* OR AB scooter*	231
S30	TI Wheelchair* OR AB Wheelchair*	4,427
S29	TI ((Adapt* N3 (home* or house*))) OR AB ((Adapt* N3 (home* or house*)))	514
S28	TI ((Adapt* N3 (cars or transport or vehicles))) OR AB ((Adapt* N3 (cars or transport or vehicles)))	79
S27	TI "walking stick*" OR AB "walking stick*"	70
S26	TI crutches OR AB crutches	398
S25	TI cane* OR AB cane*	870
S24	TI ((walking N2 (device* or aid* or equipment))) OR AB ((walking N2 (device* or aid* or equipment)))	682
S23	TI motility OR AB motility	3,562
S22	TI "mobility aid*" OR AB "mobility aid*"	Display
S21	TI "mobility device*" OR AB "mobility device*"	Display
S20	TI "mobility equipment" OR AB "mobility equipment"	Display
S19	TI "assistive equipment" OR AB "assistive equipment"	Display
S18	TI "assistive devices" OR AB "assistive devices"	Display
S17	(MM "Assistive Technology Devices")	Display
S16	S1 OR S2 OR S3 OR S4 OR S5 OR S6 OR S7 OR S8 OR S9 OR S10 OR S11 OR S12 OR S13 OR S14 OR S15	Display
S15	TI ((veteran* and (old* or home* or retire*))) OR AB ((veteran* and (old* or home* or retire*)))	Display
S14	TI geriatric* OR AB geriatric*	Display
S13	TI ((Resident* and (old* or home* or retirement or nursing))) OR AB ((Resident* and (old* or home* or retirement or nursing)))	Display
S12	TI "end of life" OR AB "end of life"	Display
S11	TI retirement OR AB retirement	Display
S10	TI pensioners OR AB pensioners	Display
S9	TI old* age* OR AB old* age*	Display
S8	TI "older women" OR AB "older women"	Display
S7	TI "older men" OR AB "older men"	Display
S6	TI "older adult*" OR AB "older adult*"	Display
S5	TI "older people" OR AB "older people"	Display
S4	TI elderly OR AB elderly	Display
S3	(MM "Frail Elderly")	Display
S2	(MH "Aged, 80 and Over")	Display
S1	(MM "Aged")	Display

## APPENDIX 4 COCHRANE LIBRARY SEARCH STRATEGY

Search Name:

Date Run: 31/07/18 14:52:15.684

Description:

ID Search Hits

#1 MeSH descriptor: [Aged] explode all trees 1381

#2 MeSH descriptor: [Aged, 80 and over] explode all trees 176

#3 MeSH descriptor: [Frail Elderly] explode all trees 707

#4 elderly:ti,ab 23565

#5 "older people":ti,ab 2886

#6 "older adult*":ti,ab 8626

#7 "older men":ti,ab 1070

#8 "older women":ti,ab 1656

#9 "old* age*":ti,ab 3327

#10 pensioners:ti,ab 23

#11 retirement:ti,ab 270

#12 "end of life":ti,ab 923

#13 (Resident* and (old* or home* or retirement or nursing)):ti,ab 3257

#14 geriatric*:ti,ab 4492

#15 (veteran* and (old* or home* or retire*)):ti,ab 930

#16 #1 or #2 or #3 or #4 or #5 or #6 or #7 or #8 or #9 or #10 or #11 or #12 or #13 or #14 or #15 43904

#17 MeSH descriptor: [Self‐Help Devices] explode all trees 440

#18 "assistive devices":ti,ab 154

#19 "assistive equipment":ti,ab 5

#20 "mobility equipment":ti,ab 0

#21 "mobility device*":ti,ab 24

#22 "mobility aid*":ti,ab 22

#23 motility:ti,ab 3258

#24 (walking near/2 (device* or aid* or equipment)):ti,ab 233

#25 cane*:ti,ab 262

#26 crutches:ti,ab 101

#27 "walking stick*":ti,ab 11

#28 (Adapt* near/3 (cars or transport or vehicles)):ti,ab 7

#29 (Adapt* near/3 (home* or house*)):ti,ab 103

#30 Wheelchair*:ti,ab 564

#31 scooter*:ti,ab 11

#32 "transfer device*":ti,ab 8

#33 (communication near (aid* or device*)):ti,ab 208

#34 MeSH descriptor: [Optical Devices] explode all trees 3992

#35 MeSH descriptor: [Hearing Aids] explode all trees 489

#36 eyeglasses:ti,ab 52

#37 glasses:ti,ab 619

#38 spectacles:ti,ab 386

#39 "hearing device*":ti,ab 24

#40 "hearing aid*":ti,ab 571

#41 "vision aid*":ti,ab 44

#42 ((Adapt* or adjust*) near/3 (door* or entry or exit)):ti,ab 31

#43 "Stair lift*":ti,ab 1

#44 "stair climbing":ti,ab 343

#45 stairs:ti,ab 450

#46 "stair rails":ti,ab 1

#47 "shallow steps":ti,ab 0

#48 (ramp or ramps):ti,ab 474

#49 MeSH descriptor: [Home Care Services] explode all trees 2709

#50 "home care service*":ti,ab 123

#51 "home support service*":ti,ab 3

#52 "home visit*":ti,ab 2466

#53 "community services":ti,ab 252

#54 shopping:ti,ab 257

#55 "house help":ti,ab 0

#56 "home help":ti,ab 46

#57 (food near (preparation or assistance or help or service or delivery)):ti,ab 432

#58 (meal* near/3 (provision or assistance or help or service* or preparation or delivery)):ti,ab 181

#59 homemaking:ti,ab 3

#60 housekeeping:ti,ab 65

#61 ((household or routine) near (jobs or tasks or chores)):ti,ab 74

#62 bathing:ti,ab 531

#63 grooming:ti,ab 58

#64 personal hygiene:ti,ab 257

#65 toileting:ti,ab 122

#66 "foot care":ti,ab 161

#67 (medication near/2 reminders):ti,ab 95

#68 (kitchen or bathroom or bedroom):ti,ab 400

#69 #17 or #17 or #18 or #19 or #20 or #21 or #22 or #23 or #24 or #25 or #26 or #27 or #28 or #29 or #30 or #31 or #32 or #33 or #34 or #35 or #36 or #37 or #38 or #39 or #40 or #41 or #42 or #43 or #44 or #45 or #46 or #47 or #48 or #49 or #50 or #51 or #52 or #53 or #54 or #55 or #56 or #57 or #58 or #59 or #60 or #61 or #62 or #63 or #64 or #65 or #66 or #67 or #68 19187

#70 #16 and #69 1814

## APPENDIX 5 EMBASE SEARCH STRATEGY

Database: Embase <1974 to 2018 July 30>

Search Strategy:

‐‐‐‐‐‐‐‐‐‐‐‐‐‐‐‐‐‐‐‐‐‐‐‐‐‐‐‐‐‐‐‐‐‐‐‐‐‐‐‐‐‐‐‐‐‐‐‐‐‐‐‐‐‐‐‐‐‐‐‐‐‐‐‐‐‐‐‐‐‐‐‐‐‐‐‐‐‐‐‐

1 Frail Elderly/(8986)

2 elderly.ti,ab. (307606)

3 older people.ti,ab. (28244)

4 older adult*.ti,ab. (77298)

5 older men.ti,ab. (10061)

6 older women.ti,ab. (16183)

7 old* age*.ti,ab. (91882)

8 pensioners.ti,ab. (1069)

9 retirement.ti,ab. (13626)

10 "end of life".ti,ab. (26578)

11 (Resident* and (old* or home* or retirement or nursing)).ti,ab. (50626)

12 geriatric*.ti,ab. (66412)

13 (veteran* and (old* or home* or retire*)).ti,ab. (7328)

14 or/1‐13 (597440)

15 self help device/(686)

16 orthopedic equipment/(7113)

17 assistive devices.ti,ab. (2030)

18 assistive equipment.ti,ab. (67)

19 mobility equipment.ti,ab. (40)

20 mobility device*.ti,ab. (366)

21 mobility aid*.ti,ab. (423)

22 motility.ti,ab. (107762)

23 (walking adj2 (device* or aid* or equipment)).ti,ab. (1867)

24 cane*.ti,ab. (7668)

25 crutch/(907)

26 crutches.ti,ab. (1592)

27 walking stick*.ti,ab. (314)

28 (Adapt* adj3 (cars or transport or vehicles)).ti,ab. (570)

29 (Adapt* adj3 (home* or house*)).ti,ab. (1916)

30 Wheelchair*.ti,ab. (9414)

31 scooter*.ti,ab. (455)

32 transfer device*.ti,ab. (324)

33 (communication adj (aid* or device*)).ti,ab. (1123)

34 eyeglasses.ti,ab. (737)

35 glasses.ti,ab. (9279)

36 spectacles.ti,ab. (2696)

37 exp hearing aid/(24552)

38 hearing aid*.ti,ab. (9542)

39 hearing device*.ti,ab. (588)

40 vision aid*.ti,ab. (467)

41 ((Adapt* or adjust*) adj3 (door* or entry or exit)).ti,ab. (302)

42 Stair lift*.ti,ab. (5)

43 stair climbing.ti,ab. (1871)

44 stairs.ti,ab. (4629)

45 stair rails.ti,ab. (4)

46 shallow steps.ti,ab. (1)

47 (ramp or ramps).ti,ab. (8753)

48 exp home care/(67776)

49 home care service*.ti,ab. (1915)

50 home support service*.ti,ab. (62)

51 home visit*.ti,ab. (9825)

52 community services.ti,ab. (3077)

53 shopping.ti,ab. (4729)

54 house help.ti,ab. (3)

55 home help.ti,ab. (526)

56 (food adj (preparation or assistance or help or service or delivery)).ti,ab. (4484)

57 (meal* adj3 (provision or assistance or help or service* or preparation or delivery)).ti,ab. (1594)

58 homemaking.ti,ab. (141)

59 housekeeping.ti,ab. (10998)

60 ((household or ktichen or routine) adj (jobs or tasks or chores)).ti,ab. (1136)

61 bathing.ti,ab. (11145)

62 grooming.ti,ab. (6112)

63 personal hygiene.ti,ab. (2422)

64 toileting.ti,ab. (1310)

65 foot care.ti,ab. (1788)

66 (medication adj2 reminders).ti,ab. (242)

67 (kitchen or bathroom or bedroom).ti,ab. (7945)

68 or/15‐67 (322386)

69 daily life activity/(78117)

70 Human Activities/(3185)

71 independent living/(3089)

72 recreation/(18512)

73 shopping/(1911)

74 social participation/(4612)

75 "activities of daily living".ti,ab. (30943)

76 "quality of life".ti,ab. (359513)

77 independence.ti,ab. (47534)

78 wellbeing.ti,ab. (18483)

79 social life.ti,ab. (5685)

80 social participation.ti,ab. (2771)

81 happiness.ti,ab. (6798)

82 happier.ti,ab. (909)

83 mental health.ti,ab. (147320)

84 functional ability.ti,ab. (6217)

85 depression.ti,ab. (400172)

86 cognitive.ti,ab. (416634)

87 sensory function*.ti,ab. (4984)

88 pain.ti,ab. (796354)

89 distress.ti,ab. (135545)

90 vitality.ti,ab. (14572)

91 energy.ti,ab. (546309)

92 fatigue.ti,ab. (127854)

93 tiredness.ti,ab. (5785)

94 self care.ti,ab. (20633)

95 self efficacy.ti,ab. (26490)

96 mobility.ti,ab. (142390)

97 community life.ti,ab. (577)

98 security.ti,ab. (47760)

99 relationships.ti,ab. (378690)

100 satisfaction.ti,ab. (157439)

101 adherence.ti,ab. (146634)

102 reablement.ti,ab. (63)

103 institutionali?ation.ti,ab. (5821)

104 or/69‐103 (3385736)

105 "systematic review"/(174301)

106 systematic*.ti,ab. (453751)

107 (meta‐analysis or metaanalysis).ti,ab. (148557)

108 (review* and (literature or studies or trials)).ab. (880084)

109 review.ti. (455568)

110 (evidence adj2 synthesi*).ti,ab. (6614)

111 overview.ti,ab. (167411)

112 pubmed.ab. (104333)

113 medline.ab. (118298)

114 or/106‐113 (1650752)

115 exp controlled clinical trial/(694556)

116 randomized.ti,ab. (638306)

117 randomly.ab. (384901)

118 trial.ti,ab. (727369)

119 groups.ab. (2487203)

120 usual care.ab. (18552)

121 or/115‐120 (3623667)

122 114 or 121 (5002455)

123 14 and 68 and 104 and 122 (3448)

***************************

## APPENDIX 6 PSYCINFO SEARCH STRATEGY

Database: PsycINFO <1806 to July Week 4 2018>

Search Strategy:

‐‐‐‐‐‐‐‐‐‐‐‐‐‐‐‐‐‐‐‐‐‐‐‐‐‐‐‐‐‐‐‐‐‐‐‐‐‐‐‐‐‐‐‐‐‐‐‐‐‐‐‐‐‐‐‐‐‐‐‐‐‐‐‐‐‐‐‐‐‐‐‐‐‐‐‐‐‐‐‐

1 elderly.ti,ab. (54174)

2 older people.ti,ab. (12110)

3 older adult*.ti,ab. (41115)

4 older men.ti,ab. (2189)

5 older women.ti,ab. (4109)

6 old* age*.ti,ab. (20699)

7 pensioners.ti,ab. (185)

8 retirement.ti,ab. (9183)

9 "end of life".ti,ab. (8017)

10 (Resident* and (old* or home* or retirement or nursing)).ti,ab. (20000)

11 geriatric*.ti,ab. (14314)

12 (veteran* and (old* or home* or retire*)).ti,ab. (3282)

13 or/1‐12 (152396)

14 assistive devices.ti,ab. (459)

15 assistive equipment.ti,ab. (12)

16 mobility equipment.ti,ab. (9)

17 mobility device*.ti,ab. (109)

18 mobility aid*.ti,ab. (96)

19 motility.ti,ab. (2556)

20 (walking adj2 (device* or aid* or equipment)).ti,ab. (158)

21 cane*.ti,ab. (672)

22 crutches.ti,ab. (96)

23 walking stick*.ti,ab. (38)

24 (Adapt* adj3 (cars or transport or vehicles)).ti,ab. (31)

25 (Adapt* adj3 (home* or house*)).ti,ab. (515)

26 Wheelchair*.ti,ab. (1489)

27 scooter*.ti,ab. (87)

28 transfer device*.ti,ab. (11)

29 (communication adj (aid* or device*)).ti,ab. (660)

30 Hearing aids/(1719)

31 eyeglasses.ti,ab. (133)

32 glasses.ti,ab. (1145)

33 spectacles.ti,ab. (464)

34 hearing device*.ti,ab. (93)

35 hearing aid*.ti,ab. (2472)

36 vision aid*.ti,ab. (53)

37 ((Adapt* or adjust*) adj3 (door* or entry or exit)).ti,ab. (116)

38 Stair lift*.ti,ab. (1)

39 stair climbing.ti,ab. (166)

40 stairs.ti,ab. (563)

41 stair rails.ti,ab. (1)

42 (ramp or ramps).ti,ab. (1042)

43 home care service*.ti,ab. (497)

44 home support service*.ti,ab. (30)

45 home visit*.ti,ab. (3811)

46 community services.ti,ab. (2062)

47 shopping.ti,ab. (5054)

48 house help.ti,ab. (1)

49 home help.ti,ab. (168)

50 (food adj (preparation or assistance or help or service or delivery)).ti,ab. (1559)

51 (meal* adj3 (provision or assistance or help or service* or preparation or delivery)).ti,ab. (474)

52 homemaking.ti,ab. (248)

53 housekeeping.ti,ab. (531)

54 ((household or ktichen or routine) adj (jobs or tasks or chores)).ti,ab. (995)

55 bathing.ti,ab. (838)

56 grooming.ti,ab. (4042)

57 personal hygiene.ti,ab. (415)

58 toileting.ti,ab. (515)

59 foot care.ti,ab. (123)

60 (medication adj2 reminders).ti,ab. (43)

61 (kitchen or bathroom or bedroom).ti,ab. (1808)

62 or/14‐61 (35245)

63 exp "Activities of Daily Living"/(5520)

64 "activities of daily living".ti,ab. (8352)

65 "quality of life".ti,ab. (59139)

66 "Quality of Life"/(37055)

67 independence.ti,ab. (24287)

68 wellbeing.ti,ab. (10013)

69 social life.ti,ab. (5978)

70 social participation.ti,ab. (2191)

71 happiness.ti,ab. (13763)

72 happier.ti,ab. (1484)

73 mental health.ti,ab. (157706)

74 functional ability.ti,ab. (1345)

75 depression.ti,ab. (219164)

76 cognitive.ti,ab. (370295)

77 sensory function*.ti,ab. (1264)

78 pain.ti,ab. (83792)

79 distress.ti,ab. (57532)

80 vitality.ti,ab. (3953)

81 energy.ti,ab. (33607)

82 fatigue.ti,ab. (22222)

83 tiredness.ti,ab. (1251)

84 self care.ti,ab. (8094)

85 self efficacy.ti,ab. (34497)

86 mobility.ti,ab. (16761)

87 community life.ti,ab. (1109)

88 security.ti,ab. (25264)

89 relationships.ti,ab. (258664)

90 satisfaction.ti,ab. (95291)

91 adherence.ti,ab. (24737)

92 reablement.ti,ab. (16)

93 institutionali?ation.ti,ab. (4822)

94 or/63‐93 (1229400)

95 systematic*.ti,ab. (108262)

96 (meta‐analysis or metaanalysis).ti,ab. (23648)

97 (review* and (literature or studies or trials)).ab. (201906)

98 review.ti. (142171)

99 (evidence adj2 synthesi*).ti,ab. (1195)

100 overview.ti,ab. (69147)

101 pubmed.ab. (9463)

102 medline.ab. (11506)

103 or/95‐102 (435243)

104 randomized.ti,ab. (63903)

105 randomly.ab. (66378)

106 trial.ti,ab. (94392)

107 groups.ab. (454479)

108 usual care.ab. (3673)

109 or/104‐108 (591784)

110 103 or 109 (980597)

111 13 and 62 and 94 and 110 (539)

***************************

## APPENDIX 7 SOCIAL POLICY AND PRACTICE SEARCH STRATEGY

Database: Social Policy and Practice <201804>

Search Strategy:

‐‐‐‐‐‐‐‐‐‐‐‐‐‐‐‐‐‐‐‐‐‐‐‐‐‐‐‐‐‐‐‐‐‐‐‐‐‐‐‐‐‐‐‐‐‐‐‐‐‐‐‐‐‐‐‐‐‐‐‐‐‐‐‐‐‐‐‐‐‐‐‐‐‐‐‐‐‐‐‐

1 elderly.ti,ab. (13983)

2 older people.ti,ab. (24556)

3 older adult*.ti,ab. (5159)

4 older men.ti,ab. (545)

5 older women.ti,ab. (1322)

6 old* age*.ti,ab. (4684)

7 pensioners.ti,ab. (876)

8 retirement.ti,ab. (3880)

9 "end of life".ti,ab. (1661)

10 (Resident* and (old* or home* or retirement or nursing)).ti,ab. (11855)

11 geriatric*.ti,ab. (2842)

12 (veteran* and (old* or home* or retire*)).ti,ab. (184)

13 or/1‐12 (55038)

14 assistive devices.ti,ab. (74)

15 assistive equipment.ti,ab. (8)

16 mobility equipment.ti,ab. (9)

17 mobility device*.ti,ab. (14)

18 mobility aid*.ti,ab. (17)

19 motility.ti,ab. (1)

20 (walking adj2 (device* or aid* or equipment)).ti,ab. (19)

21 cane*.ti,ab. (51)

22 crutches.ti,ab. (3)

23 walking stick*.ti,ab. (7)

24 (Adapt* adj3 (cars or transport or vehicles)).ti,ab. (13)

25 (Adapt* adj3 (home* or house*)).ti,ab. (310)

26 Wheelchair*.ti,ab. (316)

27 scooter*.ti,ab. (19)

28 transfer device*.ti,ab. (3)

29 (communication adj (aid* or device*)).ti,ab. (50)

30 [Hearing aids/] (0)

31 eyeglasses.ti,ab. (1)

32 glasses.ti,ab. (29)

33 spectacles.ti,ab. (17)

34 hearing device*.ti,ab. (1)

35 hearing aid*.ti,ab. (85)

36 vision aid*.ti,ab. (7)

37 ((Adapt* or adjust*) adj3 (door* or entry or exit)).ti,ab. (1)

38 Stair lift*.ti,ab. (5)

39 stair climbing.ti,ab. (10)

40 stairs.ti,ab. (70)

41 stair rails.ti,ab. (1)

42 (ramp or ramps).ti,ab. (40)

43 home care service*.ti,ab. (684)

44 home support service*.ti,ab. (49)

45 home visit*.ti,ab. (670)

46 community services.ti,ab. (1160)

47 shopping.ti,ab. (884)

48 house help.ti,ab. (0)

49 home help.ti,ab. (382)

50 (food adj (preparation or assistance or help or service or delivery)).ti,ab. (67)

51 (meal* adj3 (provision or assistance or help or service* or preparation or delivery)).ti,ab. (241)

52 homemaking.ti,ab. (12)

53 housekeeping.ti,ab. (40)

54 ((household or ktichen or routine) adj (jobs or tasks or chores)).ti,ab. (84)

55 bathing.ti,ab. (142)

56 grooming.ti,ab. (273)

57 personal hygiene.ti,ab. (47)

58 toileting.ti,ab. (61)

59 foot care.ti,ab. (26)

60 (medication adj2 reminders).ti,ab. (2)

61 (kitchen or bathroom or bedroom).ti,ab. (311)

62 or/14‐61 (5918)

63 [exp "Activities of Daily Living"/] (0)

64 "activities of daily living".ti,ab. (1300)

65 "quality of life".ti,ab. (6357)

66 ["Quality of Life"/] (0)

67 independence.ti,ab. (3826)

68 wellbeing.ti,ab. (4416)

69 social life.ti,ab. (337)

70 social participation.ti,ab. (347)

71 happiness.ti,ab. (405)

72 happier.ti,ab. (118)

73 mental health.ti,ab. (23759)

74 functional ability.ti,ab. (211)

75 depression.ti,ab. (7846)

76 cognitive.ti,ab. (7353)

77 sensory function*.ti,ab. (23)

78 pain.ti,ab. (1266)

79 distress.ti,ab. (2687)

80 vitality.ti,ab. (167)

81 energy.ti,ab. (2664)

82 fatigue.ti,ab. (320)

83 tiredness.ti,ab. (32)

84 self care.ti,ab. (659)

85 self efficacy.ti,ab. (842)

86 mobility.ti,ab. (2688)

87 community life.ti,ab. (244)

88 security.ti,ab. (5575)

89 relationships.ti,ab. (13857)

90 satisfaction.ti,ab. (4829)

91 adherence.ti,ab. (688)

92 reablement.ti,ab. (163)

93 institutionali?ation.ti,ab. (581)

94 or/63‐93 (74744)

95 systematic*.ti,ab. (5049)

96 (meta‐analysis or metaanalysis).ti,ab. (684)

97 (review* and (literature or studies or trials)).ab. (11073)

98 review.ti. (9726)

99 (evidence adj2 synthesi*).ti,ab. (113)

100 overview.ti,ab. (9080)

101 pubmed.ab. (196)

102 medline.ab. (471)

103 or/95‐102 (28484)

104 randomized.ti,ab. (634)

105 randomly.ab. (1161)

106 trial.ti,ab. (2435)

107 groups.ab. (25738)

108 usual care.ab. (230)

109 or/104‐108 (28684)

110 103 or 109 (54320)

111 13 and 62 and 94 and 110 (196)

***************************

## APPENDIX 8 SOCIAL SCIENCE CITATION INDEX (SSCI) VIA WEB OF SCIENCE SEARCH STRATEGY

SSCI via Web of Science

**Table:**

# 4	640	#3 AND #2 AND #1*Indexes* = *SSCI Timespan* = *1900‐2018*	
# 3	40,698	**TITLE:** ("systematic review" or "controlled trial")*Indexes* = *SSCI Timespan* = *1900‐2018*	
# 2	501,019	TI = (mobility or function* or "quality of life" or independence or wellbeing or social or activities)*Indexes* = *SSCI Timespan* = *1900‐2018*	
# 1	258,485	**TITLE:** (old* or elderly or aged)*Indexes* = *SSCI Timespan* = *1900‐2018*	

## APPENDIX 9 CODING TOOL

**Table:**

Category		Answer
Geographical information	WHO Regions	1. South Asia 2. Sub‐Saharan Africa 3. East Asia and Pacific 4. Europe and Central Asia 5. Latin America and Caribbean 6. Middle East and North Africa 7. North America
	World Bank Region (2019 FY)	1. Low income economies 2. Lower Middle income economies 3. Upper Middle income economies 4. High income economies
Study design	Design	1. Systematic reviews 2. ‐ RCT
	Publication status	1. Complete 2. On‐going (e.g., Protocols)
Population	Age Group	1. Includes <65 years 2. Includes >65 years 3. Includes >75 years 4. Includes >85 years
	Sex/Gender	1. Includes LGBTQ2+ 2. Proportion of females included in study
Health Conditions		1. Communicable Disease 2. Noncommunicable disease 3. Injury 4. Discharge from hospital 5. End‐of‐life 6. Physical Frailty 7. Social Frailty 8. ‐ Care Dependent
Intervention	General Social support services, systems and policies	1. Homemaking 2. Personal Care 3. Transportation 4. Family/Caregiver support 5. Befriending or friendly visits
	Health services, systems and policies	1. General health services for disease prevention 2. Health promotion services 3. Rehabilitation Services 4. Long‐term care services 5. Visiting Healthcare Professionals 6. Visiting Lay care providers
	Products and Technology	1. Personal mobility and transportation devices 2. Adaptations to physical environment
Outcome	Intrinsic Capacity	1. Mental 2. Sensory functions and pain 3. Neuro‐musculoskeletal function 4. Voice and speech 5. Cardiovascular, Haematological, Immune, Respiratory 6. Digestive, Endocrine, Metabolic functions 7. Genitourinary, Reproductive function 8. Integumentary system function
	Functional Ability	1. Basic needs 2. Learning and applying knowledge 3. Contribution 4. Mobility 5. Communication 6. Relationships
	Process and other	1. Falls 2. Cost (out of pocket) 3. Cost‐effectiveness 4. Satisfaction 5. Access 6. Safety 7. Caregiver outcomes 8. Adherence 9. Health Service Utilization 10. Quality of Life 11. Financial security and stability 12. Stigma
Setting		1. Residential home/apartment
		1. Long‐term care
		1. Independent living
		1. Assisted Living
Comparison		1. Usual Care 2. Other
Systematic Review quality		1. High 2. Moderate 3. Low 4. Critically Low 5. Protocol
PROGRESS		1. Place of residence 2. Race/Ethnicity 3. Occupation 4. Gender/sex 5. Religion 6. Education 7. Socioeconomic status 8. Social Capital
Gender Inequalities	Is there an assessment of effects by sex/gender	1. Yes 2. No 3. Planned but not reported
Other inequalities	Is there an assessment of effects by other characteristics, for example, socioeconomic status, income, race/ethnicity, etc	1. Yes 2. No 3. Planned but not reported

## APPENDIX 10 LINK TO ONLINE INTERACTIVE EGM

The online interactive EGM is available at https://globalageing.cochrane.org/sites/globalageing.cochrane.org/files/public/uploads/ageing_egm_interactive_map_may5_20.html.
