# Supplementary material for: Health, social care and technological interventions to improve functional ability of older adults living at home: An evidence and gap map
Source: Campbell Syst Rev. 2021 Jul 7;17(3):e1175. doi: 10.1002/cl2.1175 (PMC8988637; doi:10.1002/cl2.1175)
Supplement: Supplementary file 3 — Supporting information [file CL2-17-e1175-s001.html]

EPPI-Mapper


X

- Filters
- Hide Headers
  Show Headers
- Fullscreen
  Exit Fullscreen
- About
- Submit a Study
- View Records

|  |  |  |
| --- | --- | --- |
|  | Health, Social and Technological Interventions to Improve Functional Ability of Older Adults: An Evidence and Gap Map |  |

Generated using v.2.0.1 of the EPPI-Mapper
powered by EPPI Reviewer
and created with


by the
Digital Solution Foundry team.
